# Difluoroalkylation of Anilines via Photoinduced Methods

**DOI:** 10.1021/acs.joc.3c01298

**Published:** 2023-08-16

**Authors:** Albert Gallego-Gamo, Albert Granados, Roser Pleixats, Carolina Gimbert-Suriñach, Adelina Vallribera

**Affiliations:** Department of Chemistry and Centro de Innovación en Química Avanzada (ORFEO−CINQA), Universitat Autònoma de Barcelona, Cerdanyola del Vallès 08193, Barcelona, Spain

## Abstract

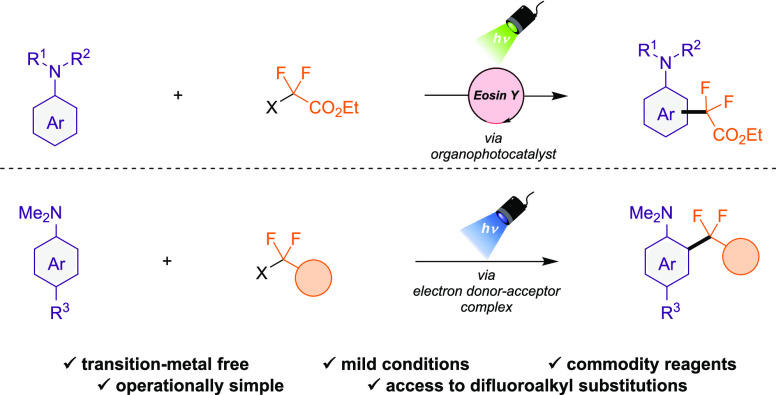

The development of sustainable and mild protocols for the fluoroalkylation of organic backbones is of current interest in chemical organic synthesis. Herein, we present operationally simple and practical transition-metal-free methods for the preparation of difluoroalkyl anilines. First, a visible-light organophotocatalytic system working via oxidative quenching is described, providing access to a wide range of difluoroalkyl anilines under mild conditions. In addition, the formation of an unprecedented electron donor–acceptor (EDA) complex between anilines and ethyl difluoroiodoacetate is reported and exploited as an alternative, efficient, and straightforward strategy to prepare difluoroalkyl derivatives.

## Introduction

**Scheme 1:**
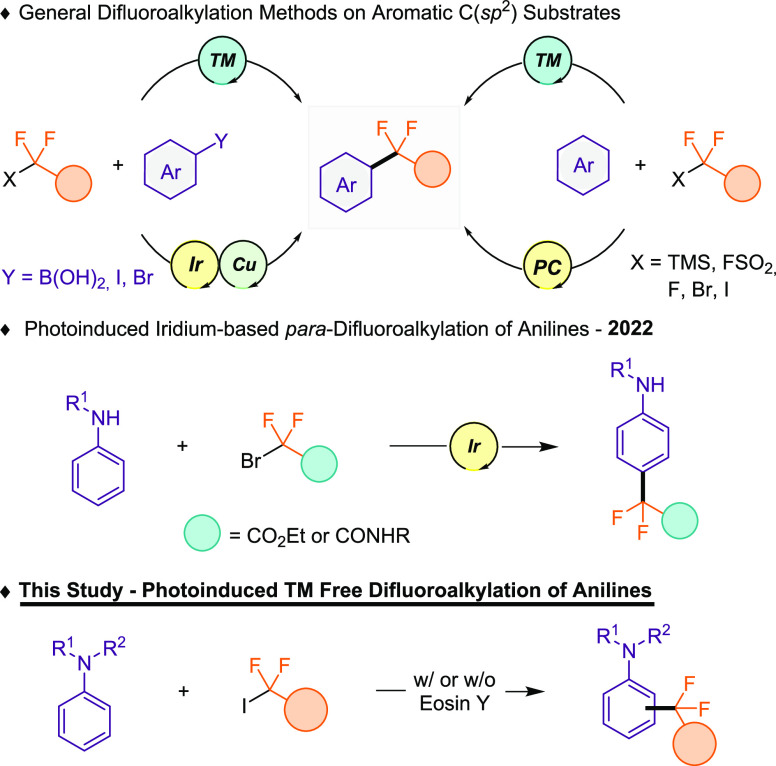
Precedents in Difluoroalkylation Protocols of Arenes and This Work (TM: Transition Metal; PC: Photocatalyst)

The presence of tethered fluorinated groups in bioactive molecules is a well-known strategy for altering their chemical properties, including conformational bias, reactivity, stability, and lipophilicity.^1^ Thus, in the last decades, synthetic organic chemists have done great efforts to develop efficient methods for the introduction of fluorinated motifs in organic architectures.^2^ Nowadays, around 20% of the marketed drugs are fluoropharmaceuticals, and in 2019, 13 new fluorinated compounds were approved by US Food and Drug Administration (FDA) accounting for 41% of all small-molecule drugs.^3^ The trifluoromethyl group has been one of the most studied fluorinated groups in medicinal chemistry;^4^ therefore, its introduction to organic molecules has been widely explored.^5^ In addition, in recent years, the *gem*-difluoro group is emerging as an equally interesting motif because of its pharmacological properties as a carbonyl or sulfonyl groups bioisostere, modulator of lipophilicity, as well as its oxidative stability.^6^ The *gem*-difluoroalkylation of arenes can be provided by transition-metal,^7^ photoredox,^8^ or metallaphotoredox^9^ catalysis approaches (Scheme 1, top). All of these methods utilize common difluoroalkylating agents, such as FSO_2_CF_2_CO_2_Me, TMSCF_2_CO_2_R, or XCF_2_Z (X = Br, I; Z = CO_2_R or P(O)(OEt)_2_).^10^ Interestingly, the installation of these motifs can offer further opportunities for derivatization, but also they can yield the corresponding difluoromethylgroup,^8c^ which can be used as a bioisostere of hydroxyl, mercapto, or amino groups.^11^

Herein, we present two methods for the photoinduced difluoroalkylation of anilines, the first based on a redox-neutral approach using an organic photocatalyst and the latest via electron donor–acceptor (EDA) complex formation. Many arene difluoroalkylation reports utilize transition-metal (TM) catalysts,^7^ such as Ni or Pd from both C–H or C–Y substitution (Scheme 1, top, Y = B(OH)_2_, I, Br). Also, photoinduced methods^8,9^ provide access to the difluoroalkylated arenes through simple photocatalysts or by synergistic transition-metal and photocatalyst dual catalysis. For example, Molander^9b^ explored the installation of the *gem*-difluoro group using boronic acids *via* Ir/Cu combination. Organic photocatalysts can be extensively modified, and they are also cheaper and more sustainable than transition-metal-based photocatalysts.^12^ In this vein of sustainability, the development of synthetic methods driven by photoactive EDA complexes is also very attractive because they are able to trigger single-electron process reactions without the presence of an exogenous photocatalyst.^13^ Last year, an efficient protocol for the difluoroalkylation of anilines was reported via an iridium-based photoredox approach (Scheme 1, middle), although the method uses large amounts of difluoroalkylating reagent and a fluorinated base.^14^ Assembling the *gem*-difluoro unit into anilines is an exciting area of research because these organic backbones are present in many synthetic routes for the elaboration of more sophisticated medicinal chemistry analogs, such as bioactive molecules and natural products.^15^

## Results and Discussion

First, to investigate the feasibility of the organophotoredox difluoroalkylation method, we selected Eosin Y (*E* (PC^+^/*PC) = −1.66 V vs Fc^+^/Fc)^16^ as a model photocatalyst, and 4-bromo-*N*,*N*-dimethylaniline (**1a**) and ethyl bromodifluoroacetate (**2a**) as model substrates (Table 1). We observed that 1 mol % of Eosin Y was the optimal loading to yield **3** (Table 1 entries 1–4) in DMF as a solvent and using K_2_CO_3_ as a base under green Kessil (λ_max_ = 525 nm) lamp irradiation. Subsequently, we observed a higher efficiency to form **3** when using the radical precursor **2b** (Table 1, entry 5), which has a higher reduction potential (*E*_red_ = −1.67 V vs Fc^+^/Fc)^16^ than **2a** (*E*_red_ = −1.94 V vs Fc^+^/Fc).^16^ The addition of [(*n*-Bu)_4_N]I (TBAI) contributed to better base solubility, providing better efficacy (Table 1, entries 5–6), and the presence of base and organophotocatalyst is also crucial for this transformation, although we observed moderate reactivity in both cases (Table 1, entries 7–8). Finally, the photochemical nature of this transformation was confirmed when control experiments in the absence of light showed no conversion to product **3** (Table 1, entry 9).

**Table 1:**
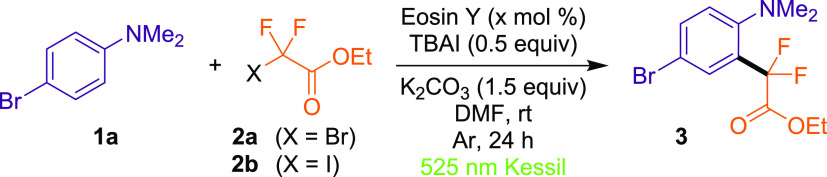
Optimization of Reaction Conditions^a^

entry	radical precursor	eosin Y loading	yield of **3** (%)^b^
1	**2a**	10	25
2	**2a**	5	40
3	**2a**	2.5	48
4	**2a**	1	52
**5**	**2b**	**1**	**79 (63)**^c^
6	**2b**	1	70^d^
7	**2b**	1	33^e^
8	**2b**	0	38
9	**2b**	1	0^f^

a Reaction conditions: aniline **1a** (0.3 mmol), **2** (0.4 mmol), Eosin Y (1 mol %) in DMF (1.0 mL), 24 h irradiation with a 525 nm Kessil lamp.

b Yields were determined by ^1^H NMR analysis.

c Isolated yield.

d No TBAI as a phase transfer agent in the reaction.

e No base added.

f Reaction in the absence of light irradiation.

With the suitable reaction conditions determined, we examined the scope of this difluoroalkylation of anilines (Table 2).

**Table 2:**
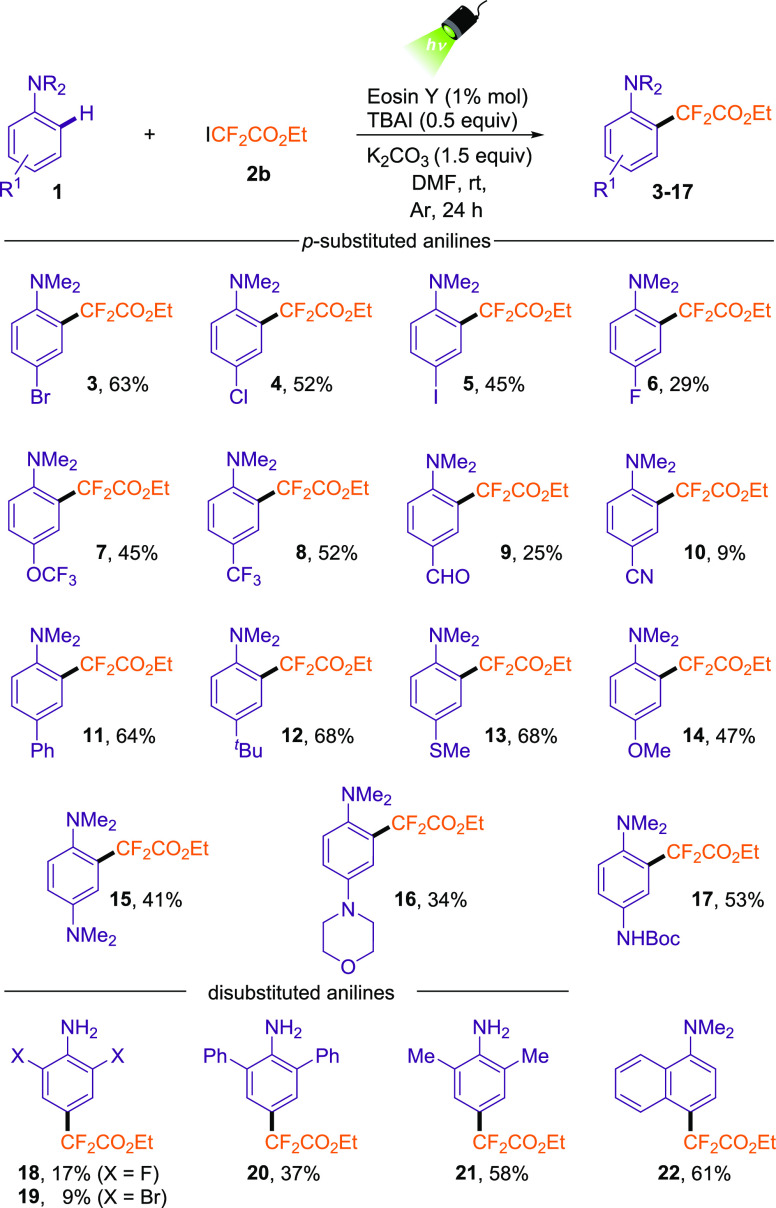
Evaluation of Substrate Scope^a^

a Reaction conditions: aniline **1** (0.3 mmol), **2b** (0.4 mmol), Eosin Y (1 mol %) in DMF (1.0 mL), 24 h irradiation with a 525 nm Kessil lamp.

First, the scope using different *N*,*N*-dimethylanilines having substituents at the *para* position was evaluated. In general, this transformation is amenable to a wide range of anilines containing electron-rich and moderate electron-withdrawing groups. The presence of halogens in this position allowed the formation of the desired difluoroalkylated product in good yields (**3**-**6**). Interestingly, the *para*-iodo *N*,*N*-dimethylaniline also proved to be a competent substrate for this transformation, providing **5**, which could serve as an excellent feedstock for cross-coupling reactions in further transformations. Next, the medicinally relevant trifluoromethyl ether and trifluoromethyl substituents were also competent substrates for this transformation (**7** and **8**). However, this methodology did not present high reactivity in the presence of aldehyde (**9**) and nitrile (**10**), indicating low yields because the starting material was not fully consumed. In general, electronically rich substituents, such as aryl (**11**), alkyl (**12**), methylthio (**13**), alkoxy (**14**), and amino groups (**15**-**17**), can be accommodated as well in this transformation. In the cases of more activated anilines (**14**-**17**), we also detected the formation of the *meta*-*N*,*N*-dimethylaniline difluoroalkylated product and the 2,5-bis(difluoroalkyl)-substituted compounds.

Then, we further evaluated some disubstituted anilines using our standard reaction conditions. Once again, deactivated anilines provide the difluoroalkylated product in low yield (**18** and **19**). On the other hand, the installation of the difluoroalkylated ester was amenable to 2,6-disubstituted aryl (**20**) and alkyl (**21**) anilines. Finally, the difluoroalkylation of *N,N*-dimethylnaphthalen-1-amine provided **22** in 61% yield.

**Scheme 2:**
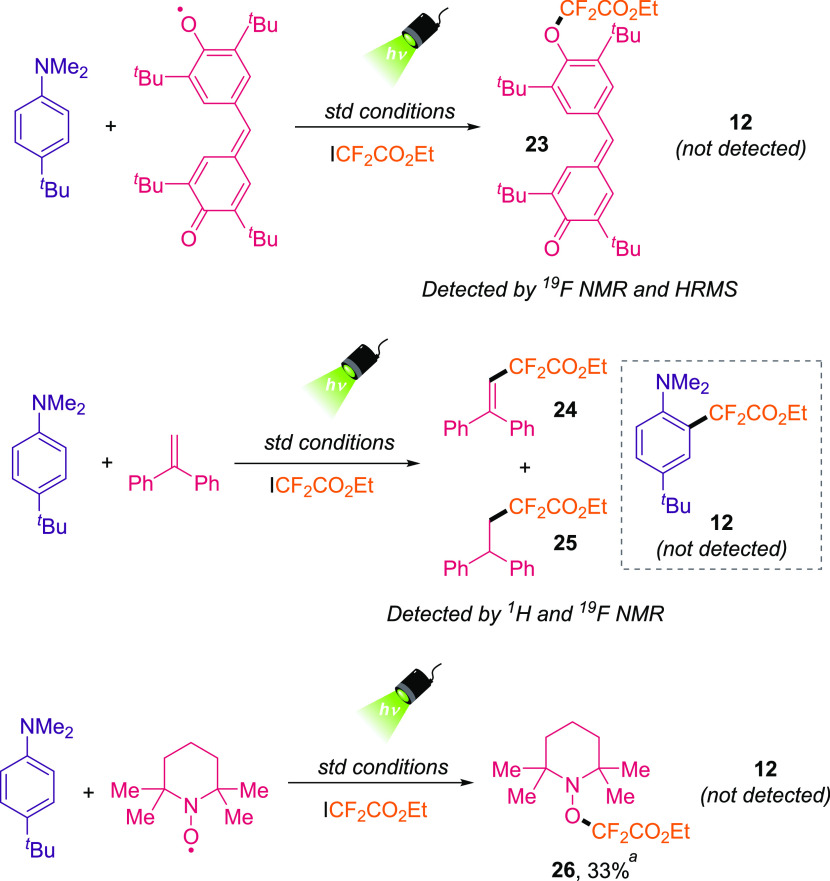
Radical-Trapping Experiments Using Galvinoxyl, 1,1-Diphenylethene and TEMPO with the Optimized Standard (std) Reaction Conditions Determined by ^1^H NMR.

Next, we sought to investigate the operative mechanism of this organophotoinduced transformation. To this end, we performed radical trapping studies under the standard reaction conditions (Scheme 2, see also the Supporting Information). The addition of the Galvinoxyl free radical totally inhibited the product formation, and we detected by ^19^F NMR and HRMS the formation of the difluoroalkylated Galvinoxyl product (**23**). The reaction was also avoided in the presence of 1,1-diphenylethene as radical scavenger. Specifically, the difluoroalkylated compounds **24** and **25** were detected as major reaction products by ^1^H and ^19^F NMR. Additionally, the presence of 2,2,6,6-tetramethyl-1-piperidinyloxy (TEMPO) also constrains the formation of aniline **12**, and we could detect difluoroalkylated-TEMPO adduct **26** in 33% yield. All of these experiments support the generation of the difluoroalkyl radical during the reaction. In addition, the redox properties of all reaction components were analyzed by cyclic voltammetry experiments to assess the thermodynamically allowed single electron transfer (SET) processes (Tables S2 and S3 in the Supporting Information).

**Scheme 3:**
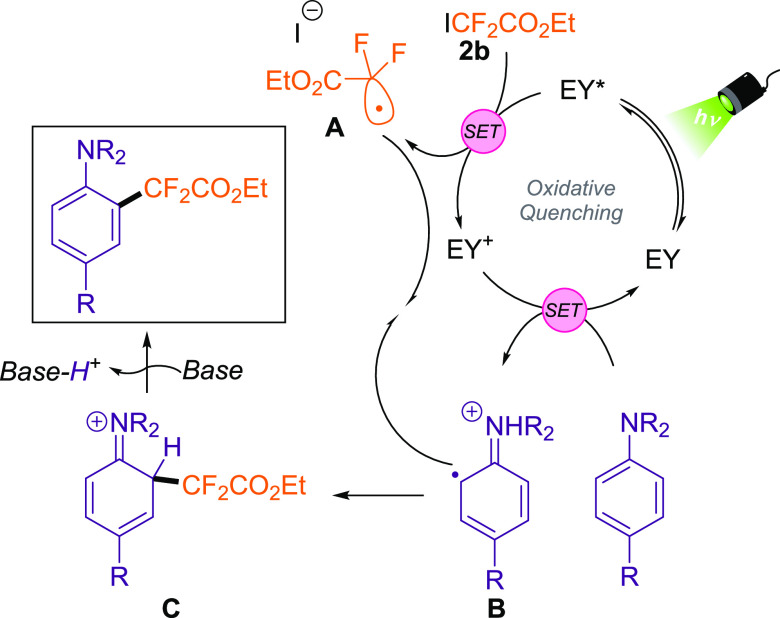
Proposed Mechanism for the Photoinduced Difluoroalkylation of Anilines Promoted by Eosin Y (EY) as Photocatalyst (SET: Single Electron Transfer)

Based on the mechanistic findings described herein, the substrate scope, the spectroscopic and electrochemical characterization of the reaction components,^16^ and reported literature,^14^ a plausible mechanism for this transformation is depicted in Scheme 3.

After photoirradiation of Eosin Y (EY) photocatalyst (*E* (PC^+^/*PC) = −1.66 V vs Fc^+^/Fc),^16^ a SET event from EY* to the fluorinated radical precursor **2b** (*E*_red_ = −1.67 V vs Fc^+^/Fc)^16^ affords the corresponding radical **A**. The generated oxidized EY^+^ undergoes a new SET with the corresponding aniline to recover the ground state of the photocatalyst (EY) and the radical cation **B**. This species collapses with radical **A** to generate the cationic **C** intermediate. The rearomatization of the phenyl ring in the presence of base provides the difluoroalkylated aniline.

At this stage, we explored the feasibility of the synthesis of the described difluoroalkylated anilines without the use of an exogenous photocatalyst. We envisioned anilines can act as electron donor molecules and **2b** as an electron-acceptor substrate. Thus, an electron donor–acceptor (EDA) complex can form if these two species efficiently interact, generating a new molecular aggregate in the ground state (Scheme 5). Further, this EDA complex can be activated by visible light to deliver a radical cationic species from aniline (**B**) and the reduced fluoroalkylated radical (**A**) pair. Control experiments in the absence of EY afforded product **3** in 38% yield, suggesting that the EDA pathway is indeed plausible (Table 1, entry 8). Thus, the next step was to study the potential of this route. Given the mild reaction conditions required to activate EDA complexes, their use has undoubtedly become a powerful strategy for the preparation of organic backbones in a sustainable manner.^13,17^

We started the study by exploring different solvents since the formation of EDA complexes is known to be directly related to solvent choice.^18^ We were delighted to observe a yield increase from 38% in DMF to 52% yield when DCM was used as a solvent in the formation of **3** (Table 3, entry 1). The examination of other solvents indicated that DMSO was the best option (Table 3, entry 2). Afterwards, we explored other bases, observing the highest yield using Na_2_CO_3_ (Table 3, entries 5–9). Finally, the use of 427 nm Kessil afforded the product in the highest yield (Table 1 entries 5, 10, and 11).

**Table 3:**
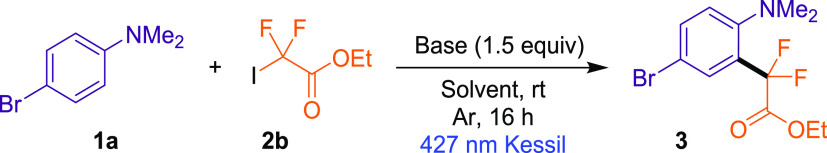
Optimization of Reaction Conditions via EDA Complex Strategy^a^

entry	solvent	base	yield of **3** (%)^b^
1	DCM	K_2_CO_3_	52
2	DMSO	K_2_CO_3_	70
3	DMA	K_2_CO_3_	60
4	MeCN	K_2_CO_3_	0
**5**	**DMSO**	**Na**_**2**_**CO**_**3**_	**79 (70)**^c^
6	DMSO	Cs_2_CO_3_	65
7	DMSO	K_3_PO_4_	46
8	DMSO	NaHCO_3_	63
9	DMSO	None	0
10	DMSO	Na_2_CO_3_	74^d^
11	DMSO	Na_2_CO_3_	0^e^

a Reaction conditions: aniline **1a** (0.3 mmol), **2b** (0.4 mmol, 1.3 equiv), base (1.5 equiv) in the indicated solvent (3 mL), 16 h irradiation with a 427 nm Kessil lamp.

b Yields were determined by ^1^H NMR analysis.

c Isolated yield.

d 456 nm Kessil lamp irradiation.

e Reaction in the dark.

Once we had the optimized conditions for this transformation via the generation of the EDA complex, we sought to explore different substituted anilines (Table 4). We were delighted to observe a more efficient reaction when using electron-deficient anilines compared to the reaction proceeding via Eosin Y. Thus, *para*-trifluoromethyl ether (**7**) and aldehyde (**9**) derivatives were isolated in moderate yields, 49 and 30%, respectively. Additionally, the presence of more electronically rich substituents in the aromatic ring of the anilines such as *para*-phenyl (**11**), *-tert*butyl (**12**), methoxy (**14**), and methyl (**21**) proved to be the best electron donor substrates for this transformation obtaining good to excellent yields. Particularly, when using 4-methoxy-*N*,*N-*dimethylaniline as substrate, we observed the formation of the dialkylated product **14** along with the bis(difluoroalkylated) aniline as a side product. In this example, we detected that after 1 h of irradiation, the intended monodifluoralkylated product **14** was formed in an 85% yield. Of note, the preparation of **12** was done at 1.0 mmol scale in high yield (80%), showcasing the efficiency of this transformation. Next, we examined *ortho*-, *meta*-substituted, and unbiased substrates (see products **23**-**26**). In general, we observed three regioisomers being the *para*-substituted the most predominant, which during the purification process underwent hydrolysis of the *gem*-difluoro moiety^19^ (see compound **29** in the Supporting Information). Interestingly, when using *p*-*tert*-butylaniline as the organic backbone in this transformation, the difluorinated indolin-2-one **27** was isolated in a good 55% yield. This experiment opens new molecular complexity in organofluorine chemical space without using any exogenous photocatalyst.

**Table 4:**
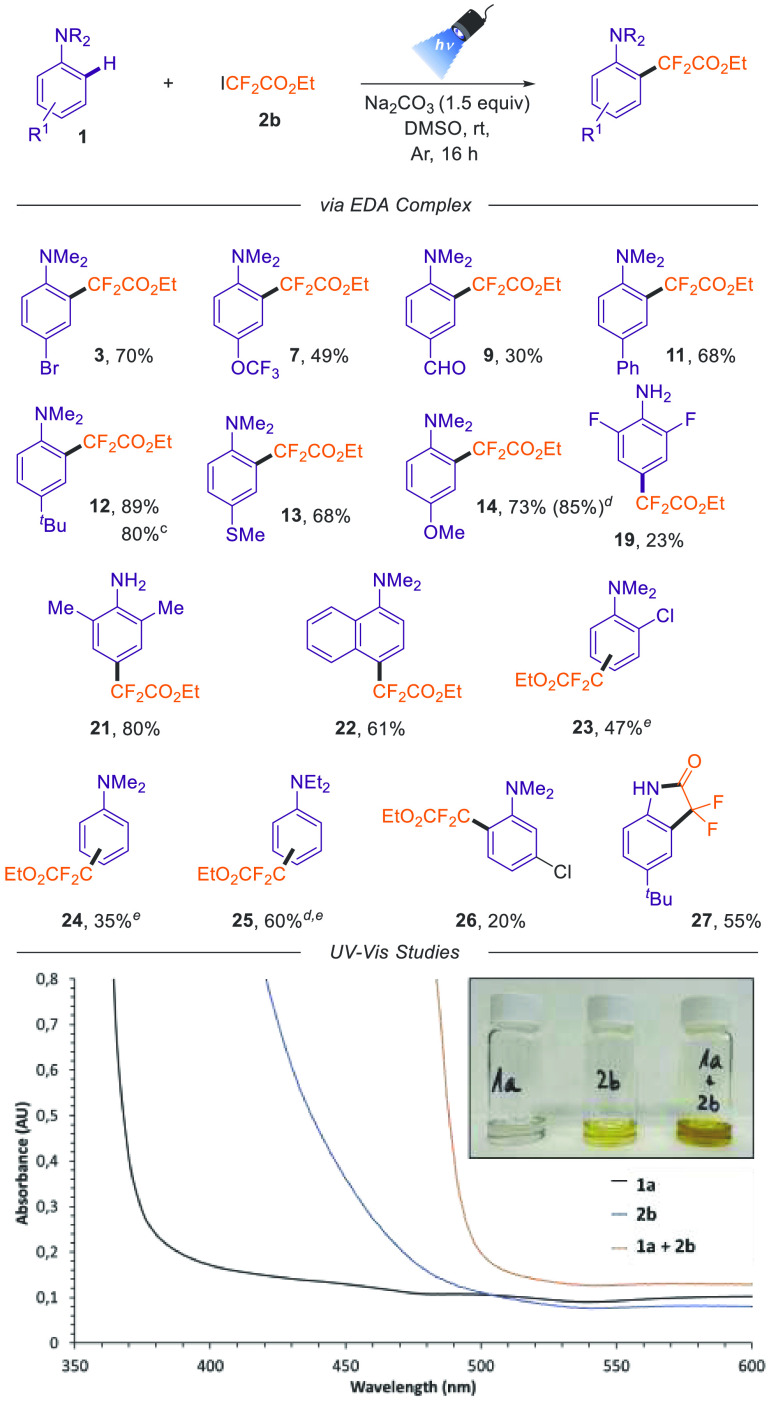
Substrate Scope for Difluoroalkylation of Anilines via EDA Complex Strategy^a^ and UV–Vis Studies^b^

a Reaction conditions: aniline (0.3 mmol), **2b** (0.4 mmol, 1.3 equiv), Na_2_CO_3_ (1.5 equiv) in DMSO (3 mL), 16 h irradiation with a 427 nm Kessil lamp.

b UV–vis absorption spectra and picture of individual reaction components and a combination thereof (see the Supporting Information for details).

c Isolated yield at 1.0 mmol scale.

d ^1^H NMR yield after 1 h of irradiation.

e Mixture of isomers (see the Experimental Section for details).

The exploration of other more electronically deficient anilines, including amides and *N*-Boc-protected anilines, resulted in only traces of product. These results support the mechanistic pathway through the EDA complex, whose formation is not favored with such electron-poor nitrogenated substrates.

**Scheme 4:**
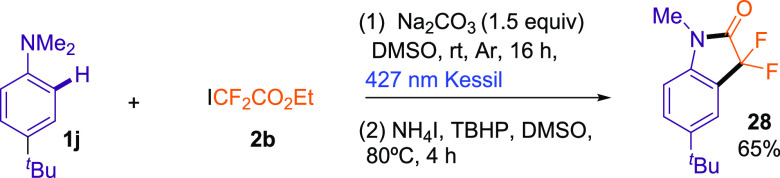
One-Pot Formation of 3,3-Difluoroindolin-2-one **28**

Then, we targeted the cyclization reaction from aniline **1j***via N*-Me bond scission in one-pot sequence (Scheme 4). After using our optimal conditions, we added NH_4_I and *tert*-butyl hydroperoxide (TBHP)^20^ and let the reaction to proceed for 4 h at 80 °C. To our delight, we isolate the corresponding compound **28** in a 65% yield.

Given the efficiency of this EDA complex strategy, we investigated the use of other fluoroalkyl iodides as radical precursors. Of note, we could extend our optimized reaction conditions for the preparation of difluorophosphonate, difluoroamide, and perfluorohexyl derivatives in moderate yield (Table 5).

**Table 5:**
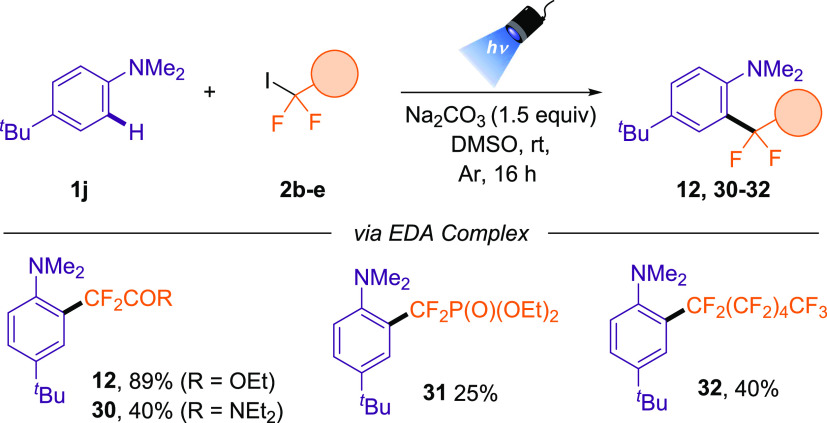
Substrate Scope for Difluoroalkylation of Aniline **1j** via EDA Complex Strategy Using Different Fluoroalkyl Iodides^a^

a Reaction conditions: aniline (0.3 mmol), **2b**–**e** (0.4 mmol, 1.3 equiv), Na_2_CO_3_ (1.5 equiv) in DMSO (3 mL), 16 h irradiation with a 427 nm Kessil lamp.

**Scheme 5:**
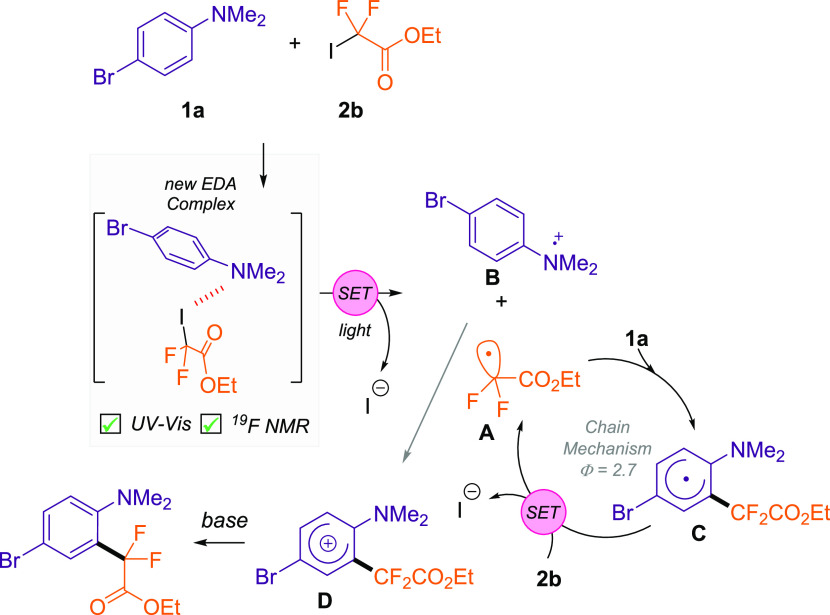
Proposed Mechanism for the Photoinduced Exogenous Photocatalyst Difluoroalkylation of Anilines

Next, we sought to study the mechanism of this transformation. The UV–vis absorption spectra of each of the reaction components and mixtures in DMSO evidenced the formation of an EDA complex between aniline **1a** and fluorinated **2b** (Table 4, bottom). Aniline **1a** (black line) presents an absorption band in the ultraviolet range and **2b** in the visible-light area (light gray line), while the mixture of reaction components exhibits a significant bathochromic shift with a visible-light absorption tailing in the 450–500 nm range. This bathochromic shift is in agreement with the visual appearance of the individuals and all of the reaction components mixture. The solution of **1a** is colorless and that of **1b** is slightly yellow, while the solution of all of the reaction components remains intense yellow (see the inset in Table 4, bottom). Additionally, the formation of this EDA complex is supported by ^19^F NMR titrations, which show a significant upfield shift of the fluorine resonance upon adding increasing amounts of aniline (Figure S3 in the Supporting Information). Photochemical quantum yield (Φ) experiments provided key information about the mechanism of this reaction. We measured Φ = 2.7, thus indicating that the radical chain scenario is operating this transformation. Given this experimental value and previous reports,^21^ we propose a radical chain mechanism initiated by a halogen bonding assisted EDA complex (Scheme 5). First, aniline **1a** and fluorinated **2b** meet in a new molecular aggregate in the ground state, which after photoirradiation undergoes SET. This event generates the fluorinated radical **A** and the cationic radical species **B**. Subsequently, **1a** reacts with **A** to yield radical species **C**. Then, this radical species reduces the fluorinated reagent **2b** and oxidized **D** is obtained. Finally, the base abstracts a proton to obtain the desired difluoroalkylated aniline. With the mechanistic findings described above, the reaction of radical pairs **A** and **B** to yield **D** is an unlikely process.

## Conclusions

In summary, we are presenting two complementary synthetic methods for the difluoroalkylation of anilines, avoiding the use of transition-metal photocatalysts. Our first protocol promotes the difluoroalkylation using Eosin Y as an organic photocatalyst under very mild conditions. The reaction works well when using electronically rich anilines. The mechanistic findings evidenced the generation of the ·CF_2_CO_2_Et radical as an intermediate. Additionally, we describe the formation of a new EDA complex formed by the combination of anilines and ethyl difluoroiodoacetate. The simple photoexcitation of this new molecular aggregate promotes the formation of difluoroalkylated arenes in an efficient manner with chemical yields up to 89%.

## Experimental Section

### General Information

All chemical transformations requiring an inert atmosphere were done using Schlenk line techniques with a 4- or 5-port dual-bank manifold. For the one-pot reaction that required heating, an oil bath was used as a heating source. The UV–vis spectra were recorded in a UV–vis spectrophotometer HP 8453 (Servei d’Anàlisi Química, UAB), at room temperature, with the appropriate solvent. The cyclic voltammetry experiments were performed with a BioLogic SP-50 Single Channel Potentiostat, in a one-compartment three-electrode setup using a glassy carbon disk as the working electrode (ø = 3 mm), platinum wire as the auxiliary electrode, and AgNO_3_/Ag (0.01 M AgNO_3_, 0.1 M [(Bu)_4_N]PF_6_ (TBAPF_6_), MeCN) as the reference electrode. Experiments were performed at room temperature using the appropriate solvent, degassing with Ar, using TBAPF_6_ as supporting electrolyte (0.1 M). All of the experiments were referred to ferrocene as an internal standard. Polishing of the working electrode has been done using an alumina polishing pad with a solution of 0.05 μm alumina in water (purchased from BAS INC.). For irradiation, commercially available Kessil PR160-purple LED lamp (30 W High Luminous DEX 2100 LED, λ_max_ = 390 nm), Kessil A160WE Tuna Blue LED lamp (40 W, λ_max_ = 456 nm), or a PR160-green LED lamp (44 W High Luminous DEX 2100 LED, λ_max_ = 525 nm) were placed 4 cm away from the reaction vials. The NMR experiments were performed in the *Servei de Ressonància Magnètica Nuclear*, UAB using the following Bruker Avance machines: DPX-250, DPX-360, NEO 300, NEO 400, or III 400SB. Structural assignments were made with additional information from gCOSY, gHSQC, and gHMBC experiments. The HRMS (ESI+) was done by the *Servei d’Anàlisi Química*, UAB using a Bruker micrOTOF-QII mass spectrometer (fly time analyzer) through positive electrospray ionization. All reagents and solvents were purchased from Sigma-Aldrich/Merck and BLDPharm except for 2,6-difluoroaniline, which was purchased from FluoroChem. All compounds were used as received, except 2,6-dimethylaniline and 2,6-difluoroaniline, which were distilled under reduced pressure.

### General Procedure 1 for the Difluoroalkylation of Anilines via Eosin Y (GP1)

In a 4 mL vial equipped with a magnetic stirring bar, Eosin Y (1.9 mg, 0.003 mmol, 0.01 equiv), the corresponding aniline (0.3 mmol, 1 equiv), K_2_CO_3_ (62.2 mg, 0.45 mmol, 1.5 equiv), TBAI (55.4 mg, 0.15 mmol, 0.5 equiv), and ICF_2_COOEt (51 μL, 0.4 mmol, 1.3 equiv) were added. The vial was closed with a screw cap provided with a rubber septum and degassed by alternating vacuum evacuation and N_2_ backfill. Then, 1 mL of anhydrous DMF was added, and the mixture was degassed again by Ar bubbling. After 5 min of degassing, the vial was well sealed with Parafilm. The reaction mixture was stirred under Ar and irradiated by a 525 nm LED (PR160L Kessil) at room temperature. After 24 h, the mixture was diluted with water and extracted with EtOAc (20 mL × 3). The organics were combined, washed with water (15 mL) and brine (15 mL × 2), and finally dried over anhydrous Na_2_SO_4_. After solvent removal under high vacuum, the product was purified by flash column chromatography through silica gel.

### General Procedure for the Difluoroalkylation of Anilines via EDA Complex (GP2)

In a 4 mL vial equipped with a magnetic stirring bar, the corresponding aniline (0.3 mmol, 1 equiv), Na_2_CO_3_ (47.7 mg, 0.45 mmol, 1.5 equiv), and ICF_2_COOEt (51 μL, 0.4 mmol, 1.3 equiv) were added. The vial was closed with a screw cap provided with a rubber septum and degassed by alternating vacuum evacuation and N_2_ backfill. Then, 1 mL of anhydrous DMSO was added, and the mixture was degassed again by Ar bubbling. After 5 min of degassing, the vial was well sealed with Parafilm. The reaction mixture was stirred under Ar and irradiated by a 427 nm LED (PR160L Kessil) at room temperature. After 16 h, the mixture was diluted with water and extracted with EtOAc (20 mL × 3). The organics were combined, washed with water (15 mL) and brine (15 mL × 2), and finally dried over anhydrous Na_2_SO_4_. After solvent removal under high vacuum, the product was purified by flash column chromatography through silica gel.

### Procedure for the Difluoroalkylation via EDA Complex at 1.0 mmol Scale

In a 20 mL Schlenk equipped with a magnetic stirring bar, 4-(*tert*-butyl)-*N*,*N*-dimethylaniline (177 mg, 1.0 mmol, 1 equiv), Na_2_CO_3_ (157 mg, 1.5 mmol, 1.5 equiv), and ICF_2_COOEt (170 μL, 1.3 mmol, 1.3 equiv) were added. The Schlenk was closed with a septum and degassed by alternating vacuum evacuation and N_2_ backfill. Then, 10 mL of anhydrous DMSO was added, and the mixture was degassed again by Ar bubbling. Then, the vial was well sealed with Parafilm. The reaction mixture was stirred under Ar and irradiated by two 427 nm LEDs (PR160L Kessil) at room temperature. After 16 h, the mixture was diluted with water and extracted with EtOAc (25 mL). The organics were combined, washed with water (15 mL) and brine (15 mL × 3), and finally dried over anhydrous Na_2_SO_4_. After solvent removal under a high vacuum, the product was purified by flash column chromatography through silica gel, yielding **12** in 80% yield (239 mg, 0.08 mmol).

#### Ethyl 2-(5-Bromo-2-(dimethylamino)phenyl)-2,2-difluoroacetate **(3)**(22)

Colorless oil (60.9 mg, 0.19 mmol, 63% yield from 60 mg of *N,N*-dimethyl-4-bromoaniline following GP1; 67.0 mg, 0.21 mmol, 70% yield from 60 mg of *N*,*N*-dimethyl-4-bromoaniline following GP2); Rf = 0.68 (hexane:ethyl acetate, 9.5:0.5). ^**1**^**H NMR** (400 MHz, CDCl_3_), δ (ppm): 7.81 (d, ^4^*J* = 4.0 Hz, 1H), 7.59 (dd, ^3^*J* = 16 Hz, ^4^*J* = 4.0 Hz, 1H), 7.20 (d, ^3^*J*_H,H_ = 16 Hz, 1 H), 4.30 (q, ^3^*J*_H,H_ = 12.0 Hz, 2H), 2.55 (s, 6 H), 1.30 (t ^3^*J*_H,H_ = 12.0 Hz, 3H); ^**13**^**C{**^**1**^**H} NMR** (100 MHz, CDCl_3_), δ (ppm): 163.9 (t, ^2^*J*_F,C_ = 34.0 Hz), 151.9 (t, ^3^*J*_F,C_ = 5.0 Hz), 135.3, 133.3 (t, ^2^*J*_F,C_ = 22.0 Hz), 129.8 (t, ^3^*J*_F,C_ = 7.0 Hz), 124.8, 118.8, 112.1 (t, ^1^*J*_F,C_ = 246.0 Hz), 62.7, 45.6 (2C), 14.4; ^**19**^**F NMR** (235 MHz, CDCl_3_), δ (ppm): −99.1; **FT-IR** (cm^–1^, neat, ATR), 2941, 1760, 1282, 1257, 1233, 1136; **HRMS** (ESI+) *m*/*z*: [M + H]^+^ Calcd for C_12_H_15_BrF_2_NO_2_ 322.0249; found 322.0250.

#### Ethyl 2-(5-Chloro-2-(dimethylamino)phenyl)-2,2-difluoroacetate **(4)**

Yellow oil (42.9 mg, 0.15 mmol, 52% yield from 46.6 mg of 4-chloro-*N*,*N*-dimethylaniline following GP1); Rf = 0.38 (hexane:ethyl acetate, 9.5:0.5). ^**1**^**H NMR** (400 MHz, CDCl_3_), δ (ppm): 7.67 (d, ^4^*J*_H,H_ = 4.0 Hz 1H), 7.44 (dd, ^3^*J*_H,H_ = 9.0 Hz, ^4^*J*_H,H_ = 4.0 Hz, 1H), 7.26 (d, ^3^*J*_H,H_ = 8.0 Hz, 1H), 4.30 (q, ^3^*J*_H,H_ = 8.0 Hz, 2H), 2.54 (s, 6H), 1.30 (t, ^3^*J*_H,H_ = 8.0 Hz, 3H); ^**13**^**C{**^**1**^**H} NMR** (100 MHz, CDCl_3_), δ (ppm): 163.8 (t, ^2^*J*_F,C_ = 32.0 Hz), 151.3, 133.0 (t, ^2^*J*_F,C_ = 24.0 Hz), 132.3, 131.2, 126.9 (t, ^3^*J*_F,C_ = 7.0 Hz), 124.5, 112.1 (t, ^1^*J*_F,C_ = 245.0 Hz), 62.7, 45.7 (2C), 14.4; ^**19**^**F NMR** (282 MHz, CDCl_3_), δ (ppm): −98.7; **FT-IR** (cm^–1^, neat, ATR), 2947, 1770, 1370, 1283, 1256, 1234, 1164; **HRMS** (ESI+) *m*/*z*: [M + Na]^+^ Calcd for C_12_H_14_ClF_2_NO_2_Na 300.0573; found 300.0578.

#### Ethyl 2-(2-(Dimethylamino)-5-iodophenyl)-2,2-difluoroacetate **(5)**

Colorless to yellow oil (49.9 mg, 0.14 mmol, 45% yield from 74.1 mg of *N,N*-dimethyl-4-iodoaniline following GP1); Rf = 0.38 (hexane:ethyl acetate, 9.5:0.5). ^**1**^**H NMR** (300 MHz, CDCl_3_), δ (ppm): 8.00 (d, ^4^*J*_H,H_ = 3.0 Hz, 1H), 7.79 (dd, ^3^*J*_H,H_ = 9.0 Hz, ^4^*J*_H,H_ = 3.0 Hz, 1H), 7.07 (d, ^3^*J*_H,H_ = 9.0 Hz 1H), 4.30 (q, ^3^*J*_H,H_ = 9.0 Hz, 2H), 2.54 (s, 6 H), 1.30 (t, ^3^*J*_H,H_ = 9.0 Hz, 3H); ^**13**^**C{**^**1**^**H} NMR** (75 MHz, CDCl_3_), δ (ppm): 163.9 (t, ^2^*J*_F,C_ = 32.3 Hz), 152.6 (t, ^3^*J*_F,C_ = 5.3 Hz), 141.3 (t, ^4^*J*_F,C_ = 1.5 Hz), 135.7 (t, ^3^*J*_F,C_ = 7.5 Hz), 133.3 (t, ^2^*J*_F,C_ = 23.3 Hz), 125.1, 111.9 (t, ^1^*J*_F,C_ = 246.0 Hz), 89.5, 62.7, 45.6 (2C), 14.5; ^**19**^**F NMR** (282 MHz, CDCl_3_), δ (ppm): −98.6; **FT-IR** (cm^–1^, neat, ATR), 2947, 1769, 1313, 1280, 1260, 1233, 1168; **HRMS** (ESI+) *m*/*z*: [M + H]^+^ Calcd for C_12_H_15_IF_2_NO_2_ 370.0110; found 370.0107.

#### Ethyl 2-(2-(Dimethylamino)-5-fluorophenyl)-2,2-difluoroacetate **(6)**

Colorless oil (22.7 mg, 0.09 mmol, 29% yield from 41.8 mg of *N,N*-dimethyl-4-fluoroaniline following GP1); Rf = 0.38 (hexane:EtOAc, 9.5:0.5). ^**1**^**H NMR** (400 MHz, CDCl_3_), δ (ppm): 7.39 (dd, *J* = 8.0 Hz, *J* = 4.0 Hz, 1H), 7.31 (dd, *J* = 4.0 Hz, *J* = 8.0 Hz, 1H), 7.18 (dd, *J* = 8.0 Hz, *J* = 4.0 Hz, 1H), 4.31 (q, ^3^*J*_H,H_ = 8.0 Hz, 2H), 2.53 (s, 6H), 1.31 (t, ^3^*J*_H,H_ = 8.0 Hz, 3H); ^**13**^**C{**^**1**^**H} NMR** (100 MHz, CDCl_3_), δ (ppm): 163.9 (t, ^2^*J*_F,C_ = 32.0 Hz), 160.2 (d, ^1^*J*_F,C_ = 244.0 Hz), 148.6 (m), 133.4 (td, ^2^*J*_F,C_ = 24.0 Hz, ^3^*J*_F,C_ = 7.0 Hz), 124.7 (t, ^3^*J*_F,C_ = 8.0 Hz), 119.1 (d, ^2^*J*_F,C_ = 22.0 Hz), 113.7 (dt, ^2^*J*_F,C_ = 25.0 Hz, ^3^*J*_F,C_ = 7.0 Hz), 112.1 (t, ^1^*J*_F,C_ = 246.0 Hz), 62.7, 45.9 (2C), 14.5; ^**19**^**F NMR** (282 MHz, CDCl_3_), δ (ppm): −98.6 (s, 2F), −115.3 (m, 1F); **FT-IR** (cm^–1^, neat, ATR), 2945, 1769, 1291, 1268, 1236, 1186; **HRMS** (ESI+) *m*/*z*: [M + Na]^+^ Calcd for C_12_H_14_F_3_NO_2_Na 284.0869; found 284.0863.

#### Ethyl 2-(2-(Dimethylamino)-5-(trifluoromethoxy)phenyl)-2,2-difluoroacetate **(7)**

Colorless to orange oil (44.4 mg, 0.14 mmol, 45% yield from 61.6 mg of *N*,*N*-dimethyl-4-(trifluoromethoxy)aniline following GP1; 48.3 mg, 0.15 mmol, 49% yield from 61.6 mg of *N*,*N*-dimethyl-4-(trifluoromethoxy)aniline following GP2); Rf = 0.43 (hexane:ethyl acetate, 9:1). ^**1**^**H NMR** (400 MHz, CDCl_3_), δ (ppm): 7.55 (d, ^3^*J*_H,H_ = 4.0 Hz, 1H), 7.33 (m, 2H), 4.31 (q, ^3^*J*_H,H_ = 8.0 Hz, 2H), 2.56 (s, 6H), 1.31 (t, ^3^*J*_H,H_ = 8.0 Hz, 3H); ^**13**^**C{**^**1**^**H} NMR** (100 MHz, CDCl_3_), δ (ppm): 163.8 (t, ^2^*J*_F,C_ = 33.0 Hz), 151.4 (t, ^3^*J*_F,C_ = 5.0 Hz), 146.5 (q, ^3^*J*_F,C_ = 2.0 Hz), 133.2 (t, ^2^*J*_F,C_ = 24.0 Hz), 124.9, 124.6, 120.8 (q, ^1^*J*_F,C_ = 259.0 Hz), 119.7 (t, ^3^*J*_F,C_ = 7.0 Hz), 112.0 (t, ^1^*J*_F,C_ = 246.0 Hz), 62.7, 45.8 (2C), 14.4; ^**19**^**F NMR** (376 MHz, CDCl_3_), δ (ppm): −58.1 (s, 3F), −98.7 (s, 2F); **FT-IR** (cm^–1^, neat, ATR), 2949, 1771, 1252, 1214, 1159; **HRMS** (ESI+) *m*/*z*: [M + H]^+^ Calcd for C_13_H_15_F_5_NO_3_ 328.0967; found 328.0964.

#### Ethyl 2-(2-(Dimethylamino)-5-(trifluoromethyl)phenyl)-2,2-difluoroacetate **(8)**

Colorless to yellow oil (33.9 mg, 0.11 mmol, 52% yield from 56.7 mg of *N*,*N*-dimethyl-4-(trifluoromethyl)aniline following GP1); Rf = 0.38 (hexane:ethyl acetate, 9.5:0.5). ^**1**^**H NMR** (400 MHz, CDCl_3_), δ (ppm): 7.98 (d, ^3^*J*_H,H_ = 4.0 Hz, 1H), 7.74 (d, ^3^*J*_H,H_ = 8.0 Hz, 1H), 7.42 (d, ^3^*J*_H,H_ = 8.0 Hz, 1H), 4.31 (q, ^3^*J*_H,H_ = 8.0 Hz, 2H), 2.61 (s, 6H), 1.31 (t, ^3^*J*_H,H_ = 8.0 Hz, 3H); ^**13**^**C{**^**1**^**H} NMR** (75 MHz, CDCl_3_), δ (ppm): 124.0 (q, ^1^*J*_F,C_ = 270.0 Hz), 163.9 (t, ^2^*J*_F,C_ = 32.0 Hz), 156.1 (t, ^3^*J*_F,C_ = 3.8 Hz), 131.9 (t, ^2^*J*_F,C_ = 24.0 Hz), 129.3 (m), 127.8 (q, ^2^*J*_F,C_ = 33.0 Hz), 124.3 (m), 124.0 (q, ^1^*J*_F,C_ = 270.0 Hz), 123.7, 112.1 (t, ^1^*J*_F,C_ = 267.0 Hz), 63.0, 45.6 (2C), 14.4; ^**19**^**F NMR** (282 MHz, CDCl_3_), δ (ppm): −62.4 (s, 3F), −98.3 (s, 2F); **FT-IR** (cm^–1^, neat, ATR), 2840, 1768, 1336, 1261, 1229, 1163; **HRMS** (ESI+) *m*/*z*: [M + H]^+^ Calcd for C_13_H_15_F_5_NO_2_ 312.1017; found 312.1017.

#### Ethyl 2-(2-(Dimethylamino)-5-formylphenyl)-2,2-difluoroacetate **(9)**

Colorless to yellow oil (20.1 mg, 0.07 mmol, 25% yield from 44.8 mg of 4-(dimethylamino)benzaldehyde following GP1; 24.1 mg, 0.09 mmol, 30% yield from 44.8 mg of 4-(dimethylamino)benzaldehyde following GP2); Rf = 0.30 (hexane:ethyl acetate, 8:2). ^**1**^**H NMR** (300 MHz, CDCl_3_), δ (ppm): 10.00 (s, 1H), 8.23 (d, ^3^*J*_H,H_ = 3.0 Hz, 1H), 8.01 (dd, ^3^*J*_H,H_ = 9.0 Hz, ^4^*J*_H,H_ = 3.0 Hz, 1H), 7.43 (d, ^3^*J*_H,H_ = 9.0 Hz, 1H), 4.29 (q, ^3^*J*_H,H_ = 6.0 Hz, 2H), 2.65 (s, 6H), 1.28 (t, ^3^*J*_H,H_ = 6.0 Hz, 3H); ^**13**^**C{**^**1**^**H} NMR** (100 MHz, CDCl_3_), δ (ppm): 190.8, 163.9 (t, ^2^*J*_F,C_ = 33.0 Hz), 158.4 (t, ^3^*J*_F,C_ = 5.0 Hz), 133.3, 132.9, 131.5 (t, ^2^*J*_F,C_ = 24.0 Hz), 129.4 (t, ^3^*J*_F,C_ = 7.0 Hz), 123.2, 112.3 (t, ^1^*J*_F,C_ = 246.0 Hz), 62.8, 45.5 (2C), 14.4; ^**19**^**F NMR** (282 MHz, CDCl_3_), δ (ppm): −97.8; **FT-IR** (cm^–1^, neat, ATR), 2948, 1767, 1696, 1370, 1303, 1269, 1242, 1176; **HRMS** (ESI+) *m*/*z*: [M + Na]^+^ Calcd for C_13_H_15_F_2_NO_3_Na 294.0912; found 294.0909.

#### Ethyl 2-(5-Cyano-2-(dimethylamino)phenyl)-2,2-difluoroacetate **(10)**

Colorless to yellow oil (7.24 mg, 0.03 mmol, 9% yield from 43.9 mg of 4-(dimethylamino)benzonitrile following GP1; traces from 43.9 mg of 4-(dimethylamino)benzonitrile following GP2); Rf = 0.48 (hexane:ethyl acetate, 8:2). ^**1**^**H NMR** (400 MHz, CDCl_3_), δ (ppm): 8.00 (d, ^3^*J*_H,H_ = 4.0 Hz, 1H), 7.75 (dd, ^3^*J*_H,H_ = 8.0 Hz, ^4^*J*_H,H_ = 4.0 Hz, 1H), 7.38 (d, ^3^*J*_H,H_ = 8.0 Hz, 1H), 4.29 (q, ^3^*J*_H,H_ = 8.0 Hz, 2H), 2.62 (s, 6H), 1.29 (t, ^3^*J*_H,H_ = 8.0 Hz, 3H); ^**13**^**C{**^**1**^**H} NMR** (100 MHz, CDCl_3_), δ (ppm): 163.6 (t, ^2^*J*_F,C_ = 33.0 Hz), 157.0 (t, ^3^*J*_F,C_ = 4.0 Hz), 135.9, 132.1 (t, ^2^*J*_F,C_ = 24.0 Hz), 131.3 (t, ^3^*J*_F,C_ = 7.0 Hz), 123.7, 118.4, 111.7 (t, ^1^*J*_F,C_ = 267.0 Hz), 109.1, 63.0, 45.5 (2C), 14.4;^**19**^**F NMR** (282 MHz, CDCl_3_), δ (ppm): −97.3; **HRMS** (ESI+) *m*/*z*: [M + Na]^+^ Calcd for C_13_H_14_F_2_N_2_O_2_Na 291.0916; found 291.0917.

#### Ethyl 2-(4-(Dimethylamino)-[1,1′-biphenyl]-3-yl)-2,2-difluoroacetate **(11)**

Yellow to colorless oil (61.4 mg, 0.19 mmol, 64% yield from 59.2 mg of *N*,*N*-dimethyl-[1,1′-biphenyl]-4-amine following GP1; 65.2 mg, 0.20 mmol, 68% yield from 59.2 mg of *N*,*N*-dimethyl-[1,1′-biphenyl]-4-amine following GP2); Rf = 0.28 (hexane:ethyl acetate, 9.5:0.5). ^**1**^**H NMR** (300 MHz, CDCl_3_), δ (ppm): 7.93 (d, ^3^*J*_H,H_ = 9.0 Hz, 1H), 7.71 (dd, ^3^*J*_H,H_ = 9.0 Hz, ^4^*J*_H,H_ = 3.0 Hz, 1H), 7.58–7.62 (m, 2H), 7.36–7.48 (m, 4H), 4.33 (q, ^3^*J*_H,H_ = 6.0 Hz, 2 H), 2.62 (s, 6 H), 1.34 (t, ^3^*J*_H,H_ = 6.0 Hz, 3H); ^**13**^**C{**^**1**^**H} NMR** (75 MHz, CDCl_3_), δ (ppm): 164.4 (t, ^2^*J*_F,C_ = 33.0 Hz), 151.8 (t, ^3^*J*_F,C_ = 4.5 Hz), 140.4, 138.9, 131.7 (t, ^2^*J*_F,C_ = 23.3 Hz), 130.8, 129.2 (2C), 127.9, 127.4 (2C), 125.3 (t, ^3^*J*_F,C_ = 6.8 Hz), 123.3, 112.9 (t, ^1^*J*_F,C_ = 244.5 Hz), 63.1, 45.8 (2C), 14.5; ^**19**^**F NMR** (235 MHz, CDCl_3_), δ (ppm): −98.2; **FT-IR** (cm^–1^, neat, ATR), 2940, 1766, 1299, 1222, 1137; **HRMS** (ESI+) *m*/*z*: [M + H]^+^ Calcd for C_18_H_20_F_2_NO_2_ 320.1456; found 320.1444.

#### Ethyl 2-(5-(*tert*-Butyl)-2-(dimethylamino)phenyl)-2,2-difluoroacetate **(12)**

Colorless to yellow oil (61.1 mg, 0.21 mmol, 68% yield from 53.2 mg of 4-(*tert*-butyl)-*N*,*N*-dimethylaniline following GP1; 80.0 mg, 0.27 mmol, 89% yield from 53.2 mg of 4-(*tert*-butyl)-*N*,*N*-dimethylaniline following GP2); Rf = 0.40 (hexane:ethyl acetate, 9.5:0.5). ^**1**^**H NMR** (400 MHz, CDCl_3_), δ (ppm): 7.70 (d, ^4^*J*_H,H_ = 4.0 Hz, 1H), 7.50 (dd, ^3^*J*_H,H_ = 12.0 Hz, ^4^*J*_H,H_ = 4.0 Hz, 1H), 7.25 (d, ^3^*J*_H,H_ = 12.0 Hz, 1H), 4.31 (q, ^3^*J*_H,H_ = 8.0 Hz, 2H), 2.54 (s, 6H), 1.34 (s, 9H), 1.33 (m, 3H); ^**13**^**C{**^**1**^**H} NMR** (100 MHz, CDCl_3_), δ (ppm): 164.6 (t, ^2^*J*_F,C_ = 32.0 Hz), 149.9 (t, ^3^*J*_F,C_ = 5.0 Hz), 148.8, 130.6 (t, ^2^*J*_F,C_ = 23.0 Hz), 129.3, 123.1 (t, ^3^*J*_F,C_ = 6.0 Hz), 122.3, 113.2 (t, ^1^*J*_F,C_ = 244.0 Hz), 109.3, 62.4, 45.7 (2C), 35.0, 31.7 (3C), 14.5; ^**19**^**F NMR** (376 MHz, CDCl_3_), δ (ppm): −97.8; **FT-IR** (cm^–1^, neat, ATR), 2955, 1768, 1366, 1231; **HRMS** (ESI+) *m*/*z*: [M + H]^+^ Calcd for C_16_H_24_F_2_NO_2_ 300.1769; found 300.1764; **Elem. Anal.** Calcd for C_16_H_23_F_2_NO_2_: C, 64.20; H, 7.74; N, 4.68. Found: C, 64.66; H, 8.09; N, 4.59.

#### Ethyl 2-(2-(Dimethylamino)-5-(methylthio)phenyl)-2,2-difluoroacetate **(13)**

Brown to orange oil (59.2 mg, 0.21 mmol, 68% yield from 50.2 mg of *N*,*N*-dimethyl-4-(methylthio)aniline following GP1; 59.2 mg, 0.21 mmol, 68% yield from 50.2 mg of *N*,*N*-dimethyl-4-(methylthio)aniline following GP2); Rf = 0.33 (hexane:ethyl acetate, 9.5:0.5). ^**1**^**H NMR** (300 MHz, CDCl_3_), δ (ppm): 7.57 (d, ^4^*J*_H,H_ = 3.0 Hz, 1H), 7.37 (dd, ^3^*J*_H,H_ = 9.0 Hz, ^4^*J*_H,H_ = 3.0 Hz, 1H), 7.25 (d, ^3^*J*_H,H_ = 9.0 Hz, 1H), 4.30 (q, ^3^*J*_H,H_ = 6.0 Hz, 2H), 2.54 (s, 6H), 2.50 (s, 3H), 1.31 (t, ^3^*J*_H,H_ = 6.0 Hz, 3H); ^**13**^**C{**^**1**^**H} NMR** (75 MHz, CDCl_3_), δ (ppm): 164.4 (t, ^2^*J*_F,C_ = 33.0 Hz), 151.8 (t, ^3^*J*_F,C_ = 4.5 Hz), 140.4, 138.9, 131.7 (t, ^2^*J*_F,C_ = 23.3 Hz), 130.8, 129.2 (2C), 127.9, 127.4 (2C), 125.3 (t, ^3^*J*_F,C_ = 6.8 Hz), 123.3, 112.9 (t, ^1^*J*_F,C_ = 244.5 Hz), 63.1, 45.8 (2C), 16.5, 14.5; ^**19**^**F NMR** (235 MHz, CDCl_3_), δ (ppm): −98.6; **FT-IR** (cm^–1^, neat, ATR), 2944, 1769, 1371, 1313, 1286, 1264, 1236, 1189; **HRMS** (ESI+) *m*/*z*: [M + H]^+^ Calcd for C_13_H_18_F_2_NO_2_S 290.1020; found 290.1011.

#### Ethyl 2-(2-(Dimethylamino)-5-methoxyphenyl)-2,2-difluoroacetate **(14)**

Yellow oil (38.3 mg, 0.14 mmol, 47% yield from 45.4 mg of 4-methoxy-*N*,*N*-dimethylaniline following GP1; 54.5 mg, 0.22 mmol, 73% yield from 45.4 mg of 4-methoxy-*N*,*N*-dimethylaniline following GP2); Rf = 0.58 (hexane:ethyl acetate, 9:1). ^**1**^**H NMR** (300 MHz, CDCl_3_), δ (ppm): 7.26 (d, ^3^*J*_H,H_ = 9.0 Hz, 1H), 7.20 (d, ^4^*J*_H,H_ = 3.0 Hz, 1H), 7.01 (dd, ^3^*J*_H,H_ = 9.0 Hz, ^4^*J*_H,H_ = 3.0 Hz, 1H), 4.31 (q, ^3^*J*_H,H_ = 9.0 Hz, 2H), 3.81 (s, 3H), 2.51 (s, 6H), 1.31 (t, ^3^*J*_H,H_ = 9.0 Hz, 3H); ^**13**^**C{**^**1**^**H} NMR** (100 MHz, CDCl_3_), δ (ppm): 164.2 (t, ^2^*J*_F,C_ = 33.0 Hz), 157.4, 145.4 (t, ^3^*J*_F,C_ = 6.0 Hz), 132.4 (t, ^2^*J*_F,C_ = 24.0 Hz), 124.1, 118.3, 112.7 (t, ^1^*J*_F,C_ = 245.0 Hz), 110.9 (t, ^3^*J*_F,C_ = 7.0 Hz), 62.4, 55.9, 45.8 (2 C), 14.5; ^**19**^**F NMR** (282 MHz, CDCl_3_), δ (ppm): −98.7; **FT-IR** (cm^–1^, neat, ATR), 2941, 1767, 1279, 1215, 1178; **HRMS** (ESI+) *m*/*z*: [M + H]^+^ Calcd for C_13_H_18_F_2_NO_3_ 274.1249; found 274.1251.

#### Ethyl 2-(2,5-bis(Dimethylamino)phenyl)-2,2-difluoroacetate **(15)**

Colorless oil (35.4 mg, 0.12 mmol, 41% yield from 49.3 mg of *N*^1^,*N*^1^,*N*^4^,*N*^4^-tetramethylbenzene-1,4-diamine following GP1); Rf = 0.20 (hexane:ethyl acetate, 9.5:0.5). ^**1**^**H NMR** (400 MHz, CDCl_3_), δ (ppm): 7.21 (d, ^3^*J*_H,H_ = 8.0 Hz, 1H), 7.00 (d, ^4^*J*_H,H_ = 4.0 Hz, 1H), 6.82 (dd, ^3^*J*_H,H_ = 8.0 Hz, ^4^*J*_H,H_ = 4.0 Hz, 1H), 4.31 (q, ^3^*J*_H,H_ = 8.0 Hz, 2H), 2.96 (s, 6H), 2.51 (s, 6H), 1.32 (t, ^3^*J*_H,H_ = 8.0 Hz, 3H); ^**13**^**C{**^**1**^**H} NMR** (100 MHz, CDCl_3_), δ (ppm): 164.4 (t, ^2^*J*_F,C_ = 33.0 Hz), 148.7, 141.5 (t, ^3^*J*_F,C_ = 5.0 Hz), 131.8 (t, ^2^*J*_F,C_ = 22.0 Hz), 123.5, 116.1, 113.2 (t, ^1^*J*_F,C_ = 245.0 Hz), 109.4 (t, ^3^*J*_F,C_ = 7.0 Hz), 62.3, 45.9 (2C), 41.1 (2C), 14.5; ^**19**^**F NMR** (376 MHz, CDCl_3_), δ (ppm): −98.2; **FT-IR** (cm^–1^, neat, ATR), 2939, 1765, 1309, 1265, 1223, 1177; **HRMS** (ESI+) *m*/*z*: [M + H]^+^ Calcd for C_14_H_21_F_2_N_2_O_2_ 287.1566; found 287.1573.

#### Ethyl 2-(2-(Dimethylamino)-5-morpholinophenyl)-2,2-difluoroacetate **(16)**

Yellow oil (33.0 mg, 0.10 mmol, 34% yield from 62.0 mg of *N*,*N*-dimethyl-4-morpholinoaniline following GP1); Rf = 0.45 (hexane:ethyl acetate, 8:2). ^**1**^**H NMR** (300 MHz, CDCl_3_), δ (ppm): 7.21 (m, 2H), 7.01 (m, 1H), 4.31 (q, ^3^*J*_H,H_ = 6.0 Hz, 2H), 3.85 (m, 4H), 3.17 (m, 4H), 2.51 (s, 6H), 1.31 (t, ^3^*J*_H,H_ = 6.0 Hz, 3H); ^**13**^**C{**^**1**^**H} NMR** (75 MHz, CDCl_3_), δ (ppm): 164.3 (t, ^2^*J*_F,C_ = 33.0 Hz), 149.3, 144.7 (t, ^3^*J*_F,C_ = 5.3 Hz), 132.0 (t, ^2^*J*_F,C_ = 22.5 Hz), 119.3 (t, ^4^*J*_F,C_ = 1.5 Hz), 113.1 (t, ^3^*J*_F,C_ = 7.5 Hz), 113.0 (t, ^1^*J*_F,C_ = 245.3 Hz), 109.4 (t, ^3^*J*_F,C_ = 7.5 Hz), 67.2 (2C), 62.4, 49.7 (2C), 45.8 (2C), 14.5; ^**19**^**F NMR** (235 MHz, CDCl_3_), δ (ppm): −98.6; **FT-IR** (cm^–1^, neat, ATR), 2920, 1743, 1318, 1303, 1274, 1244, 1117; **HRMS** (ESI+) *m*/*z*: [M + H]^+^ Calcd for C_16_H_23_F_2_N_2_O_3_ 329.1671; found 329.1695.

#### Ethyl 2-(5-((*tert*-Butoxycarbonyl)amino)-2-(dimethylamino)phenyl)-2,2-difluoroacetate **(17)**

Yellowish oil (56.5 mg, 0.16 mmol, 53% yield from 70.9 mg of *tert*-butyl (4-(dimethylamino)phenyl)carbamate following GP1); Rf = 0.65 (hexane:ethyl acetate, 8:2). ^**1**^**H NMR** (400 MHz, CDCl_3_), δ (ppm): 7.59 (m, 2H), 7.25 (d, ^3^*J*_H,H_ = 8.0 Hz, 1H), 6.59 (s, 1H), 4.29 (q, ^3^*J*_H,H_ = 8.0 Hz, 2H), 2.52 (s, 6H), 1.51 (s, 9H), 1.30 (t, ^3^*J*_H,H_ = 8.0 Hz, 3H); ^**13**^**C{**^**1**^**H} NMR** (100 MHz, CDCl_3_), δ (ppm): 164.2 (t, ^2^*J*_F,C_ = 32.0 Hz), 153.1, 147.6 (t, ^3^*J*_F,C_ = 5.0 Hz), 136.2, 132.0 (t, ^2^*J*_F,C_ = 23 Hz), 123.6, 122.5, 116.7 (t, ^3^*J*_F,C_ = 7.0 Hz), 112.6 (t, ^1^*J*_F,C_ = 245.0 Hz), 81.2, 62.5, 45.8 (2C), 28.6 (3C), 14.5; ^**19**^**F NMR** (376 MHz, CDCl_3_), δ (ppm): −98.7; **FT-IR** (cm^–1^, neat, ATR), 3356, 2980, 1762, 1725, 1522, 1455, 1425, 1393, 1368, 1298, 1234, 1152; **HRMS** (ESI+) *m*/*z*: [M + Na]^+^ Calcd for C_17_H_24_F_2_N_2_O_4_Na 381.1596; found 381.1595.

#### Ethyl 2-(4-Amino-3,5-difluorophenyl)-2,2-difluoroacetate **(18)**

Brownish oil (12.8 mg, 0.05 mmol, 17% yield from 38.7 mg of 2,6-difluoroaniline following GP1; 16.9 mg, 0.07 mmol, 23% yield from 38.7 mg of 2,6-difluoroaniline following GP2); Rf = 0.35 (hexane:ethyl acetate, 8.5:1.5). ^**1**^**H NMR** (400 MHz, CDCl_3_), δ (ppm): 7.10 (d, ^3^*J*_H,H_ = 8.0 Hz, 2 H), 4.30 (q, ^3^*J*_H,H_ = 6.0 Hz, 2H), 3.98 (bs, 2H), 1.32 (t, ^3^*J*_H,H_ = 6.0 Hz, 3H); ^**13**^**C{**^**1**^**H} NMR** (75 MHz, CDCl_3_), δ (ppm): 163.8 (t, ^2^*J*_F,C_ = 61.6 Hz), 151.5 (dd, 2C, ^1^*J*_F,C_ = 240.0 Hz, ^3^*J*_F,C_ = 8.3 Hz), 127.1 (t, ^2^*J*_F,C_ = 29.92 Hz), 121.2 (t, ^3^*J*_F,C_ = 8.3 Hz), 112.8 (tt, ^1^*J*_F,C_ = 251.0 Hz, ^4^*J*_F,C_ = 2.3 Hz), 109.2 (m, 2C), 63.6, 14.2; ^**19**^**F NMR** (282 MHz, CDCl_3_), δ (ppm): −102.6 (s, 2F), −131.1 (d, ^4^*J*_F,C_ = 3.5 Hz, 2F); **HRMS** (ESI+) *m*/*z*: [M + Na]^+^ Calcd for C_10_H_9_F_4_NO_2_Na 274.0462; found 274.0445. **Elem. Anal.** Calcd for C_10_H_9_F_4_NO_2_: C, 47.82; H, 3.61; N, 5.58. Found: C, 47.72; H, 3.64; N, 5.35.

#### Ethyl 2-(4-Amino-3,5-dibromophenyl)-2,2-difluoroacetate **(19)**

Yellow to orange powder (10.5 mg, 0.03 mmol, 9% yield from 75.3 mg of 2,6-dibromoaniline following GP1; traces from 75.3 mg of 2,6-dibromoaniline following GP2); Rf = 0.35 (hexane:ethyl acetate, 9.5:0.5); **mp**: 57–59 °C. ^**1**^**H NMR** (300 MHz, CDCl_3_), δ (ppm): 7.62 (s, 2H), 4.84 (bs, 2H), 4.31 (q, ^3^*J*_H,H_ = 6.0 Hz, 2H), 1.33 (t, ^3^*J*_H,H_ = 6.0 Hz, 3H); ^**13**^**C{**^**1**^**H} NMR** (100 MHz, CDCl_3_), δ (ppm): 164.2 (t, ^2^*J*_F,C_ = 35 Hz), 144.6, 129.5 (t, ^3^*J*_F,C_ = 7.0 Hz), 123.7 (t, ^2^*J*_F,C_ = 27.9 Hz), 112.4 (t, ^1^*J*_F,C_ = 252.0 Hz), 108.3, 63.7, 14.3; ^**19**^**F NMR** (282 MHz, CDCl_3_), δ (ppm): −102.6; **FT-IR** (cm^–1^, neat, ATR), 2924, 1770, 1244, 1098; **HRMS** (ESI+) *m*/*z*: [M + Na]^+^ Calcd for C_10_H_9_Br_2_F_2_NO_2_ 393.8860; found 393.8856.

#### Ethyl 2-(2′-Amino-[1,1′:3′,1″-terphenyl]-5′-yl)-2,2-difluoroacetate **(20)**

Red oil (40.6 mg, 0.11 mmol, 37% yield from 73.6 mg of [1,1′:3′,1″-terphenyl]-2′-amine following GP1); Rf = 0.15 (hexane:ethyl acetate, 9.5:0.5). ^**1**^**H NMR** (300 MHz, CDCl_3_), δ (ppm) 7.38–7.50 (m, 10H), 7.37 (s, 2H), 4.34 (q, ^3^*J*_H,H_ = 6.0 Hz, 2H), 4.11 (s, 2H), 1.34 (t, ^3^*J*_H,H_ = 6.0 Hz, 3H); ^**13**^**C{**^**1**^**H} NMR** (75 MHz, CDCl_3_), δ (ppm): 165.0 (t, ^2^*J*_F,C_ = 36.8 Hz), 143.7 (t, *J*_F,C_ = 2 Hz), 139.0, 129.5 (4C), 129.4 (4C), 128.1 (2C), 127.8 (2C), 127.2 (t, ^3^*J*_F,C_ = 6.0 Hz, 2C), 122.2 (t, ^2^*J*_F,C_ = 26.3 Hz), 114.1 (t, ^1^*J*_F,C_ = 249.8 Hz), 63.2, 14.3; ^**19**^**F NMR** (235 MHz, CDCl_3_), δ (ppm) −101.7; **FT-IR** (cm^–1^, neat, ATR), 3488, 3383, 2926, 1760, 1370, 1342, 1295, 1218; **HRMS** (ESI+) *m*/*z*: [M + Na]^+^ Calcd for C_22_H_19_F_2_NO_2_Na 390.1276; found 390.1257.

#### Ethyl 2-(4-Amino-3,5-dimethylphenyl)-2,2-difluoroacetate **(21)**

Brownish oil (42.3 mg, 0.17 mmol, 58% yield from 36.4 mg of 2,6-dimethylaniline following GP1; 58.3 mg, 0.24 mmol, 80% yield from 36.4 mg of 2,6-dimethylaniline following GP2); Rf = 0.43 (hexane:ethyl acetate, 8:2). ^**1**^**H NMR** (400 MHz, CDCl_3_), δ (ppm): 7.17 (s, 2H), 4.29 (q, ^3^*J*_H,H_ = 4.0 Hz, 2H), 3.80 (s, 2H), 2.19 (s, 6H), 1.30 (t, ^3^*J*_H,H_ = 4.0 Hz, 3H); ^**13**^**C{**^**1**^**H} NMR** (100 MHz, CDCl_3_), δ (ppm): 165.3 (t, ^2^*J*_F,C_ = 36.0 Hz), 145.6, 125.7 (t, 2C, ^3^*J*_F,C_ = 6.0 Hz), 121.8 (t, 2C, ^2^*J*_F,C_ = 26.0 Hz), 121.6 (2C), 114.3 (t, ^1^*J*_F,C_ = 250.0 Hz), 63.1, 17.9 (2C), 14.3; ^**19**^**F NMR** (282 MHz, CDCl_3_), δ (ppm): −101.9; **HRMS** (EI) *m*/*z*: [M + H]^+^ Calcd for C_12_H_16_F_2_NO_2_ 244.1143; found: 244.1138.

#### Ethyl 2-(4-(Dimethylamino)naphthalen-1-yl)-2,2-difluoroacetate **(22)**

Yellow oil (53.5 mg, 0.18 mmol, 61% yield from 51.4 mg of *N*,*N*-dimethylnaphthalen-1-amine following GP1; 53.5 mg, 0.18 mmol, 61% yield from 51.4 mg of *N*,*N*-dimethylnaphthalen-1-amine following GP2); Rf = 0.48 (hexane:ethyl acetate, 9.5:0.5). ^**1**^**H NMR** (400 MHz, CDCl_3_), δ (ppm): 8.29 (dd, ^3^*J*_H,H_ = 8.0 Hz, ^4^*J*_H,H_ = 4.0 Hz, 1H), 8.18 (d, ^4^*J*_H,H_ = 4.0 Hz, 1H), 7.75 (d, ^3^*J*_H,H_ = 8.0 Hz, 1H), 7.54 (m, 2H), 7.05 (d, ^3^*J*_H,H_ = 8.0 Hz, 1H), 4.29 (q, ^3^*J*_H,H_ = 8.0 Hz, 2H), 2.93 (s, 6H), 1.26 (t, ^3^*J*_H,H_ = 8.0 Hz, 3H); ^**13**^**C{**^**1**^**H} NMR** (100 MHz, CDCl_3_), δ (ppm): 165.1 (t, ^2^*J*_F,C_ = 35.0 Hz), 154.4, 131.2 (t, ^3^*J*_F,C_ = 2.0 Hz), 129.2, 127.3, 125.8, 125.7, 125.6, 125.5, 125.0 (t, ^3^*J*_F,C_ = 3.0 Hz), 122.7 (t, ^2^*J*_F,C_ = 23.0 Hz), 115.1 (t, ^1^*J*_F,C_ = 249.0 Hz), 112.6, 63.4, 45.3 (2C), 14.2; ^**19**^**F NMR** (376 MHz, CDCl_3_), δ (ppm): −99.1; **FT-IR** (cm^–1^, neat, ATR), 2943, 1761, 1335, 1264, 1248, 1191; **HRMS** (ESI+) *m*/*z*: [M + Na]^+^ Calcd for C_16_H_17_F_2_NO_2_Na 316.1120; found 316.1129.

#### Ethyl 2-(3-Chloro-2-(dimethylamino)phenyl)-2,2-difluoroacetate **(23a)**

Colorless oil (4.0 mg, 0.01 mmol, 5% yield from 47.0 mg of 2-chloro-*N*,*N*-dimethylaniline following GP2); Rf = 0.28 (hexane:ethyl acetate, 9.7:0.3). ^**1**^**H NMR** (300 MHz, CD_2_Cl_2_), δ (ppm): 7.37 (bs, 2 H), 7.26 (dd, ^3^*J*_H,H_ = 6.0 Hz, ^4^*J*_H,H_ = 3.0 Hz, 1 H), 4.31 (q, ^3^*J*_H,H_ = 6.0 Hz, 2 H), 2.79 (s, 6 H), 1.29 (t, ^3^*J*_H,H_ = 6.0 Hz, 3 H); ^**19**^**F NMR** (282 MHz, CD_2_Cl_2_), δ (ppm): −101.8; **HRMS** (ESI+) *m*/*z*: [M + H]^+^ Calcd for C_12_H_15_ClF_2_NO_2_ 278.0754; found 278.0735.

#### Ethyl 2-(4-Chloro-3-(dimethylamino)phenyl)-2,2-difluoroacetate **(23b)**

Colorless oil (12.5 mg, 0.05 mmol, 15% yield from 47.0 mg of 2-chloro-*N*,*N*-dimethylaniline following GP2); Rf = 0.43 (hexane:ethyl acetate, 9.7:0.3). ^**1**^**H NMR** (300 MHz, CD_2_Cl_2_), δ (ppm): 7.42 (d, ^3^*J*_H,H_ = 9.0 Hz, 1H), 7.25 (d, ^3^*J*_H,H_ = 3.0 Hz, 1 H), 7.14 (dd, ^3^*J*_H,H_ = 9.0 Hz, ^4^*J*_H,H_ = 3.0 Hz, 1 H), 4.29 (q, ^3^*J*_H,H_ = 6.0 Hz, 2 H), 2.82 (s, 6 H), 1.29 (t, ^3^*J*_H,H_ = 6.0 Hz, 3 H); ^**13**^**C{**^**1**^**H} NMR** (125 MHz, CD_2_Cl_2_), δ (ppm): 164.2 (t, ^2^*J*_F,C_ = 35.0 Hz), 151.3, 132.4 (t, ^2^*J*_F,C_ = 25.0 Hz), 131.4, 131.2, 120.0 (t, ^3^*J*_F,C_ = 6.3 Hz), 117.3 (t, ^3^*J*_F,C_ = 6.3 Hz), 113.6 (t, ^1^*J*_F,C_ = 250.0 Hz), 63.8, 43.6 (2 C), 14.1; ^**19**^**F NMR** (282 MHz, CD_2_Cl_2_), δ (ppm): −103.8; **FT-IR** (cm^–1^, neat, ATR), 2947, 1769, 1313, 1280, 1260, 1233, 1168; **HRMS** (ESI+) *m*/*z*: [M + H]^+^ Calcd for C_12_H_15_ClF_2_NO_2_ 278.0754; found 278.0735.

#### Ethyl 2-(3-Chloro-4-(dimethylamino)phenyl)-2,2-difluoroacetate **(23c)**

Colorless oil (22.5 mg, 0.08 mmol, 27% yield from 47.0 mg of 2-chloro-*N*,*N*-dimethylaniline following GP2); Rf = 0.35 (hexane:ethyl acetate, 9.7:0.3). ^**1**^**H NMR** (300 MHz, CDCl_3_), δ (ppm): 7.58 (d, ^3^*J*_H,H_ = 3.0 Hz, 1H), 7.42 (dd, ^3^*J*_H,H_ = 9.0 Hz, ^4^*J*_H,H_ = 3.0 Hz, 1 H), 7.07 (d, ^3^*J*_H,H_ = 9.0 Hz, 1 H), 4.30 (q, ^3^*J*_H,H_ = 6.0 Hz, 2 H), 2.86 (s, 6 H), 1.32 (t, ^3^*J*_H,H_ = 6.0 Hz, 3 H); ^**13**^**C{**^**1**^**H} NMR** (125 MHz, CDCl_3_), δ (ppm): 164.2 (t, ^2^*J*_F,C_ = 35.0 Hz), 152.8, 128.3 (t, ^3^*J*_F,C_ = 6.3 Hz), 127.7, 126.8 (t, ^2^*J*_F,C_ = 26.3 Hz), 124.9 (t, ^3^*J*_F,C_ = 6.3 Hz), 119.7, 113.0 (t, ^1^*J*_F,C_ = 250.0 Hz), 63.3, 43.5 (2 C), 14.1; ^**19**^**F NMR** (282 MHz, CDCl_3_), δ (ppm): −103.0; **FT-IR** (cm^–1^, neat, ATR), 2947, 1769, 1313, 1280, 1260, 1233, 1168; **HRMS** (ESI+) *m*/*z*: [M + H]^+^ Calcd for C_12_H_15_ClF_2_NO_2_ 278.0754; found 278.0735.

#### Ethyl 2-(2-(Dimethylamino)phenyl)-2,2-difluoroacetate **(24a)**(23)

Yellowish oil (11.2 mg, 0.05 mmol, 15% yield from 36.0 mg of *N*,*N*-dimethylaniline following GP2); Rf = 0.40 (hexane:ethyl acetate, 9.5:0.5). ^**1**^**H NMR** (300 MHz, CDCl_3_), δ (ppm): 7.71 (dd, ^3^*J*_H,H_ = 6.0 Hz, ^4^*J*_H,H_ = 3.0 Hz, 1H), 7.49 (t, ^3^*J*_H,H_ = 6.0 Hz, 1 H), 7.30 (m, 2 H), 4.30 (q, ^3^*J*_H,H_ = 6.0 Hz, 2 H), 2.57 (s, 6 H), 1.31 (t, ^3^*J*_H,H_ = 6.0 Hz, 3 H); ^**13**^**C{**^**1**^**H} NMR** (125 MHz, CDCl_3_), δ (ppm): 164.2 (t, ^2^*J*_F,C_ = 33.8 Hz), 152.5, 132.0, 131.3 (t, ^2^*J*_F,C_ = 23.8 Hz), 126.4 (t, ^3^*J*_F,C_ = 6.3 Hz), 125.6, 122.7, 112.7 (t, ^1^*J*_F,C_ = 245.0 Hz), 62.3, 45.6 (2 C), 14.3; ^**19**^**F NMR** (282 MHz, CDCl_3_), δ (ppm) −98.3; **FT-IR** (cm^–1^, neat, ATR), 2924, 1768, 1456, 1305, 1270, 1242, 1127. **HRMS** (ESI+) *m*/*z*: [M + H]^+^ Calcd for C_12_H_16_F_2_NO_2_ 244.1144; found 244.1152.

#### Ethyl 2-(4-(Dimethylamino)phenyl)-2,2-difluoroacetate **(24b)**

Brownish oil (14.0 mg, 0.06 mmol, 20% yield from 36.0 mg of *N*,*N*-dimethylaniline following GP2); Rf = 0.45 (hexane:ethyl acetate, 9.5:0.5). ^**1**^**H NMR** (500 MHz, CDCl_3_), δ (ppm): 7.45 (d, ^3^*J*_H,H_ = 10.0 Hz, 2H), 6.70 (d, ^3^*J*_H,H_ = 10.0 Hz, 2 H), 4.29 (q, ^3^*J*_H,H_ = 10.0 Hz, 2 H), 3.00 (s, 6 H), 1.31 (t, ^3^*J*_H,H_ = 10.0 Hz, 3 H); ^**13**^**C{**^**1**^**H} NMR** (125 MHz, CDCl_3_), δ (ppm): 165.0 (t, ^2^*J*_F,C_ = 36.3 Hz), 152.1 (2 C), 126.7 (t, ^3^*J*_F,C_ = 5.0 Hz), 119.7 (t, ^2^*J*_F,C_ = 26.3 Hz), 114.3 (t, ^1^*J*_F,C_ = 250.0 Hz), 111.6 (2 C), 62.9, 40.3 (2 C), 14.1; ^**19**^**F NMR** (282 MHz, CDCl_3_), δ (ppm) −101.9; **FT-IR** (cm^–1^, neat, ATR), 2923, 1762, 1614, 1593, 1530, 1368, 1275, 1195; **HRMS** (ESI+) *m*/*z*: [M + H]^+^ Calcd for C_12_H_16_F_2_NO_2_ 244.1144; found 244.1152.

#### Ethyl 2-(4-(Diethylamino)phenyl)-2,2-difluoroacetate **(25a)**

Green oil (11.3 mg, 0.04 mmol, 14% yield from 44.8 mg of *N,N*-diethylaniline following GP2); Rf = 0.33 (hexane:ethyl acetate, 9.5:0.5). ^**1**^**H NMR** (300 MHz, CDCl_3_), δ (ppm): 7.42 (d, 2 H, ^3^*J*_H,H_ = 6.0 Hz), 6.65 (d, 2 H, ^3^*J*_H,H_ = 6.0 Hz), 4.29 (q, 2 H, ^3^*J*_H,H_ = 6.0 Hz), 3.37 (q, 4 H, ^3^*J*_H,H_ = 6.0 Hz), 1.31 (t, 3 H, ^3^*J*_H,H_ = 6.0 Hz), 1.17 (t, 6 H, ^3^*J*_H,H_ = 6.0 Hz); ^**13**^**C{**^**1**^**H} NMR** (100 MHz, CDCl_3_), δ (ppm): 164.9 (t, ^2^*J*_F,C_ = 37.0 Hz), 149.2, 126.7 (t, 2 C, ^3^*J*_F,C_ = 6.0 Hz), 114.1 (t, ^1^*J*_F,C_ = 249.0 Hz), 110.9 (2 C), 110.5, 62.7, 44.5 (2 C), 13.9, 12.4 (2 C); ^**19**^**F NMR** (282 MHz, CDCl_3_), δ (ppm): −101.5 (CF_2_); **HRMS** (ESI+) *m*/*z*: [M + H]^+^ Calcd for C_14_H_20_F_2_NO_2_ 272.1456; found 272.1451. See also compound **29** in the Supporting Information.

#### Ethyl 2-(2-(Diethylamino)phenyl)-2,2-difluoroacetate **(25b)**

(traces from 44.8 mg of *N,N*-diethylaniline following GP2); ^**1**^**H NMR** (400 MHz, CDCl_3_), δ (ppm): 7.74 (d, 1 H, ^3^*J*_H,H_ = 8.0 Hz), 7.46 (t, 1 H, ^3^*J*_H,H_ = 8.0 Hz), 7.26 (t, 2 H, ^3^*J*_H,H_ = 8.0 Hz), 4.32 (q, 2 H, ^3^*J*_H,H_ = 8.0 Hz), 2.93 (q, 4 H, ^3^*J*_H,H_ = 8.0 Hz), 1.31 (t, 3 H, ^3^*J*_H,H_ = 8.0 Hz), 0.98 (t, 6 H, ^3^*J*_H,H_ = 8.0 Hz); ^**19**^**F NMR** (282 MHz, CDCl_3_), δ (ppm): −97.2 (CF_2_). **HRMS** (ESI+) *m*/*z*: [M + H]^+^ Calcd for C_14_H_20_F_2_NO_2_ 272.1456; found 272.1451.

#### Diethyl 2,2′-(4-(Diethylamino)-1,3-phenylene)bis(2,2-difluoroacetate) **(25c)**

(traces from 44.8 mg of *N,N*-diethylaniline following GP2); ^**1**^**H NMR** (400 MHz, CDCl_3_), δ (ppm): 7.99 (s, 1 H), 7.71 (d, 1 H,^3^*J*_H,H_ = 12.0 Hz), 7.33 (d, 1 H, ^3^*J*_H,H_ = 12.0 Hz), 4.32 (q, 4 H, ^3^*J*_H,H_ = 12.0 Hz), 2.96 (q, 4 H, ^3^*J*_H,H_ = 12.0 Hz), 1.34 (t, 6 H, ^3^*J*_H,H_ = 12.0 Hz), 0.98 (t, 6 H, ^3^*J*_H,H_ = 12.0 Hz); ^**19**^**F NMR** (282 MHz, CDCl_3_), δ (ppm): −97.8 (CF_2_), −103.5 (CF_2_). **HRMS** (ESI+) *m*/*z*: [M + Na]^+^ Calcd for C_18_H_23_F_4_NO_4_Na 416.1455; found 416.1447.

#### Ethyl 2-(4-Chloro-2-(dimethylamino)phenyl)-2,2-difluoroacetate **(26)**

Colorless oil (16.6 mg, 0.06 mmol, 20% yield from 47.0 mg of 3-chloro-*N*,*N*-dimethylaniline following GP2); Rf = 0.35 (hexane:ethyl acetate, 9.5:0.5). ^**1**^**H NMR** (400 MHz, CDCl_3_), δ (ppm): 7.64 (d, ^3^*J*_H,H_ = 8.0 Hz, 1H), 7.28 (s, 1 H), 7.27 (dd, ^3^*J*_H,H_ = 8.0 Hz, ^4^*J*_H,H_ = 4.0 Hz, 1 H), 4.29 (q, ^3^*J*_H,H_ = 8.0 Hz, 2 H), 2.56 (s, 6 H), 1.30 (t, ^3^*J*_H,H_ = 8.0 Hz, 3 H); ^**13**^**C{**^**1**^**H} NMR** (125 MHz, CDCl_3_), δ (ppm): 163.9 (t, ^2^*J*_F,C_ = 33.8 Hz), 153.8 (t, ^3^*J*_F,C_ = 5.0 Hz), 137.8, 129.7 (t, ^2^*J*_F,C_ = 23.8 Hz), 127.8 (t, ^3^*J*_F,C_ = 6.3 Hz), 125.8, 123.4, 112.3 (t, ^1^*J*_F,C_ = 245.0 Hz), 62.5, 45.5 (2 C), 14.3; ^**19**^**F NMR** (376 MHz, CDCl_3_), δ (ppm): −98.2; **FT-IR** (cm^–1^, neat, ATR), 2947, 1771, 1595, 1268, 1136, 1104; **HRMS** (ESI+) *m*/*z*: [M + H]^+^ Calcd for C_12_H_15_ClF_2_NO_2_ 278.0754; found 278.0735.

#### 5-(*tert*-Butyl)-3,3-difluoroindolin-2-one **(27)**(24)

Orange solid (36.1 mg, 0.16 mmol, 55% yield from 44.7 mg of 4-(*tert*-butyl)aniline following GP2); Rf = 0.17 (hexane:ethyl acetate, 9:1); **mp**: 133–135 °C. ^**1**^**H NMR** (400 MHz, CDCl_3_), δ (ppm): 8.43 (bs, 1 H), 7.58 (d, ^4^*J*_H,H_ = 4.0 Hz, 1H), 7.48 (dd, ^3^*J*_H,H_ = 8.0 Hz, ^4^*J*_H,H_ = 4.0 Hz, 1H), 6.89 (d, ^3^*J*_H,H_ = 8.0 Hz, 1 H), 1.32 (s, 9 H); ^**13**^**C{**^**1**^**H} NMR** (100 MHz, CDCl_3_), δ (ppm): 167.5 (t, ^2^*J*_F,C_ = 30.0 Hz), 147.8 (t, ^4^*J*_F,C_ = 2.0 Hz), 138.6 (t, ^3^*J*_F,C_ = 8.0 Hz), 130.7, 122.2, 120.2 (t, ^2^*J*_F,C_ = 22.0 Hz), 111.4 (t, ^1^*J*_F,C_ = 249.0 Hz), 111.3, 34.9, 31.5 (3C); ^**19**^**F NMR** (376 MHz, CDCl_3_), δ (ppm) −111.4; **FT-IR** (cm^–1^, neat, ATR), 3342, 3279, 2960, 1729, 1631, 1489, 1249, 1151; **HRMS** (ESI+) *m*/*z*: [M + Na]^+^ Calcd for C_12_H_13_F_2_NONa 248.0857; found 248.0851.

#### 5-(*tert*-Butyl)-3,3-difluoro-1-methylindolin-2-one **(28)**

Orange oil (46.7 mg, 0.2 mmol, 65% yield) from 44.7 mg of 4-(*tert*-butyl)-*N*,*N*-dimethylaniline. The product was prepared following GP2 and subsequently the reported procedure;^20^ Rf = 0.65 (hexane:ethyl acetate, 8:2). ^**1**^**H NMR** (400 MHz, CDCl_3_), δ (ppm): 7.59 (d, ^4^*J*_H,H_ = 4.0 Hz, 1H), 7.51 (dd, ^3^*J*_H,H_ = 8.0 Hz, ^4^*J*_H,H_ = 4.0 Hz, 1H), 6.83 (dd, ^3^*J*_H,H_ = 8.0 Hz, ^4^*J*_H,H_ = 4.0 Hz, 1 H), 3.20 (s, 3 H), 1.33 (s, 9 H); ^**13**^**C{**^**1**^**H} NMR** (100 MHz, CDCl_3_), δ (ppm): 165.8 (t, ^2^*J*_F,C_ = 30.0 Hz), 147.9, 141.8 (t, ^3^*J*_F,C_ = 7.0 Hz), 130.6, 122.1, 120.2 (t, ^2^*J*_F,C_ = 23.0 Hz), 111.6 (t, ^1^*J*_F,C_ = 248.0 Hz), 109.1, 35.1, 31.7 (3C), 26.6; ^**19**^**F NMR** (376 MHz, CDCl_3_), δ (ppm): −112.0; **FT-IR** (cm^–1^, neat, ATR), 2961, 1747, 1626, 1496, 1300, 1246, 1118; **HRMS** (ESI+) *m*/*z*: [M + H]^+^ Calcd for C_13_H_16_F_2_NO 240.1200; found 240.1216.

#### 2-(5-(*tert*-Butyl)-2-(dimethylamino)phenyl)-N,N-diethyl-2,2-difluoroacetamide **(30)**

Yellowish oil (39.1 mg, 0.12 mmol, 40% yield from 53.2 mg of 4-(*tert*-butyl)-*N*,*N*-dimethylaniline following GP2); Rf = 0.25 (hexane:ethyl acetate, 9:1). ^**1**^**H NMR** (400 MHz, CDCl_3_), δ (ppm): 7.69 (d, 1 H, ^4^*J*_H,H_ = 4.0 Hz), 7.45 (dd, 1 H, ^3^*J*_H,H_ = 8.0 Hz, ^4^*J*_H,H_ = 4.0 Hz), 7.20 (dd, 1 H, ^3^*J*_H,H_ = 8.0 Hz, ^4^*J*_H,H_ = 4.0 Hz), 3.37 (q, 2 H, ^3^*J*_H,H_ = 8.0 Hz), 3.10 (q, 2 H, ^3^*J*_H,H_ = 8.0 Hz), 2.55 (s, 6 H), 1.31 (s, 9 H), 1.16 (t, 3 H, ^3^*J*_H,H_ = 8.0 Hz), 0.85 (t, 3 H, ^3^*J*_H,H_ = 8.0 Hz); ^**13**^**C{**^**1**^**H} NMR** (100 MHz, CDCl_3_), δ (ppm): 163.1 (t, ^2^*J*_F,C_ = 29.0 Hz), 150.3 (t, ^3^*J*_F,C_ = 8.0 Hz), 148.0, 131.2 (t, ^2^*J*_F,C_ = 29.0 Hz), 128.5, 122.7 (t, ^3^*J*_F,C_ = 7.0 Hz), 122.0, 114.8 (t, ^1^*J*_F,C_ = 246.0 Hz), 45.9 (2 C), 41.8 (t, *J* = 3.0 Hz), 41.4, 34.7, 31.4 (3 C), 13.5, 12.6; ^**19**^**F NMR** (376 MHz, CDCl_3_), δ (ppm): −91.93; **HRMS** (ESI+) *m*/*z*: [M + H]^+^ Calcd for C_18_H_29_F_2_N_2_O 327.2242; found 327.2228.

#### Diethyl ((5-(*tert*-Butyl)-2-(dimethylamino)phenyl)difluoromethyl)phosphonate **(31)**

Yellow oil (29.1 mg, 0.08 mmol, 25% yield from 53.2 mg of 4-(*tert*-butyl)-*N*,*N*-dimethylaniline following GP2); Rf = 0.28 (hexane:ethyl acetate, 8:2). ^**1**^**H NMR** (400 MHz, CDCl_3_), δ (ppm): 7.60 (t, 1 H, ^4^*J*_H,H_ = 4.0 Hz), 7.45 (dt, 1 H, ^3^*J*_H,H_ = 12.0 Hz, *J* = 4.0 Hz), 7.28 (d, 1 H, ^3^*J*_H,H_ = 12.0 Hz), 4.20 (b, 4 H), 2.65 (s, 6 H), 1.32 (t, 6 H, ^3^*J*_H,H_ = 8.0 Hz), 1.31 (s, 9 H); ^**13**^**C{**^**1**^**H} NMR** (100 MHz, CDCl_3_), δ (ppm): 151.8, 147.6, 128.9, 124.5 (td, ^3^*J*_F,C_ = 12.0 Hz, ^3^*J*_P,C_ = 5.0 Hz), 122.6, 64.3 (d, 2 C, *J*_C,P_ = 7.0 Hz), 46.6 (2 C), 34.7, 31.4 (3 C), 16.5 (d, 2 C, *J*_P,C_ = 6.0 Hz), two quaternary carbons (CF_2_, α-CF_2_) are not seen due to oversplitting with nearby F and P; ^**19**^**F NMR** (376 MHz, CDCl_3_), δ (ppm): −98.90 (d, ^2^*J*_P,F_ = 154.2 Hz); ^**31**^**P NMR** (162 MHz, CDCl_3_), δ (ppm): 7.39 (tquint, ^2^*J*_F,P_ = 153.9 Hz, ^3^*J*_H,P_ = 9.7 Hz); **HRMS** (ESI+) *m*/*z*: [M + H]^+^ Calcd for C_17_H_29_F_2_NO_3_P 364.1848; found 364.1853.

#### 4-(*tert*-Butyl)-*N*,*N*-dimethyl-2-(perfluorohexyl)aniline **(32)**

Colorless oil (59.4 mg, 0.12 mmol, 40% yield from 53.2 mg of 4-(*tert*-butyl)-*N*,*N*-dimethylaniline following GP2); Rf = 0.64 (hexane). ^**1**^**H NMR** (300 MHz, CDCl_3_), δ (ppm): 7.55 (m, 2 H), 7.37 (dd, 1 H, ^3^*J*_H,H_ = 9.0 Hz, ^4^*J*_H,H_ = 3.0 Hz), 2.63 (s, 6 H), 1.33 (s, 9 H); ^**13**^**C{**^**1**^**H} NMR** (100 MHz, CDCl_3_), δ (ppm): 153.5, 148.2, 126.1 (t, ^3^*J*_F,C_ = 9.0 Hz), 125.3 (t, ^2^*J*_F,C_ = 21.0 Hz), 124.1, 108.5–119.5 (br, 6 C), 46.9 (2 C), 34.9, 31.5 (3 C); ^**19**^**F NMR** (282 MHz, CDCl_3_), δ (ppm): −80.9 (t, 3 F, ^3^*J*_F,F_ = 5.6 Hz), −104.4 (t, 2 F, ^3^*J*_F,F_ = 14.1 Hz), −120.32 (m, 2 F), −121.8 (m, 2 F), −122.7 (m, 2 F), −126.2 (m, 2 F); **HRMS** (ESI+) *m*/*z*: [M + H]^+^ Calcd for C_18_H_19_F_13_N 496.1304; found 496.1297.

## Data Availability

The data underlying this study are available in the published article and its Supporting Information.
