# Supplementary material for: Difluoroalkylation of Anilines via Photoinduced Methods
Source: J Org Chem. 2023 Aug 16;88(17):12585–96. doi: 10.1021/acs.joc.3c01298 (PMC10476199; doi:10.1021/acs.joc.3c01298)
Supplement: Supplementary file 1 — jo3c01298_si_001.pdf [file jo3c01298_si_001.pdf]

Supporting Information

Difluoroalkylation of Anilines *via* Photoinduced Methods

Albert Gallego-Gamo, Albert Granados, Roser Pleixats, Carolina Gimbert-Suriñach* and Adelina Vallribera*

Department of Chemistry and Centro de Innovación en Química Avanzada (ORFEO-CINQA), Universitat Autònoma de Barcelona, Cerdanyola del Vallès, 08193 Barcelona, Spain

*To whom correspondence should be addressed. e-mails: carolina.gimbert@uab.cat; adelina.vallribera@uab.cat

Table of Content

1. List of Anilines Used in This Study S2
2. Reaction Workflow for the Synthesis of Difluoroalkylanilines S3
3. Characterization of Compound **29** S3
4. Mechanistic Investigation S3
5. UV-Vis Spectra and Collected Data S14
6. Cyclic Voltammetry Experiments and Collected Data S19
7. NMR Spectra S24

1. List of Anilines Used in This Study

The starting *N*,*N*-dimethylaniline derivatives were synthetized by two modified methodologies found on the literature[1] except for 4-bromo-*N*,*N*-dimethylaniline, *N*,*N*-diethylaniline and 4-dimethylaminobenzaldehyde which were purchased from Sigma-Aldrich. The *ortho*-disubstituted anilines were commercially available.

mono substituted anilines

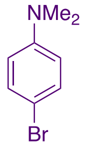

1a

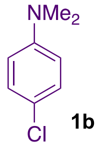

1b

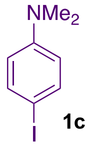

1c

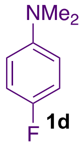

1d

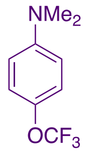

1e

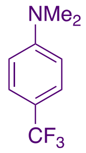

1f

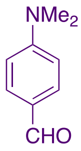

1g

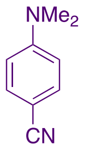

1h

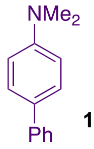

1i

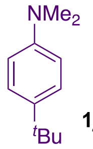

1j

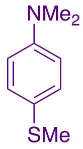

1k

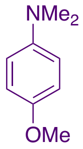

1l

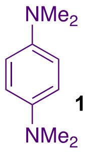

1m

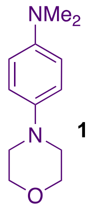

1n

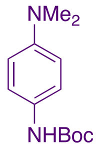

1o

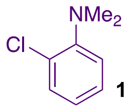

1p

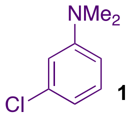

1q

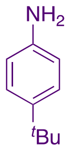

1r

disubstituted anilines

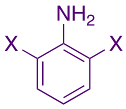

1s (X = F)
1t (X = Br)

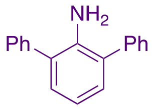

1u

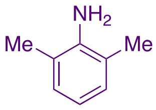

1v

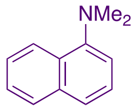

1w

unsubstituted anilines

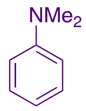

1x

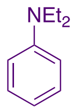

1y

[1] (a) Lewis, R. S.; Wisthoff, M. F.; Grissmerson, J.; Chain, W. J. *Org. Lett.* **2014**, *16*, 3832-3835. (b) Bush, T. S.; Yap, G. P. A.; Chain, W. J. *Org. Lett.* **2018**, *20*, 5406-5409

2. Reaction Workflow for the Synthesis of Difluoroalkylanilines

All photoredox reactions were performed with blue and green LED PR160L Kessil® (light-emitting diode, λ_{max} = 427 nm and λ_{max} = 525 nm respectively) employed at a distance of ~4 cm from the reaction vials. A fan was used to ensure reactions remained near room temperature within a ventilated fume hood. A typical reaction setup is shown below for 0.1-0.3 mmol and 1.0 mmol scale.

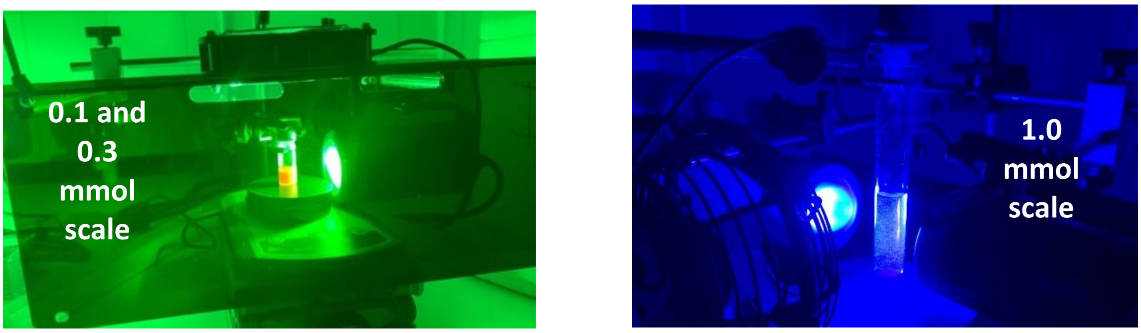

***Figure S1**: Reaction setups for the photoinduced formation of difluoroalkylanilines.*

3. Characterization of Compound 29

During the chromatographic purification of compound **25**, it converted to compound **29**.

Ethyl p-(N,N-diethylamino)phenylglyoxylate **(29)**.

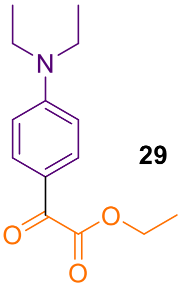

29

Isolated when purifiying compound **25**. Yellow oil (35 mg, 0.14 mmol, 46% yield from 44.8 mg of *N*,*N*-diethylaniline following GP1); R_f = 0.15 (hexane:ethyl acetate, 9.5:0.5). **^{1}H NMR** (400 MHz, $CDCl_3$), δ (ppm): 7.87 (d, 2 H, $^3J_{H2,H1}$ = 12.0 Hz), 6.65 (d, 2 H, $^3J_{H1,H2}$ = 12.0 Hz), 4.41 (q, 2 H, $^3J_{H,H}$ = 8.0 Hz), 3.44 (q, 4 H, $^3J_{H,H}$ = 8.0 Hz), 1.40 (t, 3 H, $^3J_{H,H}$ = 12.0 Hz), 1.21 (t, 6 H, $^3J_{H,H}$ = 12.0 Hz); **^{13}C{^{1}H} NMR** (100 MHz, $CDCl_3$), δ (ppm): 163.5 (t, $^2J_{F,C}$ = 34.0 Hz), 151.5 (t, $^3J_{F,C}$ = 5.0 Hz), 135.0, 132.3 (t, $^2J_{F,C}$ = 22.0 Hz), 129.4 (t, $^3J_{F,C}$ = 7.0 Hz), 124.5, 118.4, 111.7 (t, $^1J_{F,C}$ = 246.0 Hz), 62.4, 45.3 (2 C), 14.11; **FT-IR** (cm^{-1}, neat, ATR), 2976, 2934, 1731, 1584, 1175, 1151; **HR-MS** (ESI+) m/z: $[M+Na]^+$ Calcd for $C_{14}H_{19}NO_3Na$ 272.1257; found 272.1251.

4. Mechanistic Investigation

4.1. Protocol proceeding via Eosin Y

<u>TEMPO radical trapping</u>

Into a 4 mL vial equipped with a magnetic stirring bar, EOSIN Y (1.9 mg, 0.003 mmol, 0.01 equiv.), 4-(*tert*-butyl)-*N*,*N*-dimethylaniline (53.2 mg, 0.3 mmol, 1 equiv.), K_2CO_3 (62.2 mg, 0.45 mmol, 1.5 equiv.), TBAI (55.4 mg, 0.15 mmol, 0.5 equiv.), ICF_2COOEt (51 µL, 0.4 mmol, 1.3 equiv.) and TEMPO (62.5 mg, 0.4 mmol, 1.3 equiv.) were added. The vial was closed with a screw cap provided with a rubber septa and degassed by alternating vacuum evacuation and N_2 backfill. Then 1 mL of anhydrous DMF was added and the mixture was degassed again by Ar bubbling. After 5 min of degassing, the vial was well sealed with Parafilm®. The reaction mixture was stirred under Ar and irradiated by a 525 nm LED (PR160L Kessil®) at room temperature. After 24 h, the mixture was diluted with water and extracted with ethyl acetate (20 mL × 3). The organic mixture was washed with water (15 mL) and brine (15 mL × 2) then dried with anhydrous Na_2SO_4. The crude was analyzed by ^{1}H and ^{19}F NMR. The formation of compound **26** was confirmed and **12** was not observed.

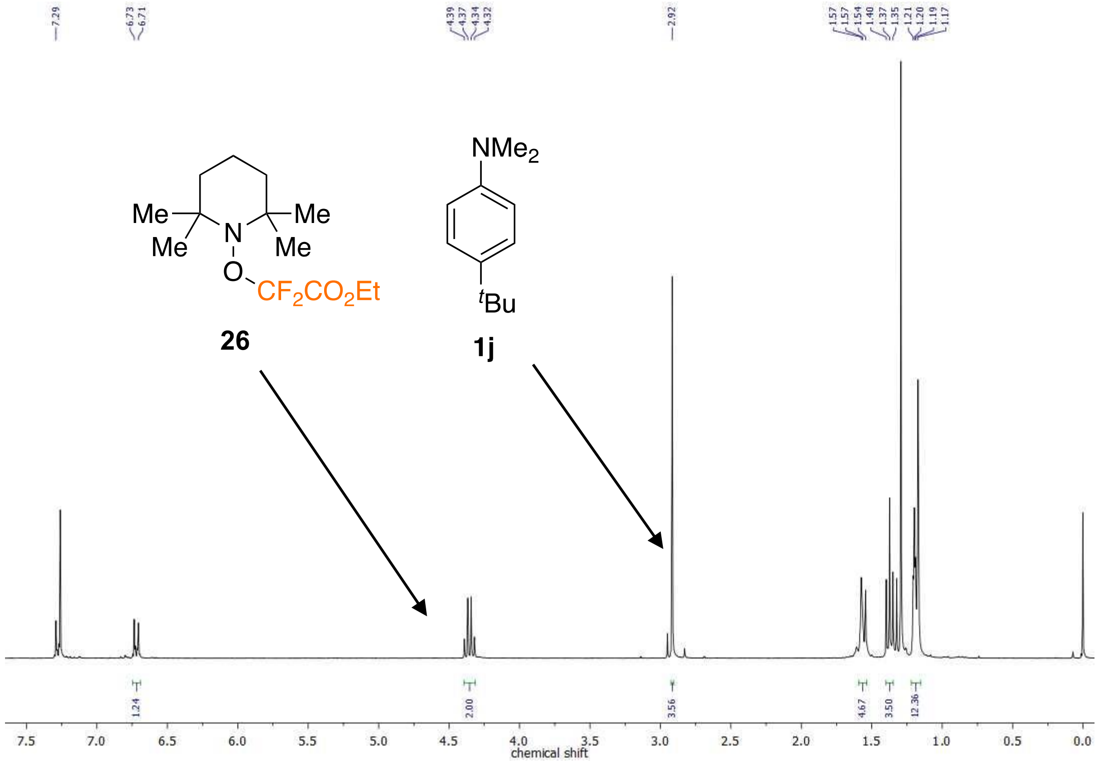

^{1}H NMR (300 MHz, $CDCl_3$) of the experiment with TEMPO

[2] Levitre, A.; Granados, A.; Cabrera-Afonso, M. J. Molander, G. A. *Org. Lett.* **2022**, *24*, 3194–3198.

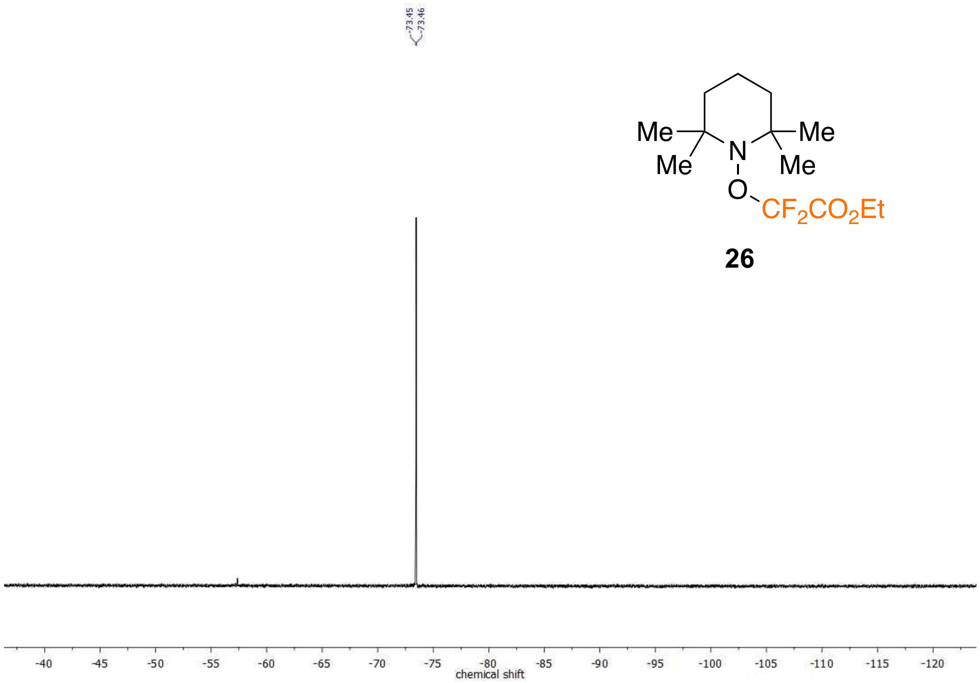

^{19}F NMR (235 MHz, $CDCl_3$) of compound **26** in the experiment with TEMPO

<u>Galvinoxyl radical trapping</u>

Into a 4 mL vial equipped with a magnetic stirring bar, EOSIN Y (1.9 mg, 0.003 mmol, 0.01 equiv.), 4-(*tert*-butyl)-*N*,*N*-dimethylaniline (53.2 mg, 0.3 mmol, 1 equiv.), K_2CO_3 (62.2 mg, 0.45 mmol, 1.5 equiv.), TBAI (55.4 mg, 0.15 mmol, 0.5 equiv.), ICF_2COOEt (51 µL, 0.4 mmol, 1.3 equiv.) and Galvinoxyl (168.6 mg, 0.4 mmol, 1.3 equiv.) were added. The vial was closed with a screw cap provided with a rubber septa and degassed by alternating vacuum evacuation and N_2 backfill. Then 1 mL of anhydrous DMF was added and the mixture was degassed again by Ar bubbling. After 5 min of degassing, the vial was well sealed with Parafilm®. The reaction mixture was stirred under Ar and irradiated by a 525 nm LED (PR160L Kessil®) at room temperature. After 24 h, the mixture was diluted with water and extracted with EtOAc (20 mL × 3). The organic mixture was washed with water (15 mL) and brine (15 mL × 2) then dried with anhydrous Na_2SO_4. The crude was analyzed by ^{19}F-NMR as well as HR-MS. The formation of compound **23** was detected and the formation of **12** was not observed.

Ethyl 2-(2,6-di-tert-butyl-4-((3,5-di-tert-butyl-4-oxocyclohexa-2,5-dien-1-ylidene)methyl)phenoxy)-2,2-difluoroacetate **(23)**

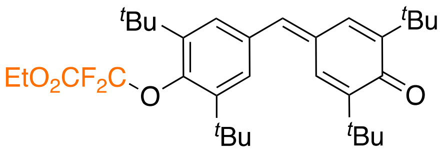

23

^{19}F NMR (235 MHz, $CDCl_3$), δ (ppm): -101.70 (CF_2); **HR-MS** (ESI+) m/z: $[M+Na]^+$ Calcd for $C_{33}H_{46}F_2O_4Na$ 567.3256; found 567.3235.

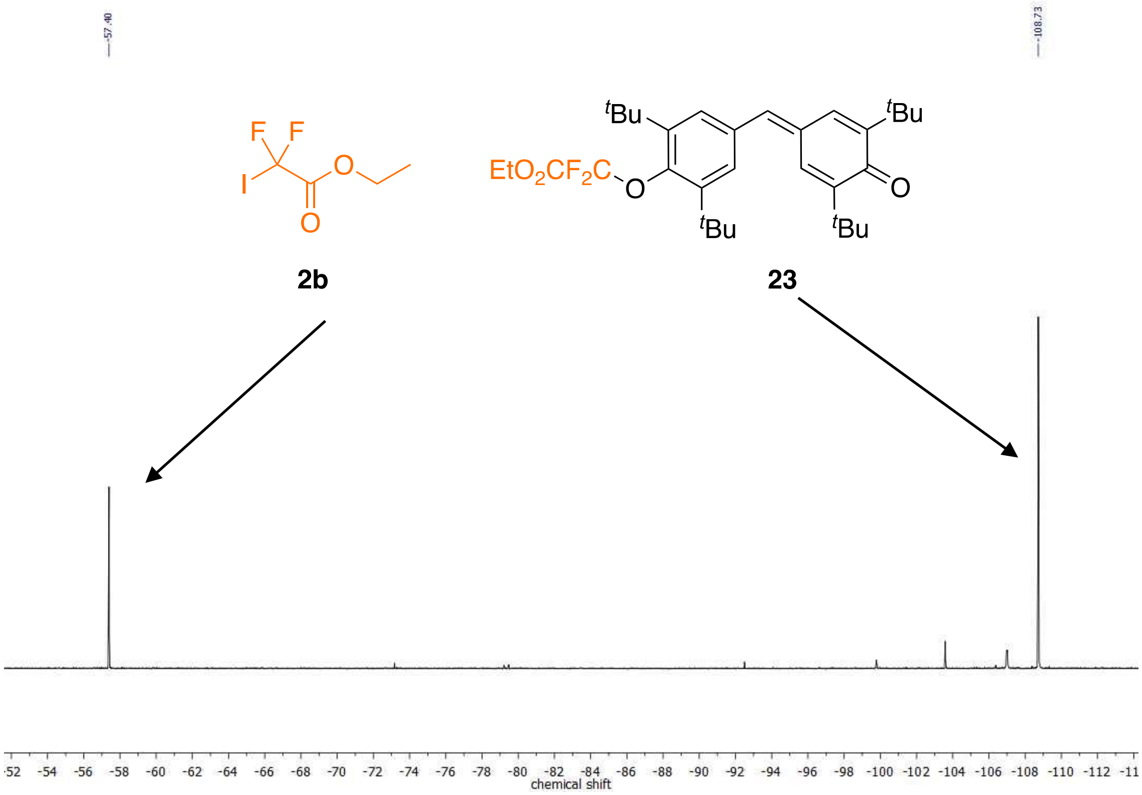

^{19}F NMR (235 MHz, $CDCl_3$) of experiment with Galvinoxyl

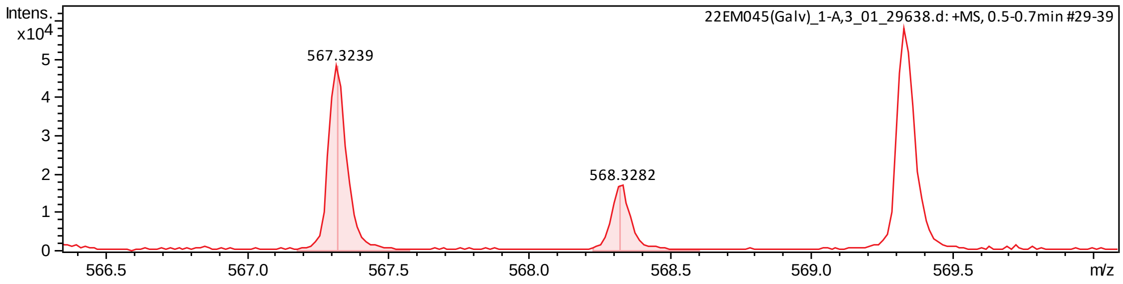

HR-MS (ESI+) of compound **23** in the experiment with Galvinoxyl

Diphenylethene radical trapping

Into a 4 mL vial equipped with a magnetic stirring bar, EOSIN Y (1.9 mg, 0.003 mmol, 0.01 equiv.), 4-(*tert*-butyl)-*N*,*N*-dimethylaniline (53.2 mg, 0.3 mmol, 1 equiv.), K_2CO_3 (62.2 mg, 0.45 mmol, 1.5 equiv.), TBAI (55.4 mg, 0.15 mmol, 0.5 equiv.), ICF_2COOEt (51 µL, 0.4 mmol, 1.3 equiv.) and 1,1-diphenylethene (72.1 mg, 0.4 mmol, 1.3 equiv.) were added. The vial was closed with a screw cap provided with a rubber septa and degassed by alternating vacuum evacuation and N_2 backfill. Then 1 mL of anhydrous DMF was added and the mixture was degassed again by Ar bubbling. After 5 min of degassing, the vial was well sealed with Parafilm®. The reaction mixture was stirred under Ar and irradiated by a 525 nm LED (PR160L Kessil®) at room temperature. After 24 h, the mixture was diluted with water and extracted with EtOAc (20 mL × 3). The organic mixture was washed with water (15 mL) and brine (15 mL × 2) then dried with anhydrous Na_2SO_4. The crude was analyzed by ^1H and ^{19}F-NMR. The formation of compounds **24** and **25** were confirmed and the formation of **12** was not observed.

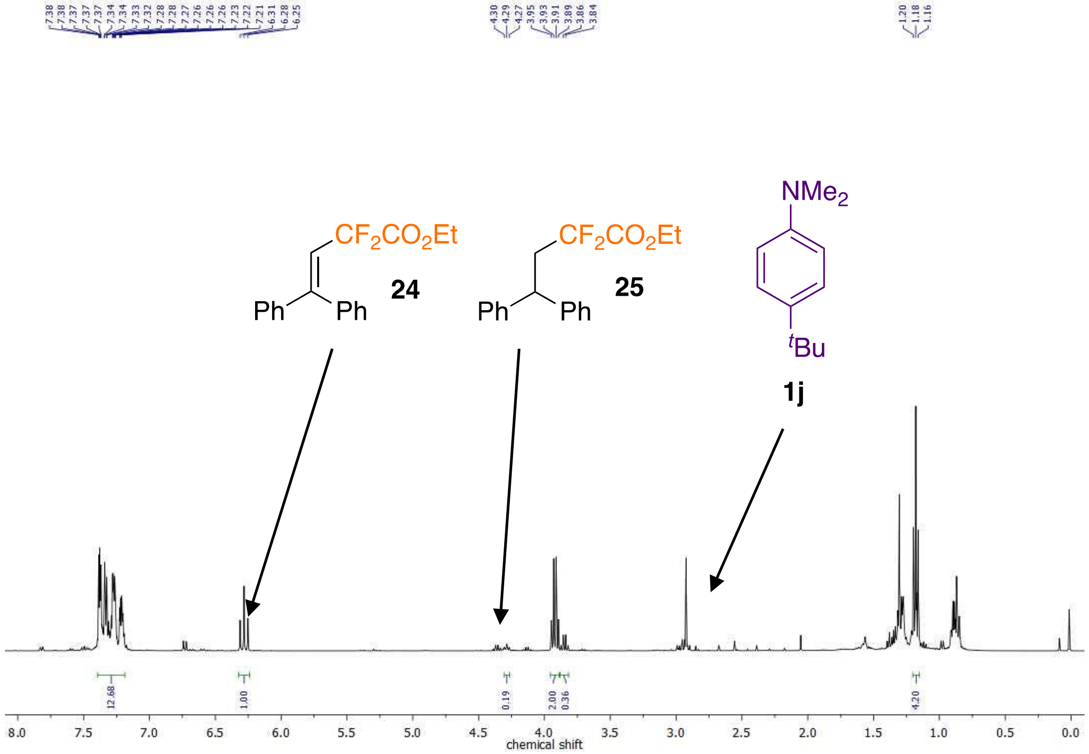

^1H NMR (300 MHz, $CDCl_3$) of the experiment with diphenylethene

[3] Zhu, Y.-Q.; Hui, L.-W.; Zhang, S.-B.; *Adv. Synth. Catal.* **2021**, *363*, 2170-2176.

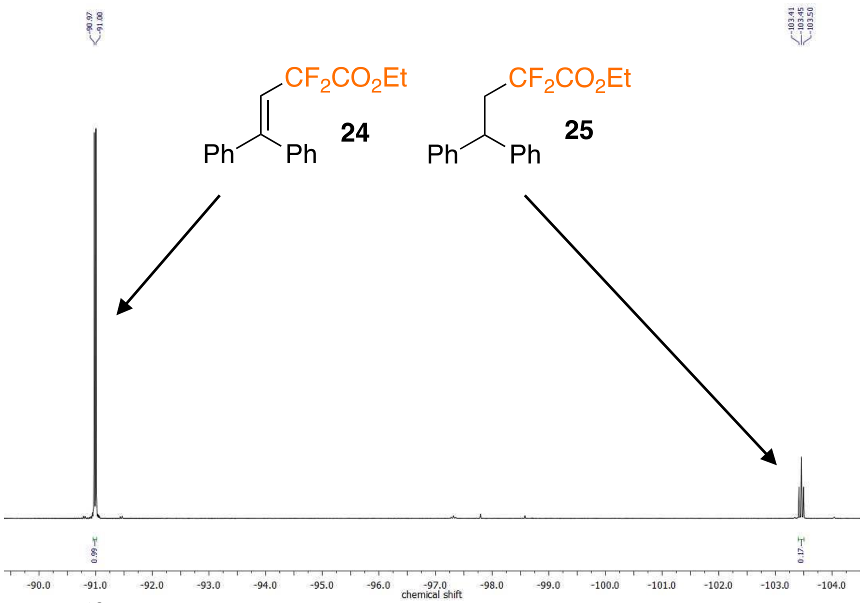

^{19}F NMR (235 MHz, $CDCl_3$) of compounds **24** and **25**

4.2. Protocol proceeding EDA Complex formation

<u>UV-Vis Studies</u>

A 0.1 M solution of **1a**, 0.13 M solution of **2b** and a mixture 1:1.3 of **1a**:**2b** in DMSO were prepared. UV-Vis absorption spectra were measured in a 1 cm quartz cuvette. Absorption spectra of individual reaction components and mixtures thereof were recorded. A bathochromic shift was observed for a mixture of aniline **1a** and fluorinated **2b** in DMSO, which was a visibly intense yellow in color (inset in Figure S2). This indicates the formation of an electron donor-acceptor (EDA) complex (Figure S2, orange band).

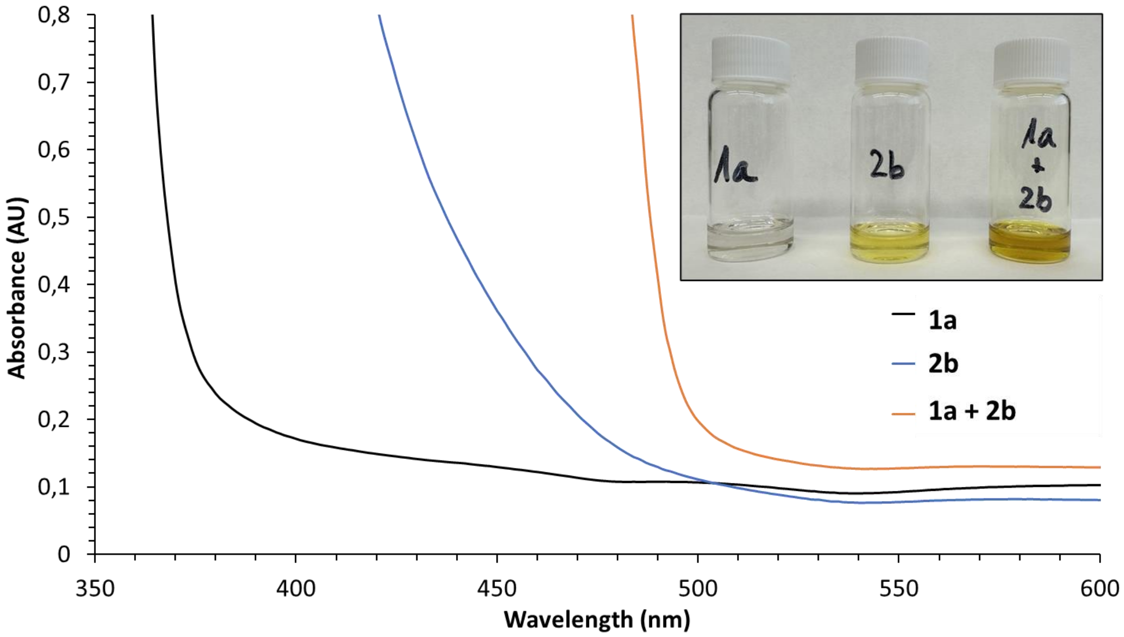

Figure S2: *UV-Vis absorption spectra of individual reaction components and a combination thereof. All spectra were measured in DMSO and with a concentration of 0.1 M aniline **1a** and 0.13 M **2b**. The stoichiometry and concentration of samples reflects the used reaction conditions.*

^{19}F NMR Titrations

^{19}F-NMR spectra of mixtures of **2b** and *N*,*N*-dimethyl-4-bromoaniline in $CDCl_3$ were recorded at 298 K. In an NMR tube, the total volume of the mixture was 0.6 mL, the concentration of **2b** (0.05 mmol) was kept constant at 0.08 M, and that of *N*,*N*-dimethyl-4-bromoaniline was varied from 0 to 0.32 M. A tube of hexafluorobenzene (-164.9 ppm) was used as external standard. The ^{19}F NMR signal of -CF_2I group in **2b** shifted upfield along increasing the amount of *N*,*N*-dimethyl-4-bromoaniline, indicating the formation of EDA complex between **2b** and *N*,*N*-dimethyl-4-bromoaniline (Figure S3).

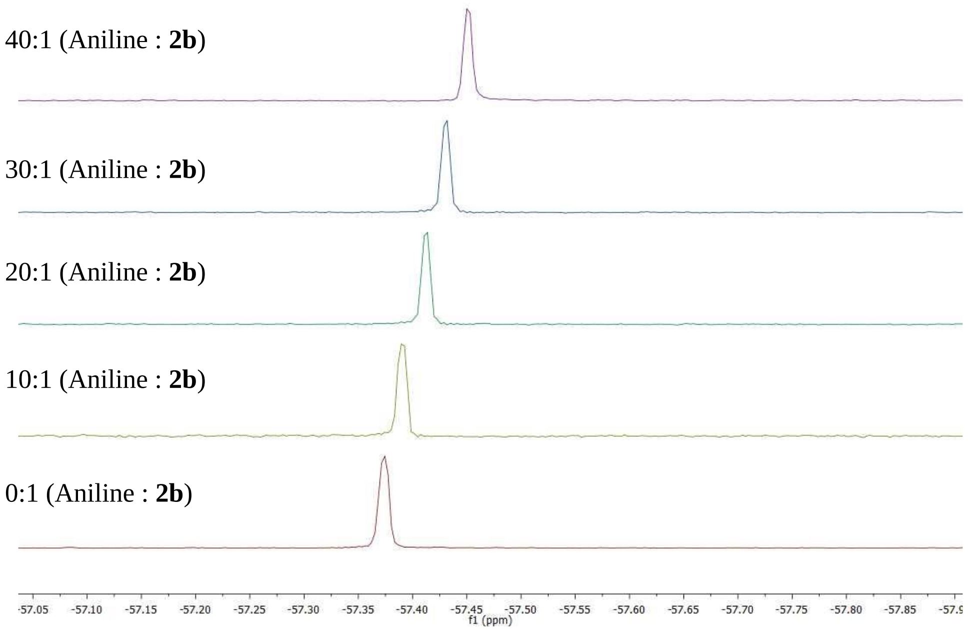

Figure S3: *^{19}F NMR shift of mixtures of **2b** with N,N-dimethyl-4-bromoaniline.*

<u>Quantum yield</u>

The quantum yield of the reaction was determined using the procedure reported previously:[4] **1a**, reagent **2b**, and Na_2CO_3 were used as model substrates to determinate the quantum yield of this transformation, using 1,3,5-trimethoxybenzene as internal standard in a proportion 1:1 with **1a**. The yield of the reaction after 1 h irradiation was 30%.

The quantum yield of the reaction is defined as:

$$\Phi(\text{reaction at 427 nm}) = \frac{\text{mol of formed product}}{\text{mol of photon flux} \cdot \text{t} \cdot \text{f}} \qquad (1)$$

where Φ is the quantum yield of the reaction, t is the time of the reaction (s), and f is the incident light absorbed by the EDA complex at 438 nm. The photon flux is calculated by standard ferrioxalate actinometry[5] (see below).

<u>Incident light absorbed by the EDA complex</u>

The fraction of light, f, absorbed was determined according to equation 2:

[4] El Khatib, M.; Serafim, R. A. M.; Molander, G. A. *Angew. Chem. Int. Ed.* **2016**, *55*, 254.
[5] Demas, J. N.; Bowman, W. D.; Zalewski, E. F.; Velapoidl, R. *J. Phys. Chem.* **1981**, *85*, 2766.

$$F = 1 - 10^{-A} \qquad (2)$$

Where A is the absorbance of the EDA complex in DMSO at 438 nm. The wavelength of 438 nm was chosen based on the known absolute $\Phi(Fe^{+2})$ value. The absorbance of EDA complex was measured (0.1 M **1a**, 0.15 M **2a**, and 0.15 M K_2CO_3) in DMSO (1 mL) to a cuvette. The absorbance was recorded. The absorbance (A) at 438 nm was determined to be >2, thus indicating the fraction of light absorbed is ~1 according to equation 2.

The photoredox reaction

The photoredox transformation was developed using the general procedure for 60 min (3600 s). Afterwards, 1,3,5-trimethoxybenzene was added as internal standard, and the reaction was worked up. The yield of the reaction was determined by ^1H NMR, where 0.03 mmols (30%) of the desired compound were obtained.

Photon flux at 438 nm

Standard ferrioxalate actinometry was used to determine the photon flux of the spectrophotometer using equations 3 and 4. For the ferrioxalate actinometer, the production of iron(II) ions proceeds by the following reactions:[5]

$$[Fe(C_2O_4)n]^{+(3-2n)} \xrightarrow{\text{light}} Fe^{+2} + (n-1)(C_2O_4)^{-2} + C_2O_4^{-}$$

$$[Fe(C_2O_4)n]^{+(3-2n)} + C_2O_4^{-} \longrightarrow Fe^{+2} + n(C_2O_4)^{-2} + 2\,CO_2$$

The moles of Fe^{+2} formed are determined spectrophotometrically by development with 1,10-phenanthroline (phen) to form the red $[Fe(phen)_3]^{+2}$ moiety (λ = 510 nm).[3] The photon flux is defined as shown in equation 3:

$$\text{Photon flux} = \frac{\text{mol } Fe^{+2}}{\Phi(Fe^{+2}) \cdot t \cdot f} \qquad (3)$$

where Φ is the quantum yield for the ferrioxalate actinometer (1.01 at λ = 438 nm),[4] t is the time (s), f ~1, and the mol of Fe^{+2} are calculated according to equation 4.

$$\text{mol } (Fe^{+2}) = \frac{V \cdot \Delta A}{l \cdot \epsilon} \qquad (4)$$

where V is the total volume of the solution, ΔA is the difference in absorbance between irradiated and nonirradiated solutions, l is the path length (1.0 cm), and ε is the molar absorptivity at 510 nm (11110 L mol^{-1} cm^{-1}).[5]

Experimental

The following solutions were prepared in the dark (flasks were wrapped in aluminum foil) and stored in the dark at room temperature:

– Ferrioxalate solution (0.15 M): Potassium ferrioxalate hydrate (0.65 g) was added to a flask wrapped in aluminum foil containing H_2SO_4 (10 mL, 0.05 M). The flask was stirred for complete solvation of the green solid in complete darkness. It is noteworthy that the solution should not be exposed to any incident light.

– Developer solution: 1,10-Phenanthroline (50 mg) and NaOAc (11.25 g) was added to a flask containing H_2SO_4 (50 mL, 0.5 M) and sonicated until completely solvated.

The absorbance of the non-irradiated sample. The buffered solution of phen (350 µL) was added to a ferrioxalate solution (2.0 mL) in a vial that had been covered with aluminum foil and with the lights of the laboratory switched off. The vial was capped and allowed to rest for 1 h and then transferred to a cuvette. The absorbance of the non-irradiated solution was measured at 510 nm to be 0.02 (see Figure S4).

The absorbance of the irradiated sample. In a cuvette equipped with a stir bar was added the ferrioxalate solution (2.0 mL), and the stirred solution was irradiated for 90 s at λ = 427 nm with an excitation slit width = 10.0 nm. After irradiation, the buffered phen solution (350 µL) was added to the cuvette and allowed to rest for 1 h in the dark to allow the ferrous ions to coordinate completely to phen. The absorbance was measured at 510 nm to be 1.32 (average of two determinations, Figure S4).

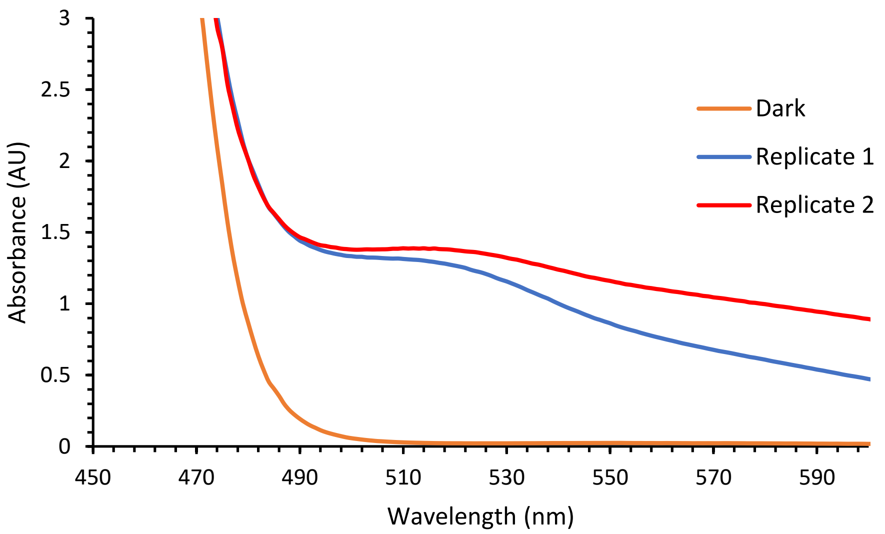

Figure S4. Absorption spectra for irradiated and non-irradiated samples of red $[Fe(phen)_3]^{+2}$

Photon flux sample calculation:

$$\text{mol }(Fe^{+2}) = \frac{V \cdot \Delta A}{l \cdot \epsilon} \qquad (4)$$

$$\text{mol }(Fe^{+2}) = \frac{0.00235\ L \cdot 1.32}{1.0\ cm \cdot 11100\ L \cdot mol^{-1} cm^{-1}} = 2.80x10^{-7} mol$$

$$\text{Photon flux} = \frac{\text{mol } Fe^{+2}}{\Phi(Fe^{+2}) \cdot t \cdot f} \qquad (3)$$

$$\text{Photon flux} = \frac{2.80x10^{-7} mol}{1.01 \cdot 90\ s \cdot 1} = 3.08x10^{-9}\ \text{einstein}\ s^{-1}$$

<u>Quantum yield of the photoinduced transformation</u>

Therefore, the quantum yield of the reaction was determined to be:

$$\Phi(\text{reaction at 427 nm}) = \text{ mol of formed} \frac{\text{product}}{\text{mol of photon flux} \cdot t \cdot f} \qquad (1)$$

$$\mathbf{\Phi(reaction\ at\ 427\ nm)} = \frac{3x10^{-5}\ mol}{3.08x10^{-9}\ \text{einstein}\ s^{-1} \cdot 3600\ s \cdot 1} = \mathbf{2.7}$$

The quantum yield studies indicate that this is a radical-chain process as evidenced by the Φ value. In other words, the quantum yield value indicated that 2.7 equivalents of product are formed for every photon absorbed, which is a result that could only be consistent with a radical chain mechanism.

5. UV-Vis Spectra and Collected Data

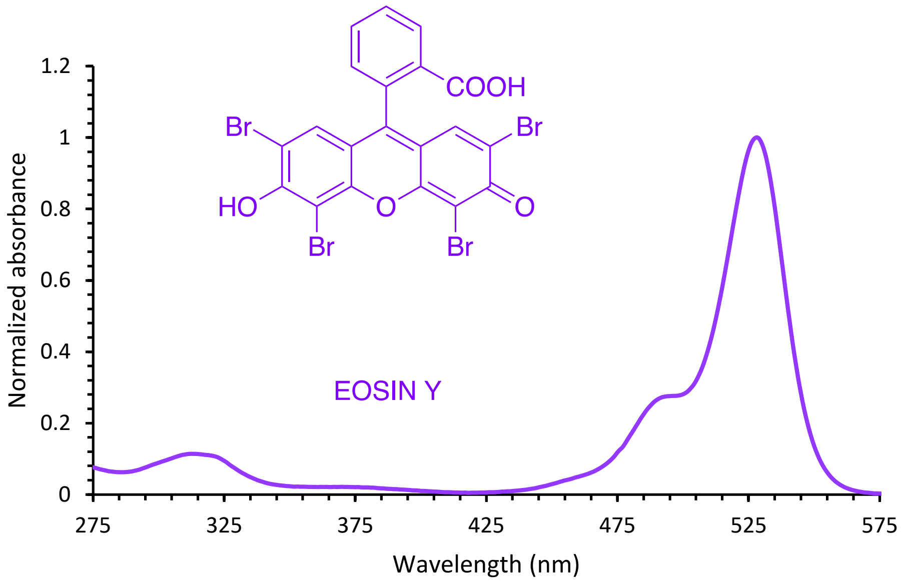

Figure S5: *Recorded UV-Vis spectra of the photosensitizer EOSIN Y (1.0 · 10^{-5} M).*

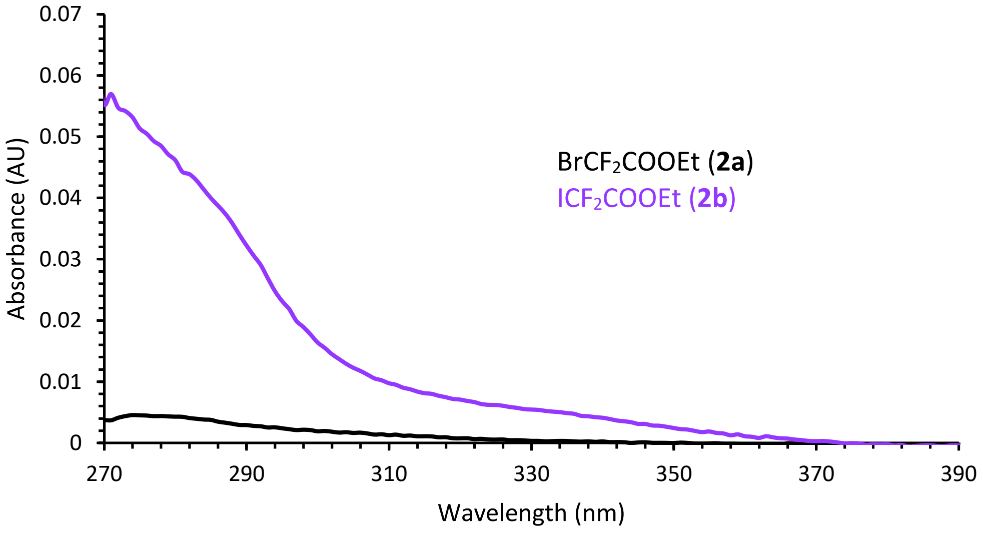

Figure S6: *UV-Vis spectra for reagents **2a** (1.0 · 10^{-5} M) and **2b** (1.0 · 10^{-6} M) in DMF.*

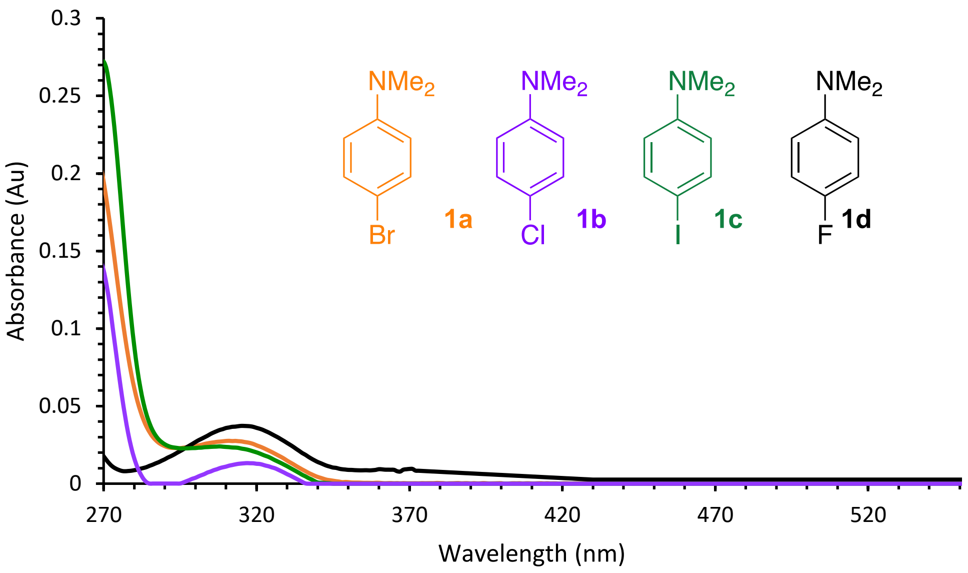

Figure S7: *UV-Vis spectra for anilines **1a-1d. 1a** (1.0 · 10^{-5} M), **1b** (1.0 · 10^{-5} M), **1c** 1.2 · 10^{-5} M) and **1d** (1.0 · 10^{-5} M).*

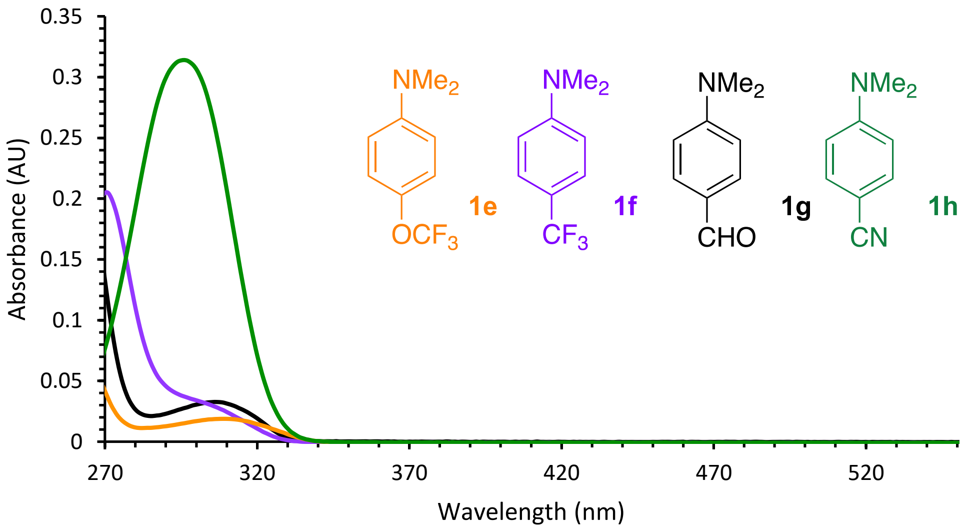

Figure S8: *Recorded UV-Vis spectra for anilines **1e-1h**. **1e** (1.1 · 10^{-5} M), **1f** (1.0 · 10^{-5} M), **1g** (1.0 · 10^{-5} M) and **1h** (1.60 · 10^{-5} M).*

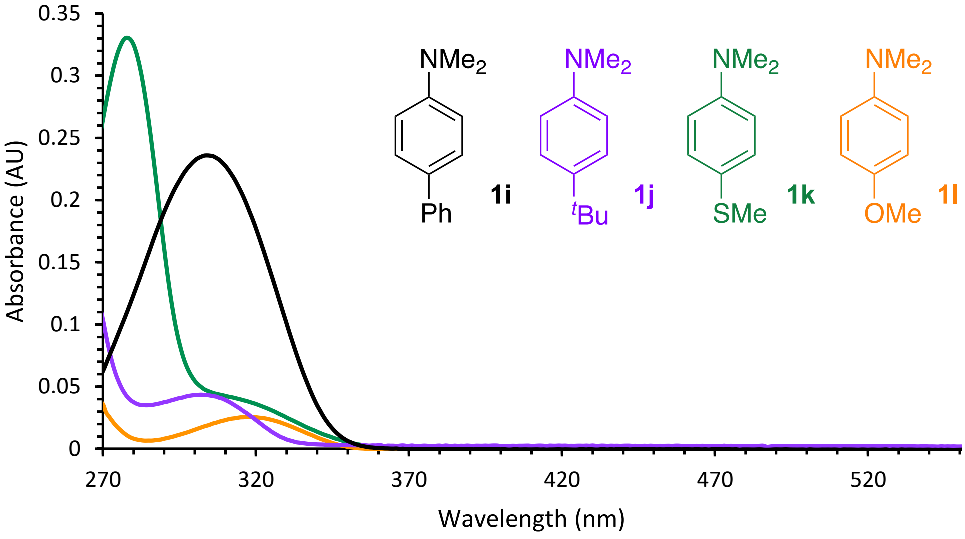

Figure S9: *Recorded UV-Vis spectra for anilines* ***1i-1j****.* ***1i*** *(1.0 · 10*$^{-5}$ *M),* ***1j*** *(1.0 · 10*$^{-5}$ *M),* ***1k*** *(1.0 · 10*$^{-5}$ *M) and* ***1l*** *(9.9 · 10*$^{-6}$ *M).*

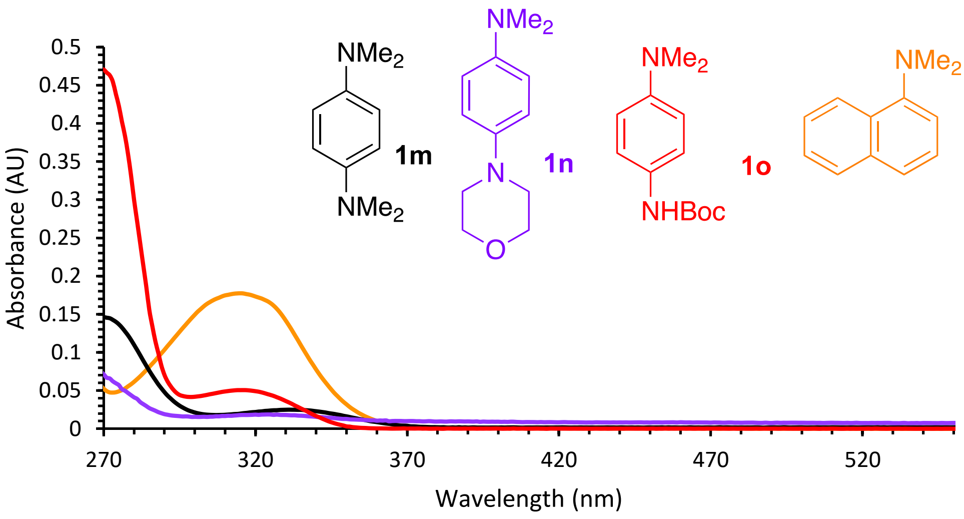

Figure S10: *Recorded UV-Vis spectra for anilines* ***1m-1o*** *and* ***1t****.* ***1m*** *(7.9 · 10*$^{-6}$ *M),* ***1n*** *(1.5 · 10*$^{-5}$ *M),* ***1o*** *(2.0 · 10*$^{-5}$ *M) and* ***1t*** *(5.3 · 10*$^{-5}$ *M).*

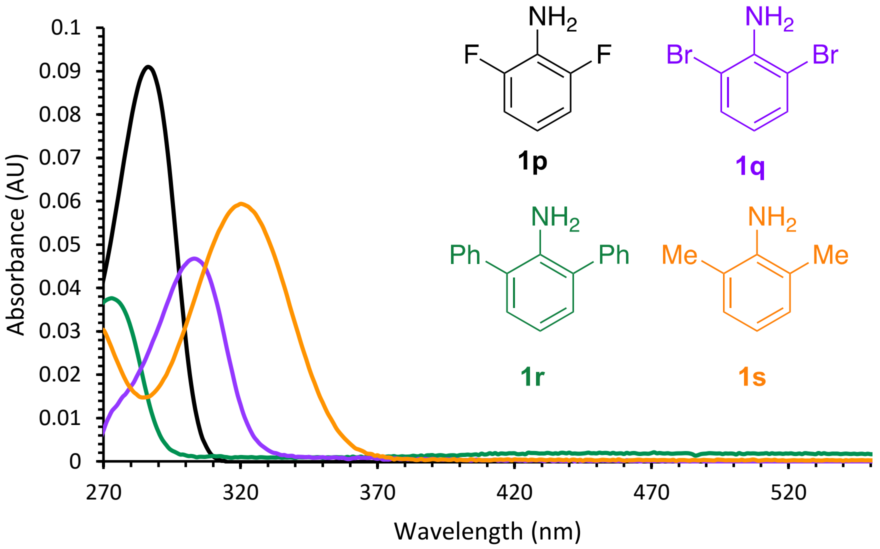

Figure S11: *Recorded UV-Vis spectra for anilines* ***1p-1s****.* ***1p*** *(1.0 · 10^{-5} M),* ***1q*** *(1.1 · 10^{-5} M),* ***1r*** *(9.35 · 10^{-6} M) and* ***1s*** *(1.0 · 10^{-5} M).*

Table S1: *UV-Vis data collection of photosensitizers and reagents.*

Reagent	λ_{max} (nm)	ε ($M^{-1}cm^{-1}$)
EOSIN Y	538	25783
$BrCF_2COOEt$ ***(2a)***	274	455
ICF_2COOEt ***(2b)***	330	5440
4-Fluoro-N,N-dimethylaniline ***(1d)***	315	3720
4-Chloro-N,N-dimethylaniline ***(1b)***	317	1302
4-Bromo-N,N-dimethylaniline ***(1a)***	313	2317
4-Iodo-N,N-dimethylaniline ***(1c)***	308	2405
N,N-Dimethyl-[1,1'-biphenyl]-4-amine ***(1i)***	304	23346
4-tert-butyl-N,N-dimethylaniline ***(1j)***	302	4356
4-Methoxy-N,N-dimethylaniline ***(1l)***	317	2603
N,N-Dimethyl-4-(methylthio)benzenamine ***(1k)***	320	3610
4-Dimethylaminobenzaldehyde ***(1g)***	306	3038
N,N-Dimethyl-4-(trifluoromethyl)benzenamine ***(1f)***	305	3001
N,N-Dimethyl-4-(trifuloromethoxy)benzenamine) ***(1e)***	309	1884
4-(Dimethyl)benzonitrile ***(1h)***	296	19630
N^1,N^1,N^4,N^4-tetramethylbenzene-1,4-diamine ***(1m)***	331	3162
N,N-dimethyl-4-morpholinoaniline ***(1n)***	323	1294
tert-butyl (4-(dimethylamino)phenyl)carbamate ***(1o)***	316	2560
2,6-Difluoroaniline ***(1p)***	286	9092
2,6-Dibromoaniline ***(1q)***	303	4371
2,6-Dimethylaniline ***(1s)***	273	3765
2,6-Diphenylaniline ***(1r)***	320	6349
N,N-dimethylnaphthalen-1-amine ***(1t)***	315	17734

6. Cyclic Voltammetry Experiments and Collected Data

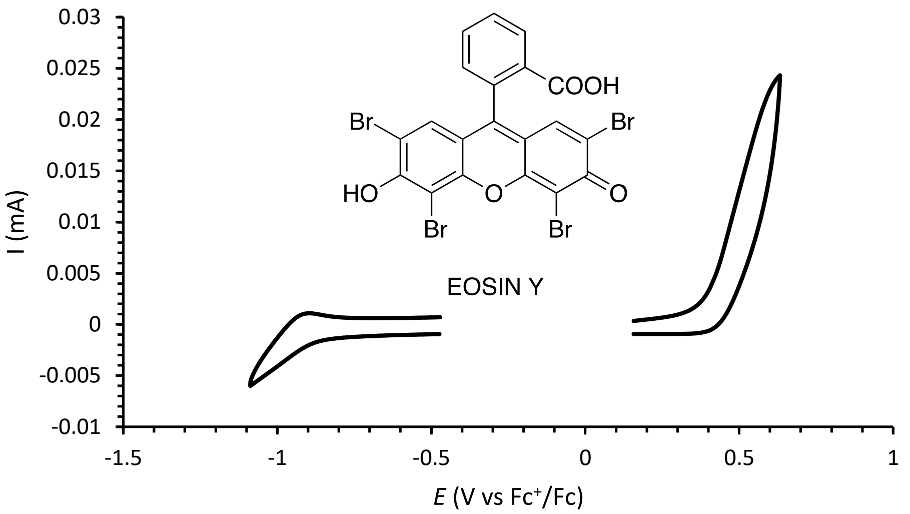

Figure S12: *Cyclic voltammetry of EOSIN Y. Conditions: 0.6 mM (500mV/s), DMF, TBAPF$_6$ 0.1 M, r.t. Start at 0.0 V, scan direction to negative potentials for reduction; start at 0.0 V, scan direction to positive values for oxidation. Glassy carbon disk as working electrode, platinum wire as auxiliary electrode and AgNO$_3$/Ag as reference electrode. IUPAC plotting.*

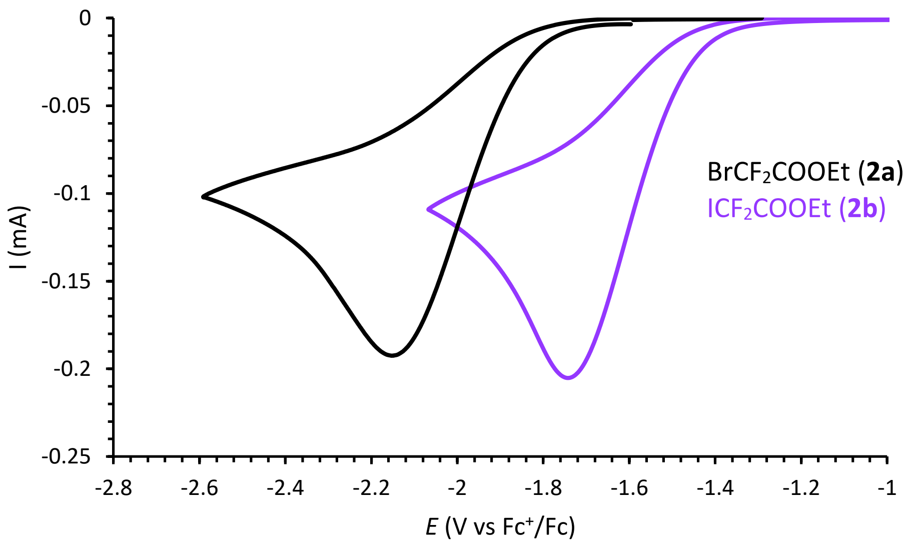

Figure S13: *Cyclic voltammetry of compounds **2a** and **2b**. Conditions: **2a** 3.9 mM (100mV/s), **2b** 3.9 mM (100 mV/s), in DMF, TBAPF$_6$ 0.1 M, r.t. Start at 0.0 V, scan direction to negative potentials. Glassy carbon disk as working electrode, platinum wire as auxiliary electrode and AgNO$_3$/Ag as reference electrode. IUPAC plotting.*

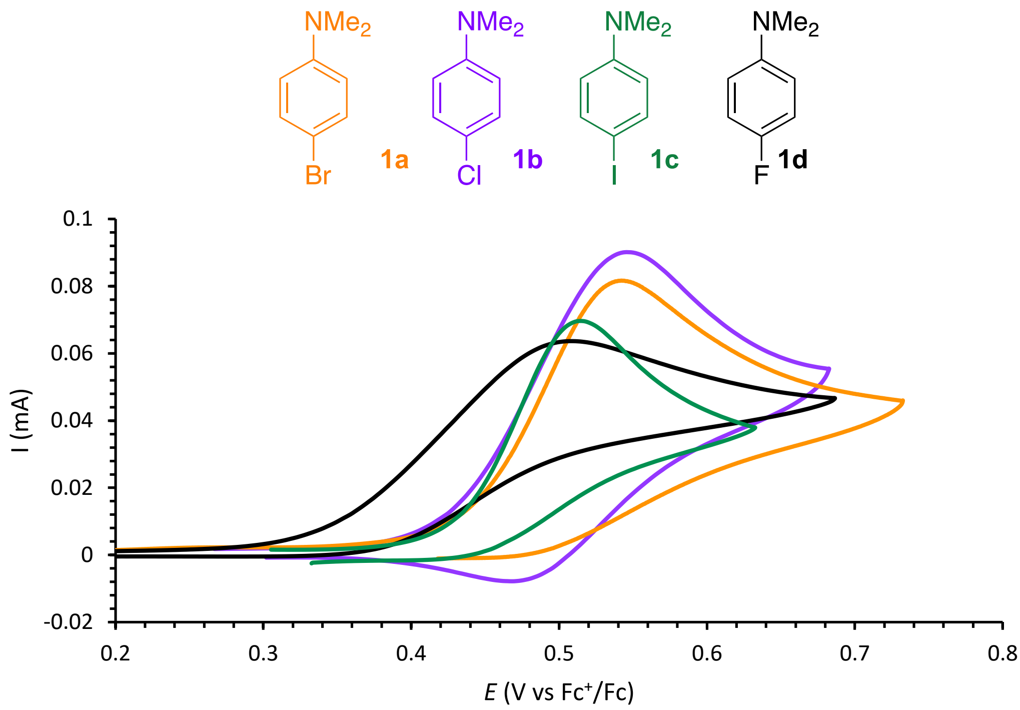

Figure S14: *Cyclic voltammetry experiments of compounds* ***1a-1d****. Conditions:* ***1a*** *2.5 mM (100mV/s),* ***1b*** *4.0 mM (100 mV/s),* ***1c*** *3.3 mM (100mV/s),* ***1d*** *2.5 mM (100 mV/s), in DMF, $TBAPF_6$ 0.1 M, r.t. Start at 0.0 V, scan direction to positive potentials. Glassy carbon disk as working electrode, platinum wire as auxiliary electrode and $AgNO_3$/Ag as reference electrode. IUPAC plotting.*

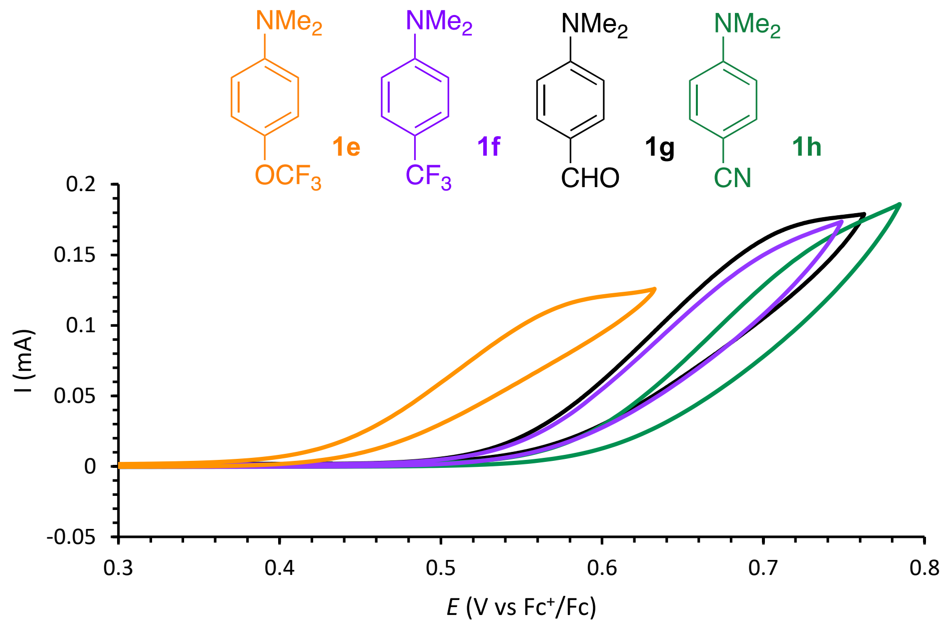

Figure S15: *Cyclic voltammetry experiments of compounds* ***1e-1h****. Conditions:* ***1e*** *4.0 mM (100mV/s),* ***1f*** *2.4 mM (100 mV/s),* ***1g*** *3.2 mM (100mV/s),* ***1h*** *2.4 mM (100 mV/s), in DMF, $TBAPF_6$ 0.1 M, r.t. Start at 0.0 V, scan direction to positive potentials. Glassy carbon disk as working electrode, platinum wire as auxiliary electrode and $AgNO_3$/Ag as reference electrode. IUPAC plotting.*

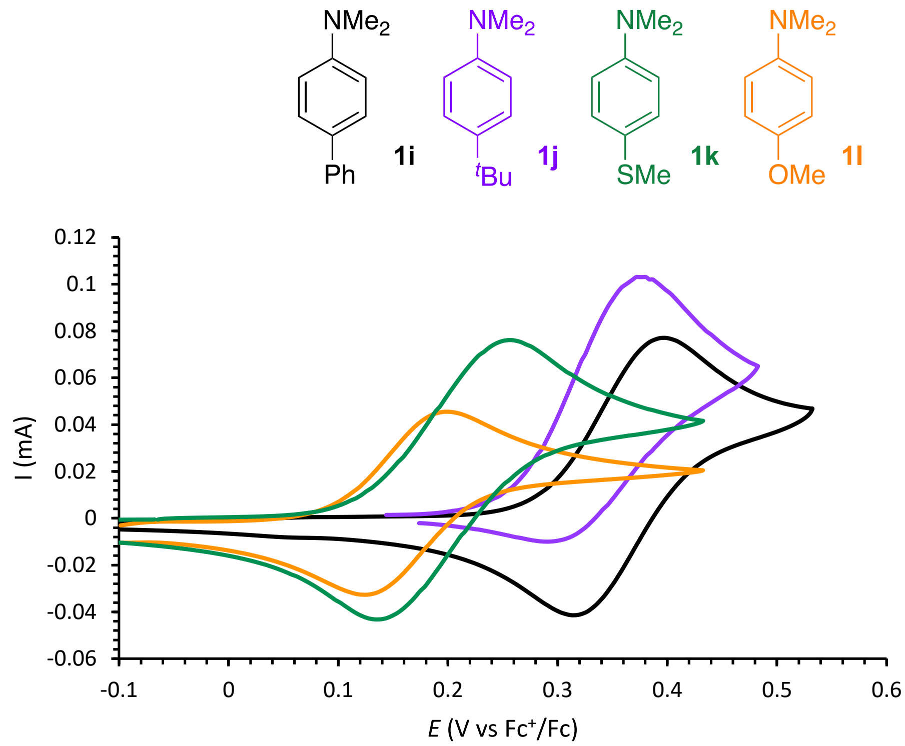

Figure S16: *Cyclic voltammetry experiments of compounds* ***1i-1l****. Conditions:* ***1i*** *3.9 mM (100mV/s),* ***1j*** *3.9 mM (100 mV/s),* ***1k*** *2.5 mM (100mV/s),* ***1l*** *2.4 mM (100 mV/s), in DMF, $TBAPF_6$ 0.1 M, r.t. Start at 0.0 V, scan direction to positive potentials. Glassy carbon disk as working electrode, platinum wire as auxiliary electrode and $AgNO_3$/Ag as reference electrode. IUPAC plotting.*

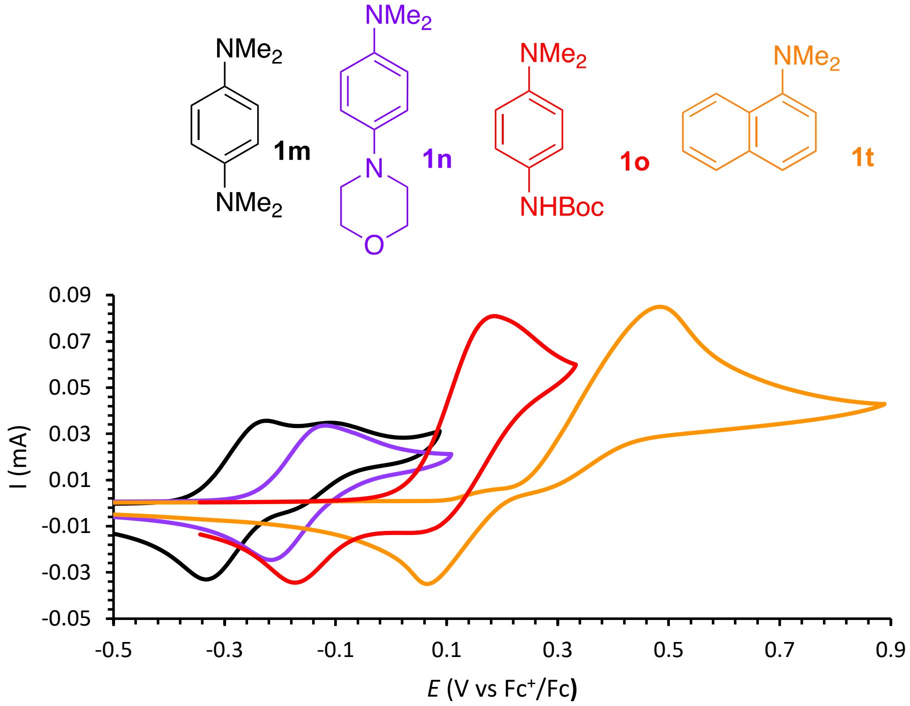

Figure S17: *Cyclic voltammetry experiments of compounds* ***1m****,* ***1n****,* ***1o*** *and* ***1t****. Conditions:* ***1m*** *2.6 mM (100mV/s),* ***1n*** *2.5 mM (100 mV/s),* ***1o*** *2.5 mM (100 mV/s),* ***1t*** *2.12 mM (100mV/s) in DMF, $TBAPF_6$ 0.1 M, r.t. Start at 0.0 V, scan direction to positive potentials. Glassy carbon disk as working electrode, platinum wire as auxiliary electrode and $AgNO_3$/Ag as reference electrode. IUPAC plotting.*

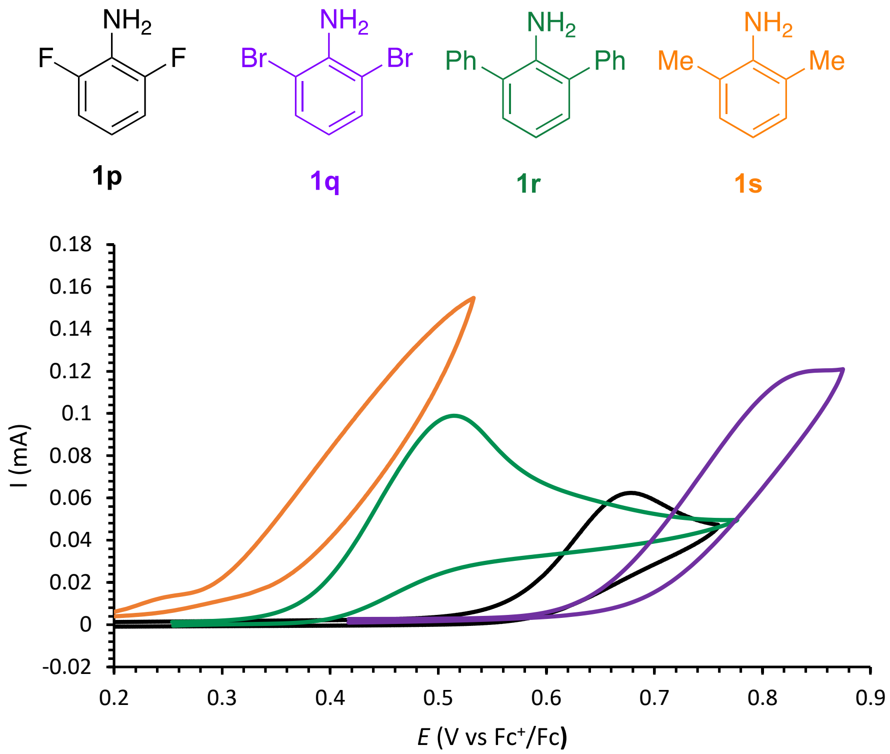

Figure S18: *Cyclic voltammetry experiments of compounds **1p-1s**. Conditions: **1p** 2.5 mM (100mV/s), **1q** 2.9 mM (100 mV/s), **1r** 2.4 mM (500mV/s), **1s** 2.6 mM (100 mV/s), in DMF, $TBAPF_6$ 0.1 M, r.t. Start at 0.0 V, scan direction to positive potentials. Glassy carbon disk as working electrode, platinum wire as auxiliary electrode and $AgNO_3$/Ag as reference electrode. IUPAC plotting.*

Table S2: *Experimental redox values extracted from CV experiments for EOSIN Y.*

Reagent	$E_{1/2}$ (P^+/P)	$E_{1/2}$ (P/P^-)	E_a	E_c	E (P^+/*P)	E (*P/P^-)
EOSIN Y	-	-	0.63	-1.09	-1.66	1.20

All values are given in V *vs* Fc^+/Fc. For those that presented irreversible waves, E_a or E_c are reported.

Calculus for the Excited State potentials were performed with the following equation:

1) $E_{1/2}$ (P^+/*P) = $E_{1/2}$ (P^+/P) – $E_{0\text{-}0}$
2) $E_{1/2}$ (*P/P^-) = $E_{1/2}$ (P/P^-) + $E_{0\text{-}0}$
3) E (eV) = 1.2398/λ (in µm), then $E_{0\text{-}0}$ is when $\lambda = (\lambda_{max,abs} + \lambda_{max,em})/2$ (*Rehem-Weller equation)*

Table S3: *Experimental redox values extracted from CV experiments for aniline scope*

Reagent	$E_{1/2}$ **(ox)**	$E_{1/2}$ **(red)**	E_a	E_c
BrCF₂COOEt ***(2a)***	-	-	-	-1.94
ICF₂COOEt ***(2b)***	-	-	0.22	-1.67
4-Fluoro-N,N-dimethylaniline ***(1d)***	-	-	0.51	-
4-Chloro-N,N-dimethylaniline ***(1b)***	-	-	0.53	-
4-Bromo-N,N-dimethylaniline ***(1a)***	-	-	0.58	-
4-Iodo-N,N-dimethylaniline ***(1c)***	-	-	0.50	-
N,N-Dimethyl-[1,1'-biphenyl]-4-amine ***(1i)***	0.34	-	-	-
4-tert-butyl-N,N-dimethylaniline ***(1j)***	-	-	0.36	-
4-Methoxy-N,N-dimethylaniline ***(1l)***	0.15	-	-	-
N,N-Dimethyl-4-(methylthio)benzenamine ***(1k)***	0.20	-	-	-
4-Dimethylaminobenzaldehyde ***(1g)***	-	-	0.74	-
N,N-Dimethyl-4-(trifluoromethyl)benzenamine ***(1f)***	-	-	0.75	-
N,N-Dimethyl-4-(trifuloromethoxy)benzenamine) ***(1e)***	-	-	0.63	-
4-(Dimethyl)benzonitrile ***(1h)***	-	-	0.78	-
N^1,N^1,N^4,N^4-tetramethylbenzene-1,4-diamine ***(1m)***	-0.27	-	-	-
N,N-dimethyl-4-morpholinoaniline ***(1n)***	-0.33	-	-	-
tert-butyl (4-(dimethylamino)phenyl)carbamate ***(1o)***	-	-	0.19	-
2,6-Difluoroaniline ***(1p)***	-	-	0.67	-
2,6-Dibromoaniline ***(1q)***	-	-	0.86	-
2,6-Dimethylaniline ***(1s)***	-	-	0.51	-
2,6-Diphenylaniline ***(1r)***	-	-	0.52	-
N,N-dimethylnaphthalen-1-amine ***(1t)***	-	-	0.51	-

All values are given in V *vs* Fc^+/Fc. For those that presented irreversible waves, E_a or E_c are reported.

7. NMR Spectra

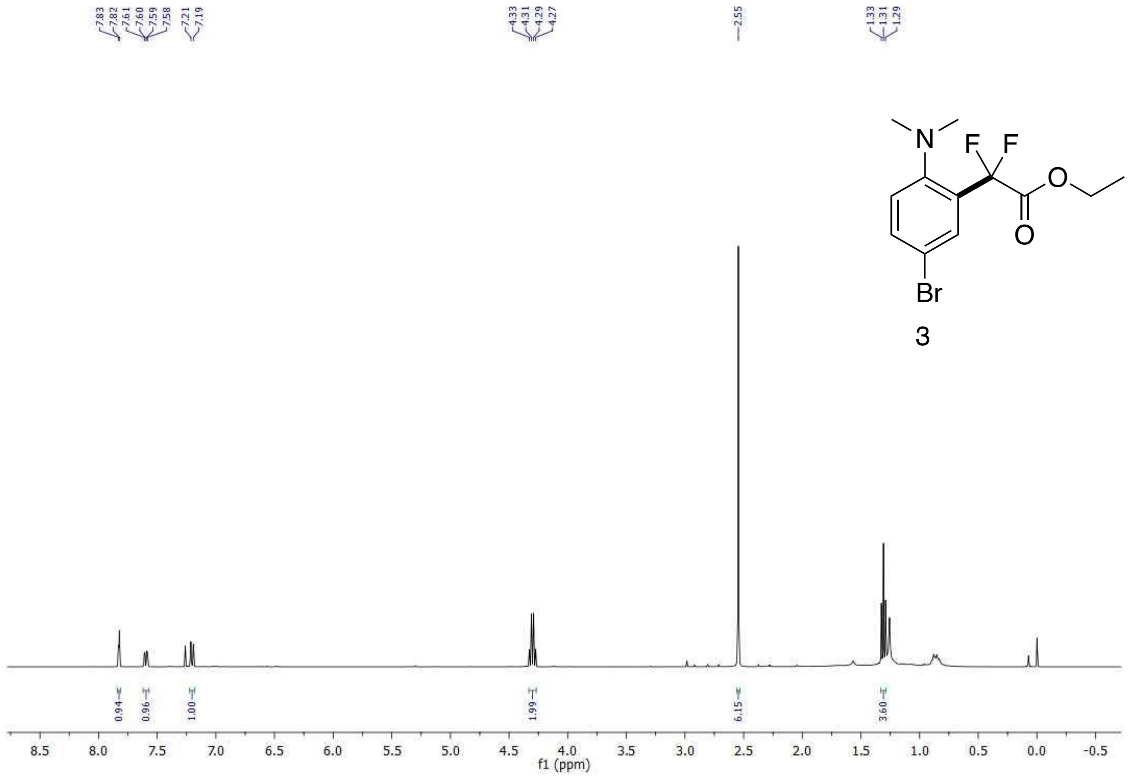

^{1}H NMR (400 MHz, $CDCl_3$) of compound **3**

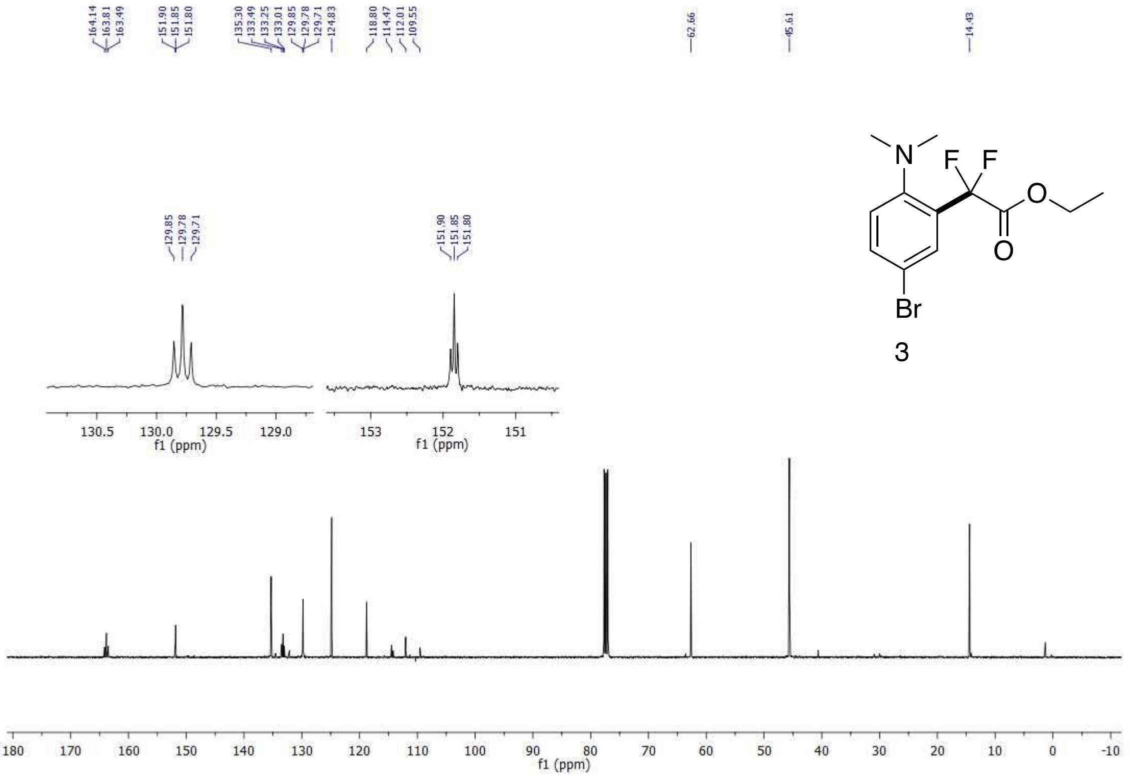

$^{13}C\{^{1}H\}$ NMR (100 MHz, $CDCl_3$) of compound **3**

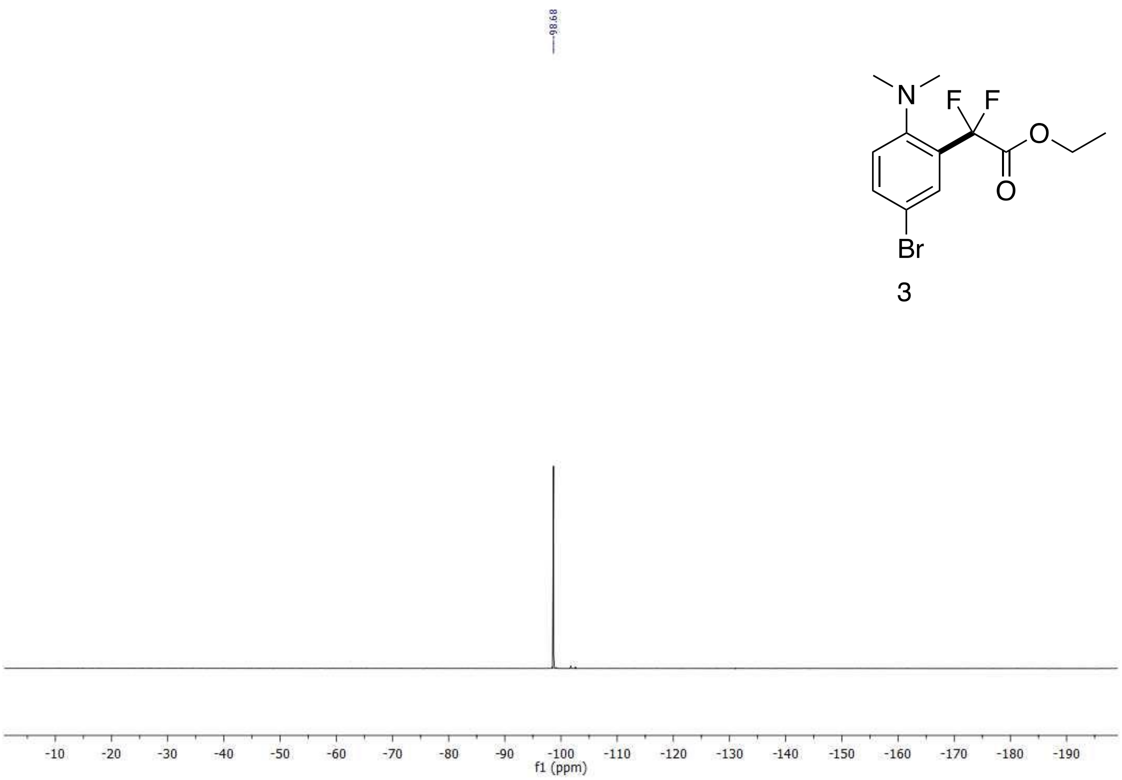

^{19}F NMR (235 MHz, $CDCl_3$) of compound **3**

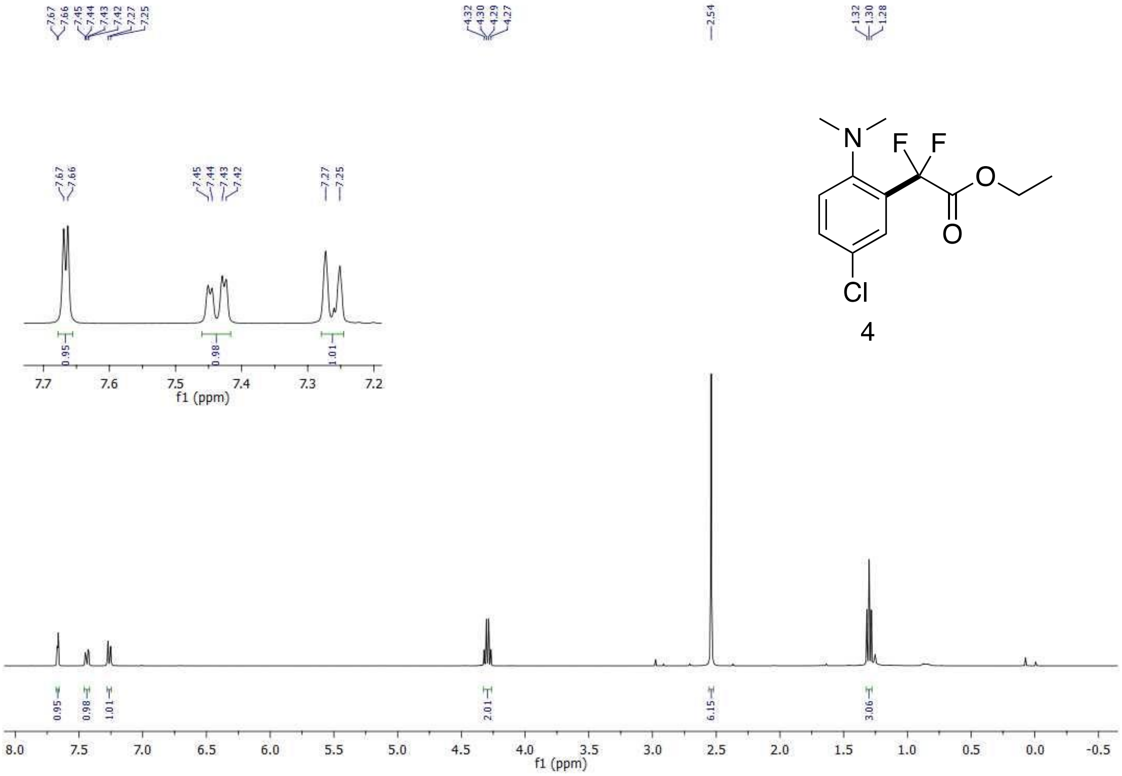

^{1}H NMR (400 MHz, $CDCl_3$) of compound **4**

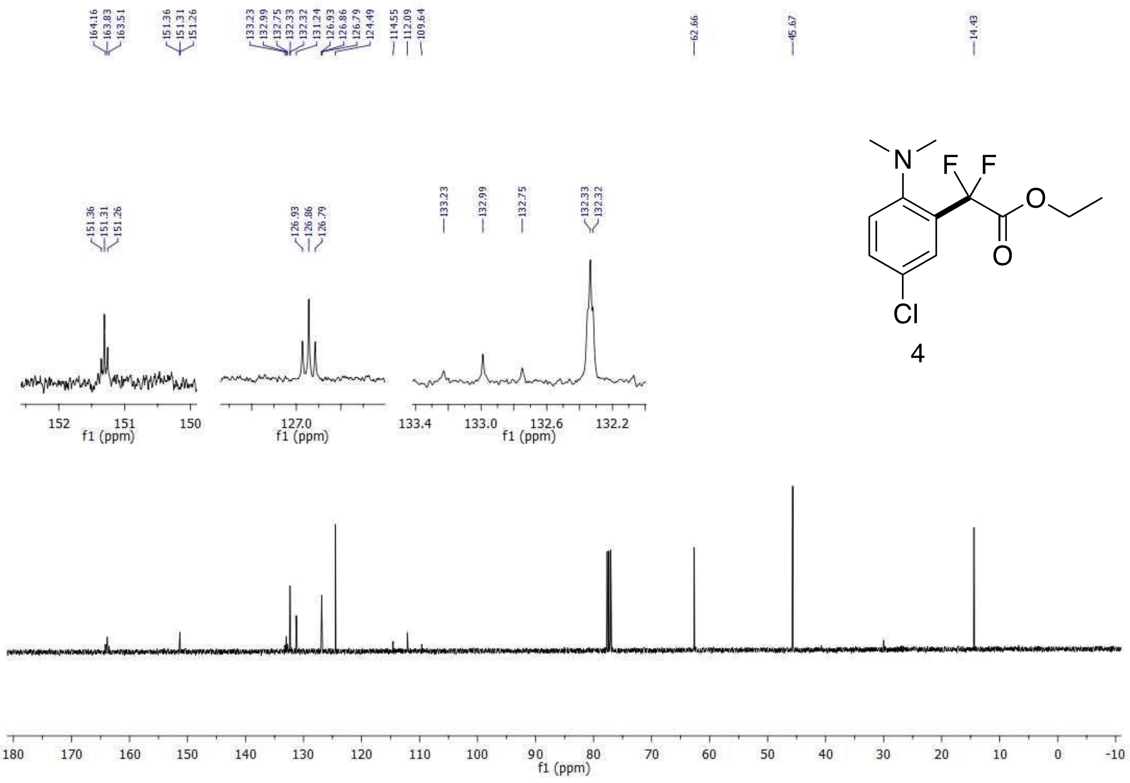

$^{13}C\{^{1}H\}$ NMR (100 MHz, $CDCl_3$) of compound **4**

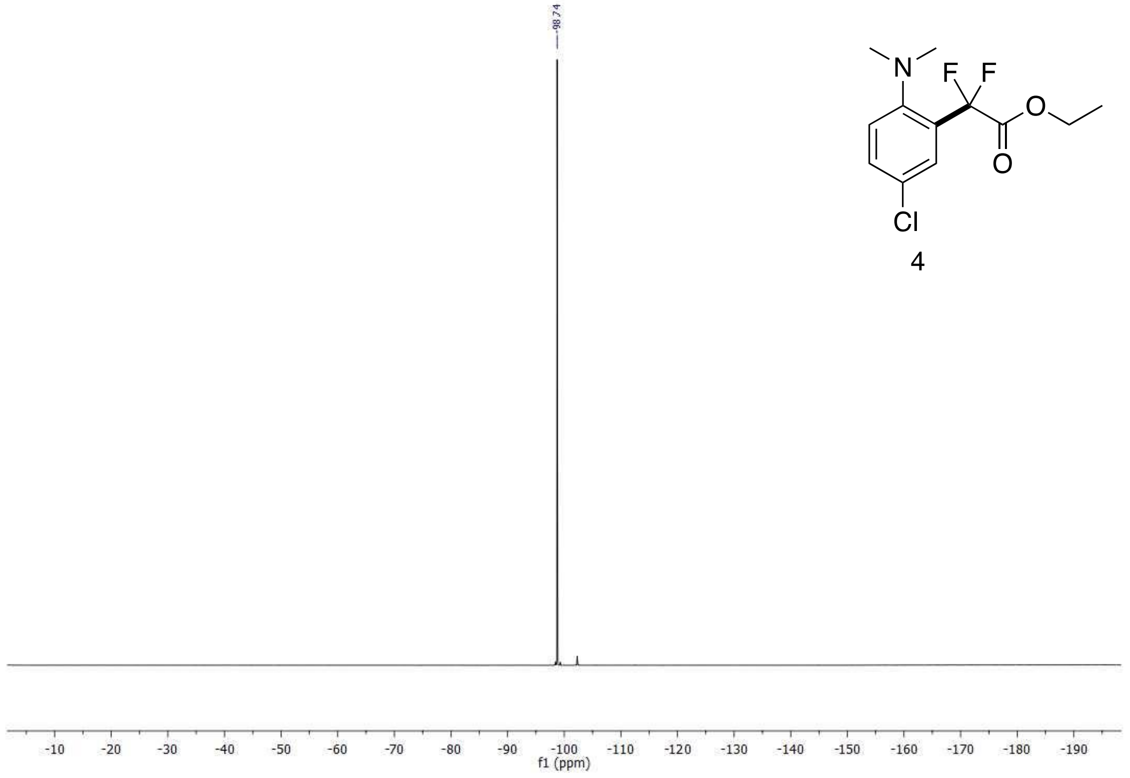

^{19}F NMR (282 MHz, $CDCl_3$) of compound **4**

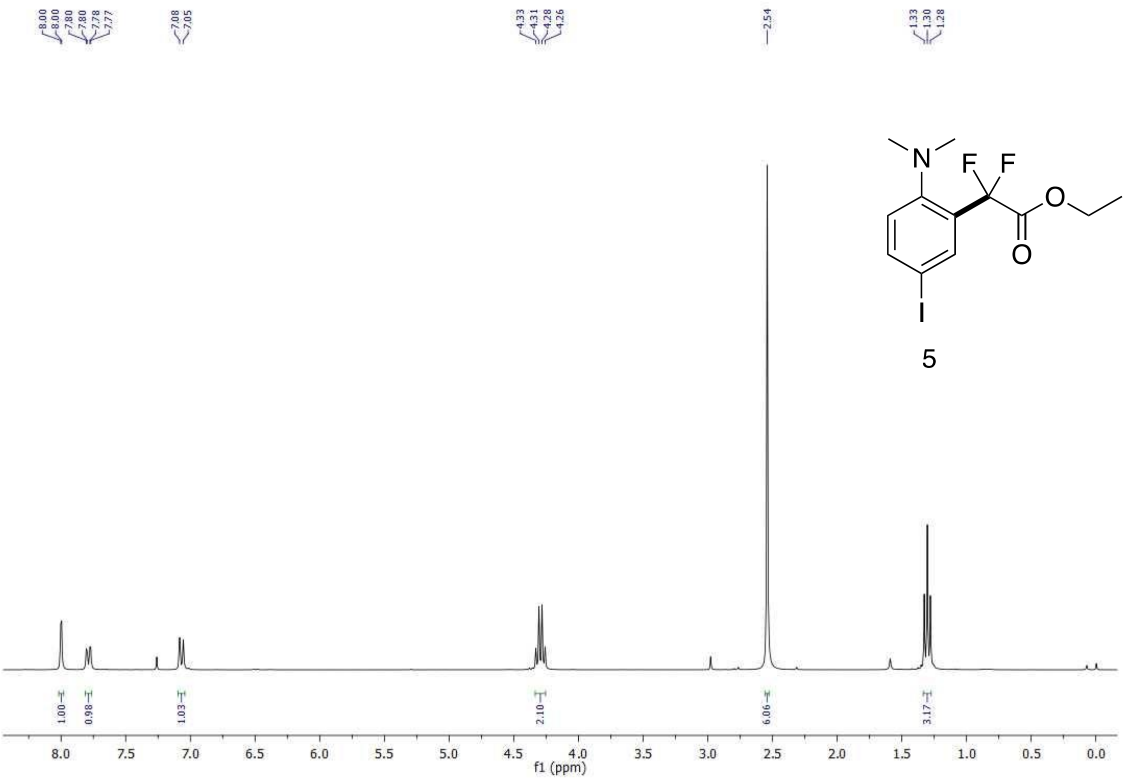

^{1}H NMR (300 MHz, $CDCl_3$) of compound **5**

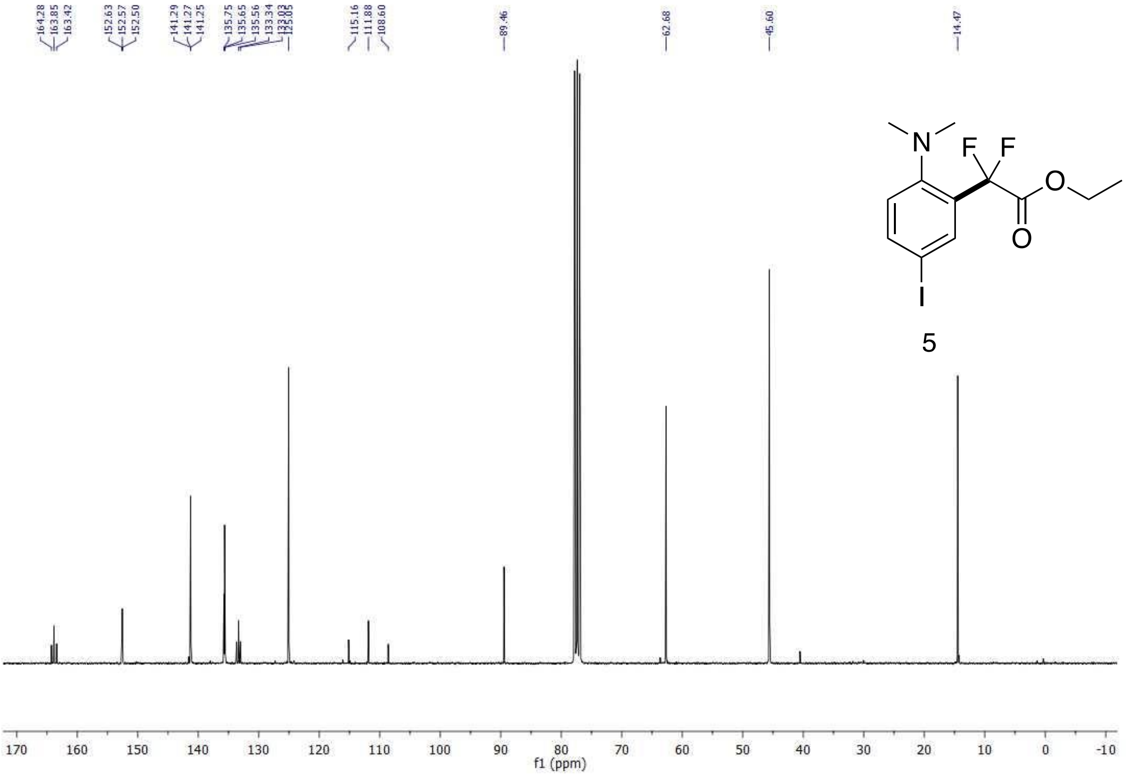

^{13}C{^{1}H} NMR (75 MHz, $CDCl_3$) of compound **5**

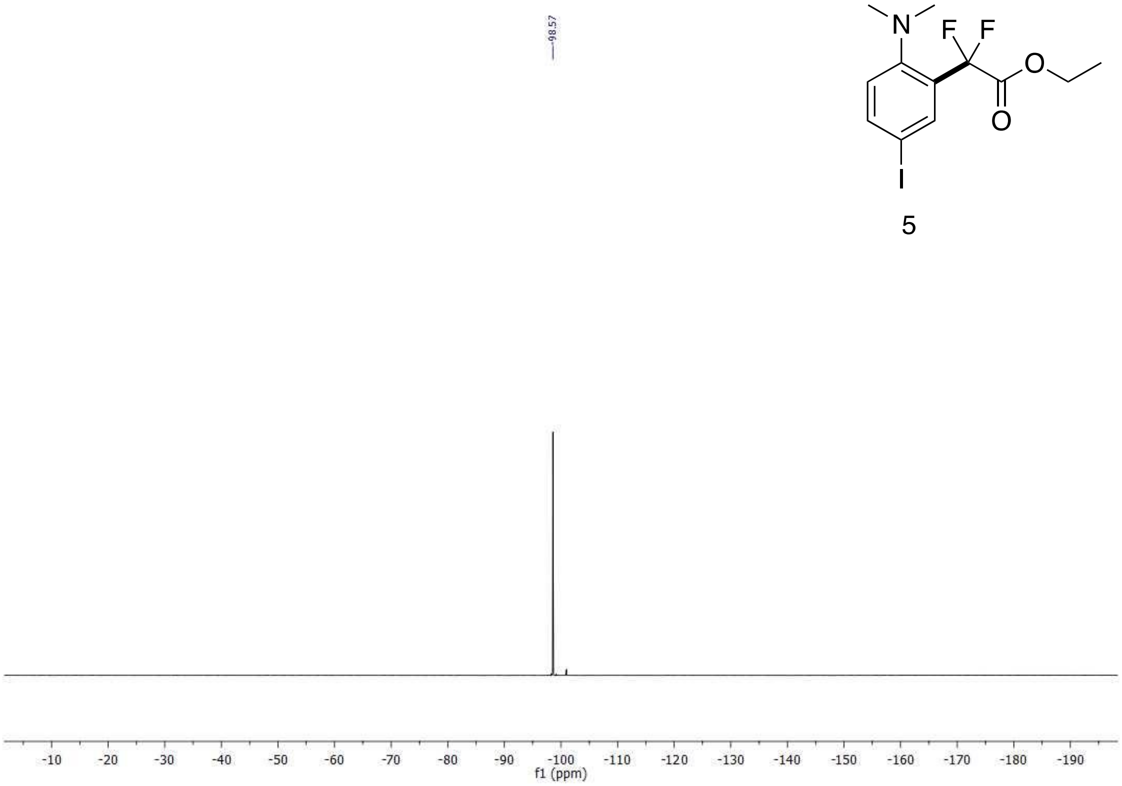

^{19}F NMR (282 MHz, $CDCl_3$) of compound **5**

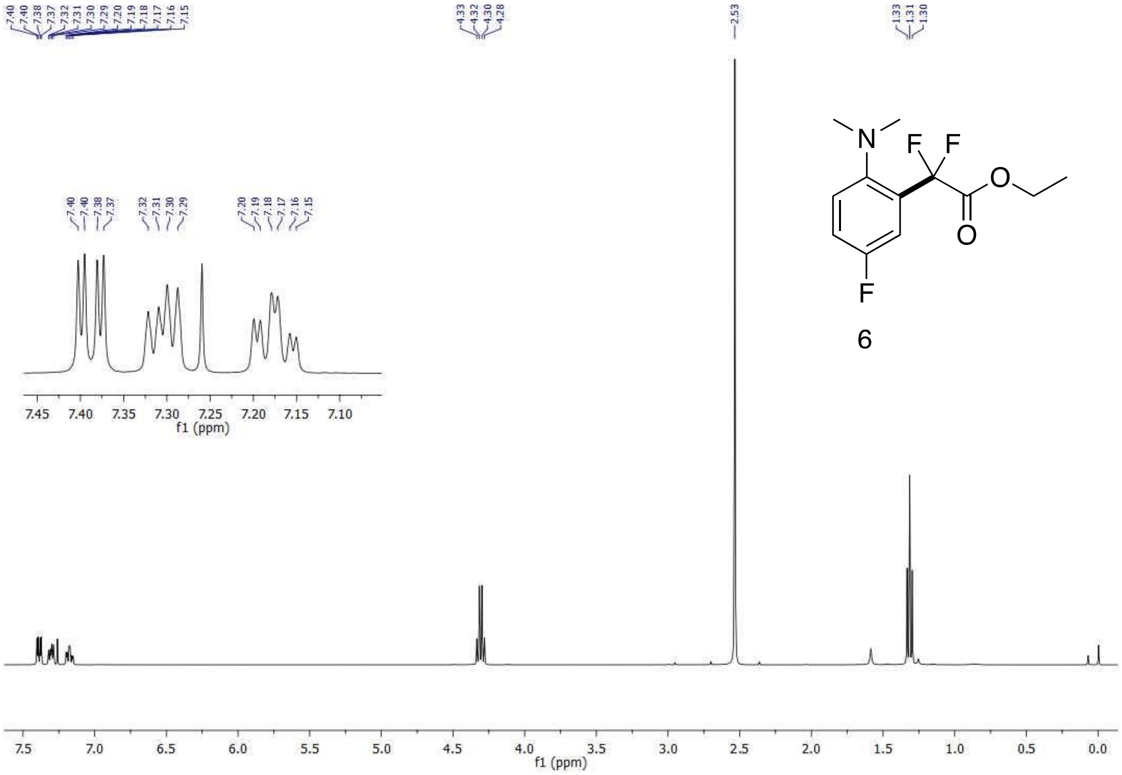

^{1}H NMR (400 MHz, $CDCl_3$) of compound **6**

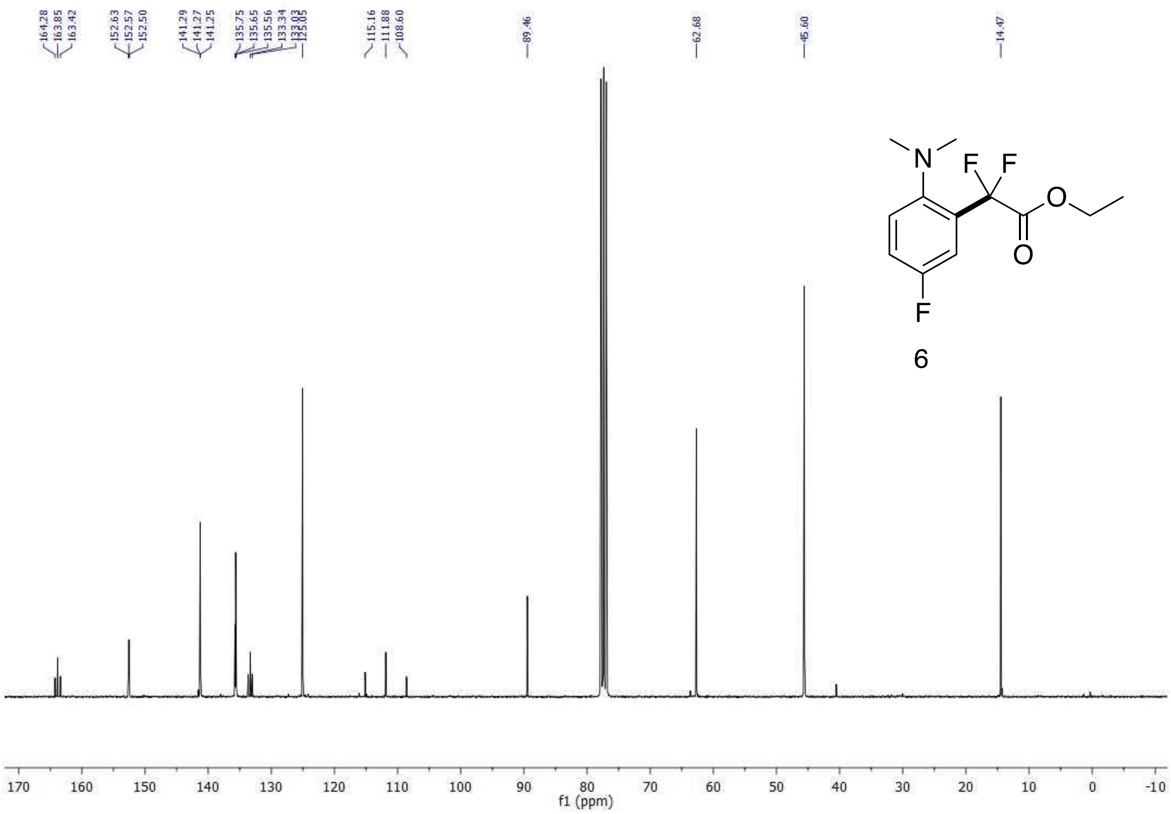

$^{13}C\{^{1}H\}$ NMR (100 MHz, $CDCl_3$) of compound **6**

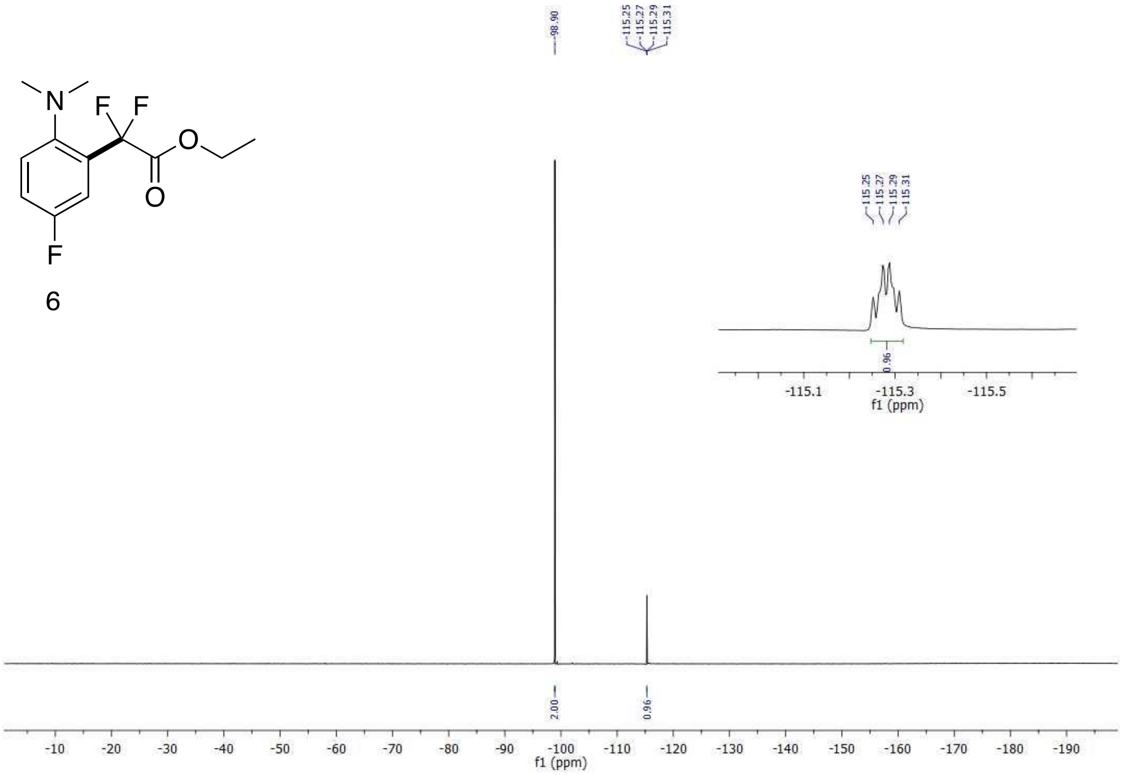

^{19}F NMR (282 MHz, $CDCl_3$) of compound **6**

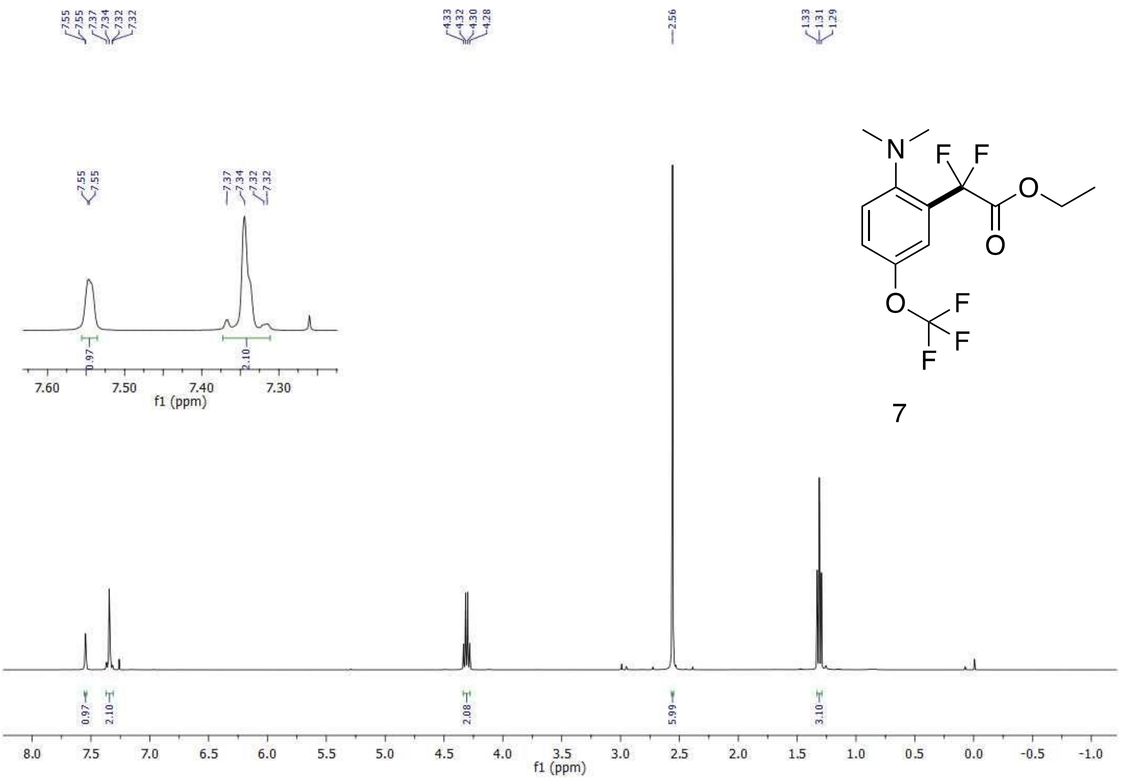

^{1}H NMR (400 MHz, $CDCl_3$) of compound **7**

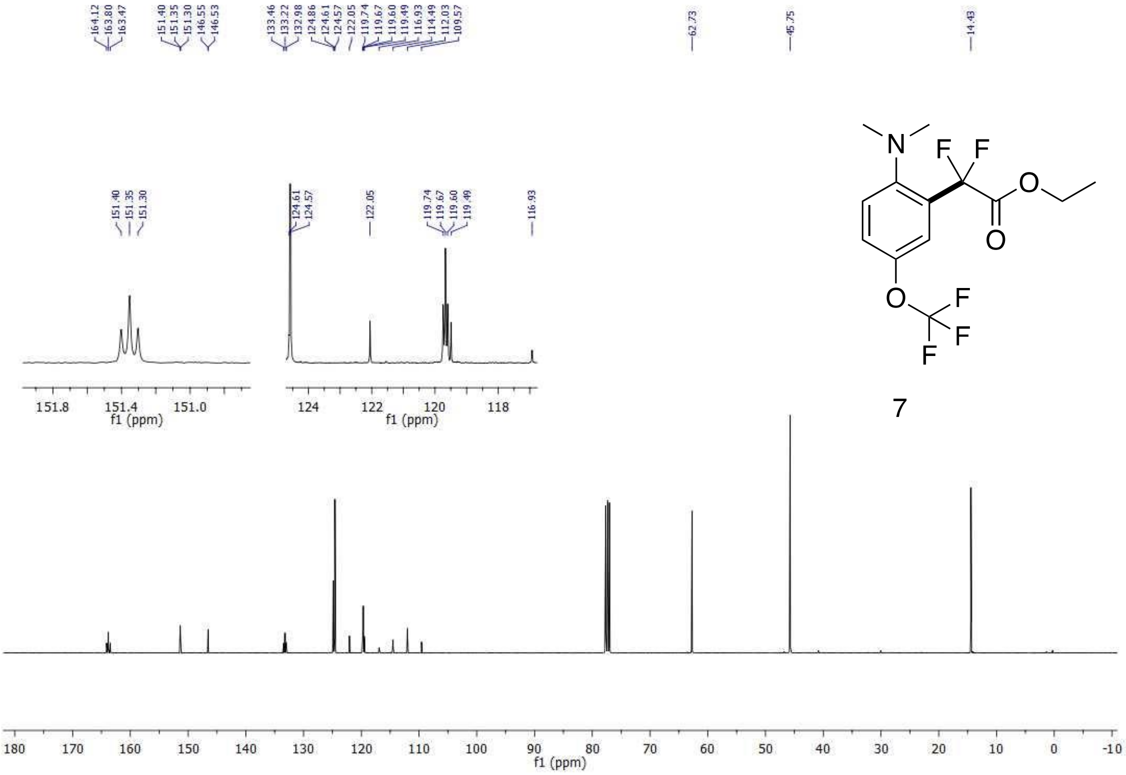

^{13}C{^{1}H} NMR (100 MHz, $CDCl_3$) of compound **7**

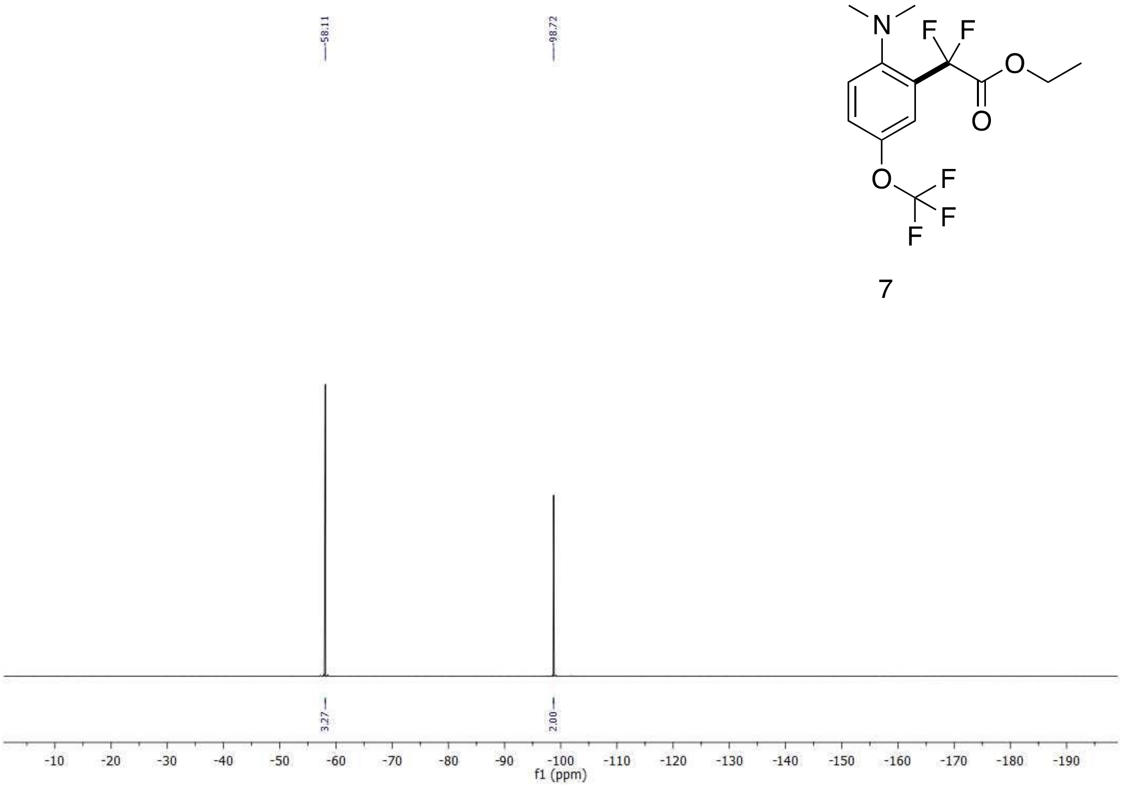

^{19}F NMR (376 MHz, $CDCl_3$) of compound **7**

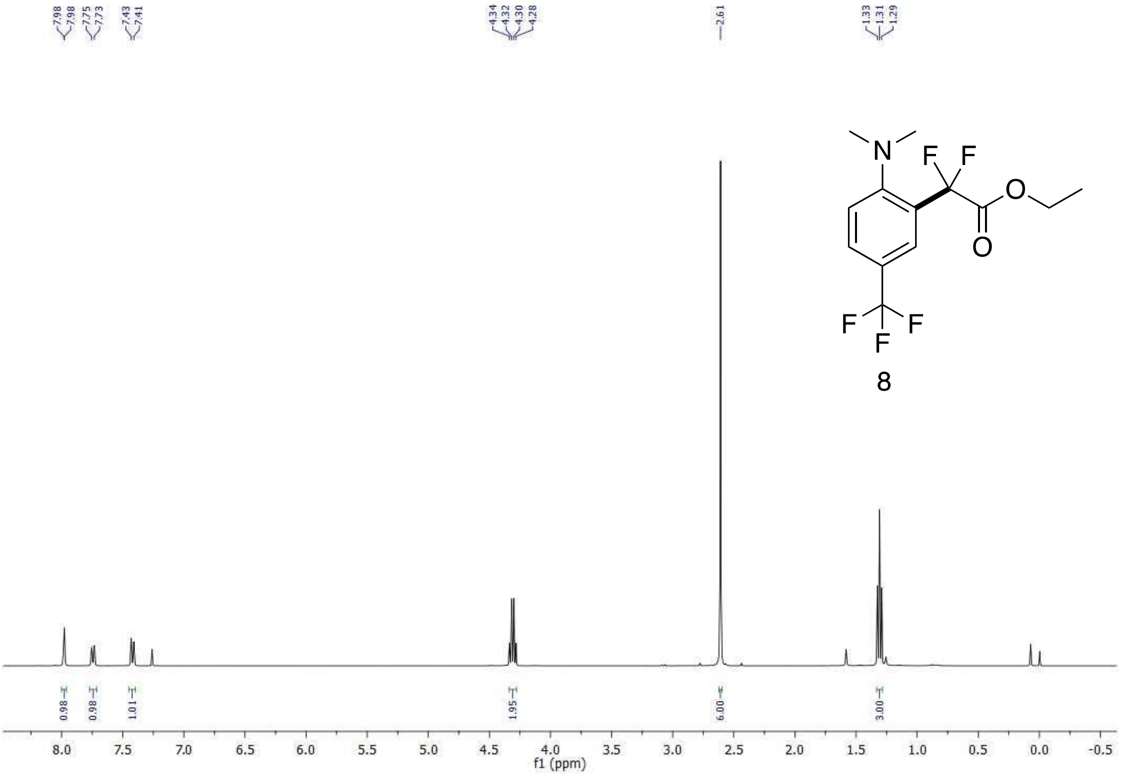

^{1}H NMR (400 MHz, $CDCl_3$) of compound **8**

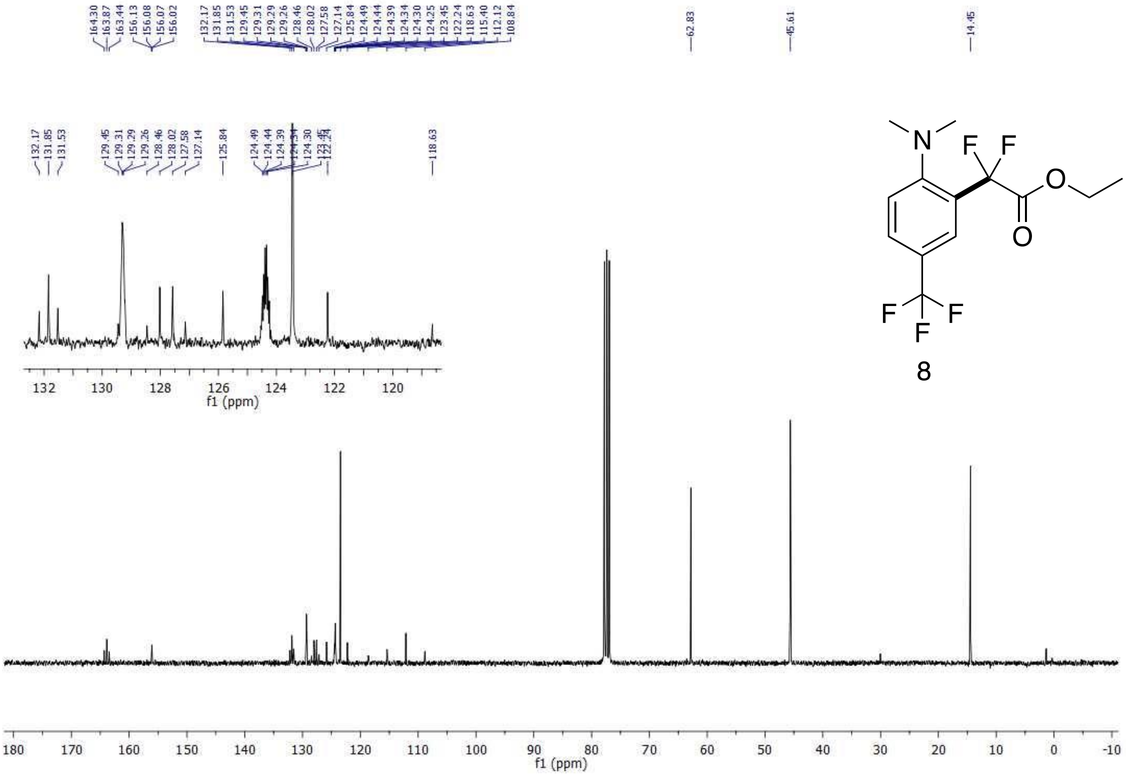

$^{13}C\{^{1}H\}$ NMR (75 MHz, $CDCl_3$) of compound **8**

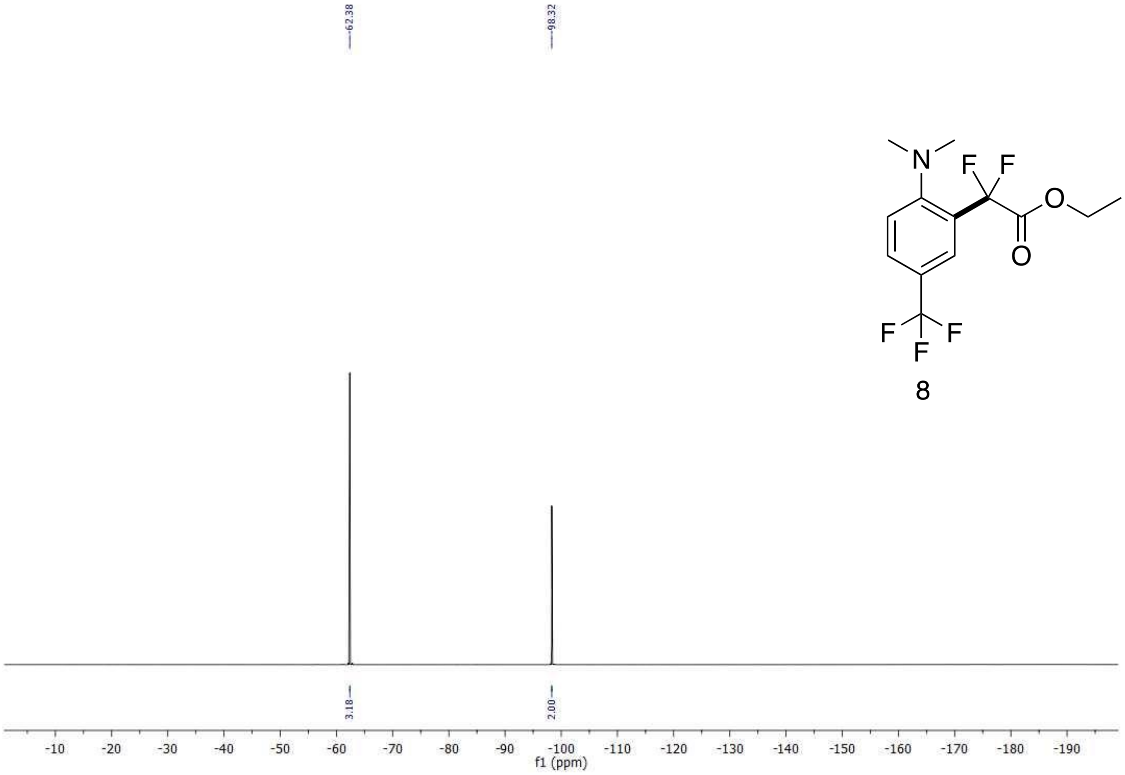

^{19}F NMR (282 MHz, $CDCl_3$) of compound **8**

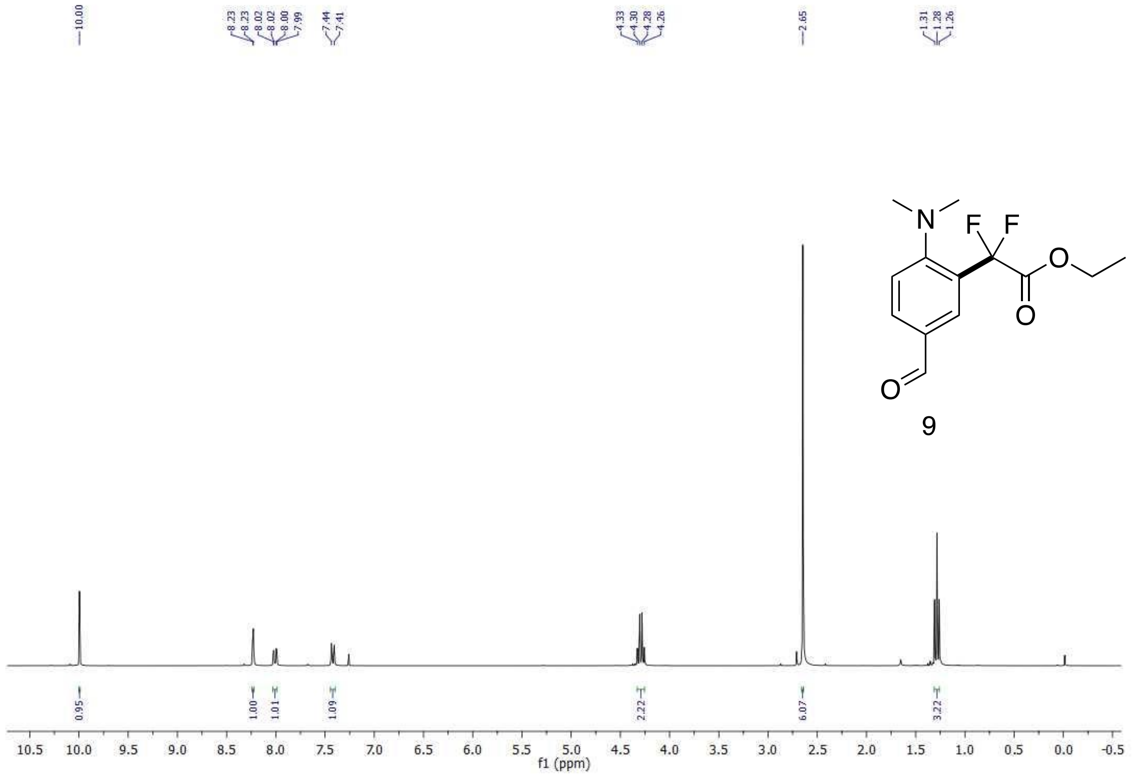

^{1}H NMR (300 MHz, $CDCl_3$) of compound **9**

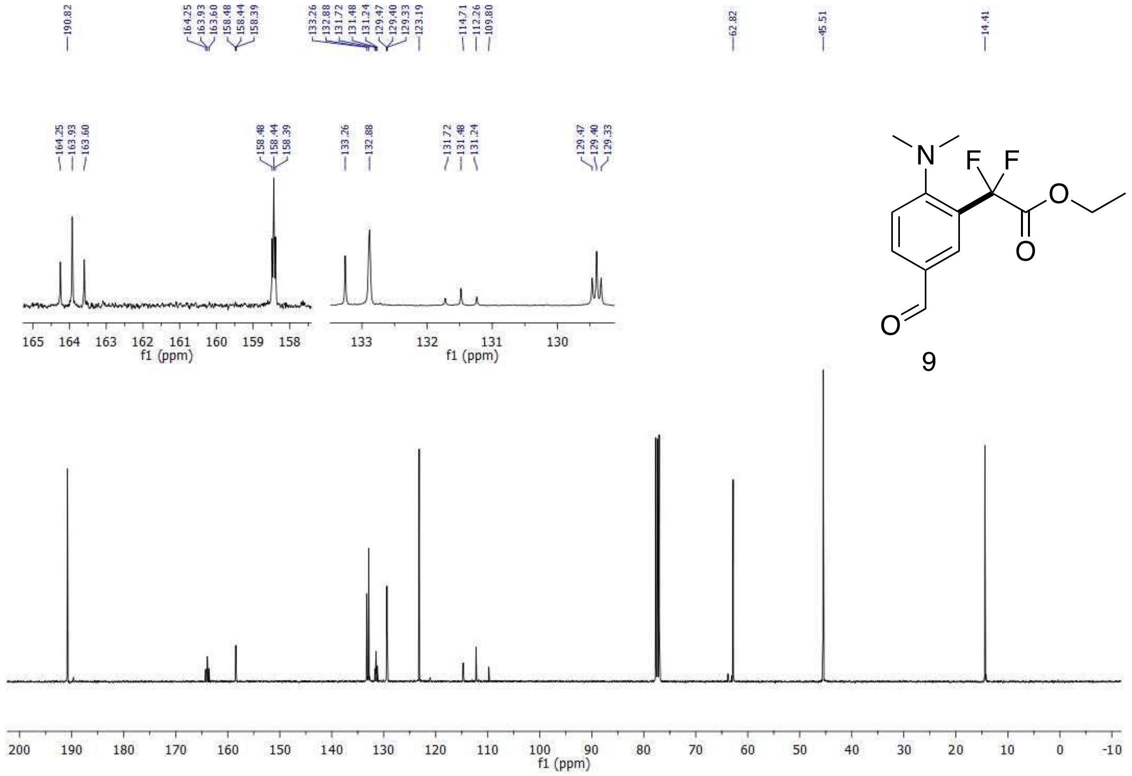

$^{13}C\{^{1}H\}$ NMR (100 MHz, $CDCl_3$) of compound **9**

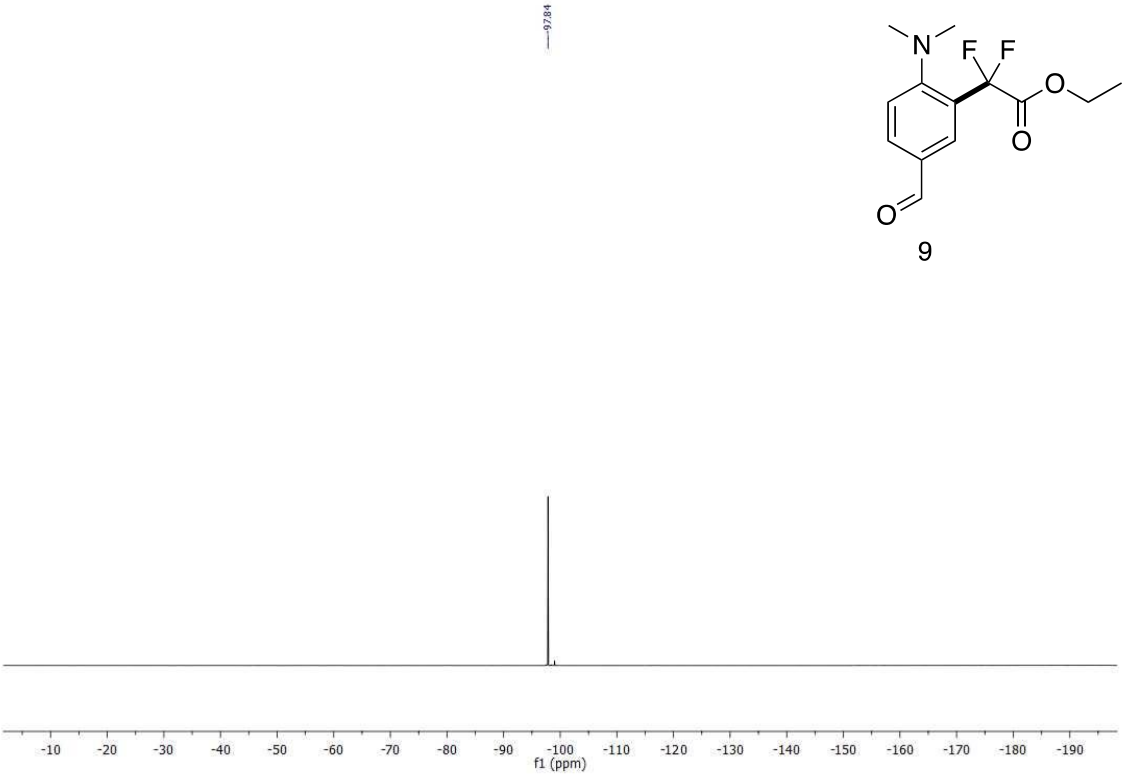

^{19}F NMR (282 MHz, $CDCl_3$) of compound **9**

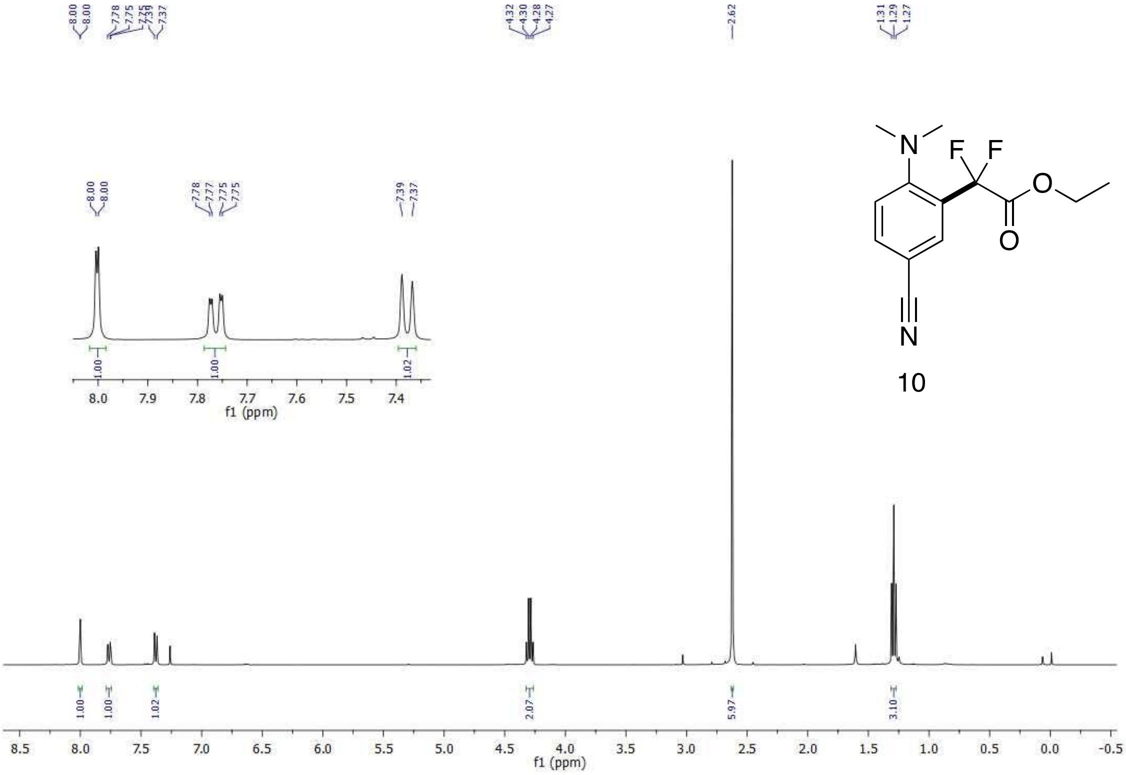

^{1}H NMR (400 MHz, $CDCl_3$) of compound **10**

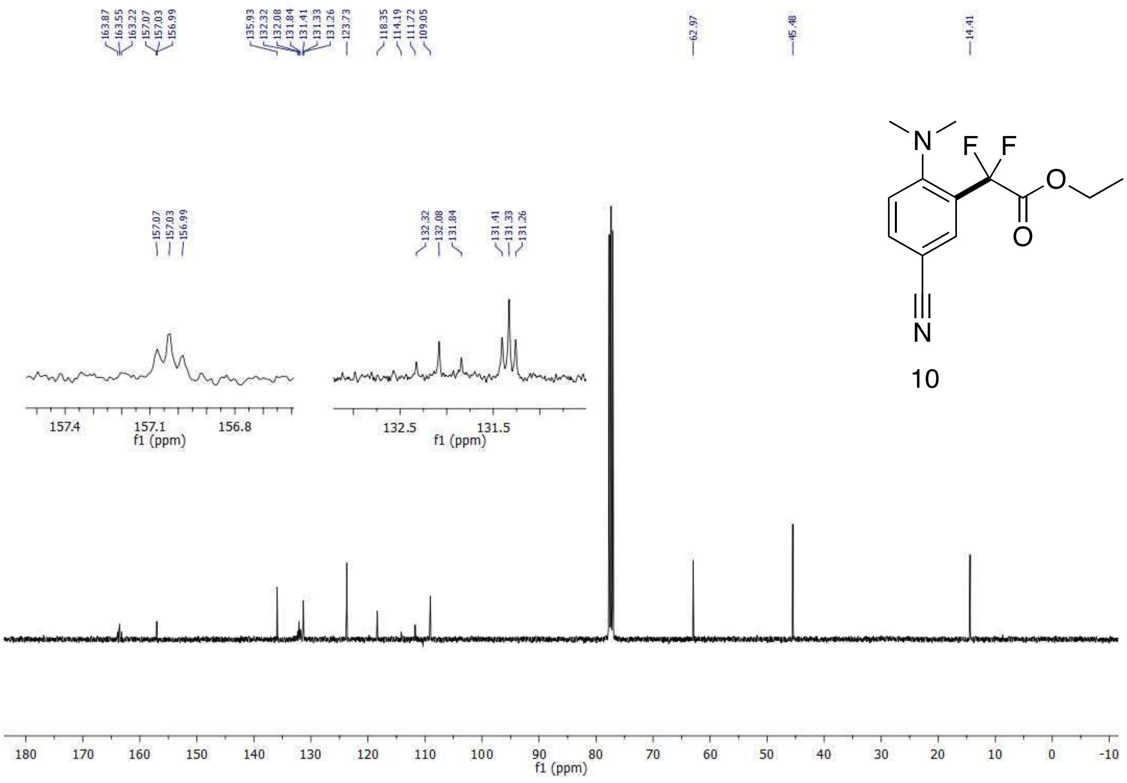

$^{13}C\{^{1}H\}$ NMR (100 MHz, $CDCl_3$) of compound **10**

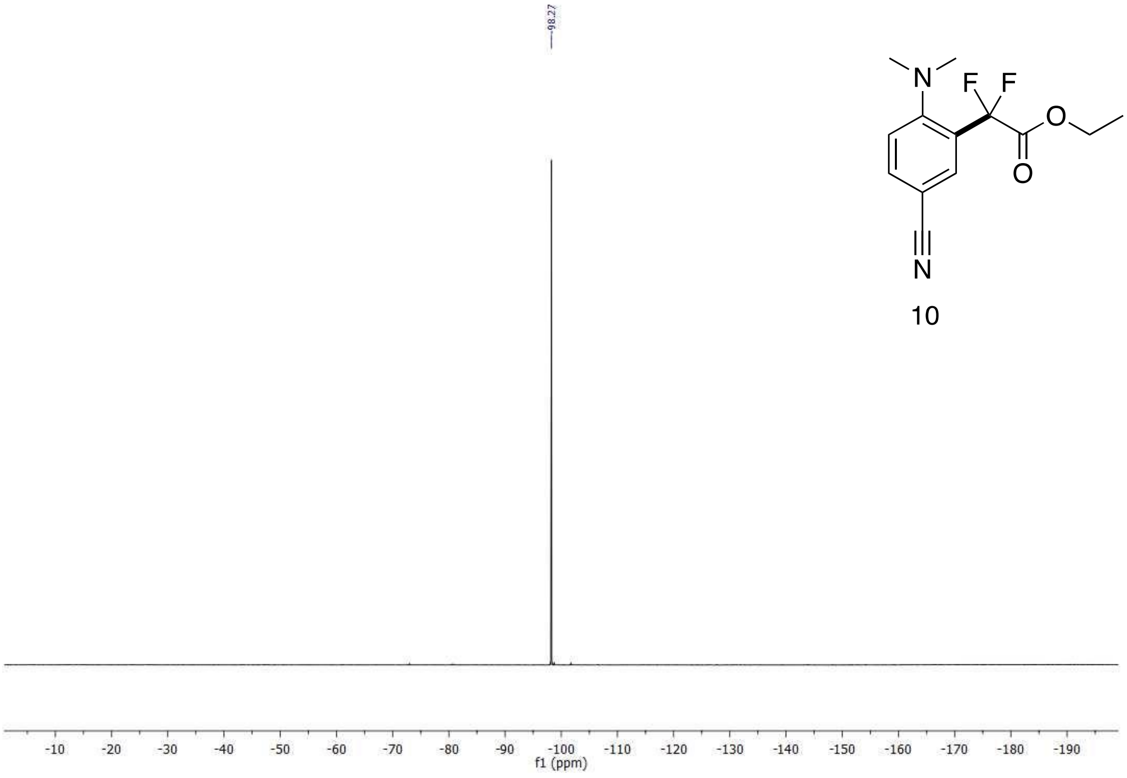

^{19}F NMR (282 MHz, $CDCl_3$) of compound **10**

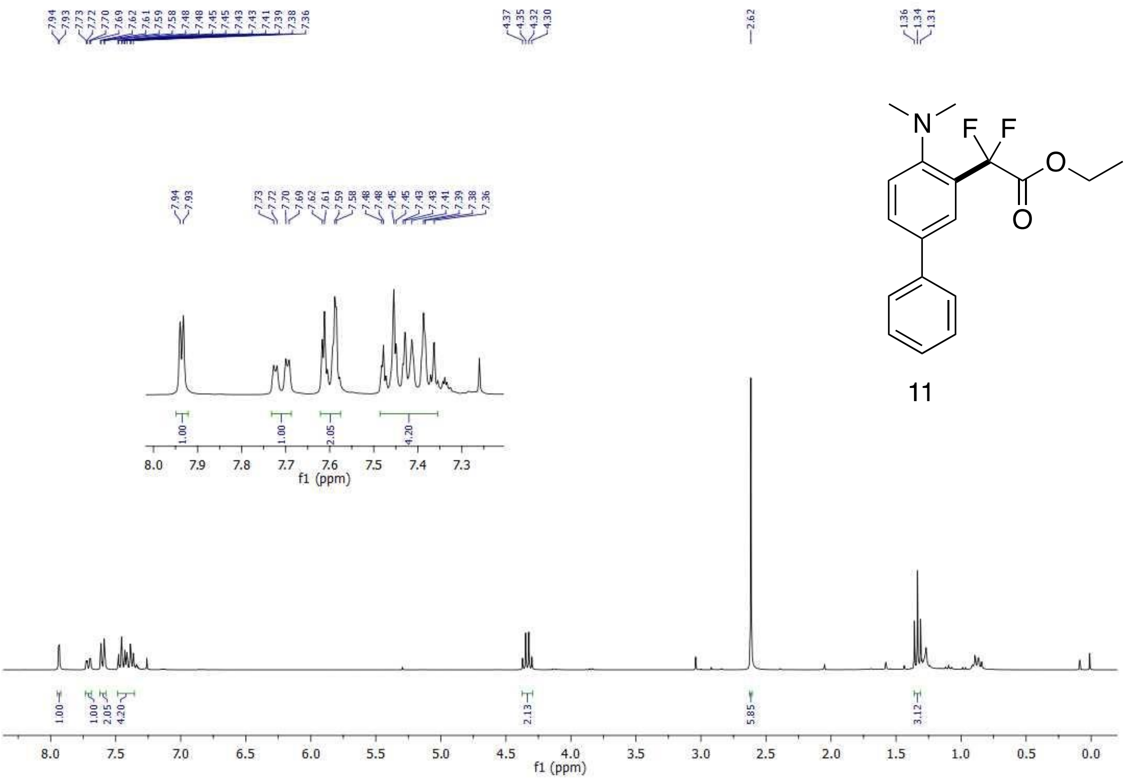

^{1}H NMR (300 MHz, $CDCl_3$) of compound **11**

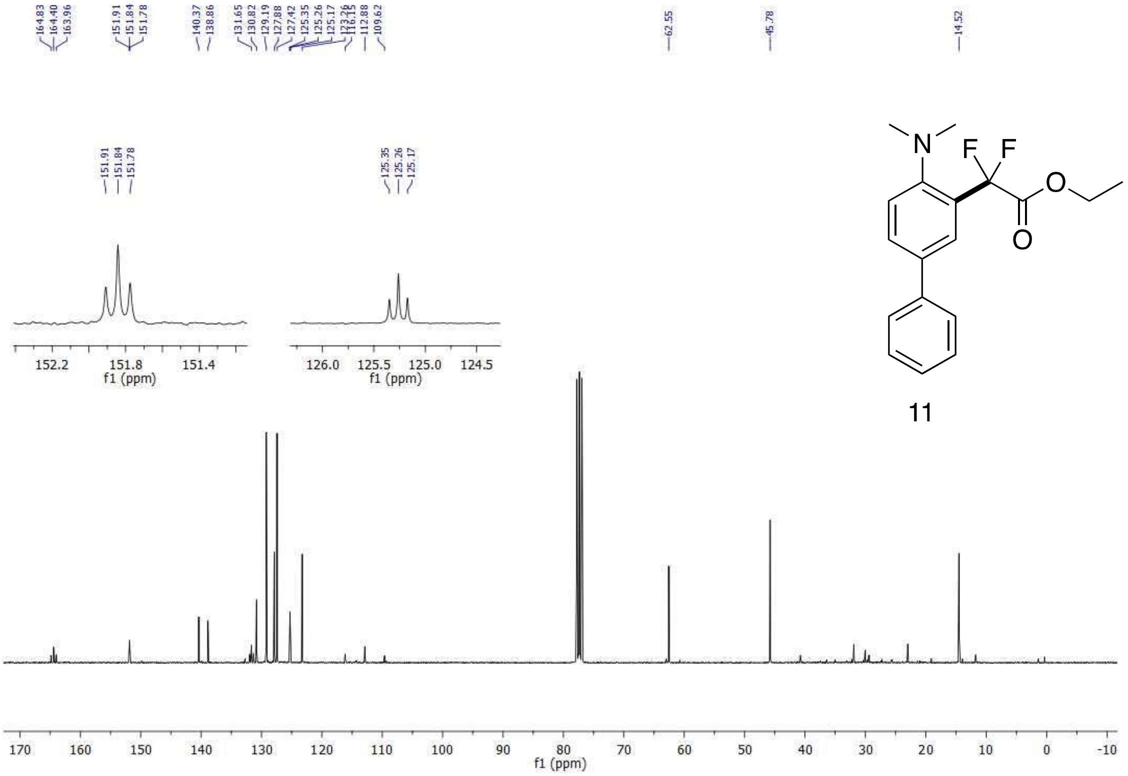

$^{13}C\{^{1}H\}$ NMR (75 MHz, $CDCl_3$) of compound **11**

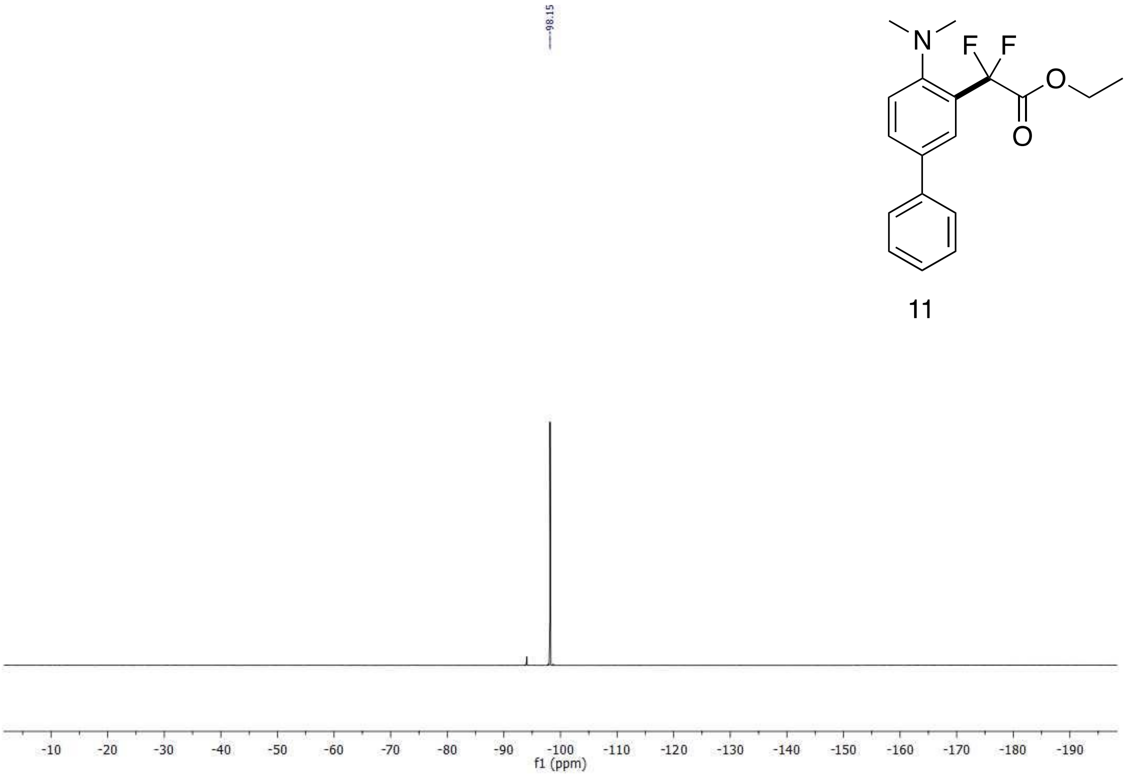

^{19}F NMR (235 MHz, $CDCl_3$) of compound **11**

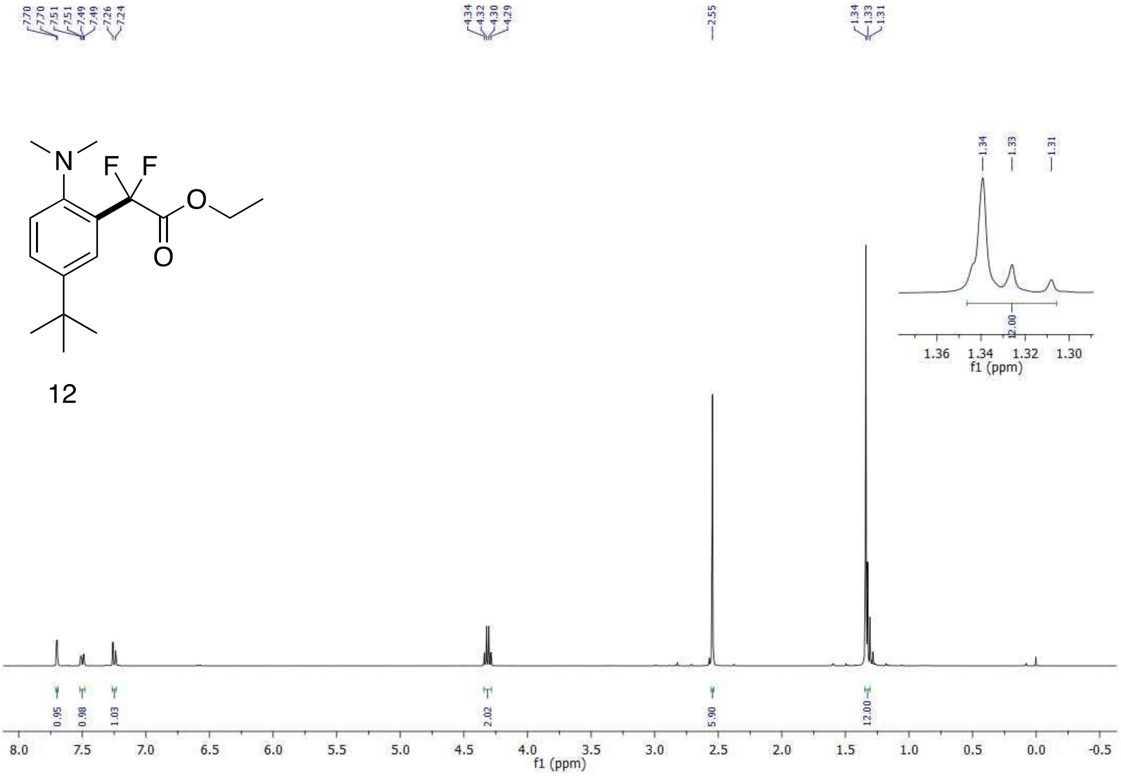

^{1}H NMR (400 MHz, $CDCl_3$) of compound **12**

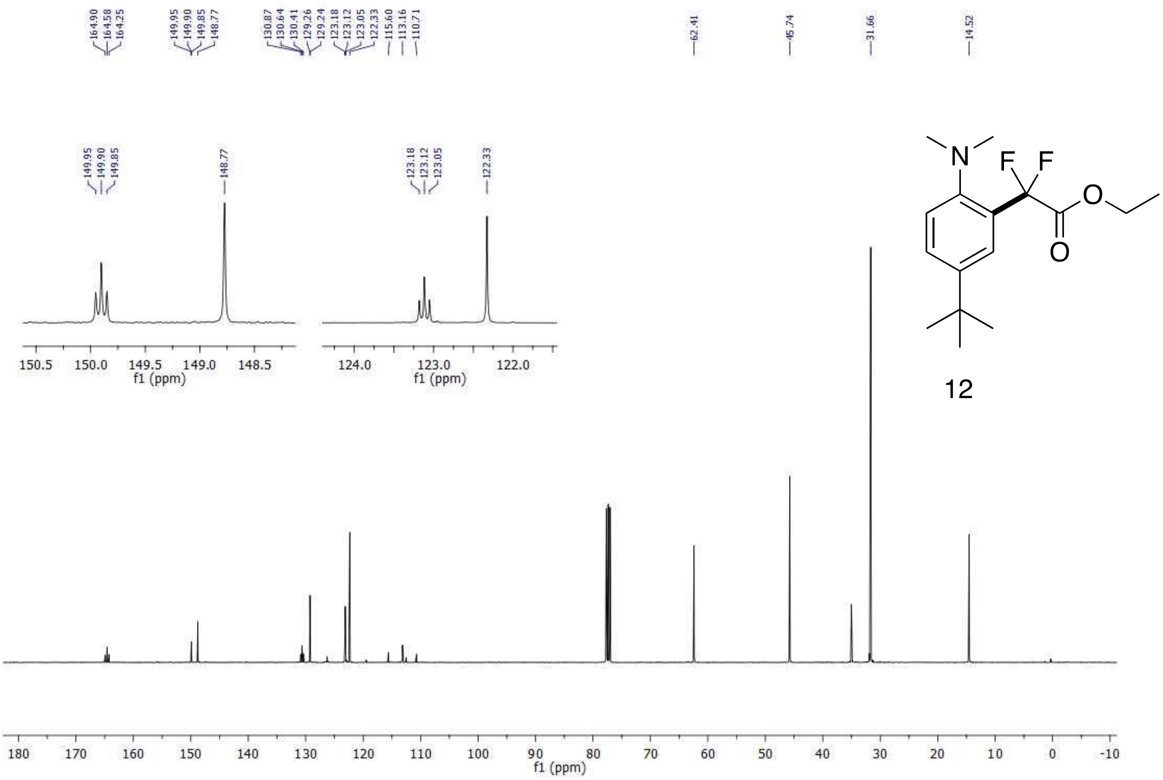

$^{13}C\{^{1}H\}$ NMR (100 MHz, $CDCl_3$) of compound **12**

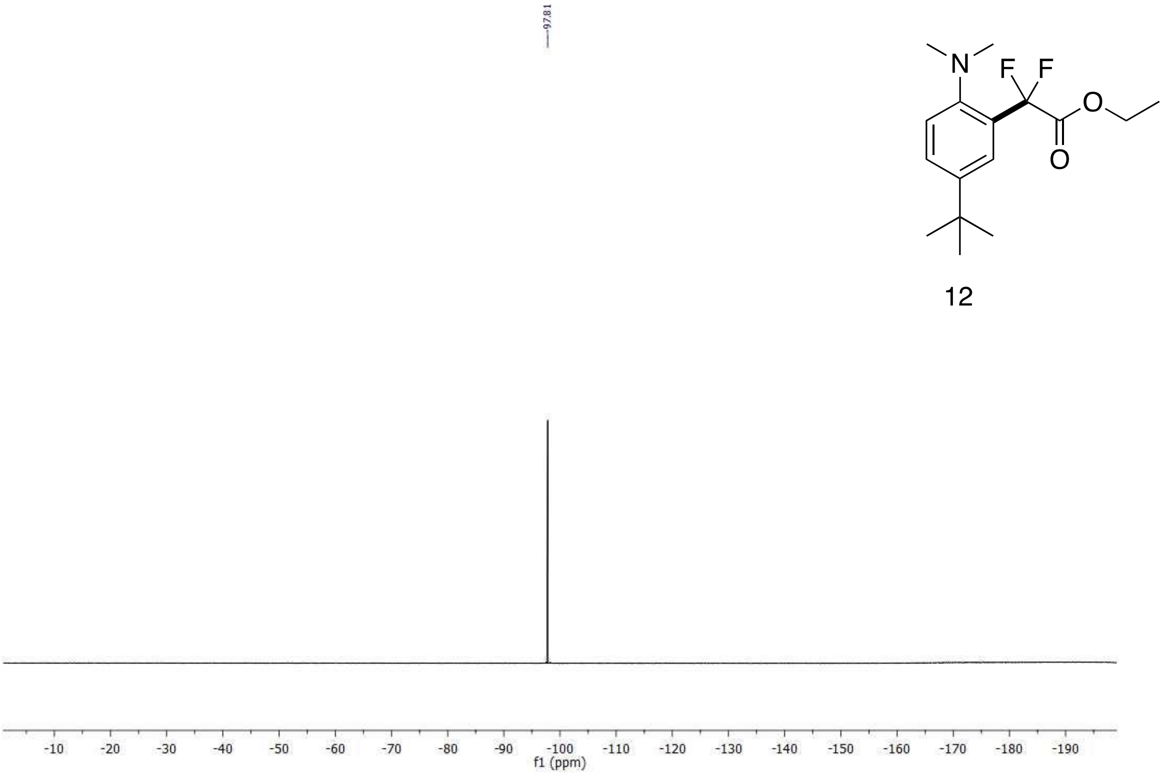

^{19}F NMR (376 MHz, $CDCl_3$) of compound **12**

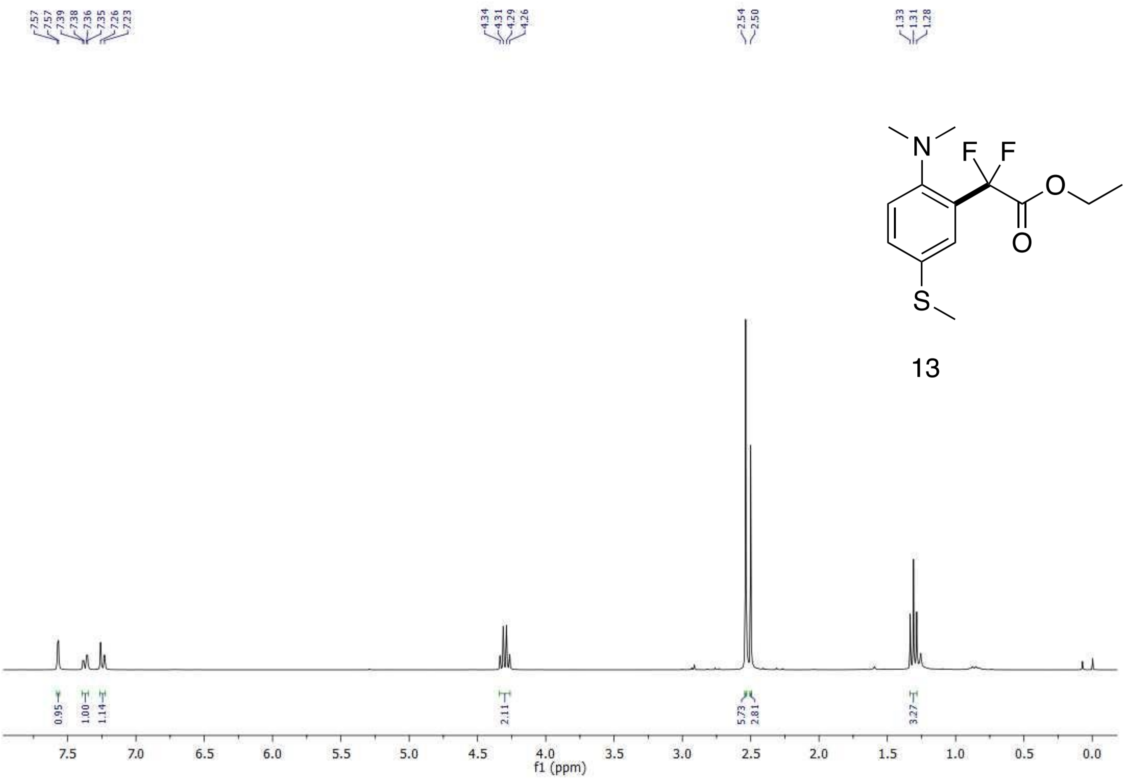

^{1}H NMR (300 MHz, $CDCl_3$) of compound **13**

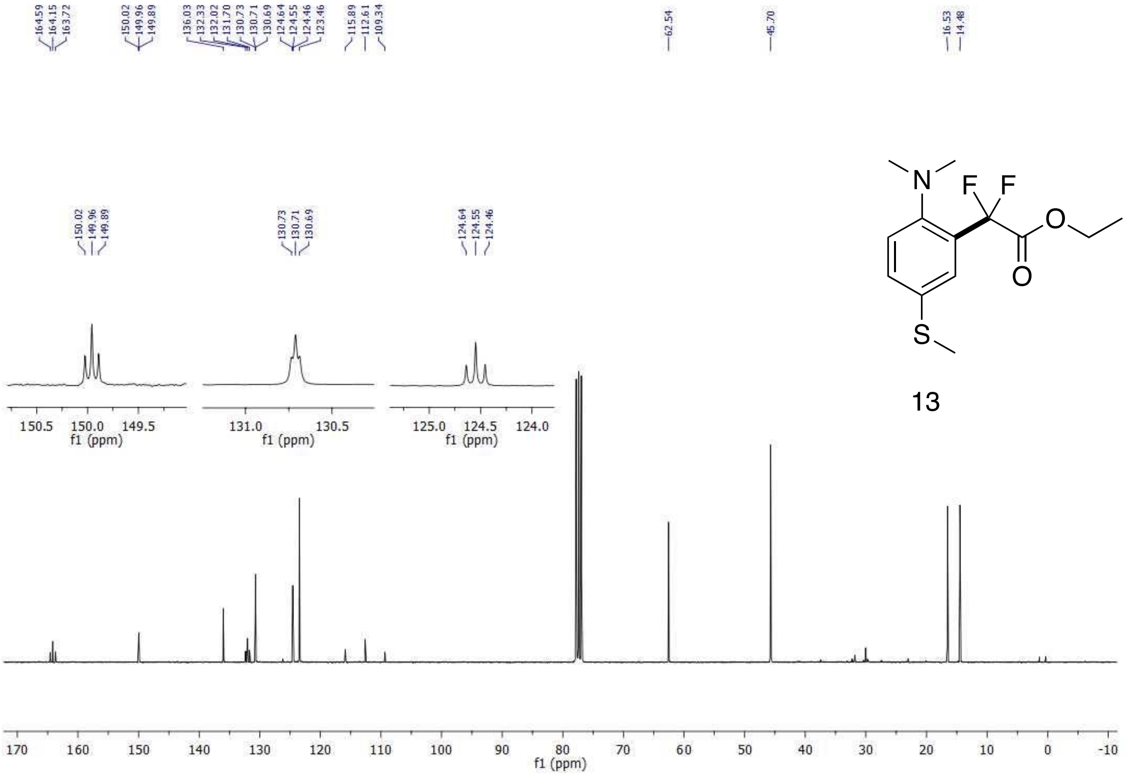

^{13}C{^{1}H} NMR (75 MHz, $CDCl_3$) of compound **13**

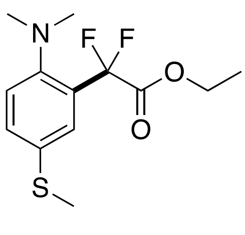

13

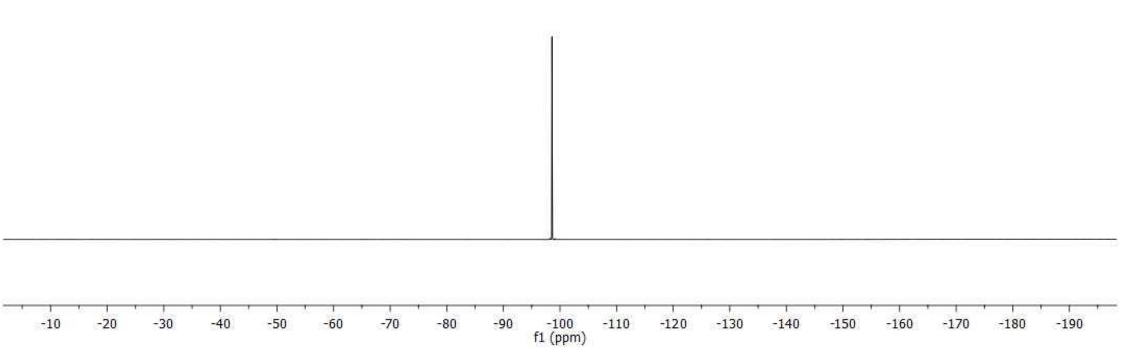

^{19}F NMR (235 MHz, $CDCl_3$) of compound **13**

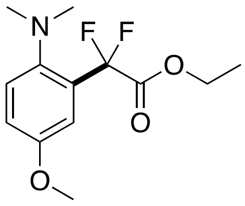

14

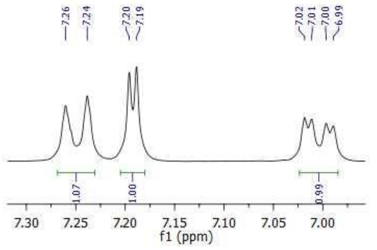

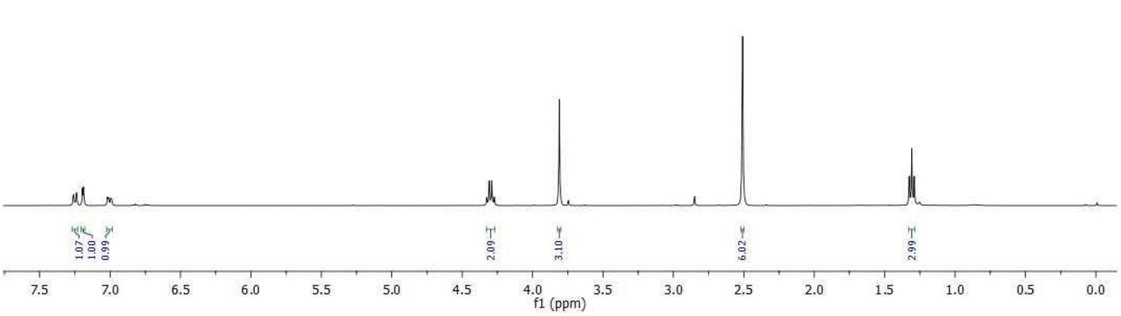

^{1}H NMR (300 MHz, $CDCl_3$) of compound **14**

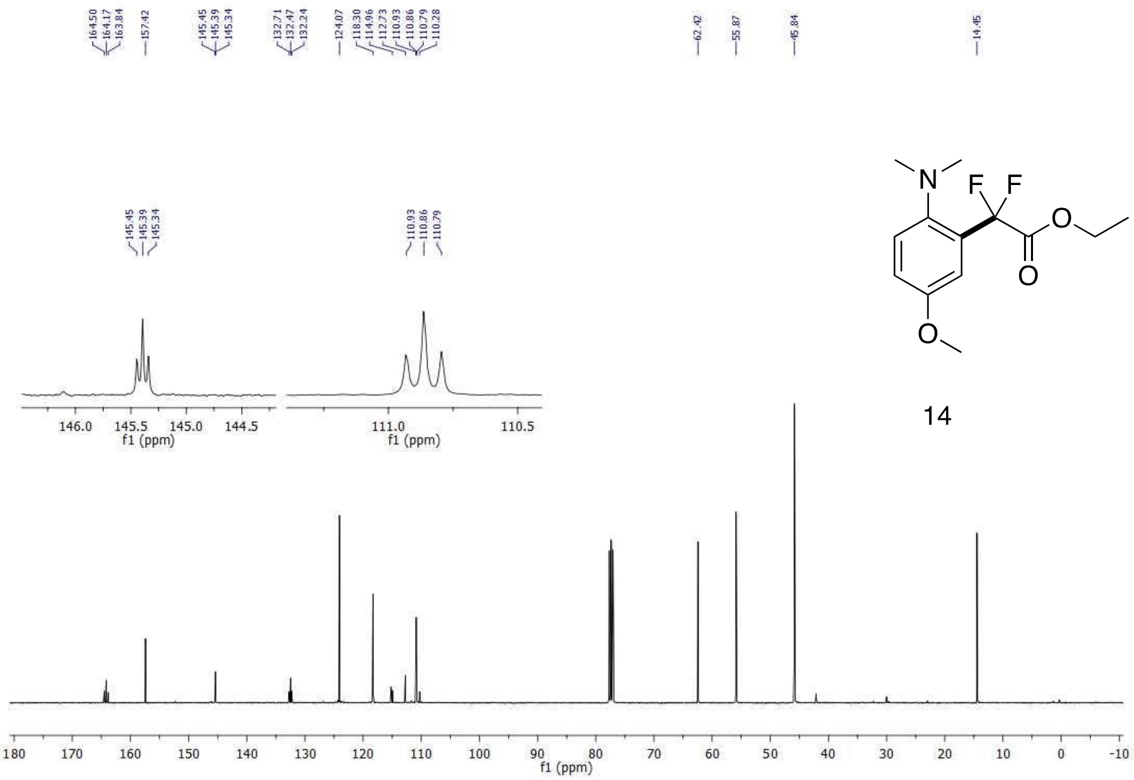

$^{13}C\{^{1}H\}$ NMR (100 MHz, $CDCl_3$) of compound **14**

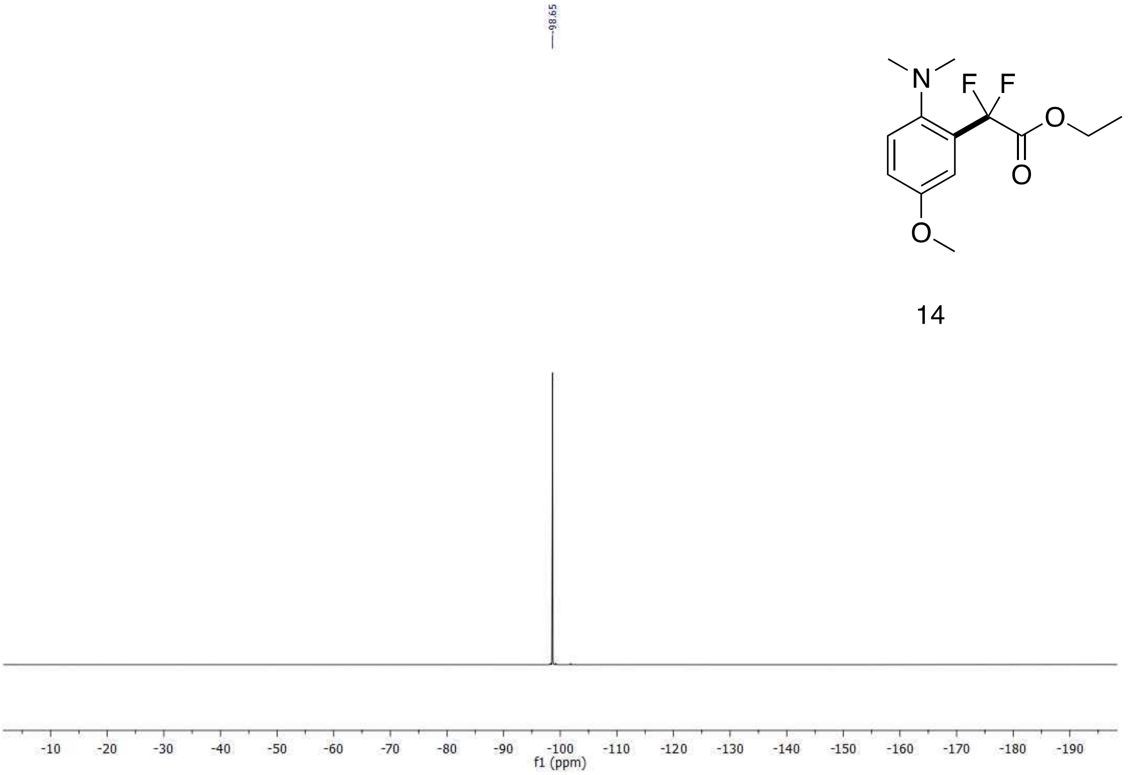

^{19}F NMR (282 MHz, $CDCl_3$) of compound **14**

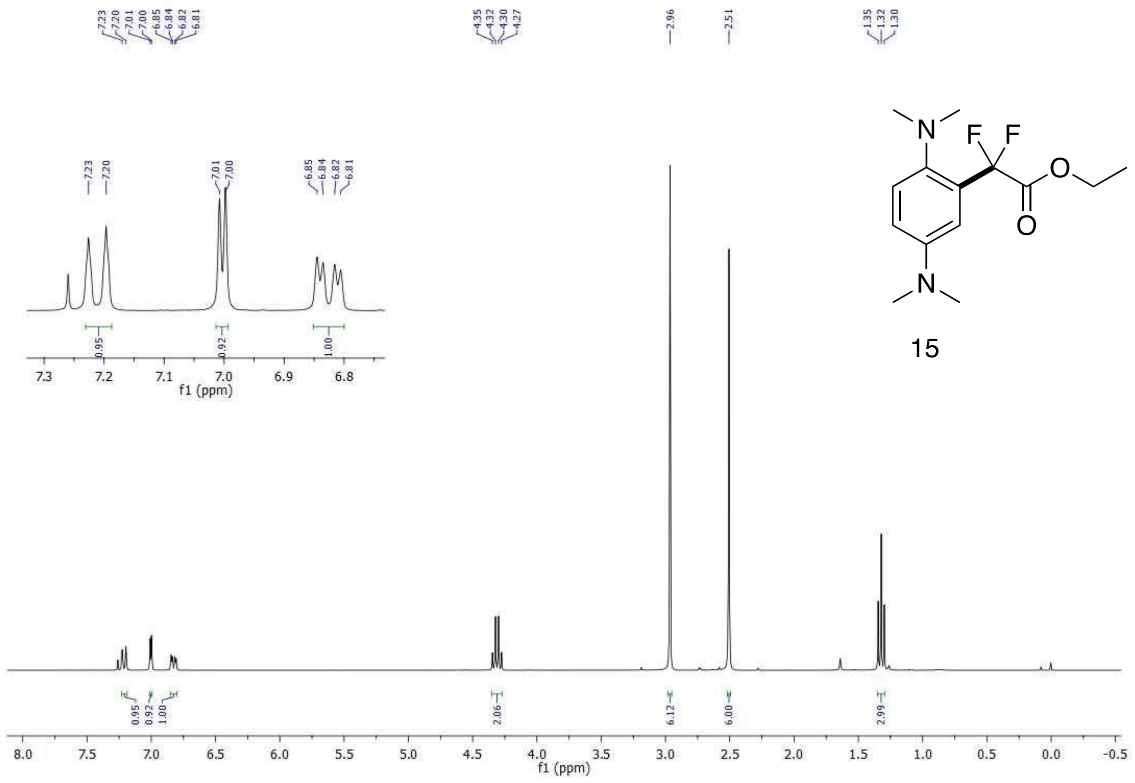

^{1}H NMR (400 MHz, $CDCl_3$) of compound **15**

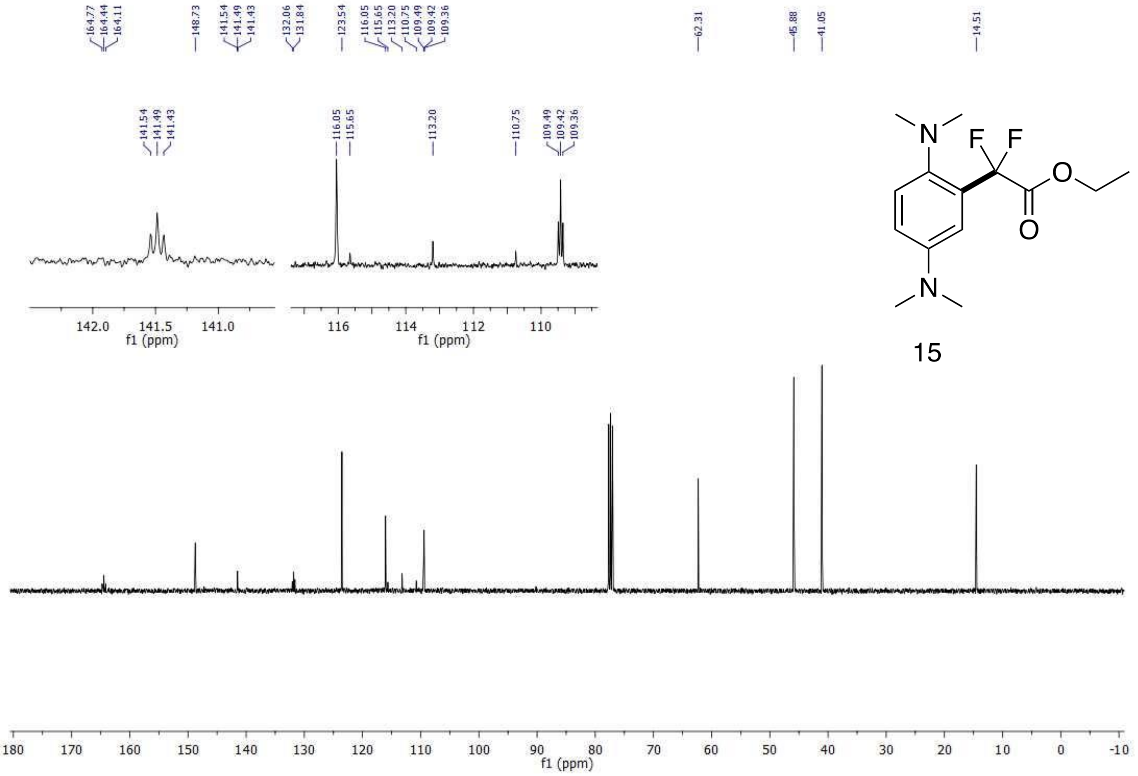

$^{13}C\{^{1}H\}$ NMR (100 MHz, $CDCl_3$) of compound **15**

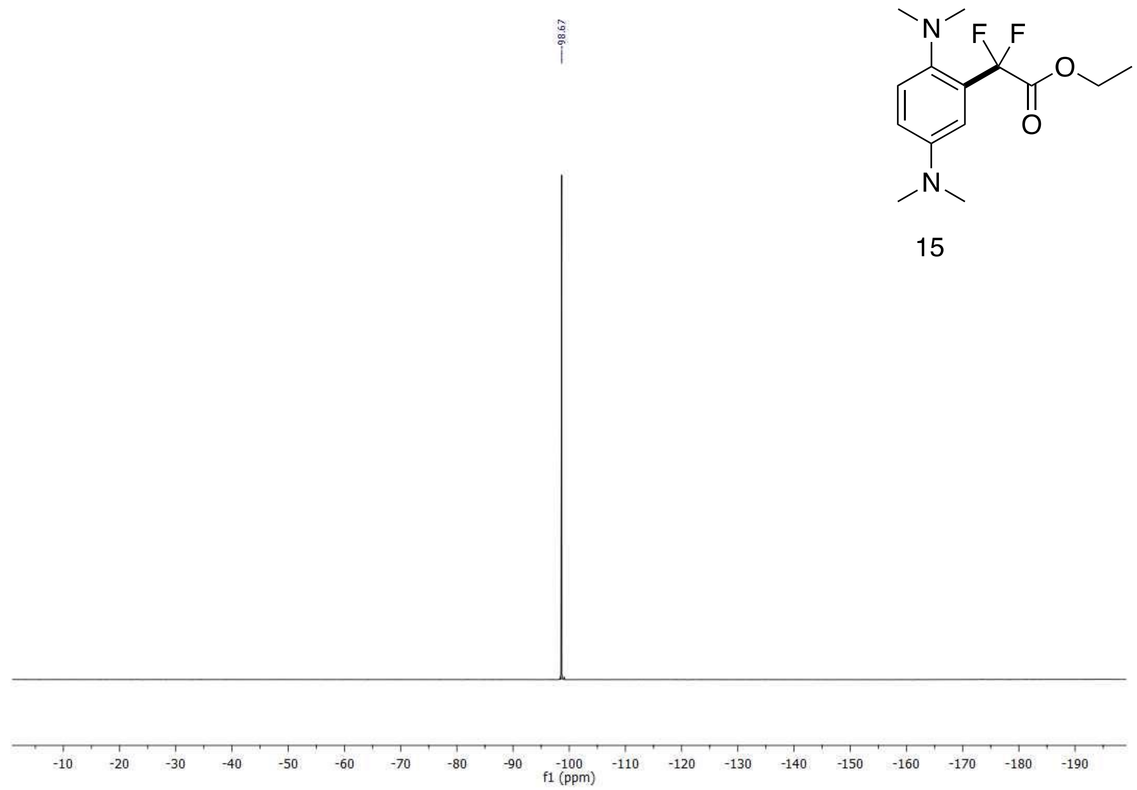

^{19}F NMR (376 MHz, $CDCl_3$) of compound **15**

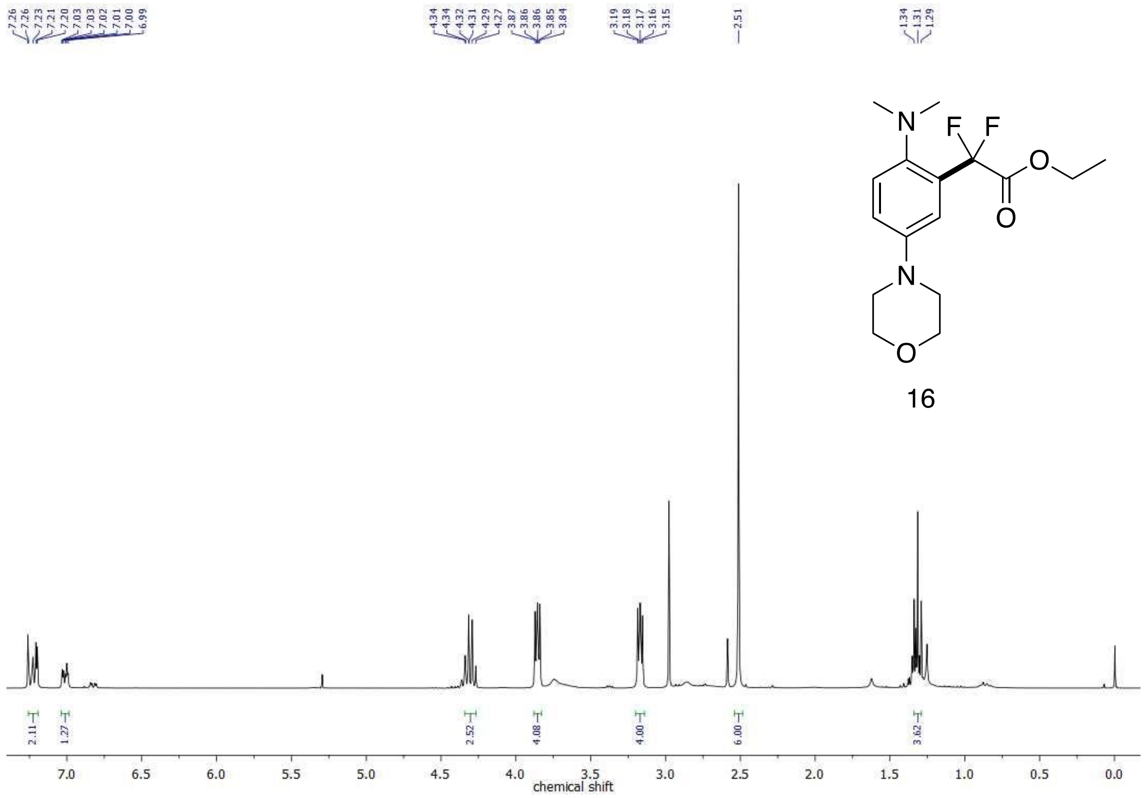

^{1}H NMR (300 MHz, $CDCl_3$) of compound **16**

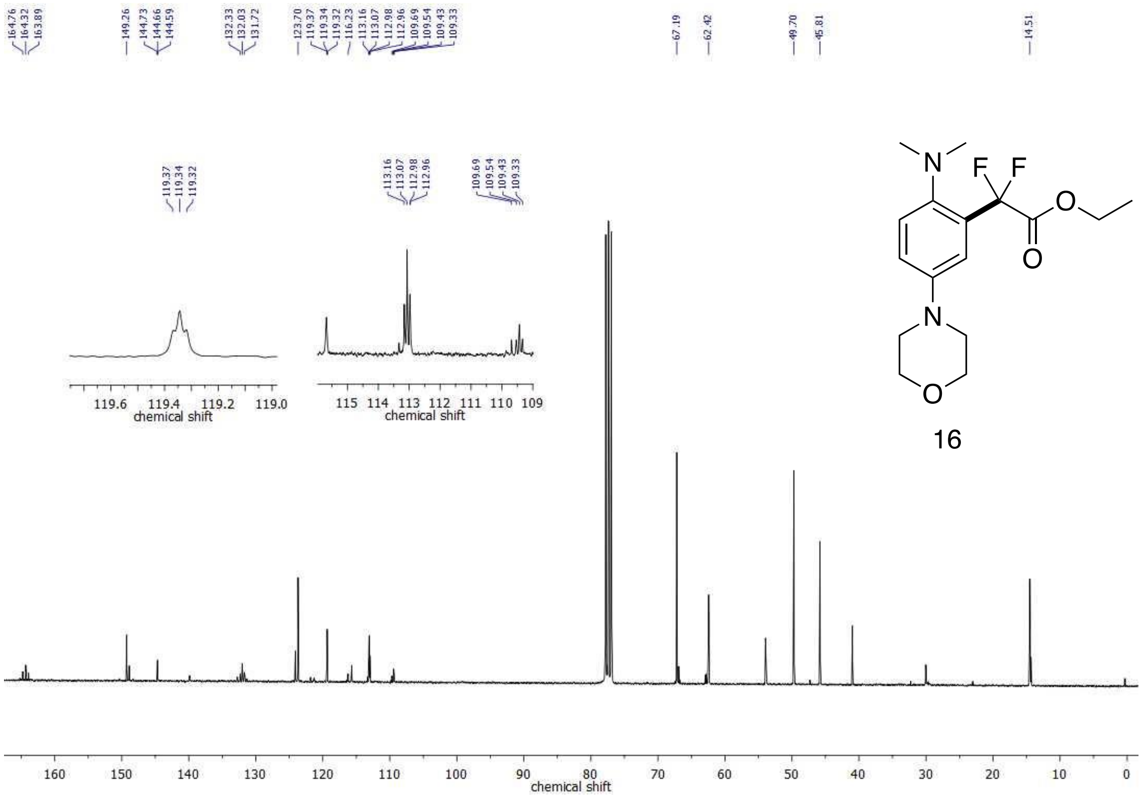

$^{13}C\{^{1}H\}$ NMR (75 MHz, $CDCl_3$) of compound **16**

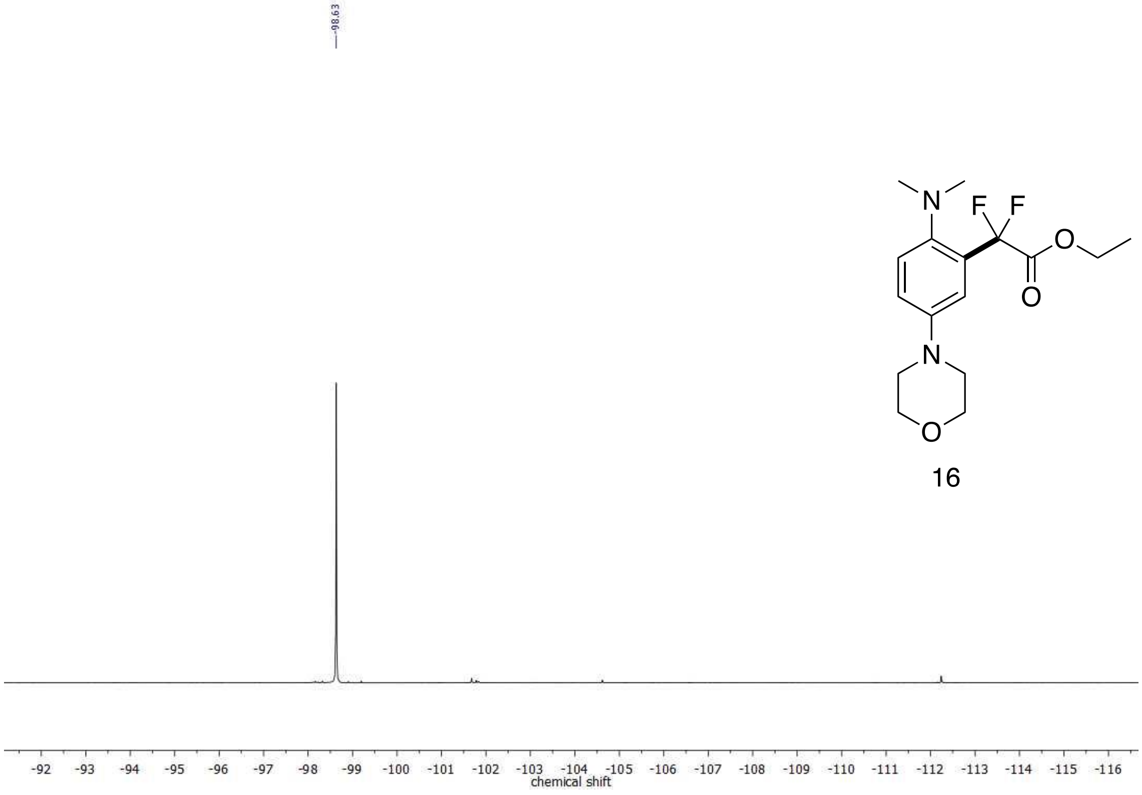

^{19}F NMR (235 MHz, $CDCl_3$) of compound **16**

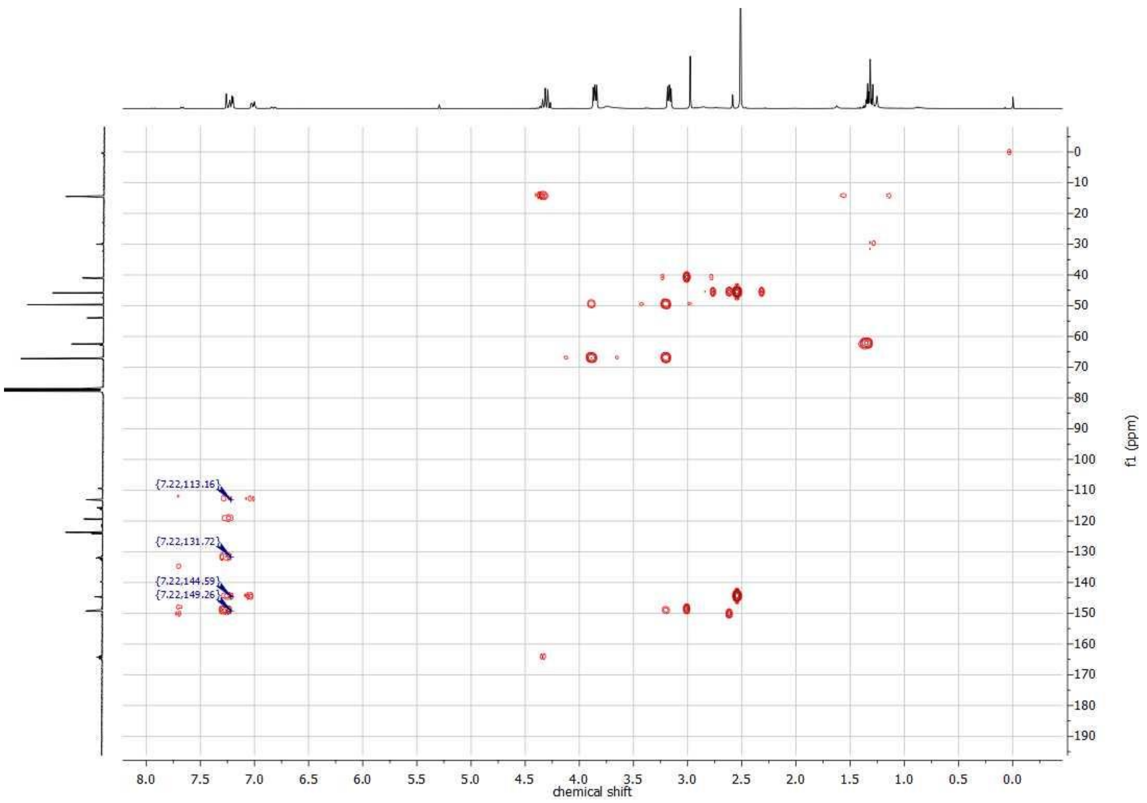

HMBC (75 MHz, $CDCl_3$) of compound **16**

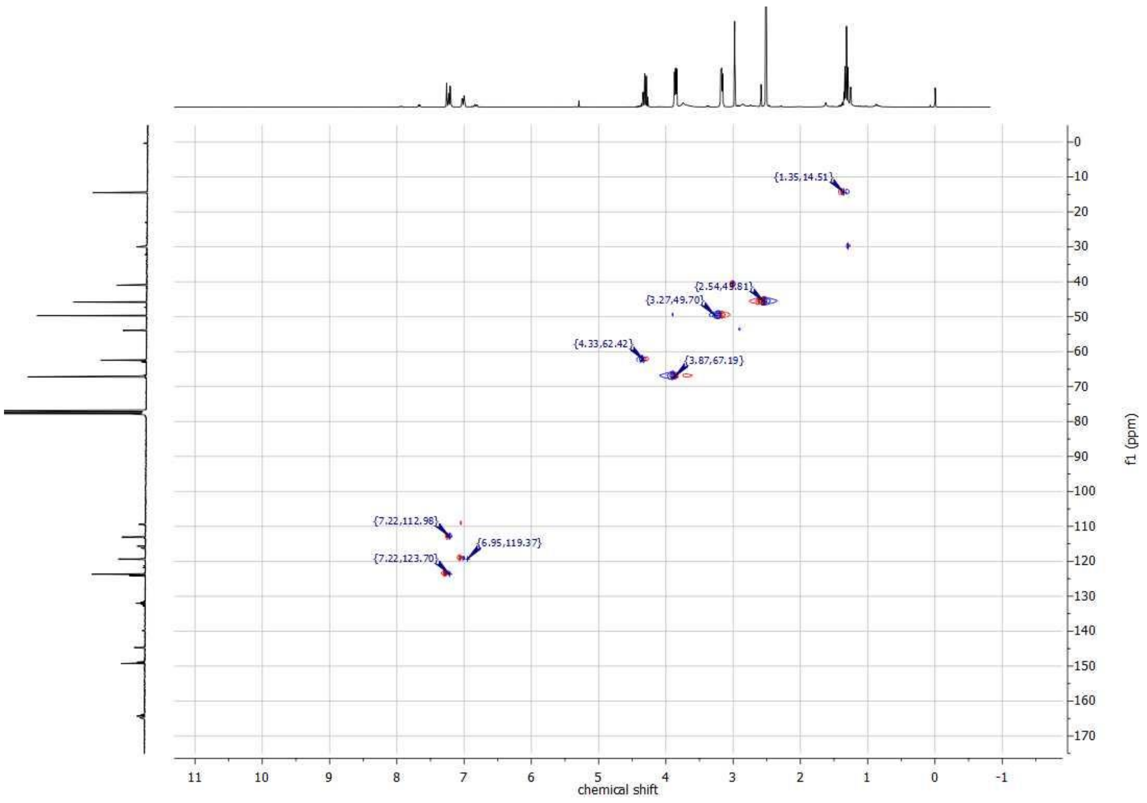

HSQCed (75 MHz, $CDCl_3$) of compound **16**

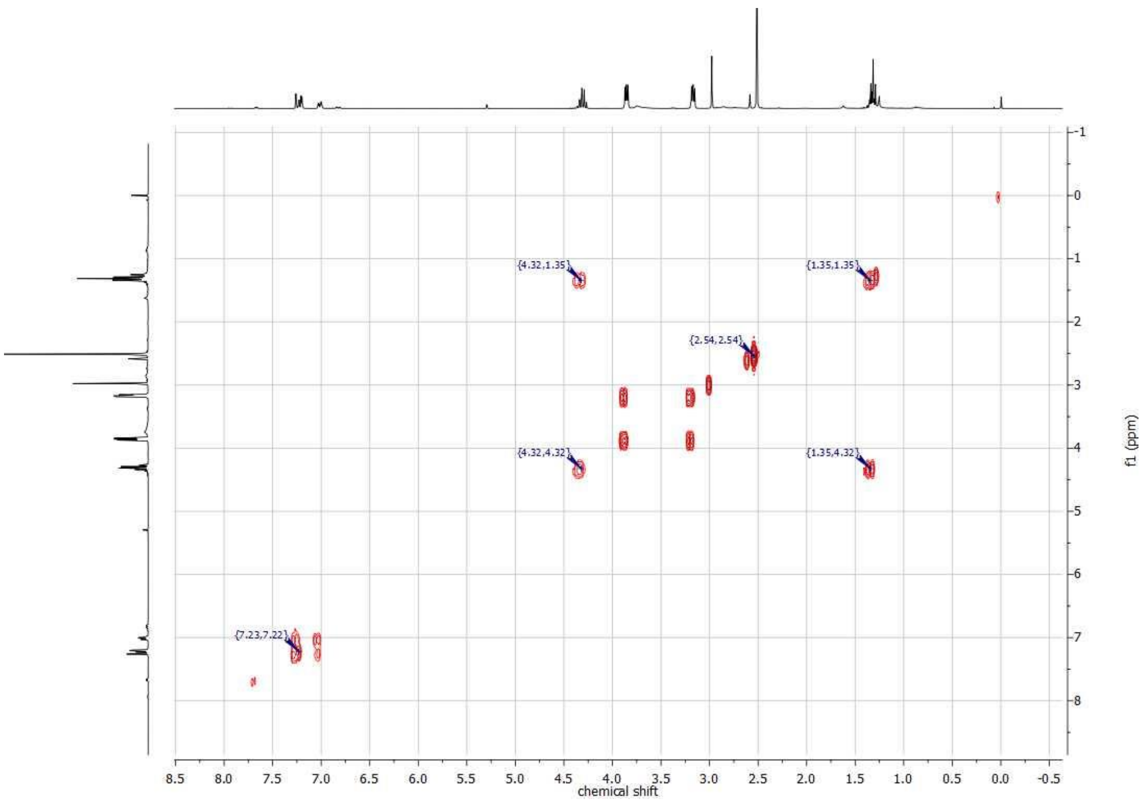

COSY (400 MHz, $CDCl_3$) of compound **16**

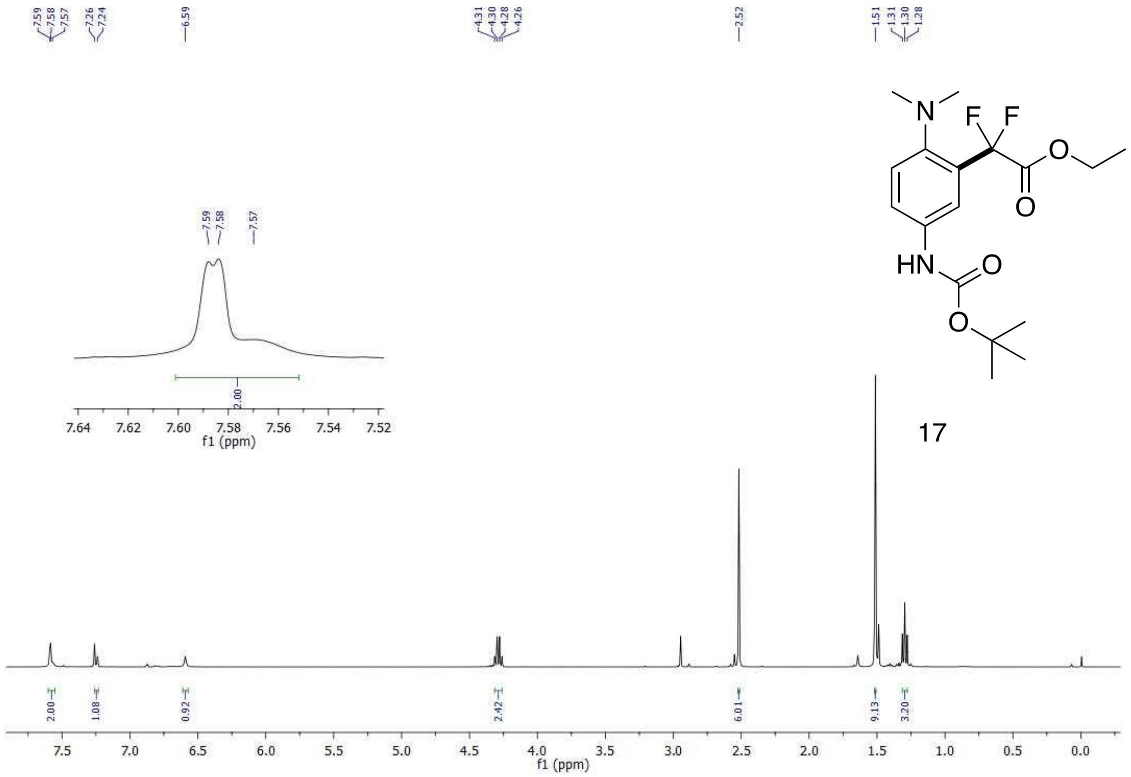

17

^{1}H NMR (400 MHz, $CDCl_3$) of compound **17**

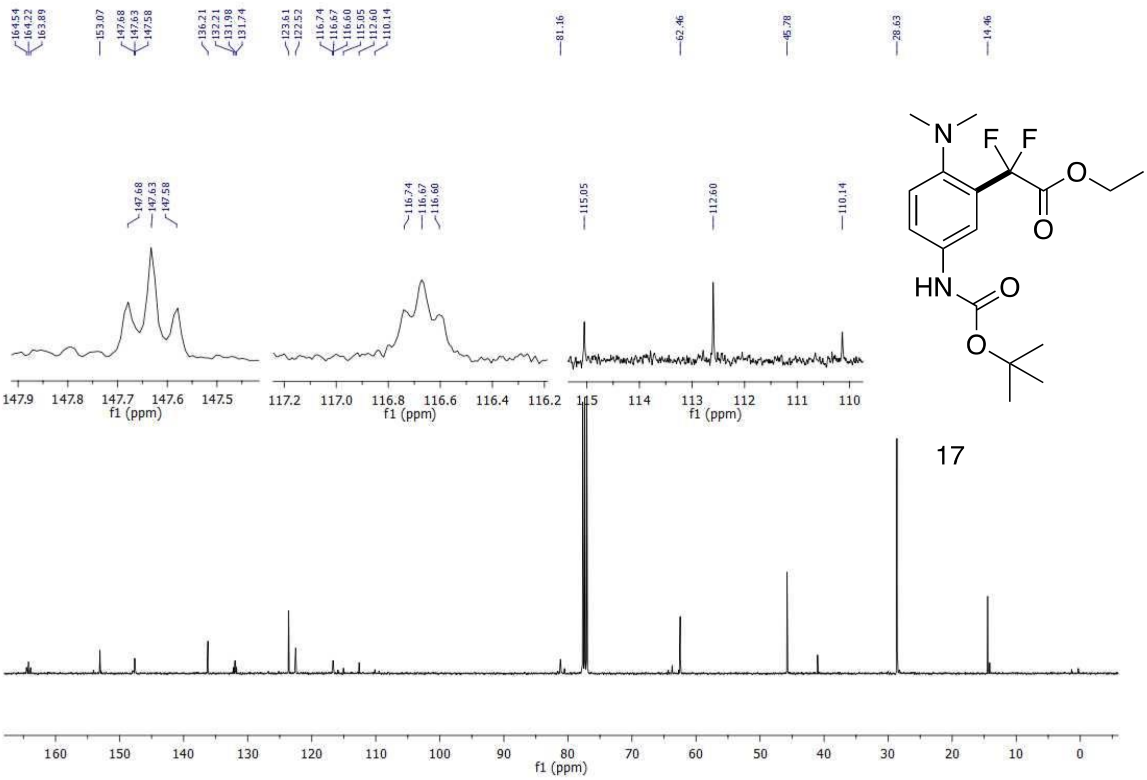

17

$^{13}C\{^{1}H\}$ NMR (100 MHz, $CDCl_3$) of compound **17**

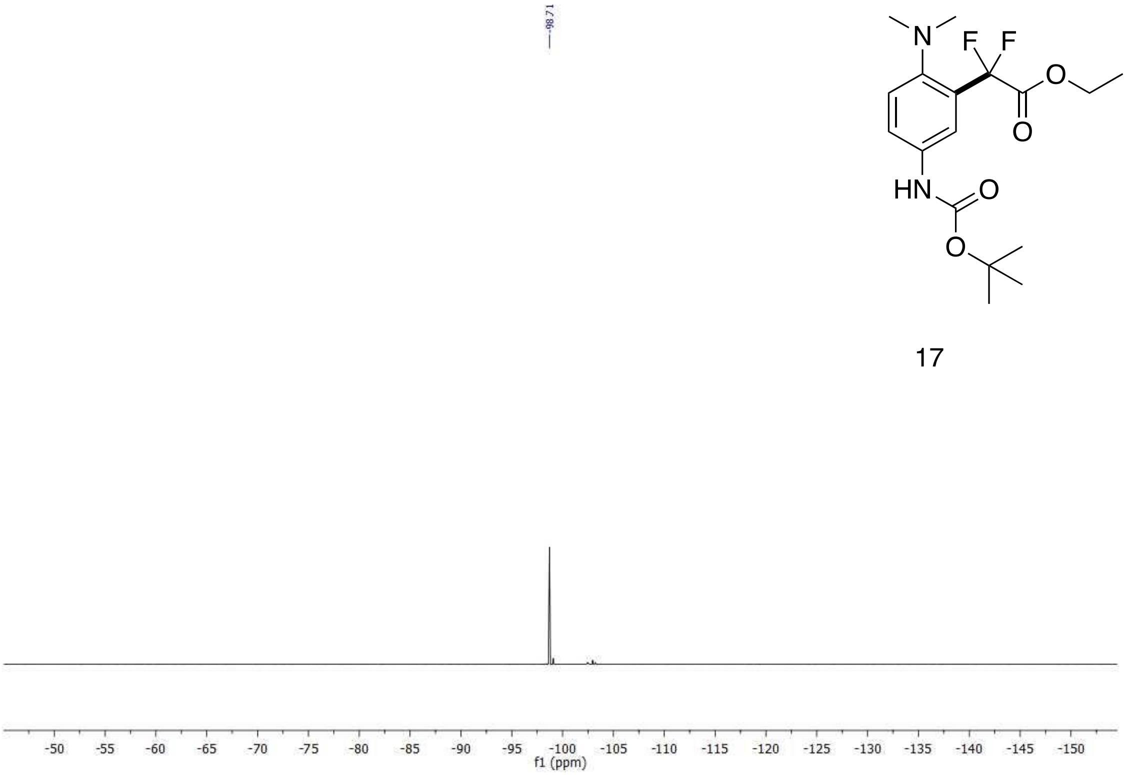

17

^{19}F NMR (376 MHz, $CDCl_3$) of compound **17**

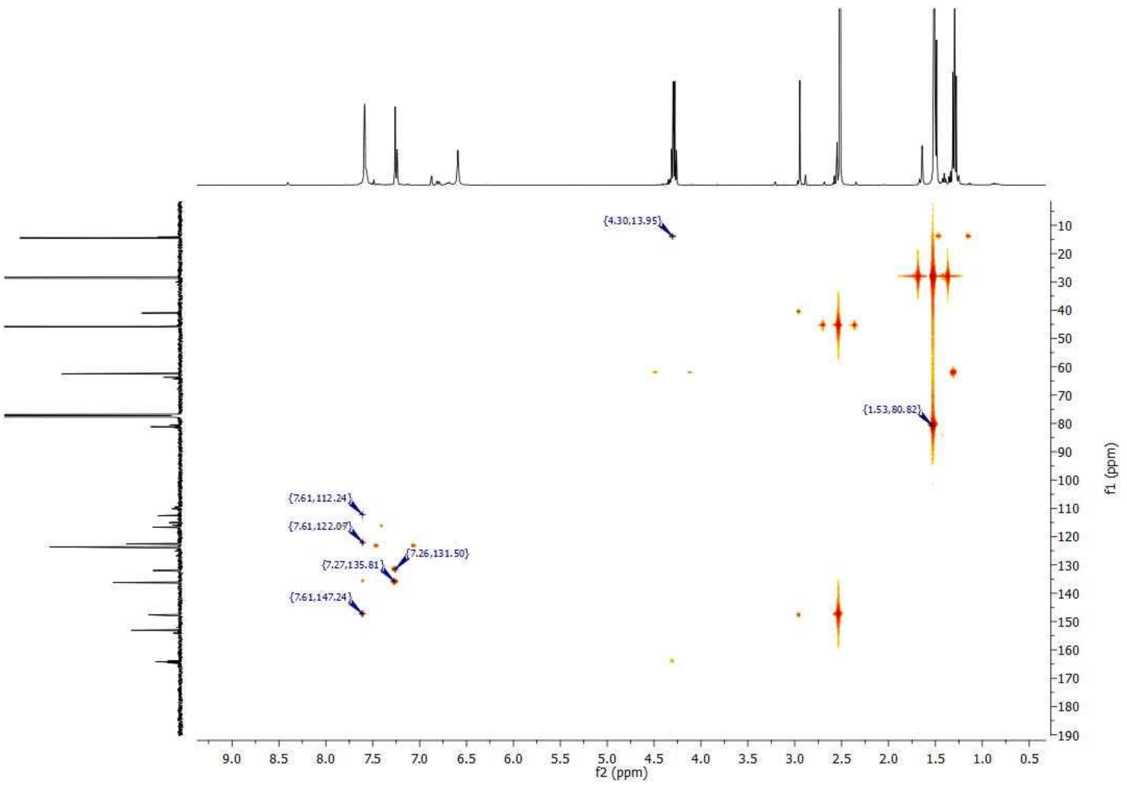

HMBC (100 MHz, $CDCl_3$) of compound **17**

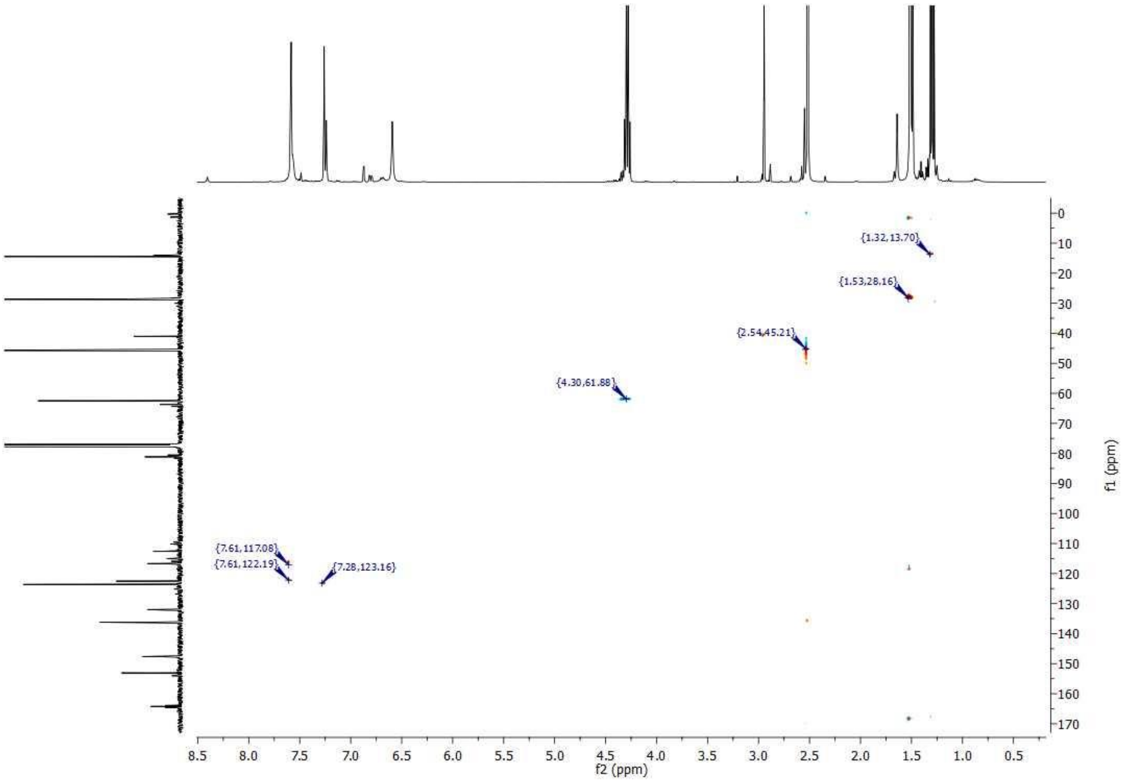

HSQCed (100 MHz, $CDCl_3$) of compound **17**

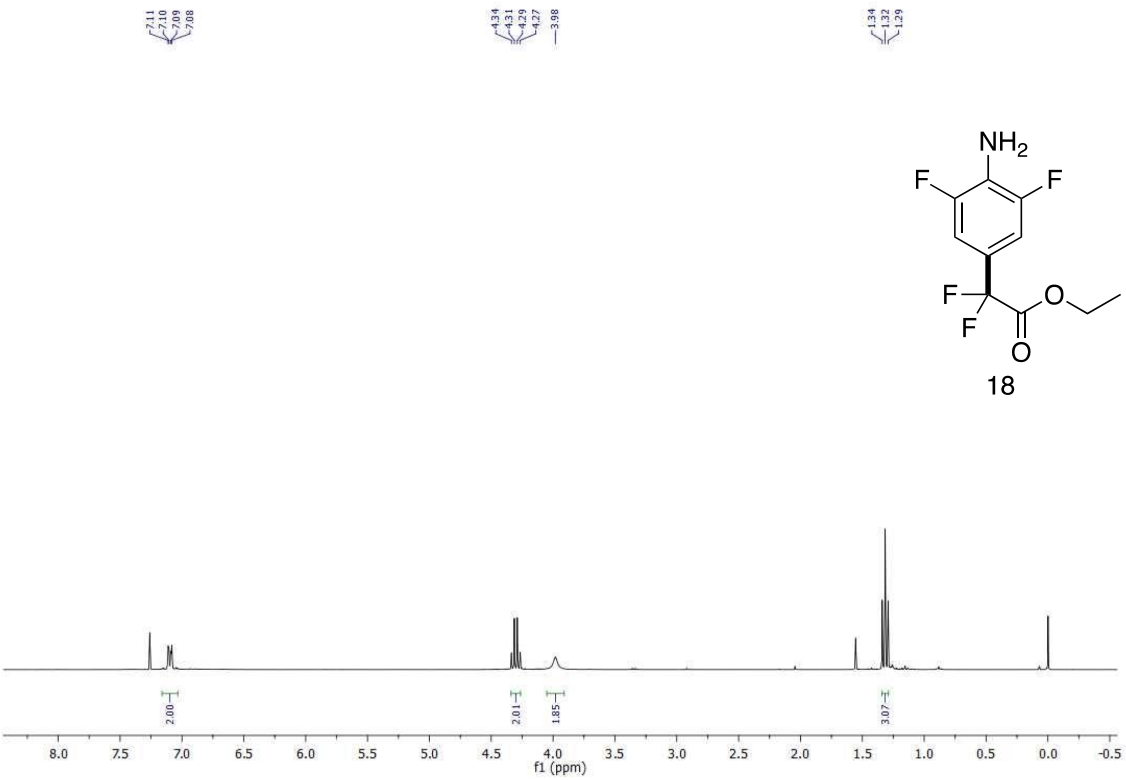

^1H NMR (400 MHz, $CDCl_3$) of compound **18**

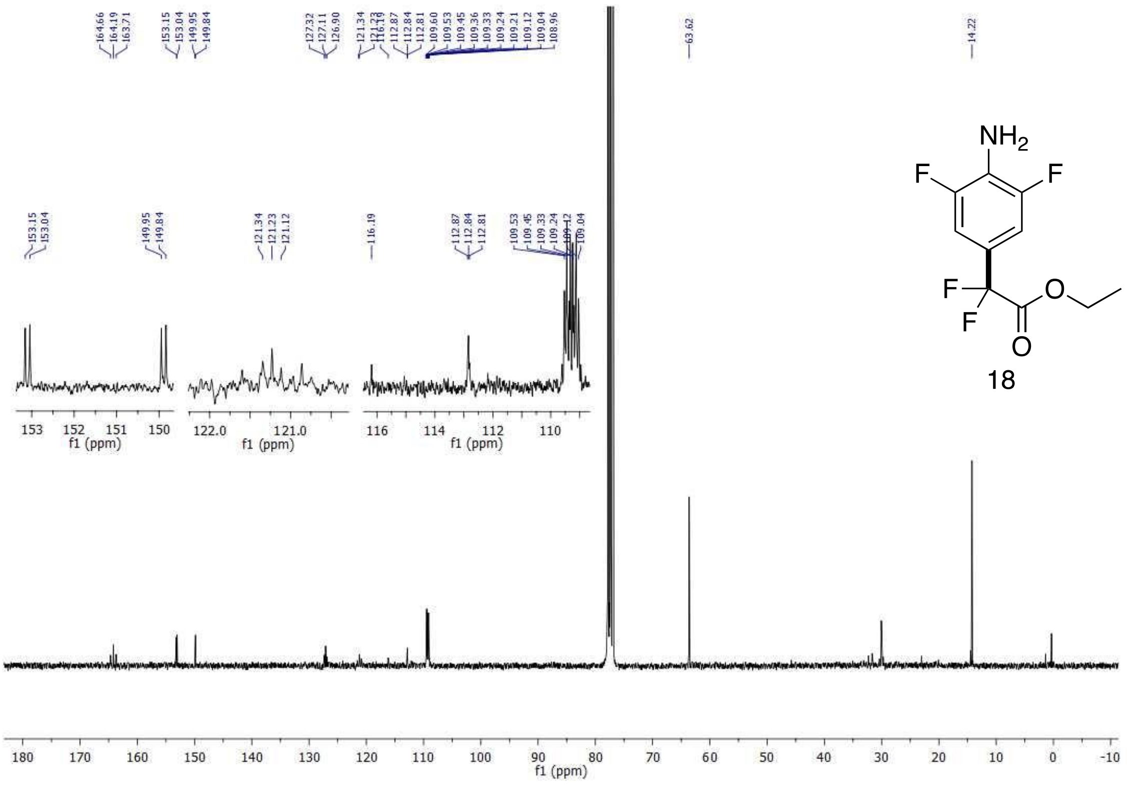

$^{13}C\{^1H\}$ NMR (75 MHz, $CDCl_3$) of compound **18**

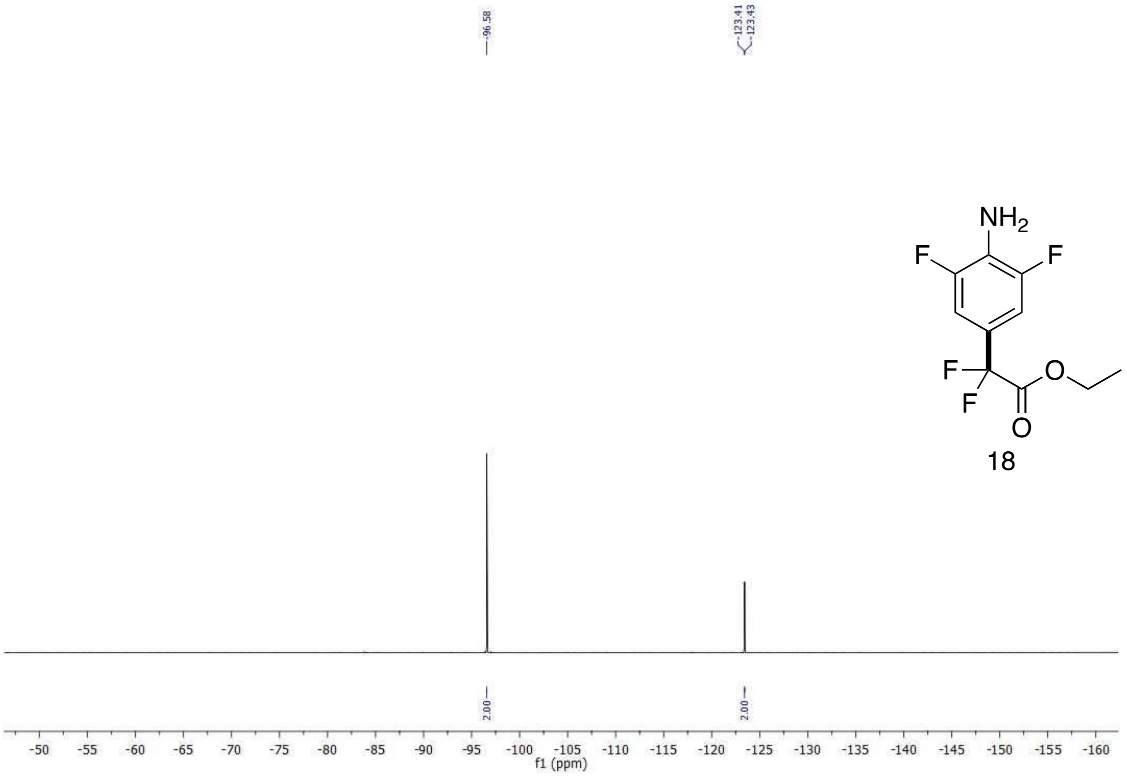

^{19}F NMR (282 MHz, $CDCl_3$) of compound **18**

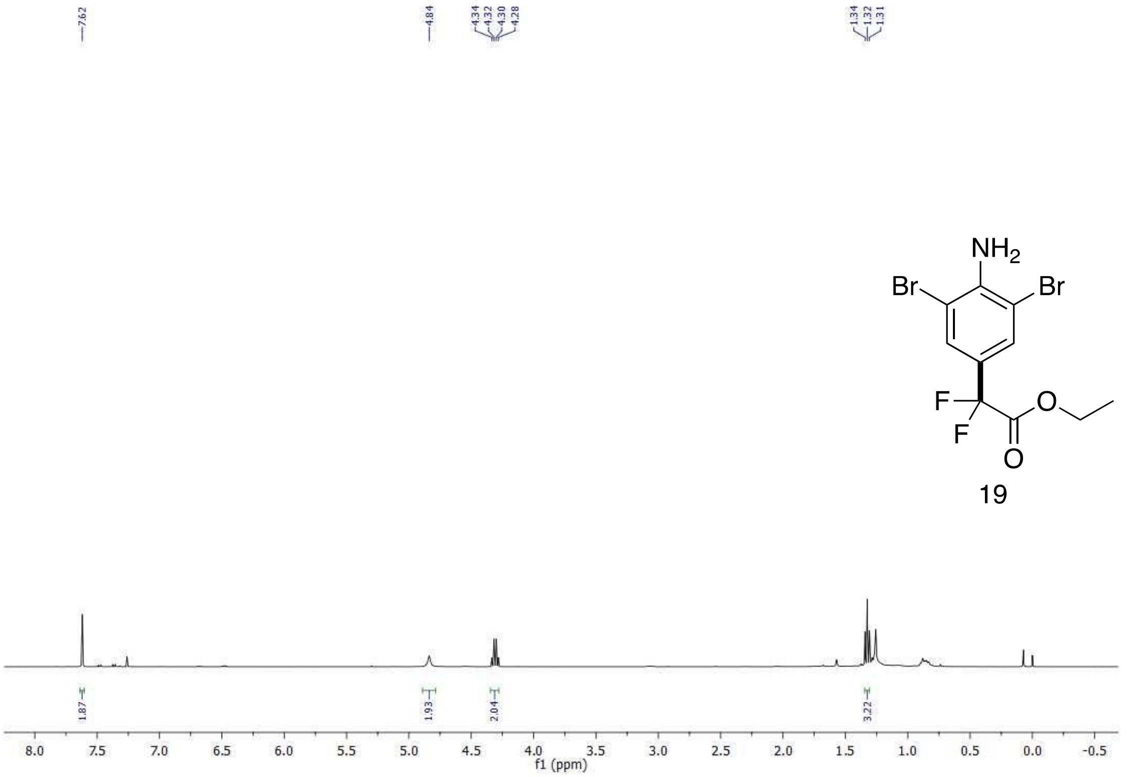

^{1}H NMR (300 MHz, $CDCl_3$) of compound **19**

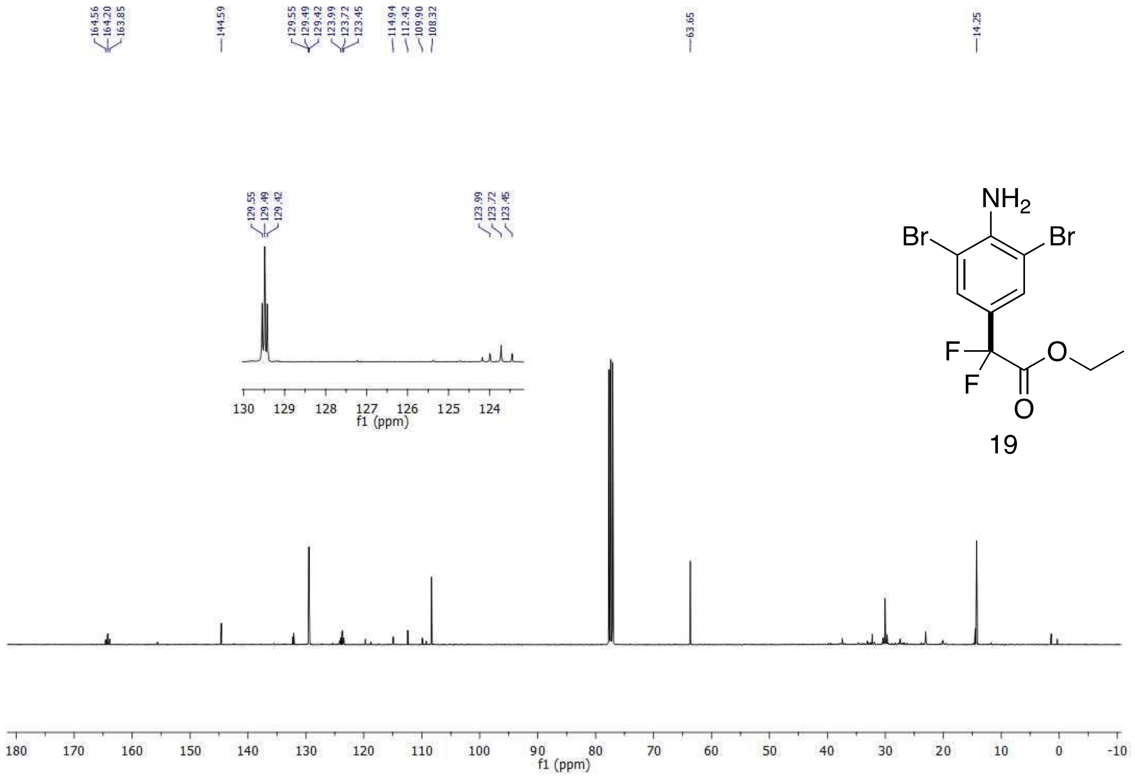

$^{13}C\{^{1}H\}$ NMR (100 MHz, $CDCl_3$) of compound **19**

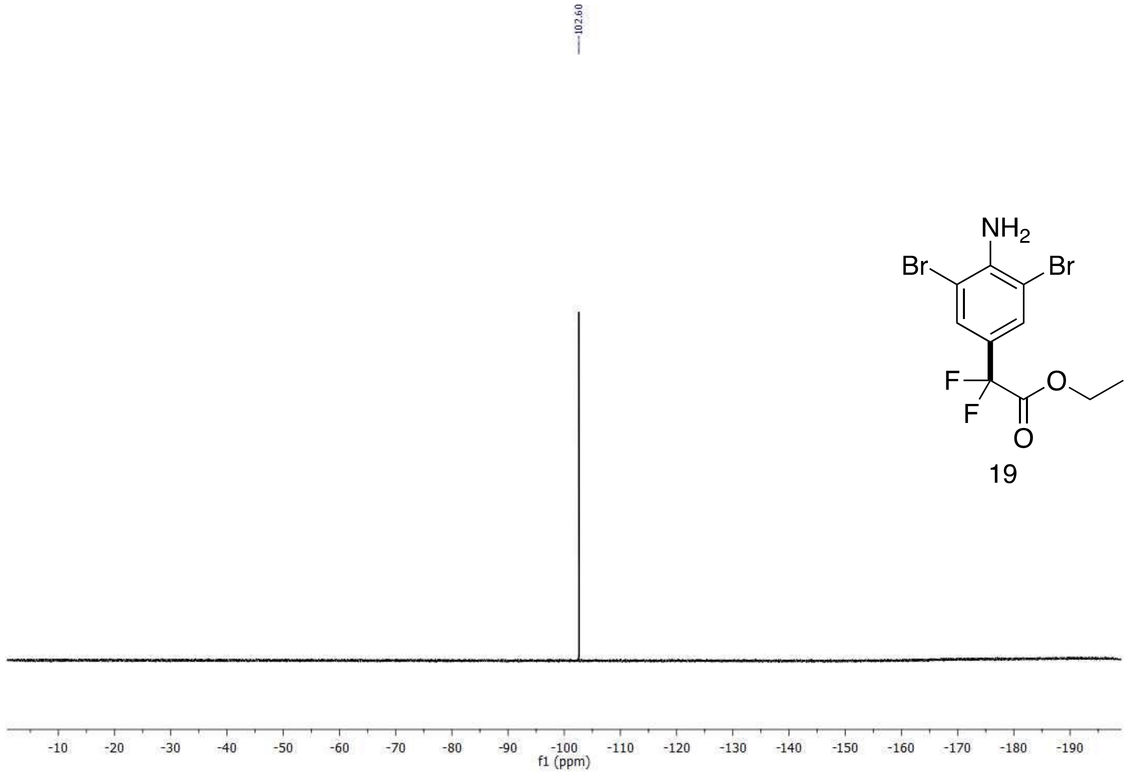

^{19}F NMR (282 MHz, $CDCl_3$) of compound **19**

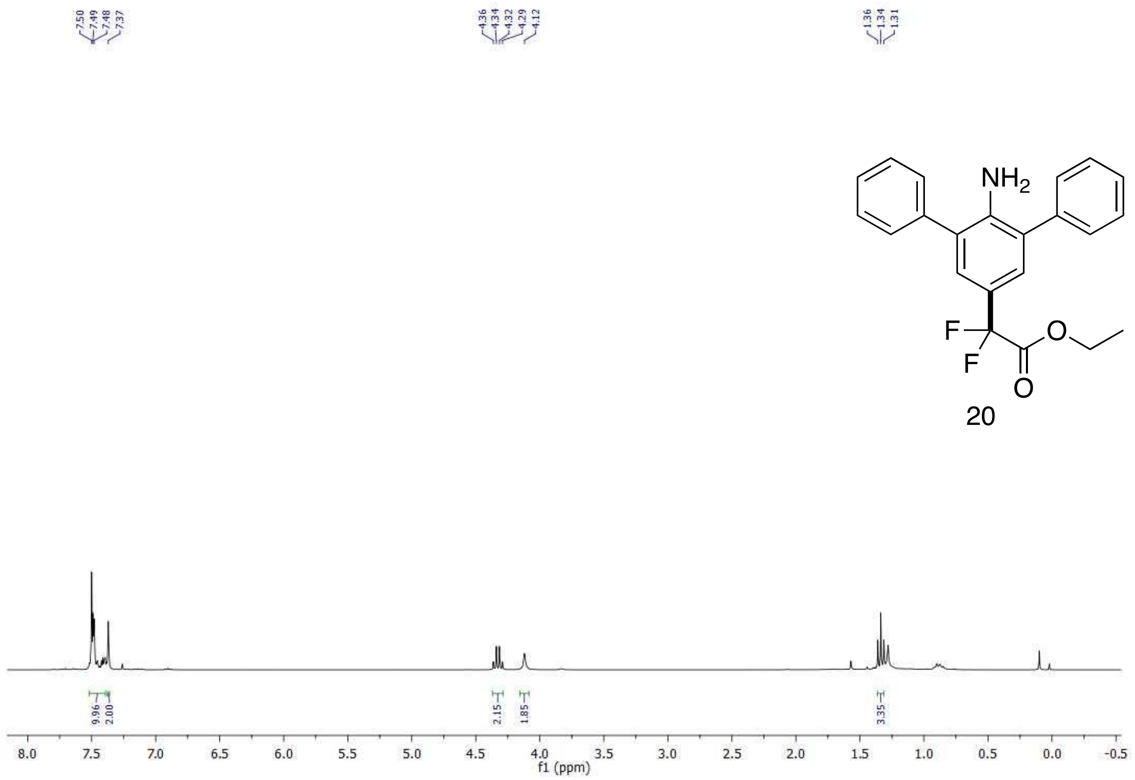

^{1}H NMR (300 MHz, $CDCl_3$) of compound **20**

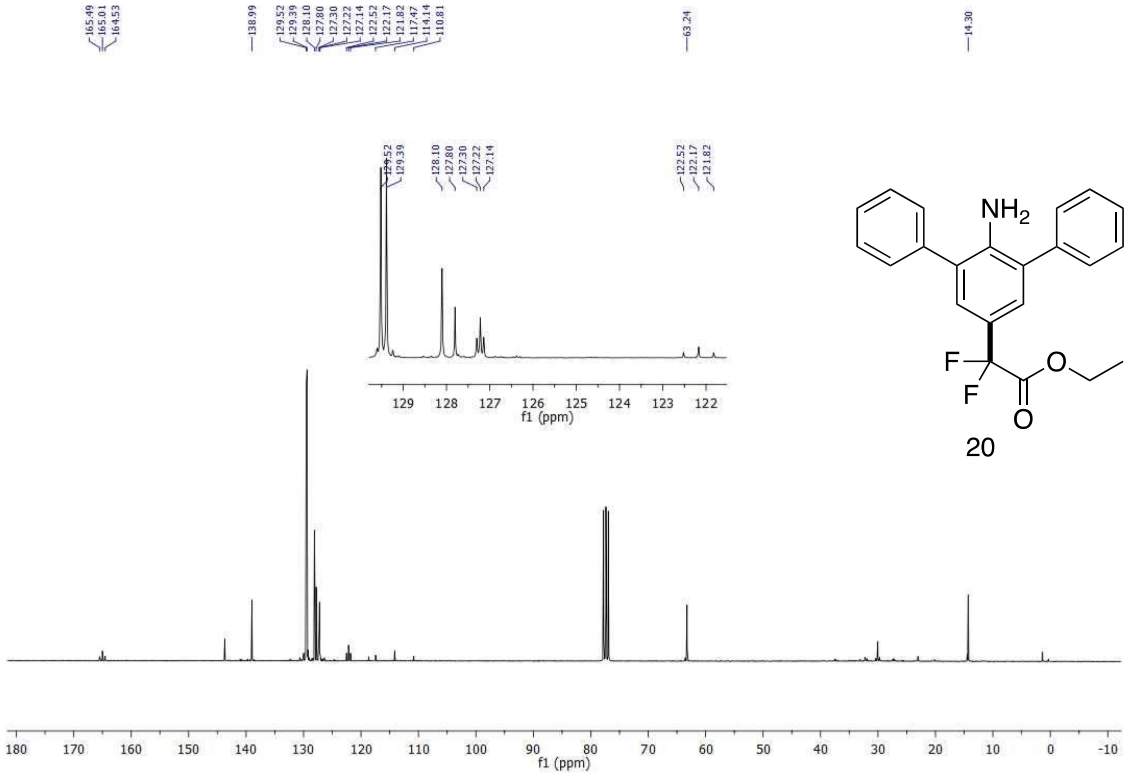

^{13}C{^{1}H} NMR (75 MHz, $CDCl_3$) of compound **20**

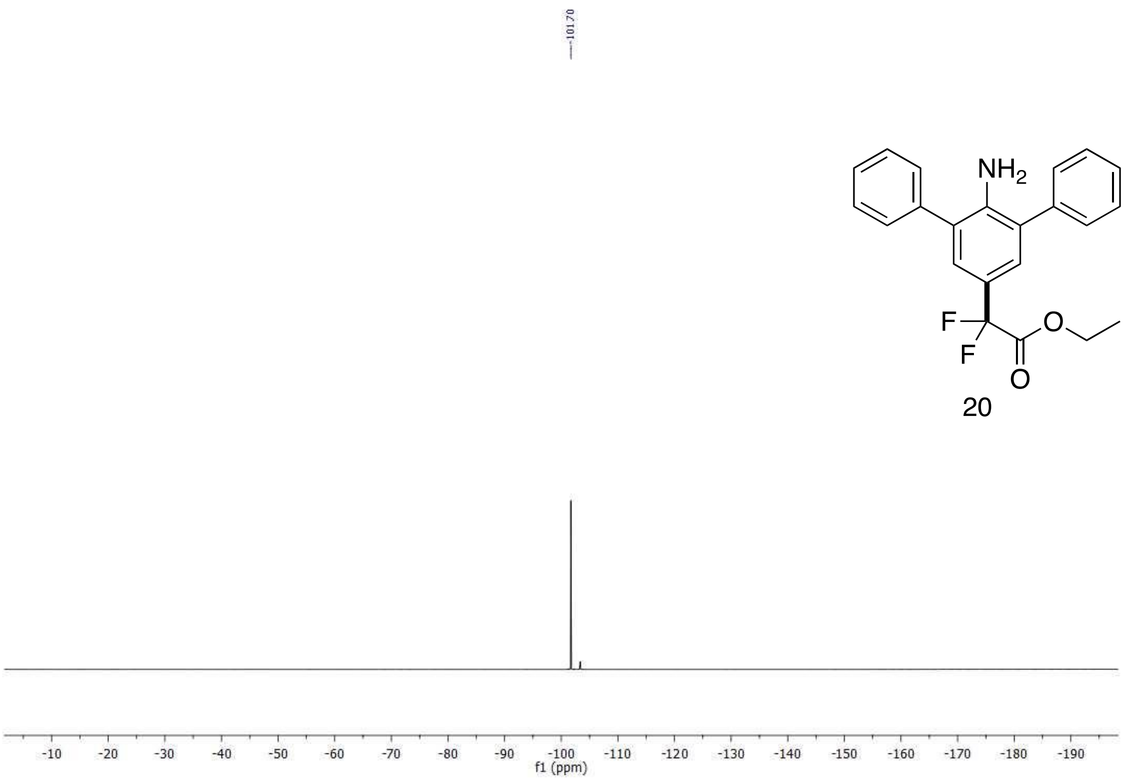

^{19}F NMR (235 MHz, $CDCl_3$) of compound **20**

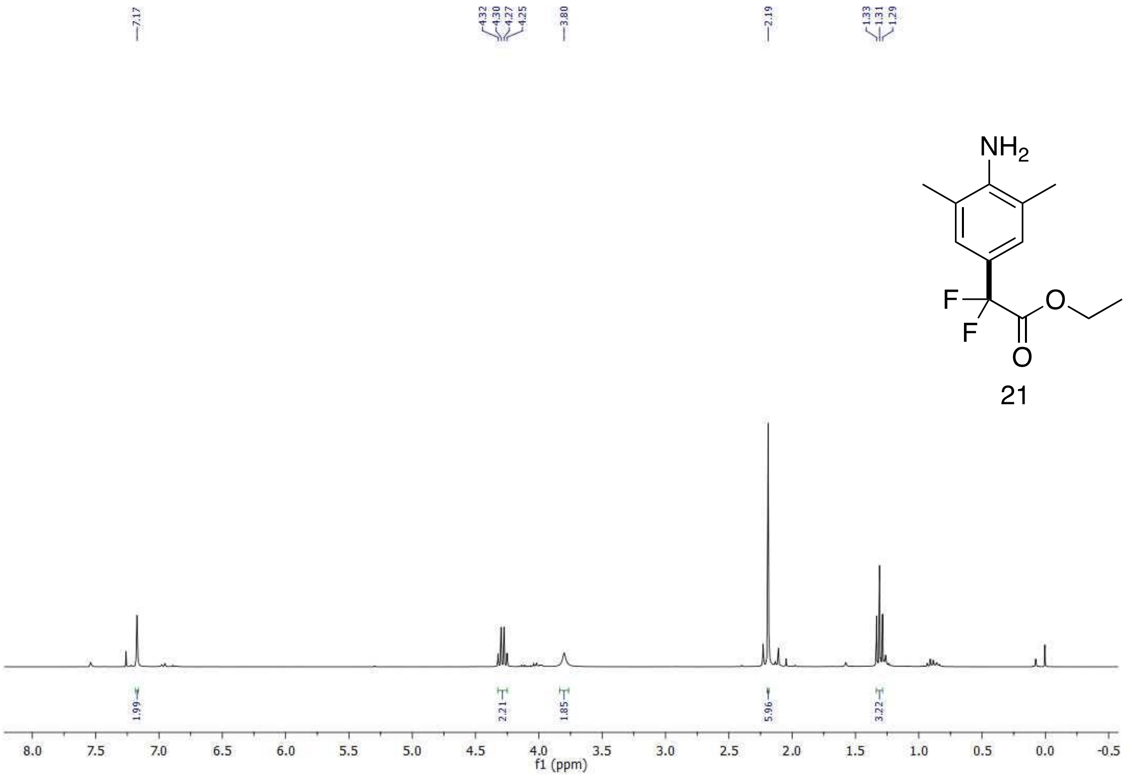

^{1}H NMR (400 MHz, $CDCl_3$) of compound **21**

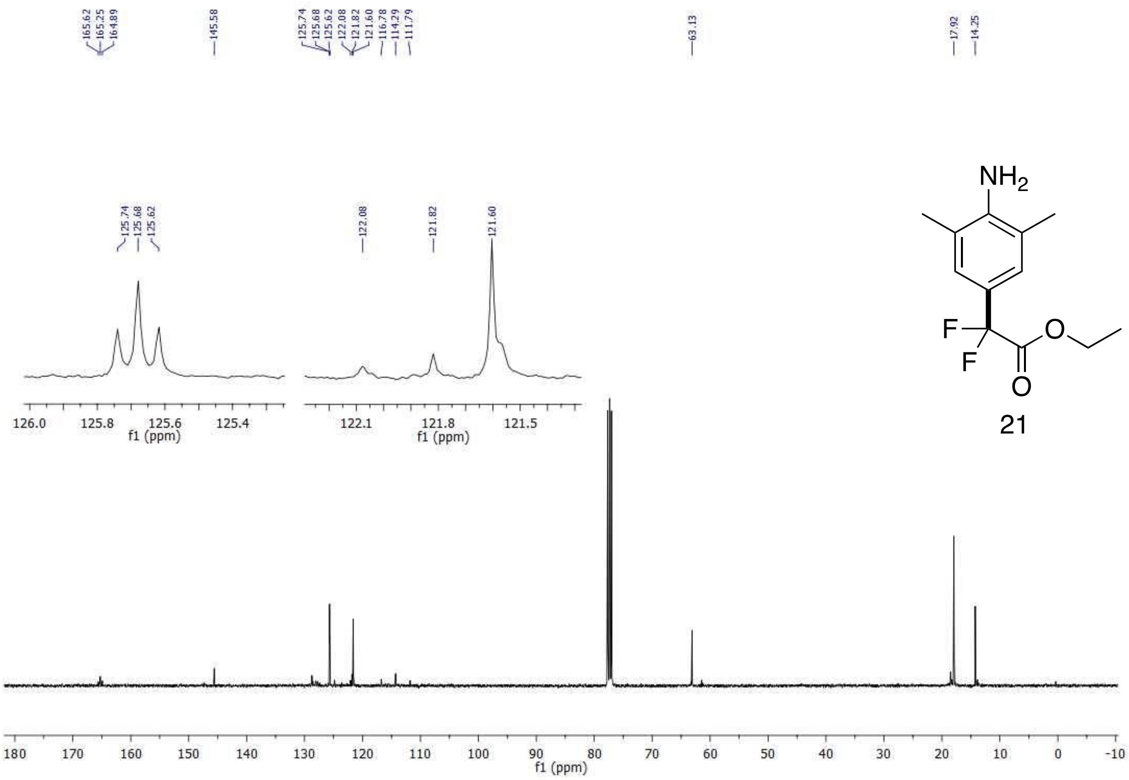

$^{13}C\{^{1}H\}$ NMR (100 MHz, $CDCl_3$) of compound **21**

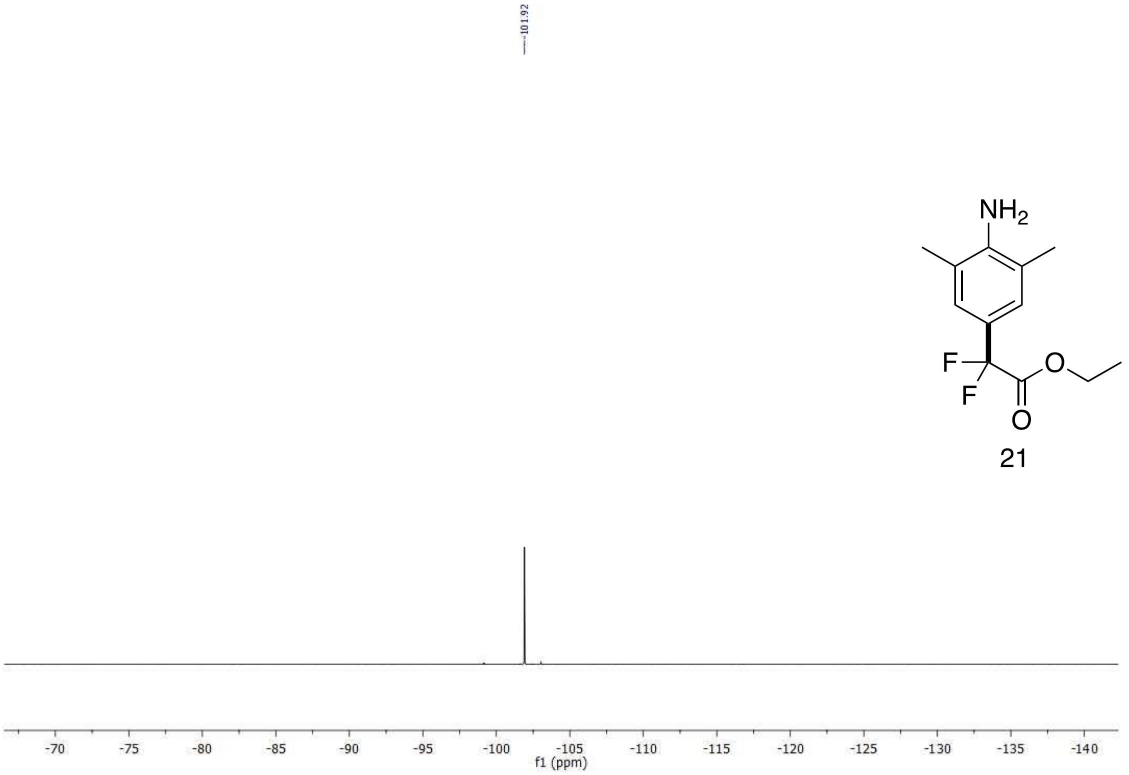

^{19}F NMR (282 MHz, $CDCl_3$) of compound **21**

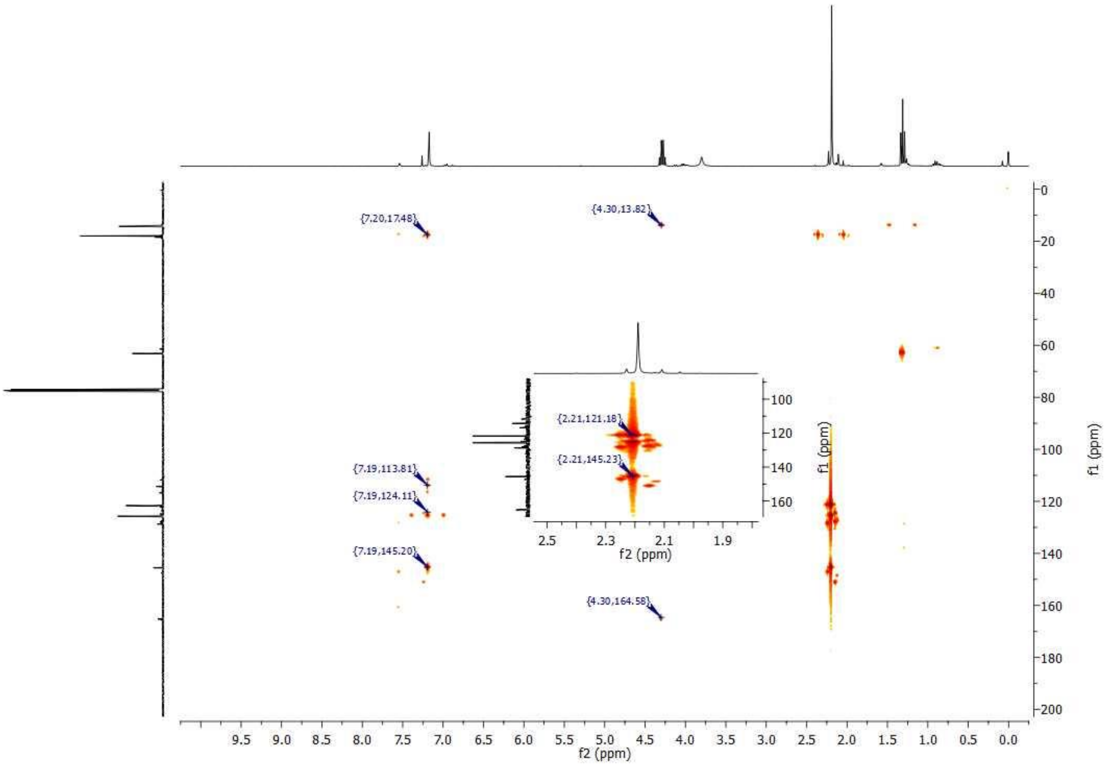

HMBC (100 MHz, $CDCl_3$) of compound **21**

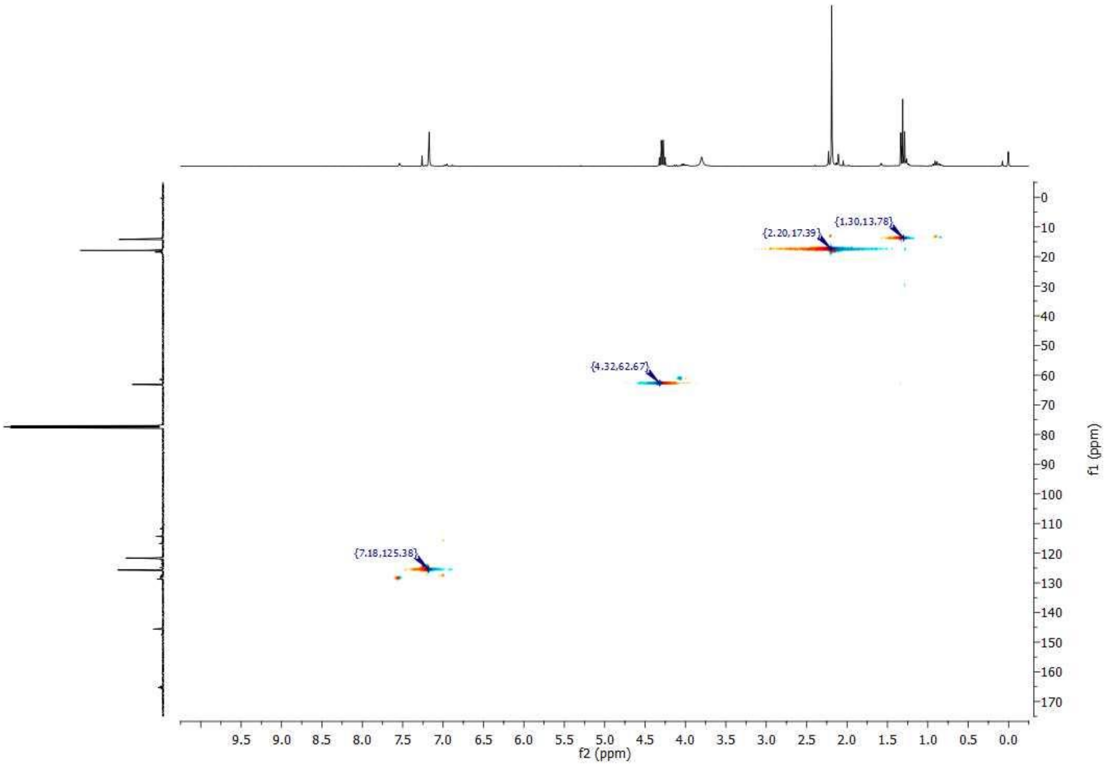

HSQCed (100 MHz, $CDCl_3$) of compound **21**

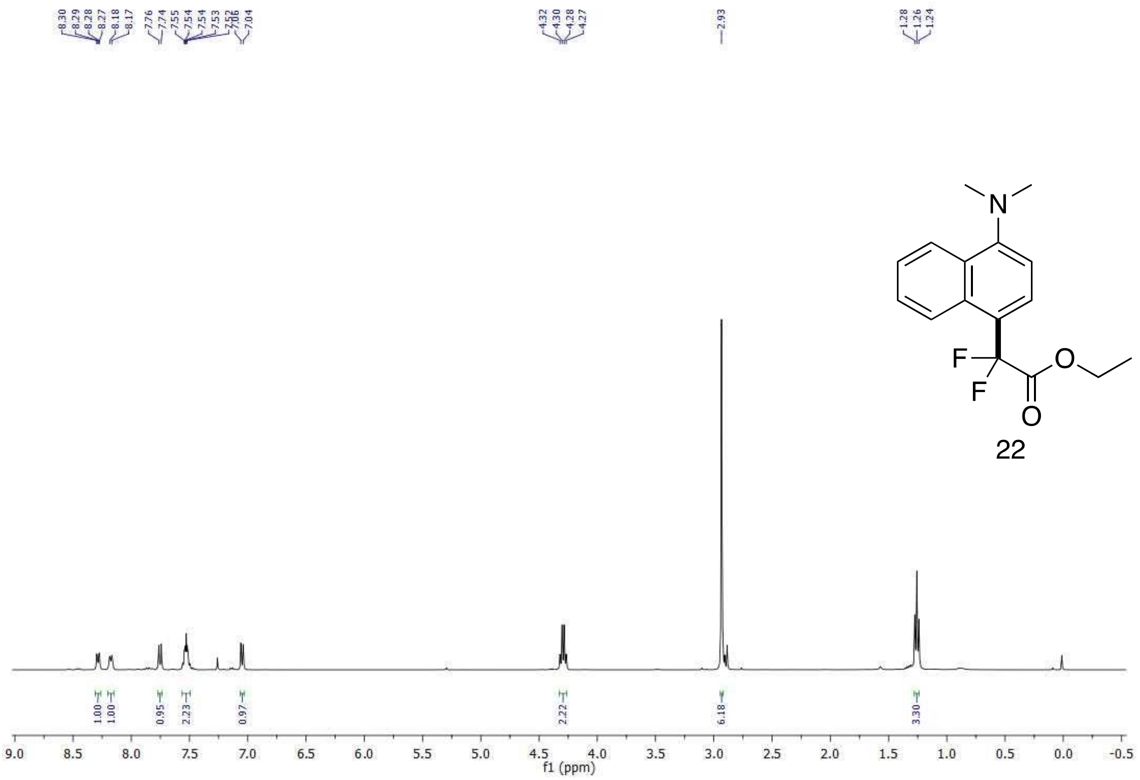

^{1}H NMR (400 MHz, $CDCl_3$) of compound **22**

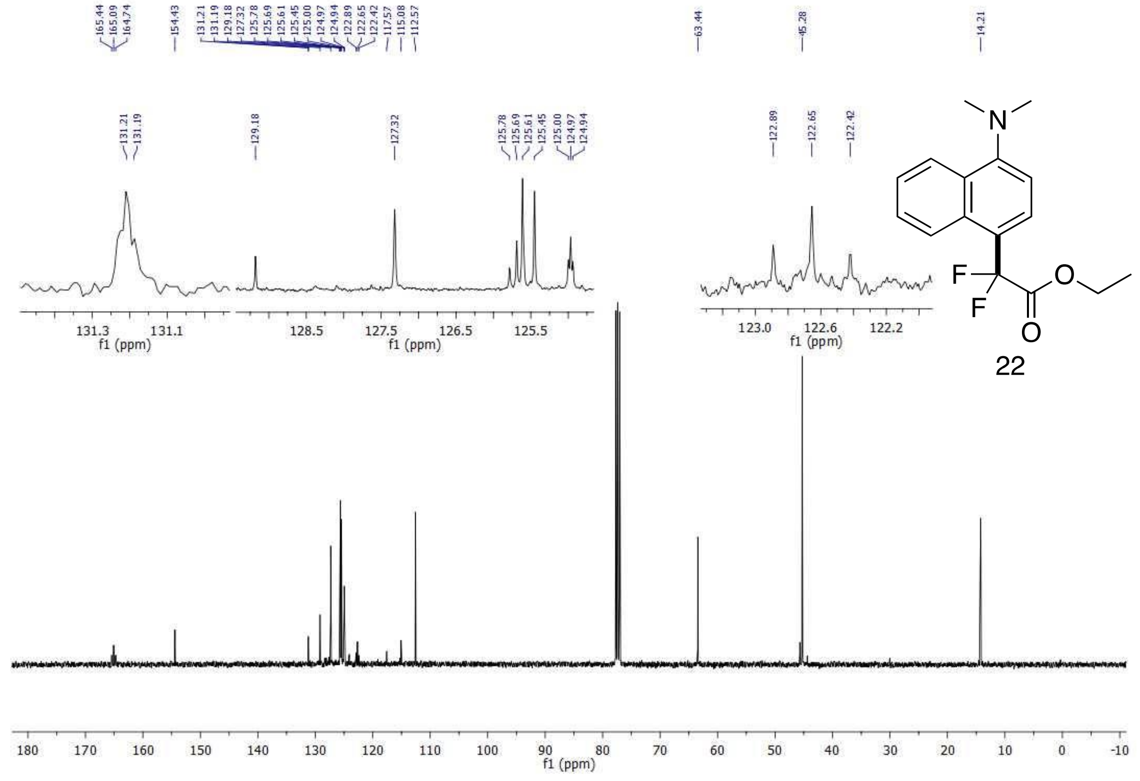

$^{13}C\{^{1}H\}$ NMR (100 MHz, $CDCl_3$) of compound **22**

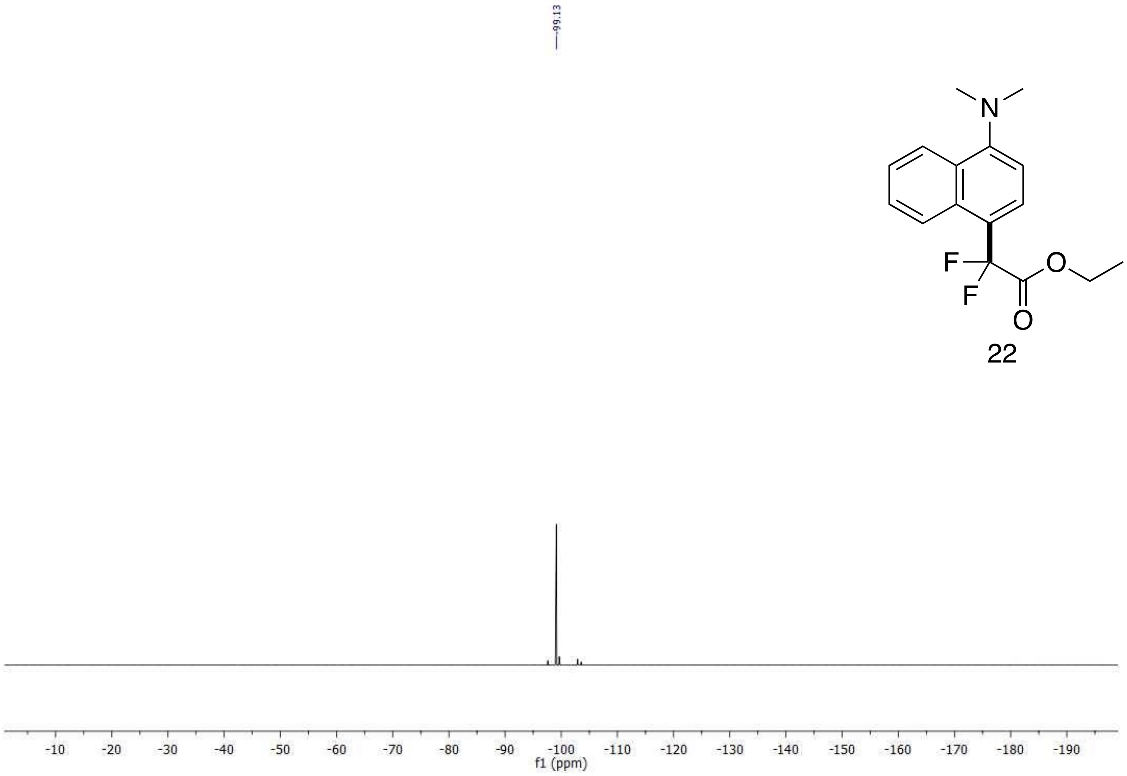

22

^{19}F NMR (376 MHz, $CDCl_3$) of compound **22**

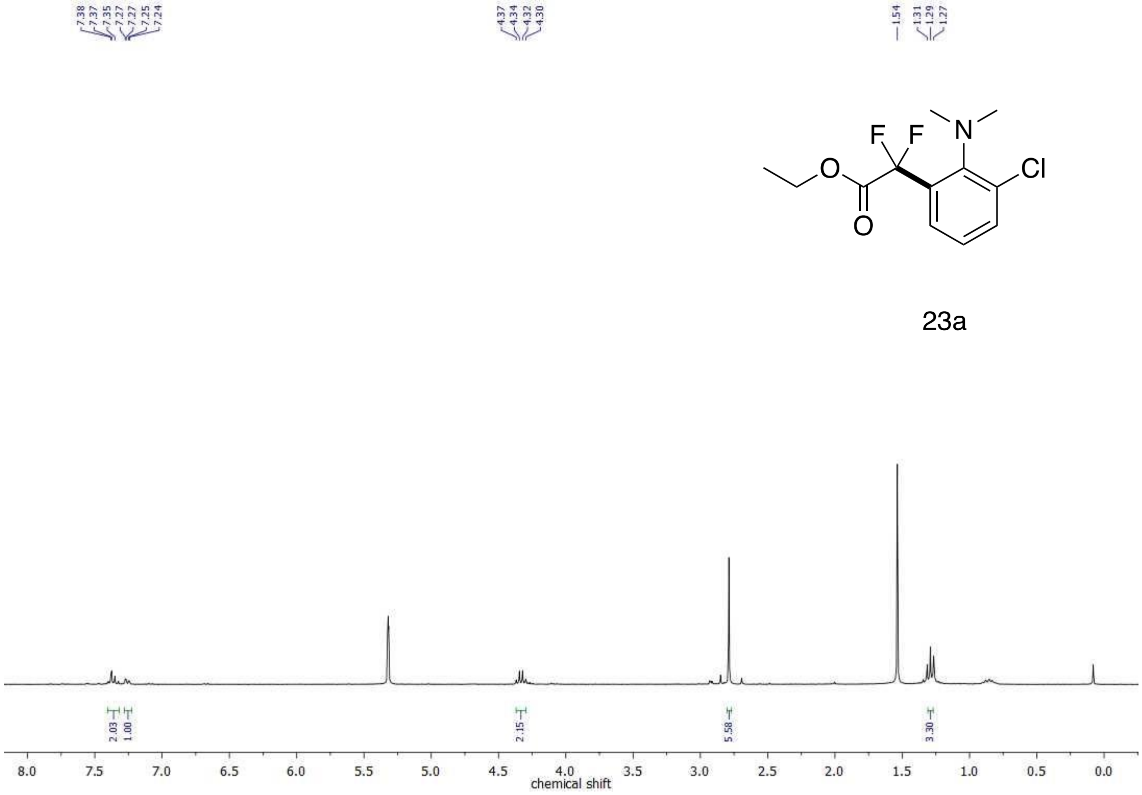

23a

^{1}H NMR (300 MHz, CD_2Cl_2) of compound **23a**

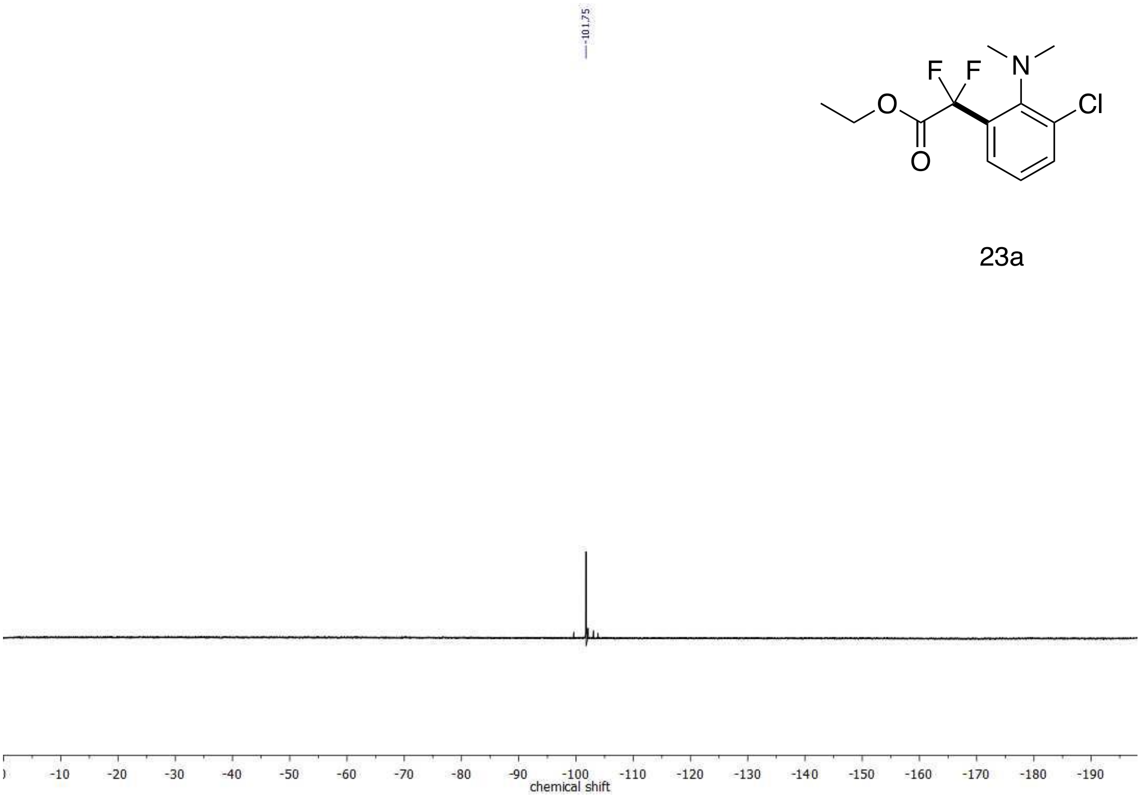

^{19}F NMR (282 MHz, CD_2Cl_2) of compound **23a**

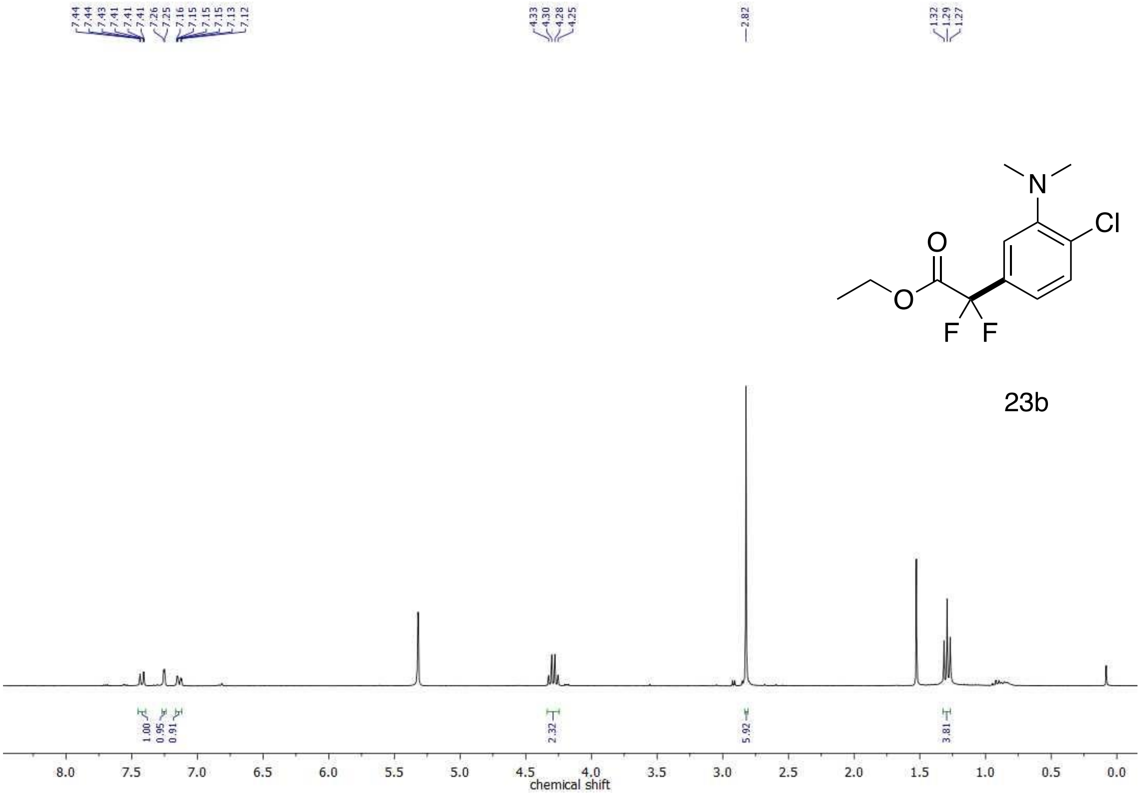

^{1}H NMR (300 MHz, CD_2Cl_2) of compound **23b**

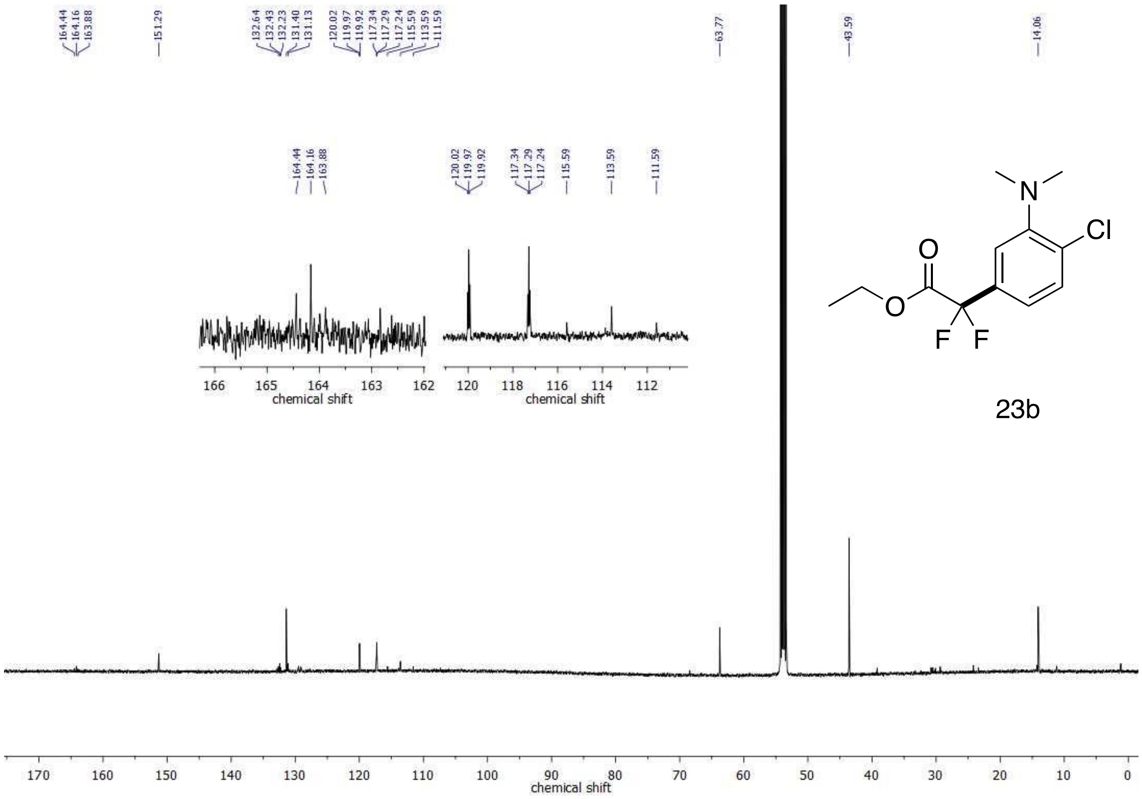

$^{13}C\{^1H\}$ NMR (125 MHz, CD_2Cl_2) of compound **23b**

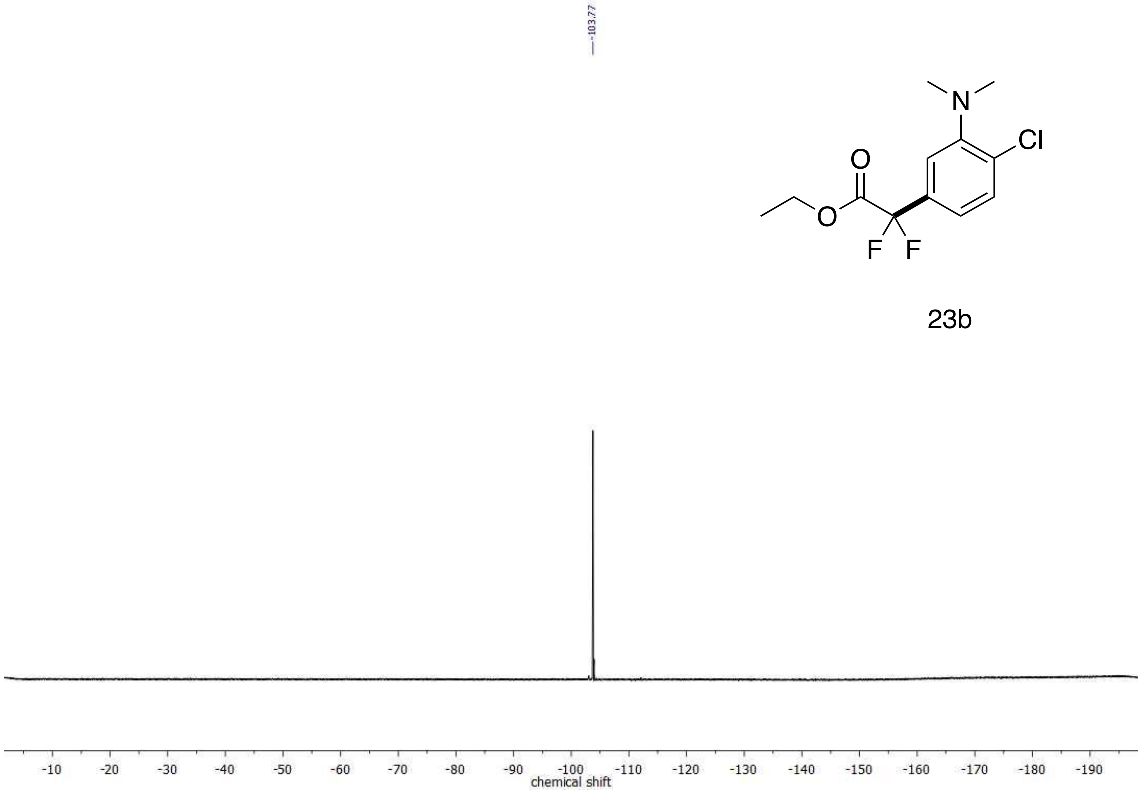

^{19}F NMR (282 MHz, CD_2Cl_2) of compound **23b**

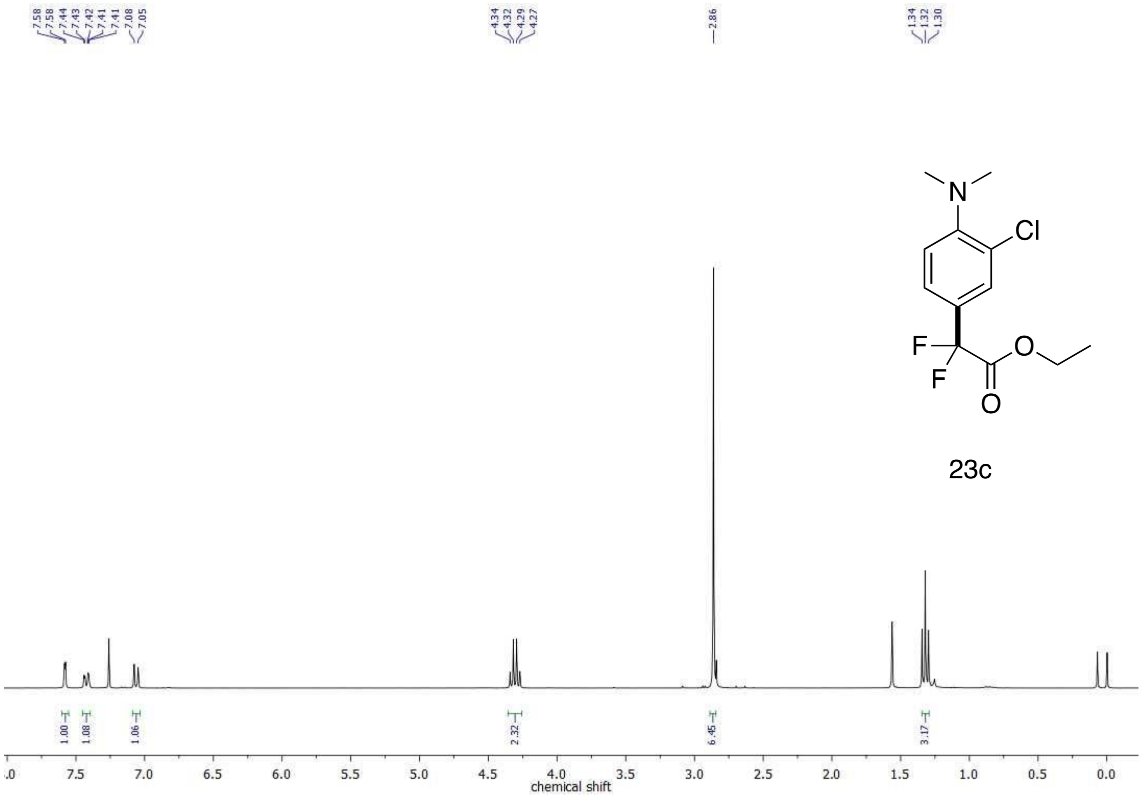

^{1}H NMR (300 MHz, $CDCl_3$) of compound **23c**

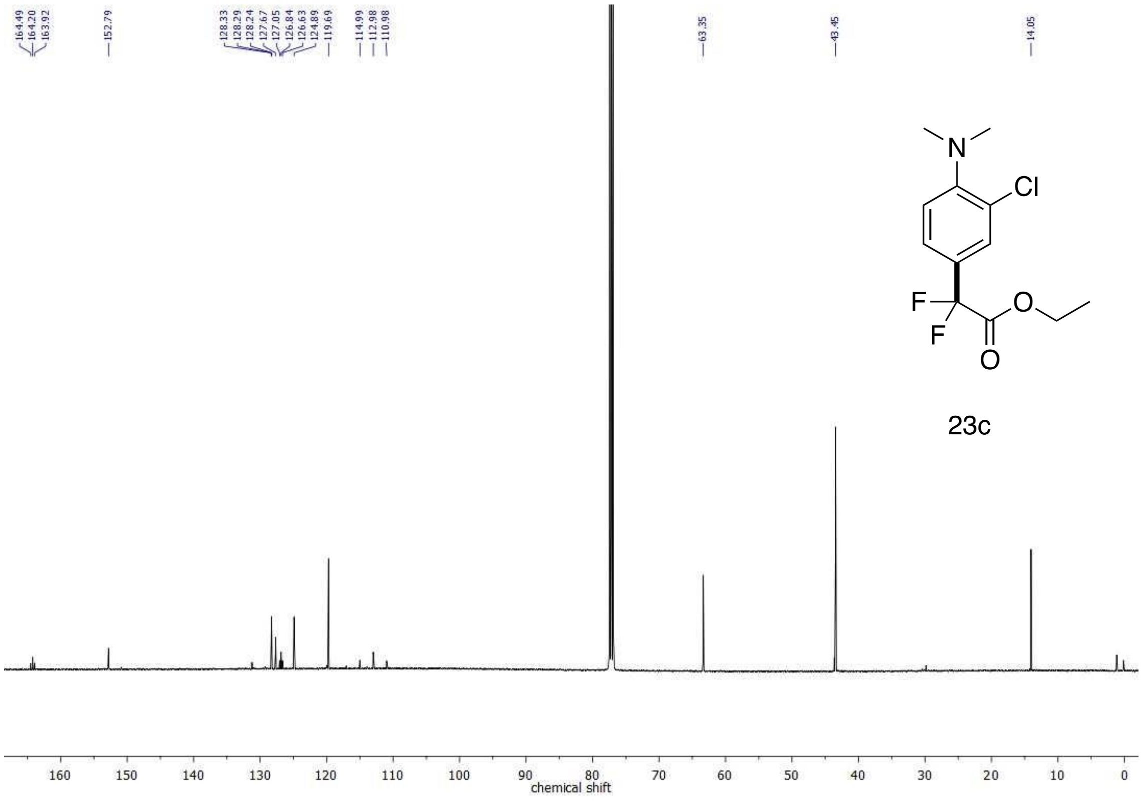

^{13}C{^{1}H} NMR (125 MHz, $CDCl_3$) of compound **23c**

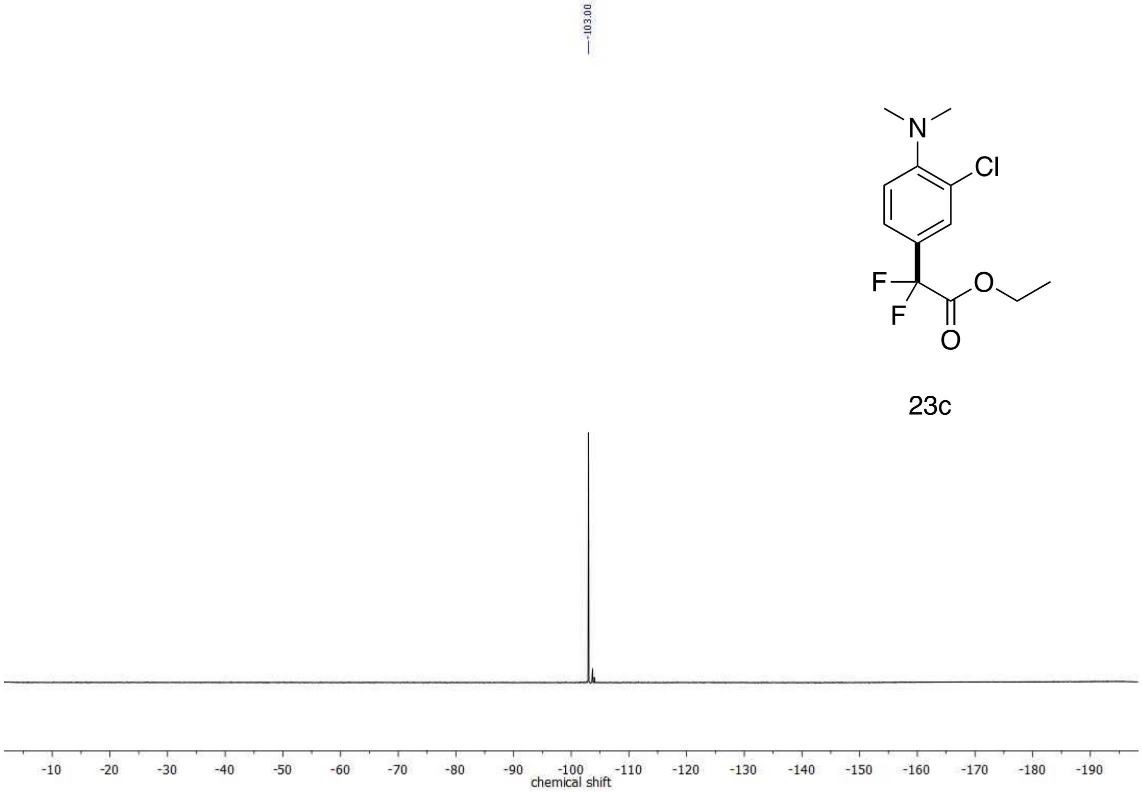

^{19}F NMR (282 MHz, $CDCl_3$) of compound **23c**

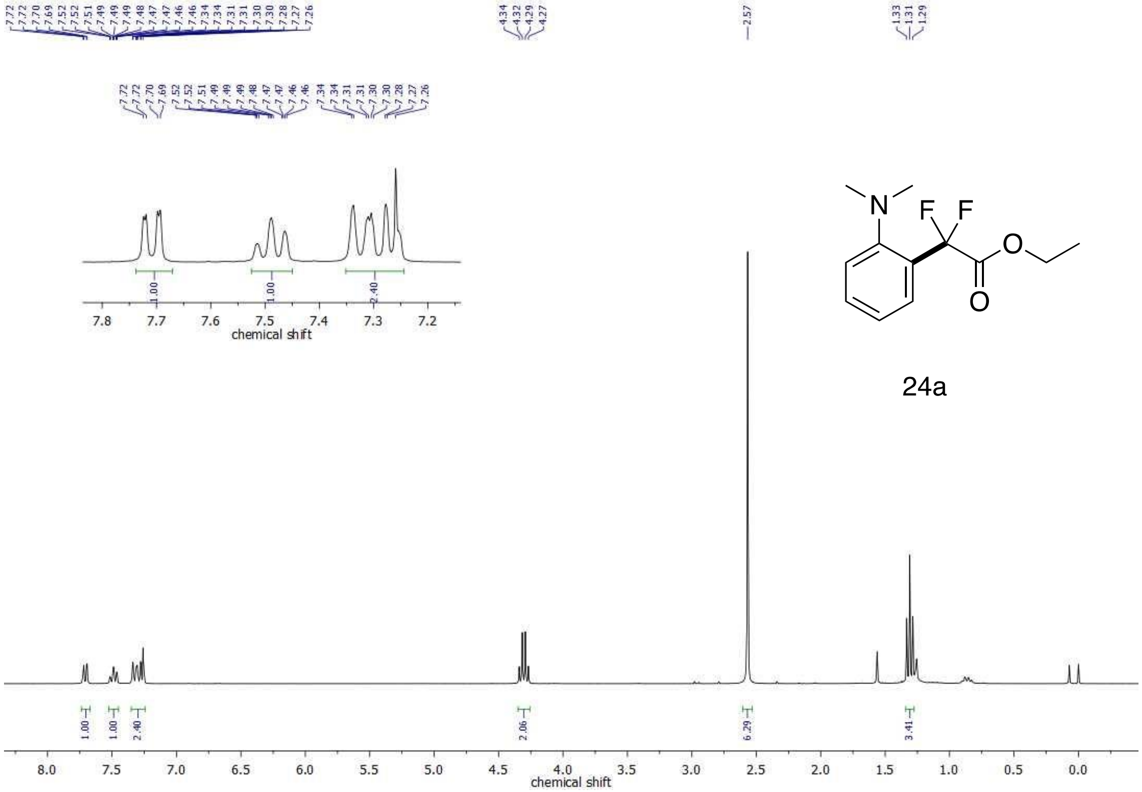

^{1}H NMR (300 MHz, $CDCl_3$) of compound **24a**

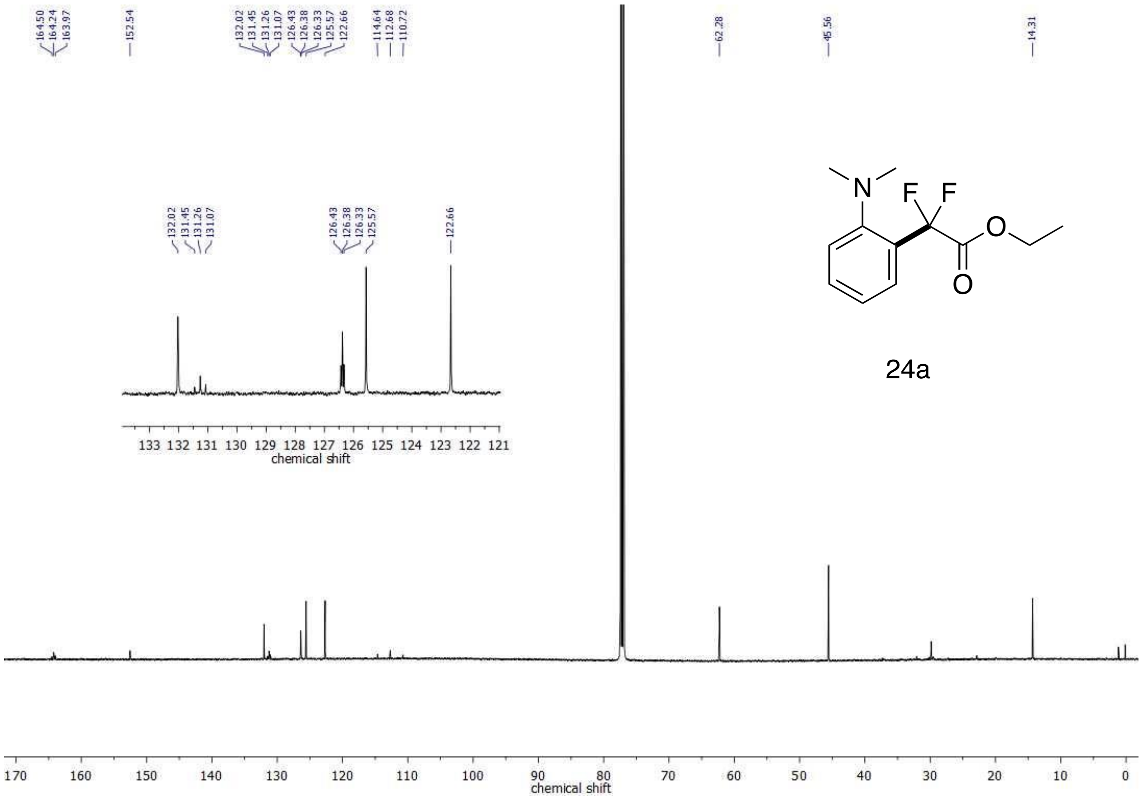

$^{13}C\{^{1}H\}$ NMR (125 MHz, $CDCl_3$) of compound **24a**

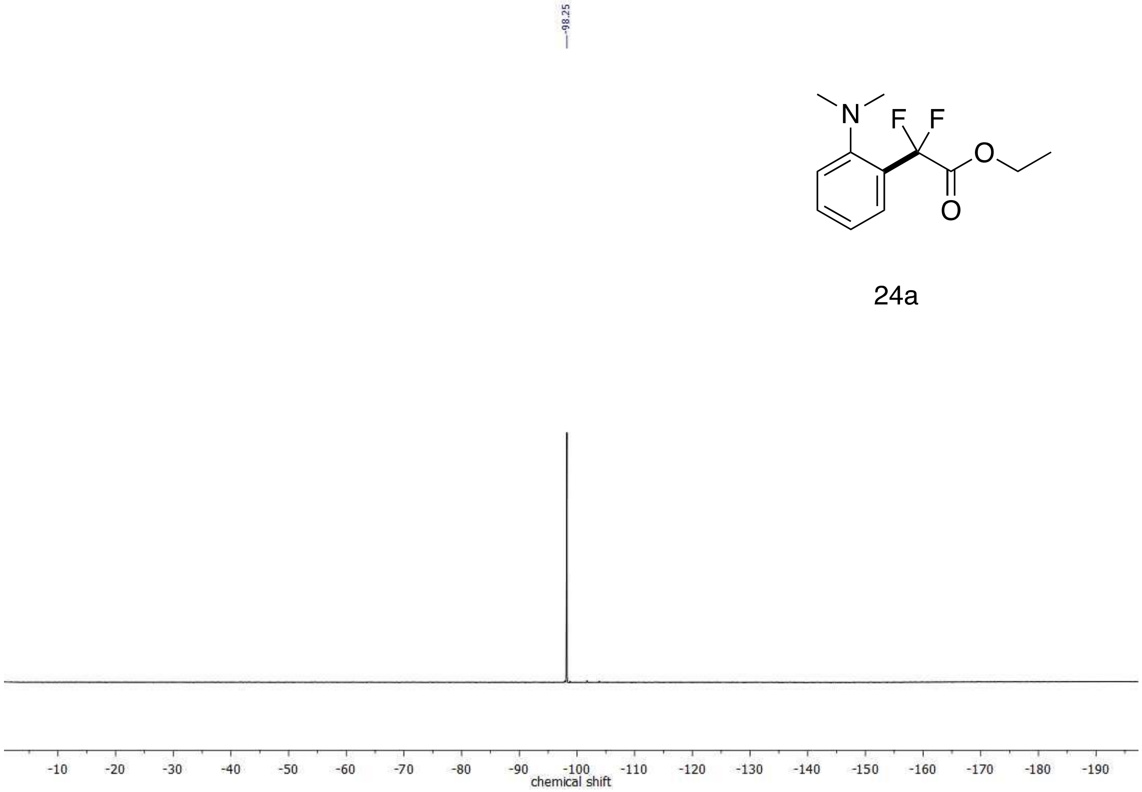

^{19}F NMR (282 MHz, $CDCl_3$) of compound **24a**

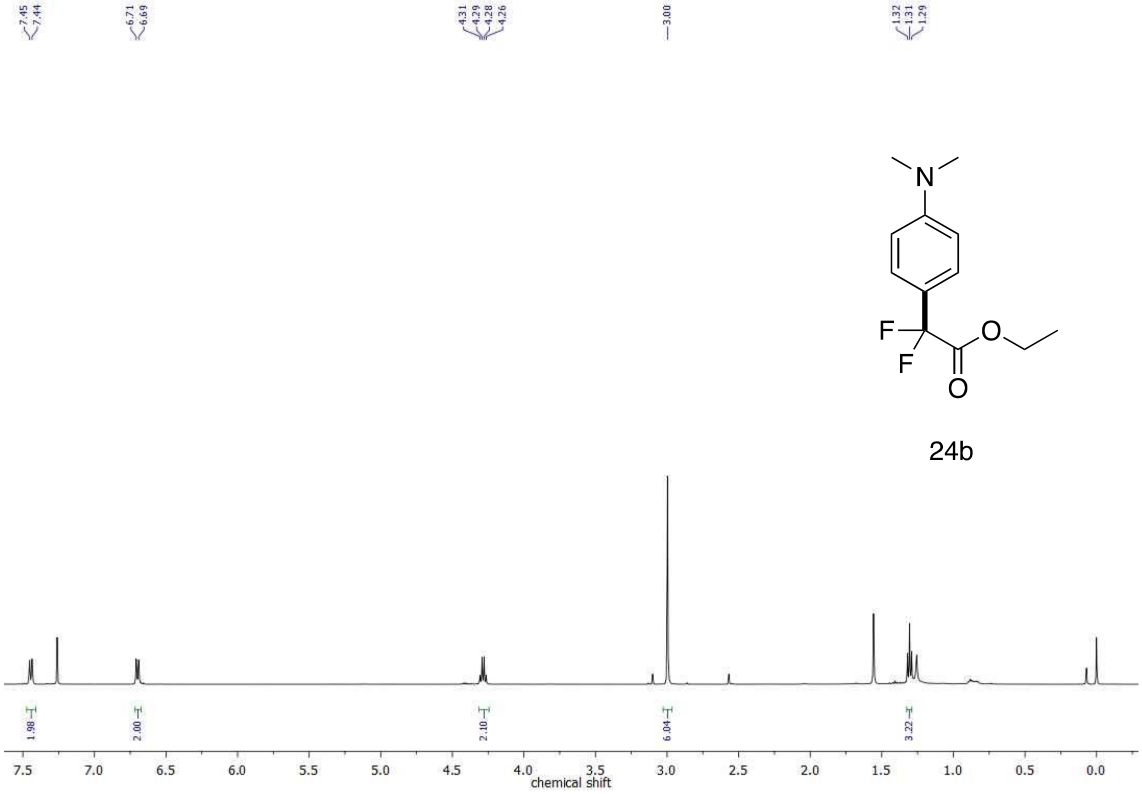

^{1}H NMR (500 MHz, $CDCl_3$) of compound **24b**

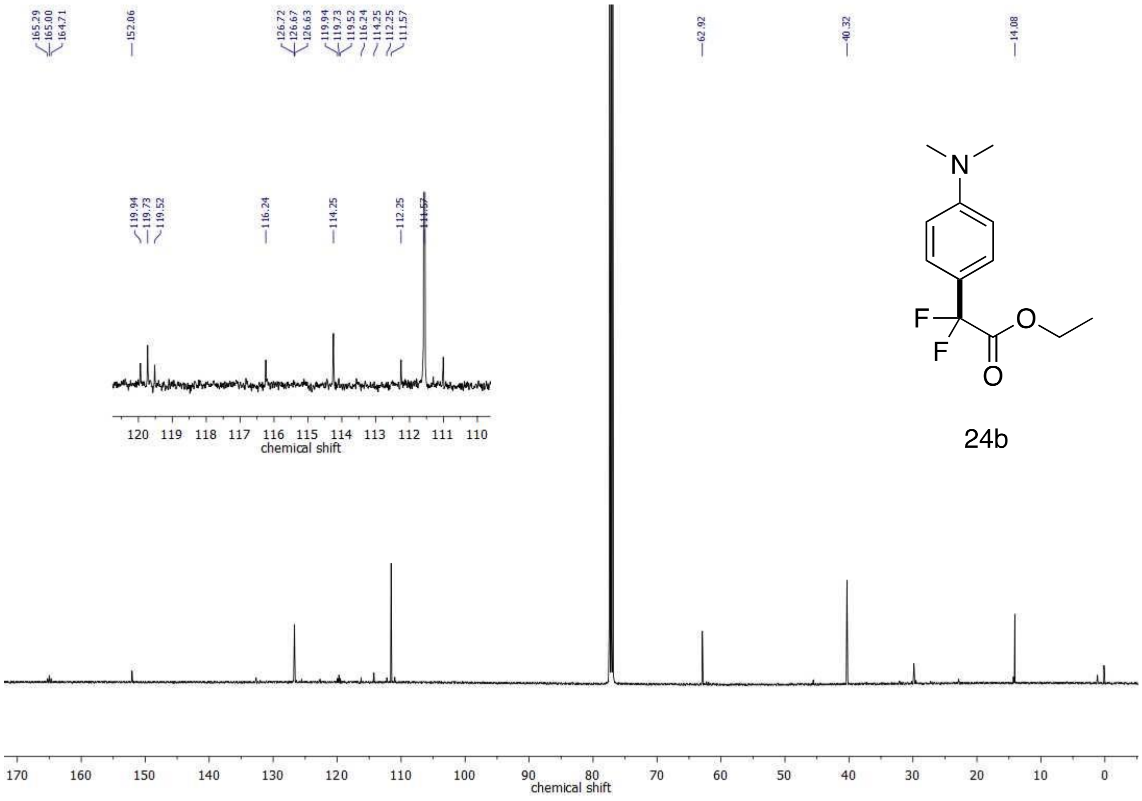

$^{13}C\{^1H\}$ NMR (125 MHz, $CDCl_3$) of compound **24b**

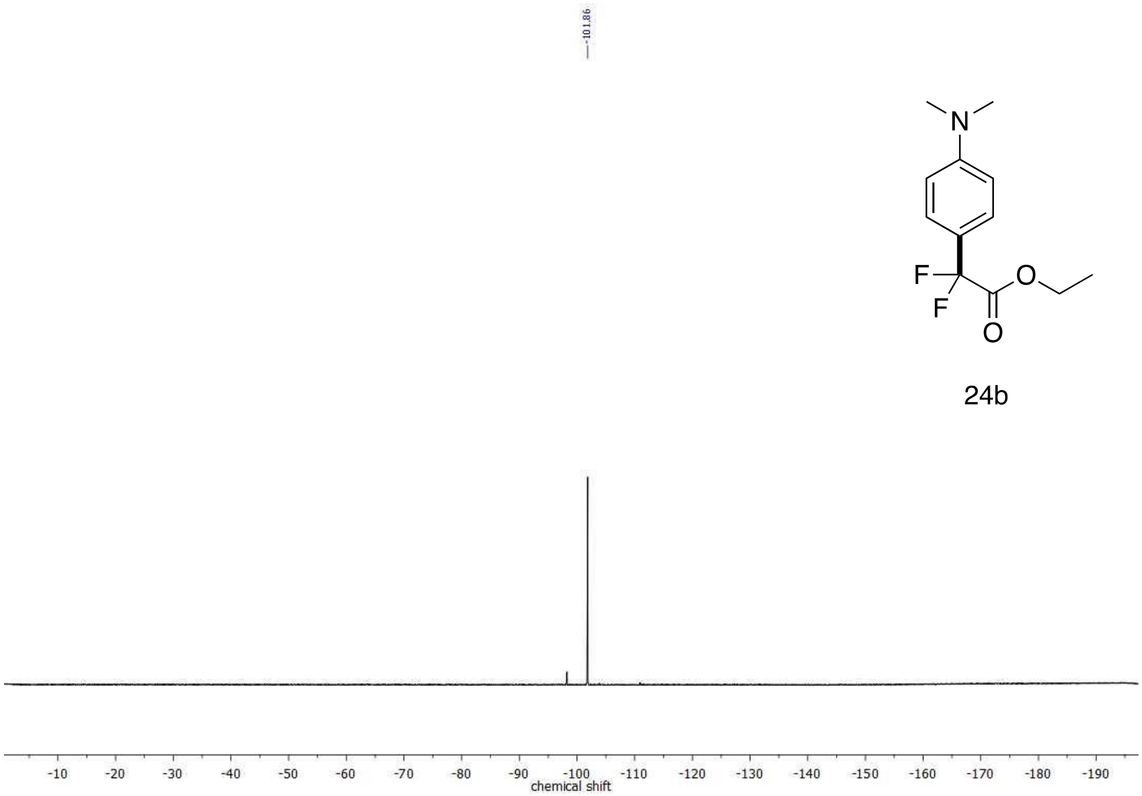

^{19}F NMR (282 MHz, $CDCl_3$) of compound **24b**

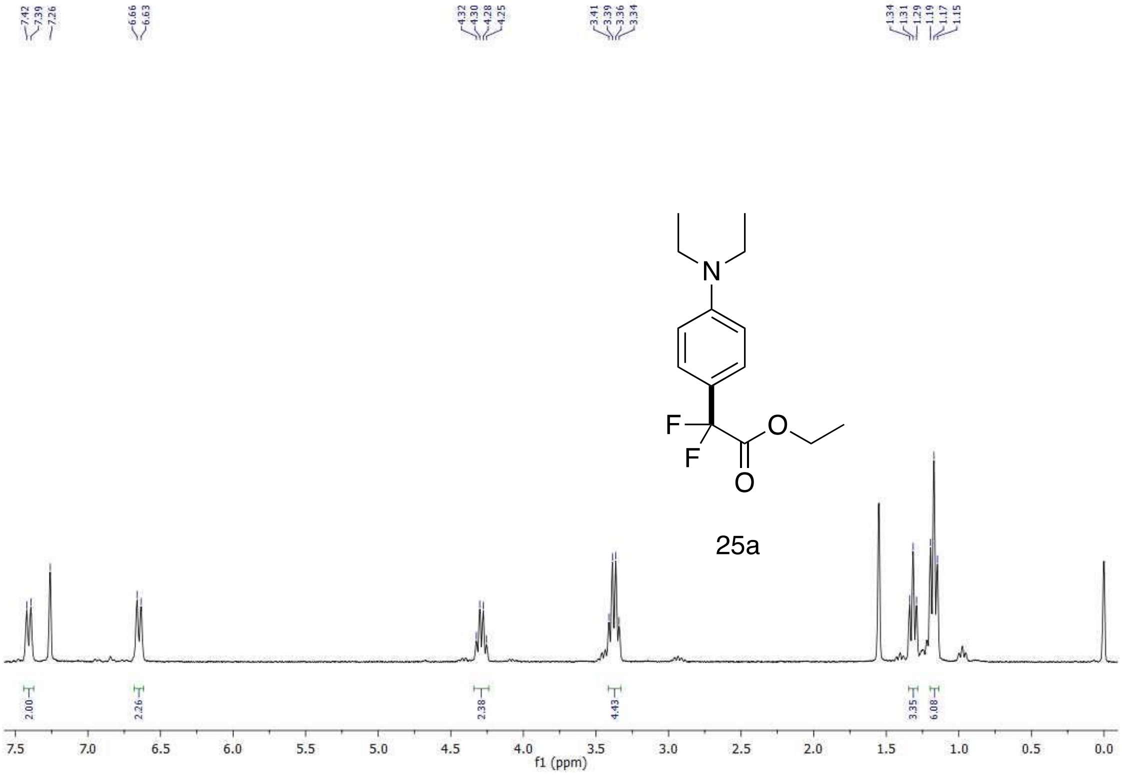

^{1}H NMR (300 MHz, $CDCl_3$) of compound **25a**

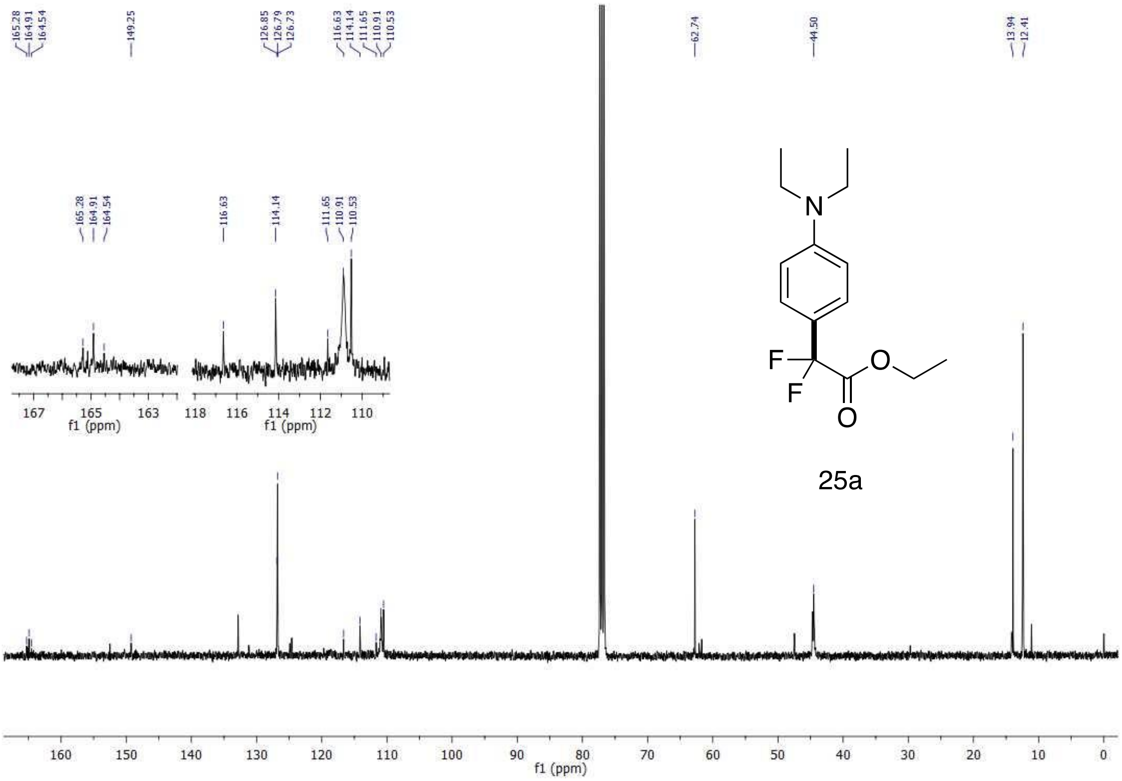

$^{13}C\{^1H\}$ NMR (100 MHz, $CDCl_3$) of compound **25a**

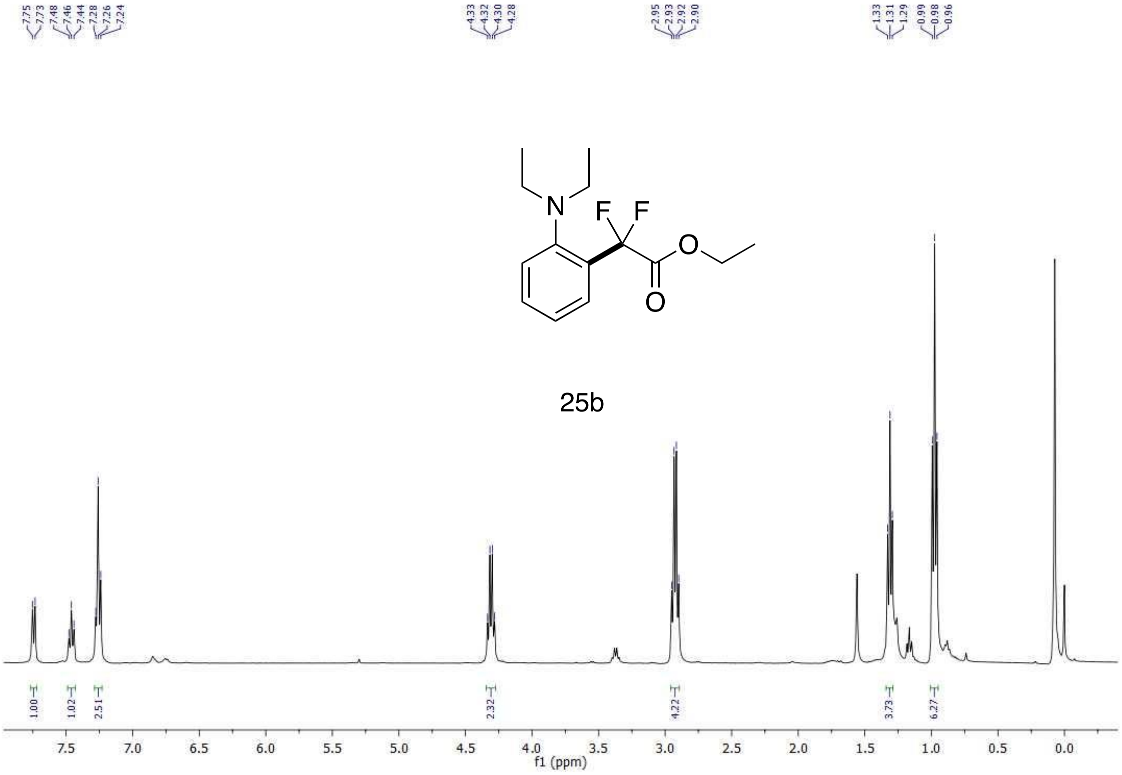

^1H NMR (400 MHz, $CDCl_3$) of compound **25b**

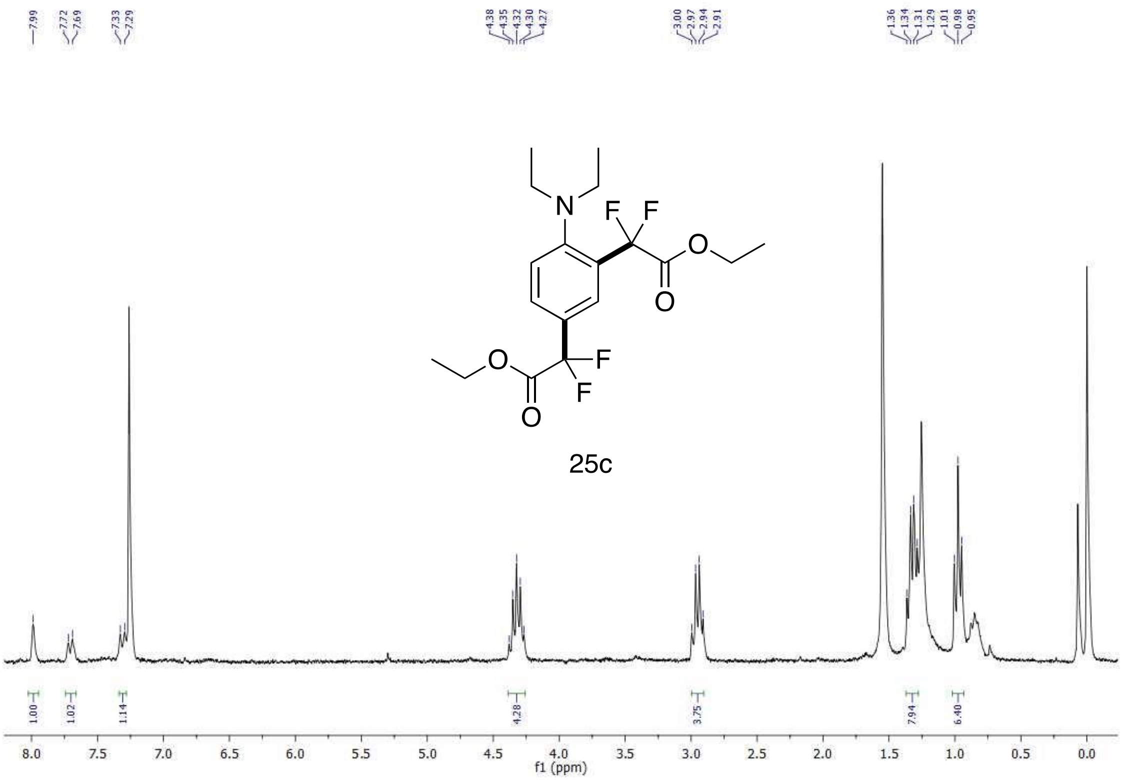

^{1}H NMR (400 MHz, $CDCl_3$) of compound **25c**

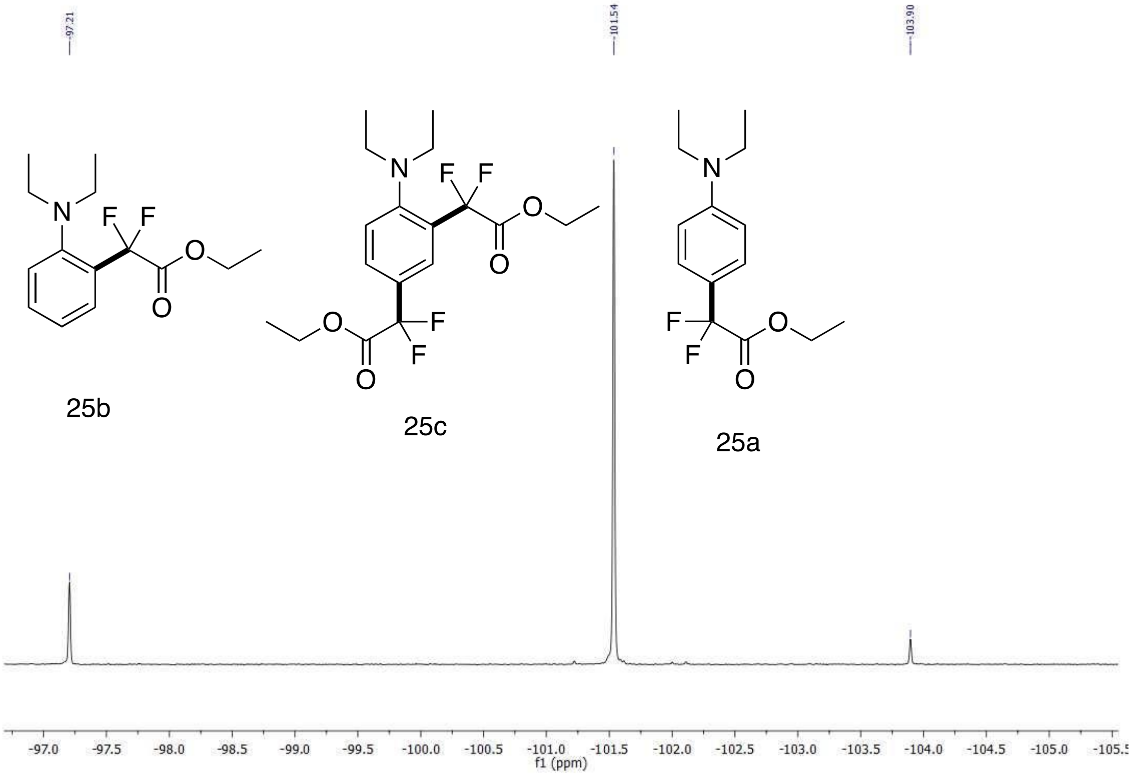

^{19}F NMR (282 MHz, $CDCl_3$) of a mixture of compounds **25a** (-101.54), **25b** (-97.21) and **25c** (-97.21, -103.90)

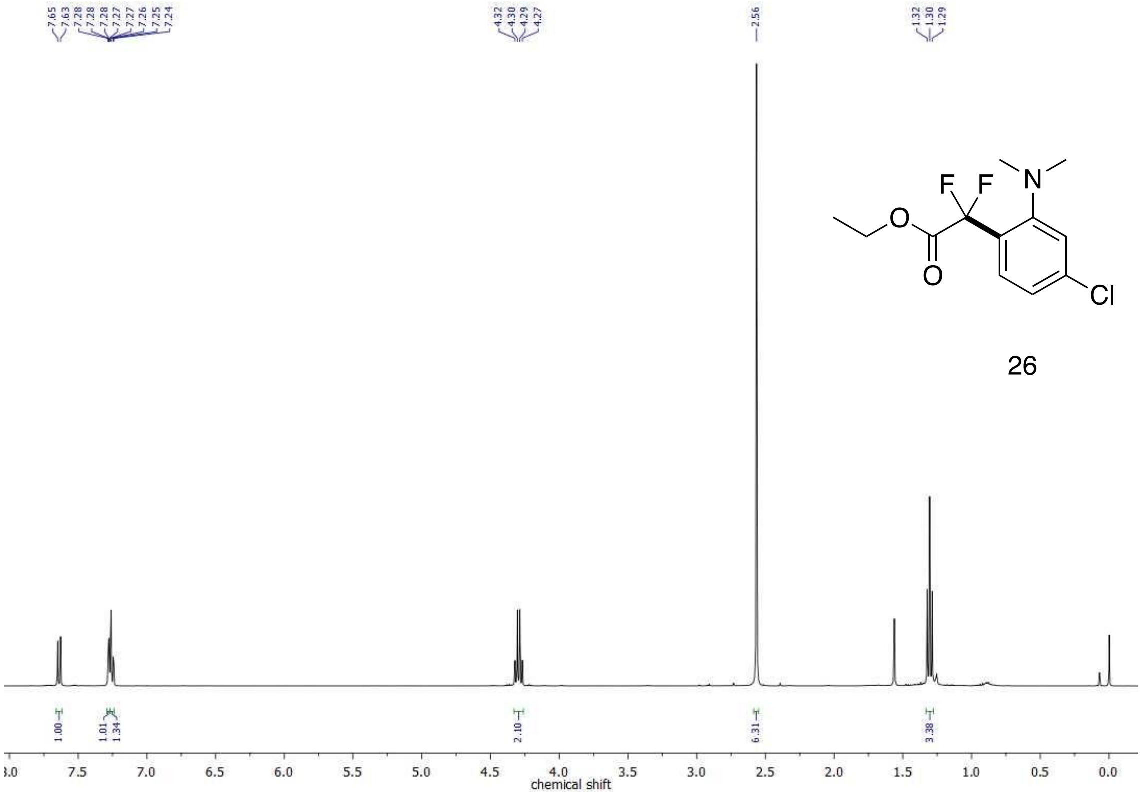

^{1}H NMR (400 MHz, $CDCl_3$) of compound **26**

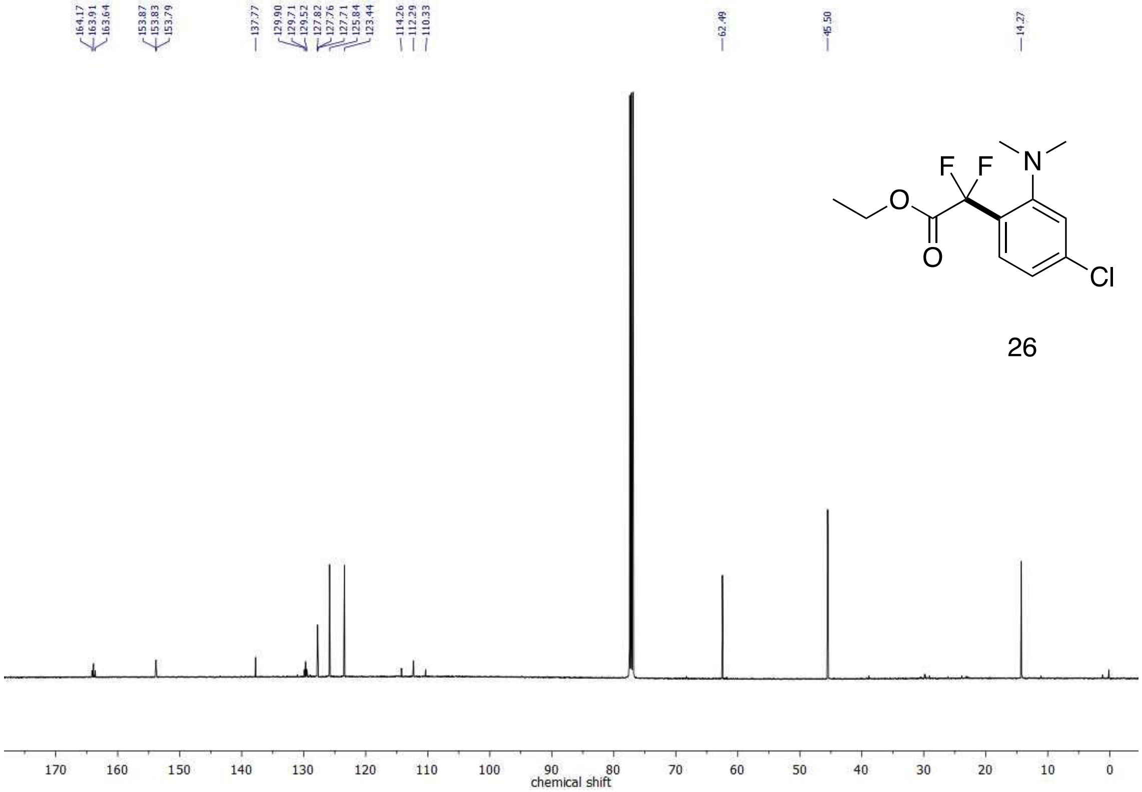

^{13}C{^{1}H} NMR (125 MHz, $CDCl_3$) of compound **26**

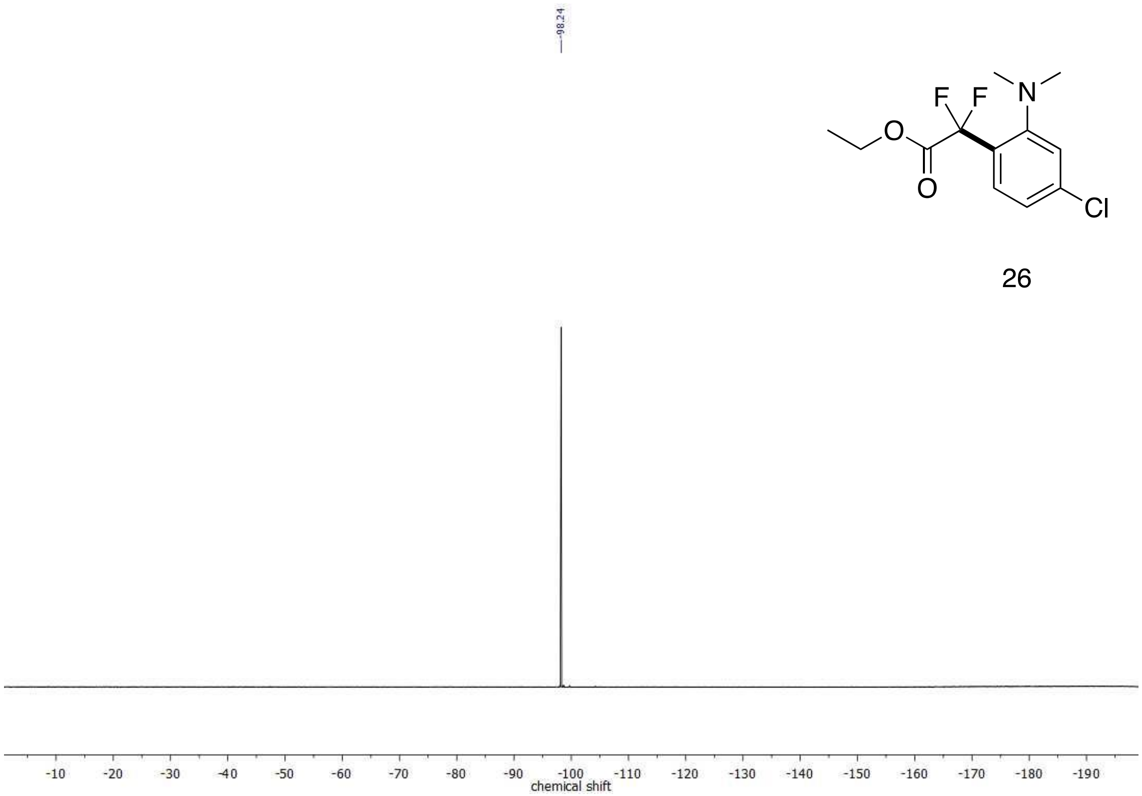

26

^{19}F NMR (376 MHz, $CDCl_3$) of compound **26**

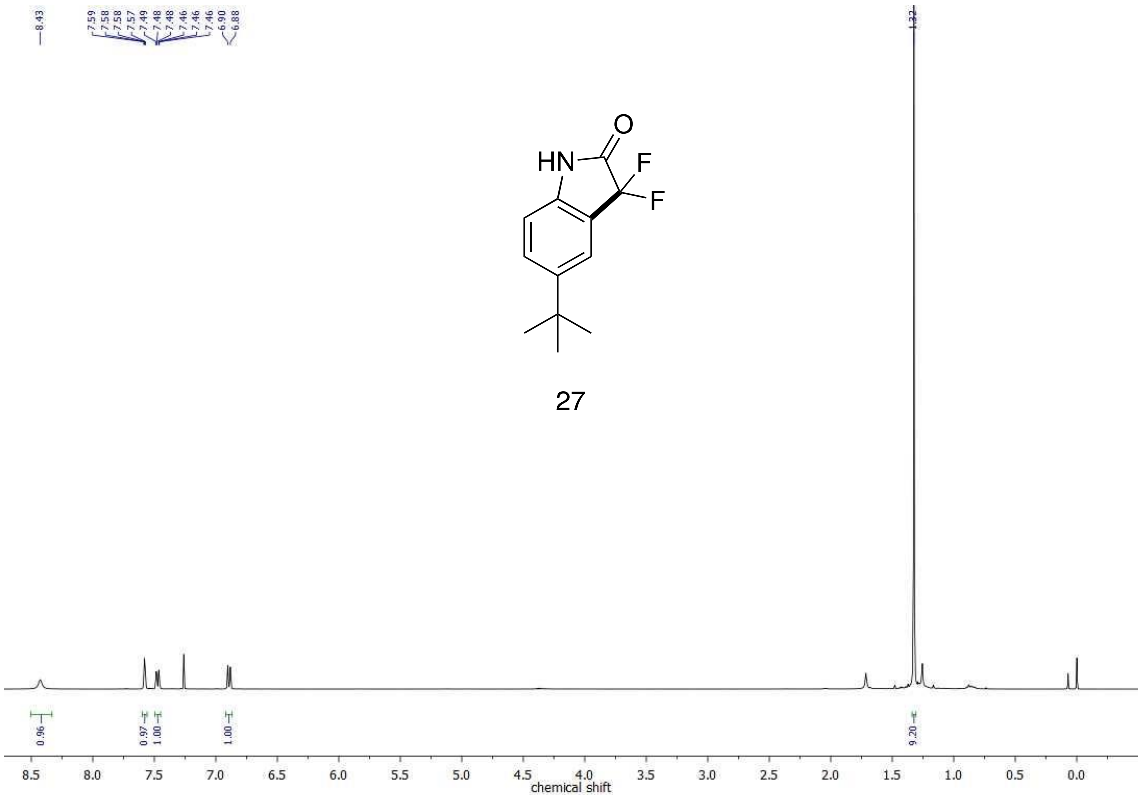

27

^{1}H NMR (400 MHz, $CDCl_3$) of compound **27**

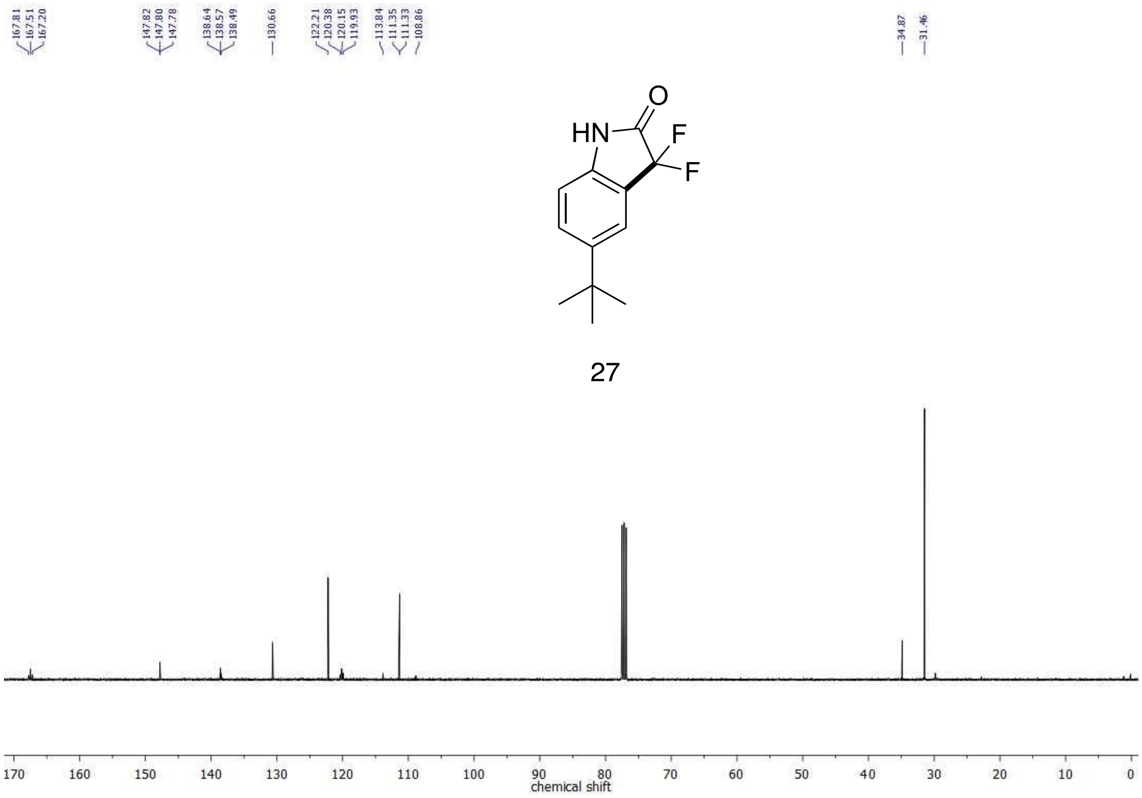

$^{13}C\{^{1}H\}$ NMR (100 MHz, $CDCl_3$) of compound **27**

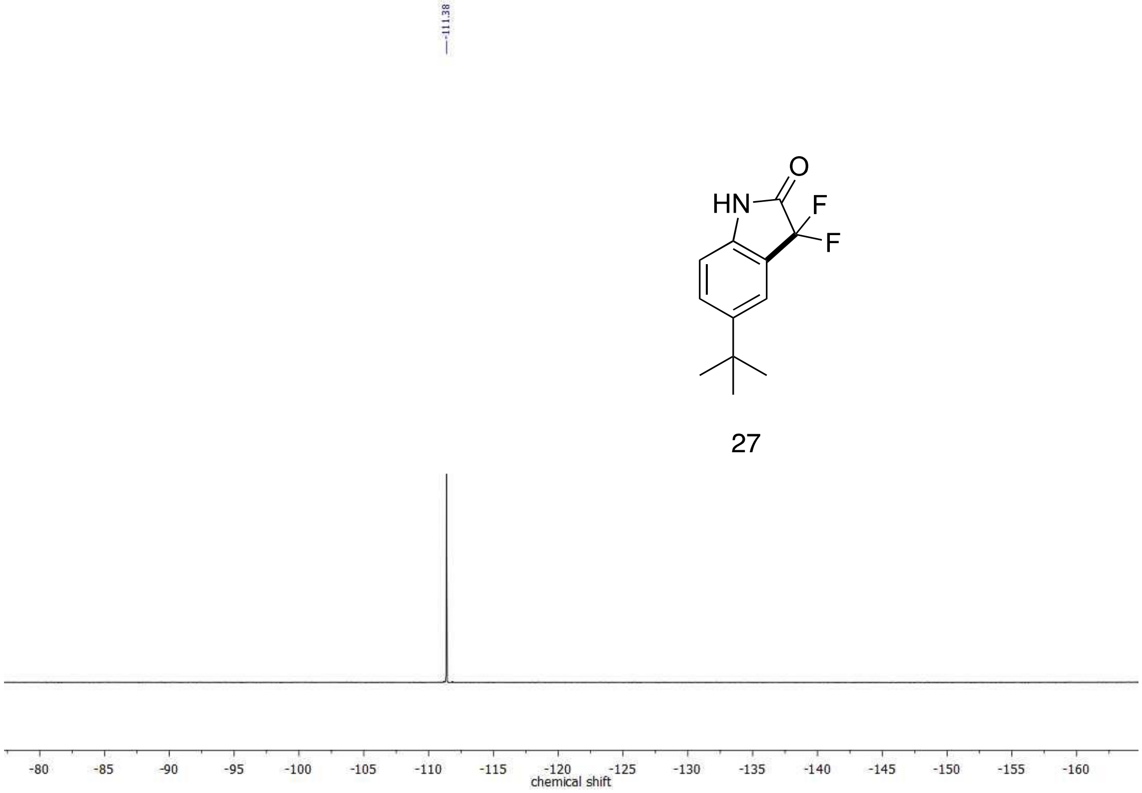

^{19}F NMR (376 MHz, $CDCl_3$) of compound **27**

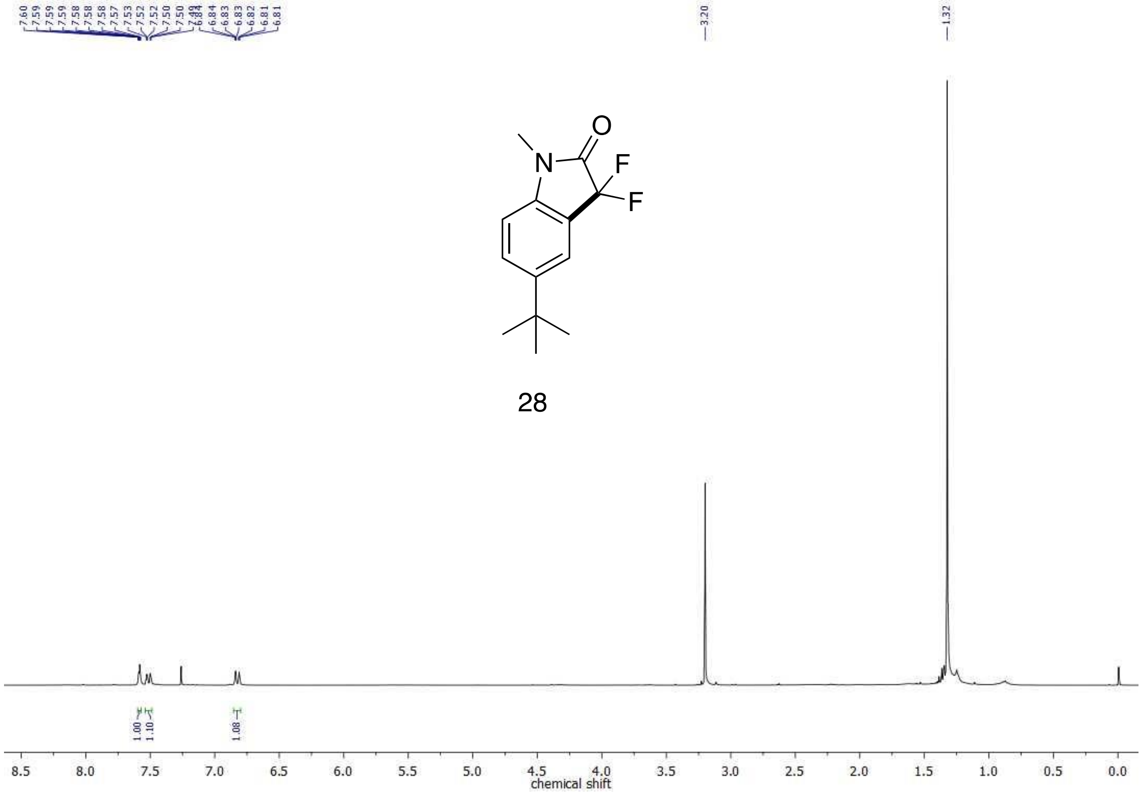

^{1}H NMR (400 MHz, $CDCl_3$) of compound **28**

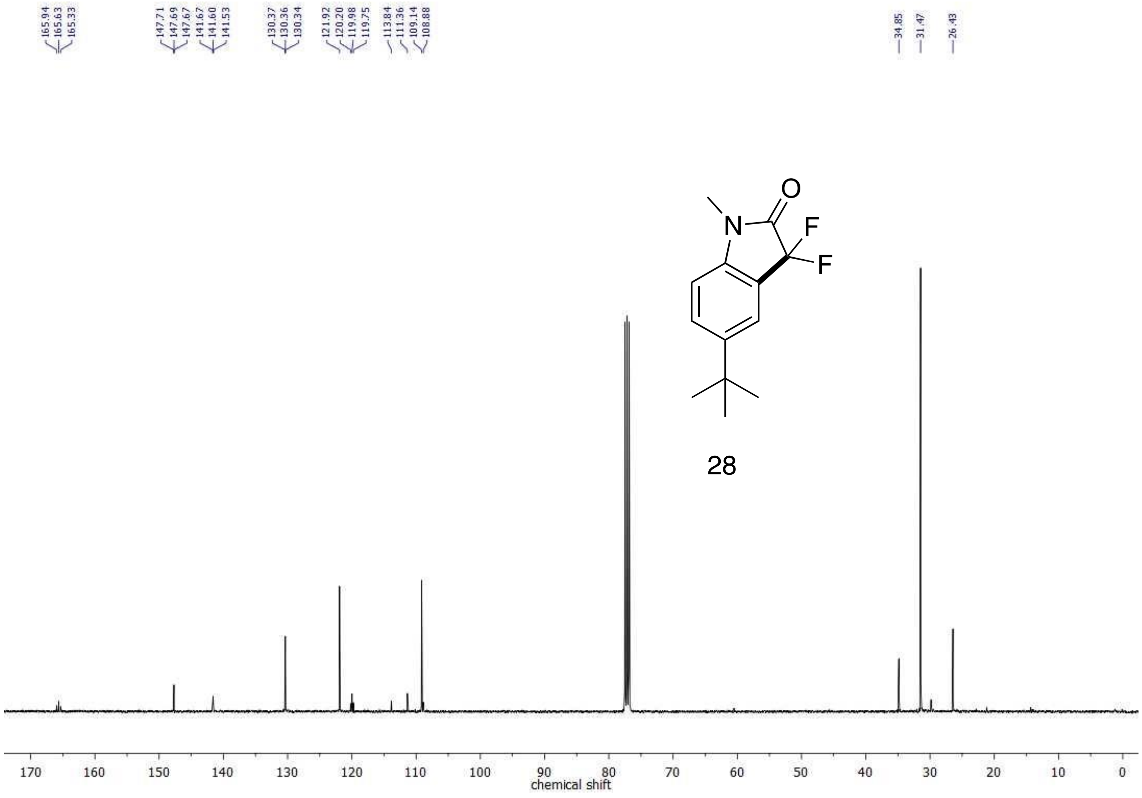

$^{13}C\{^{1}H\}$ NMR (100 MHz, $CDCl_3$) of compound **28**

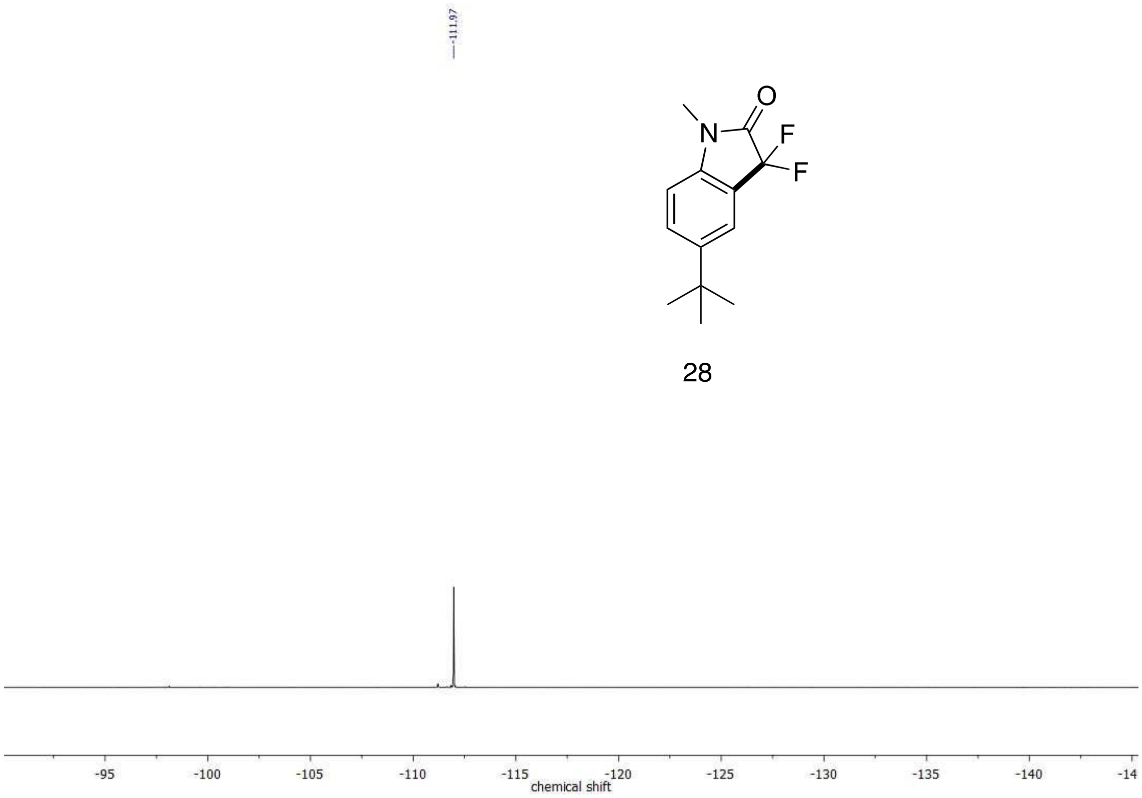

^{19}F NMR (376 MHz, CDCl$_3$) of compound **28**

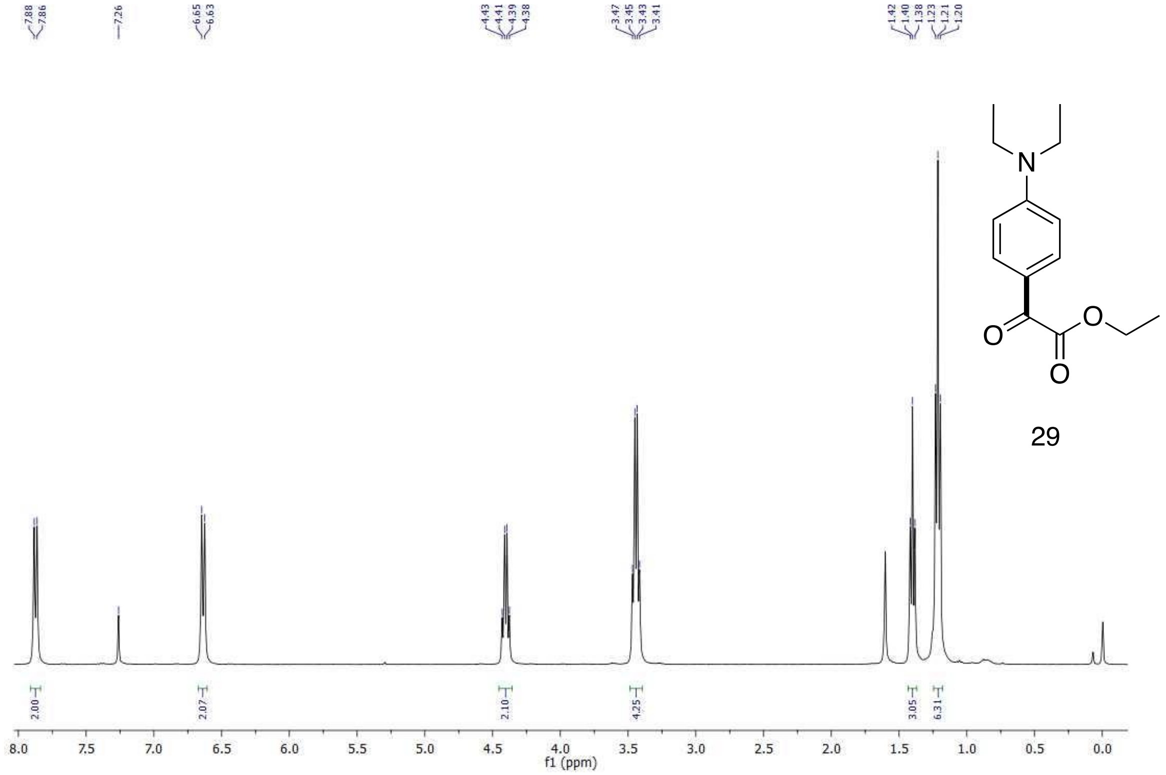

^{1}H NMR (400 MHz, CDCl$_3$) of compound **29**

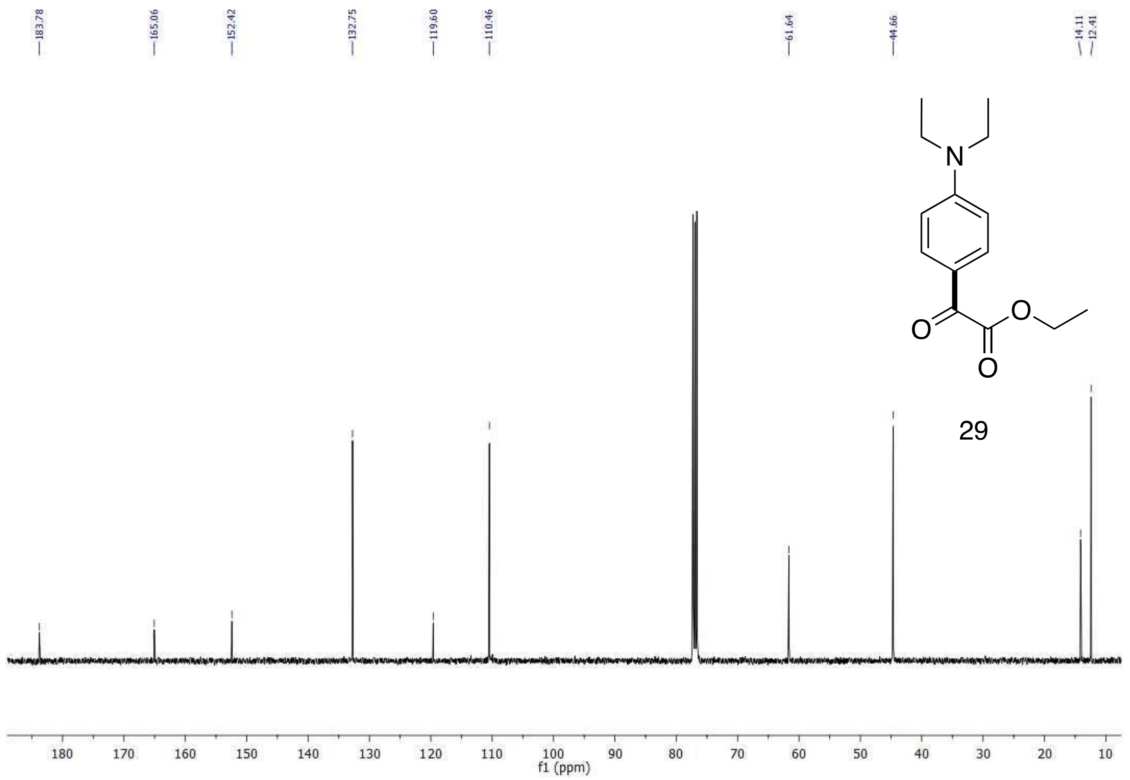

$^{13}C\{^{1}H\}$ NMR (100 MHz, $CDCl_3$) of compound **29**

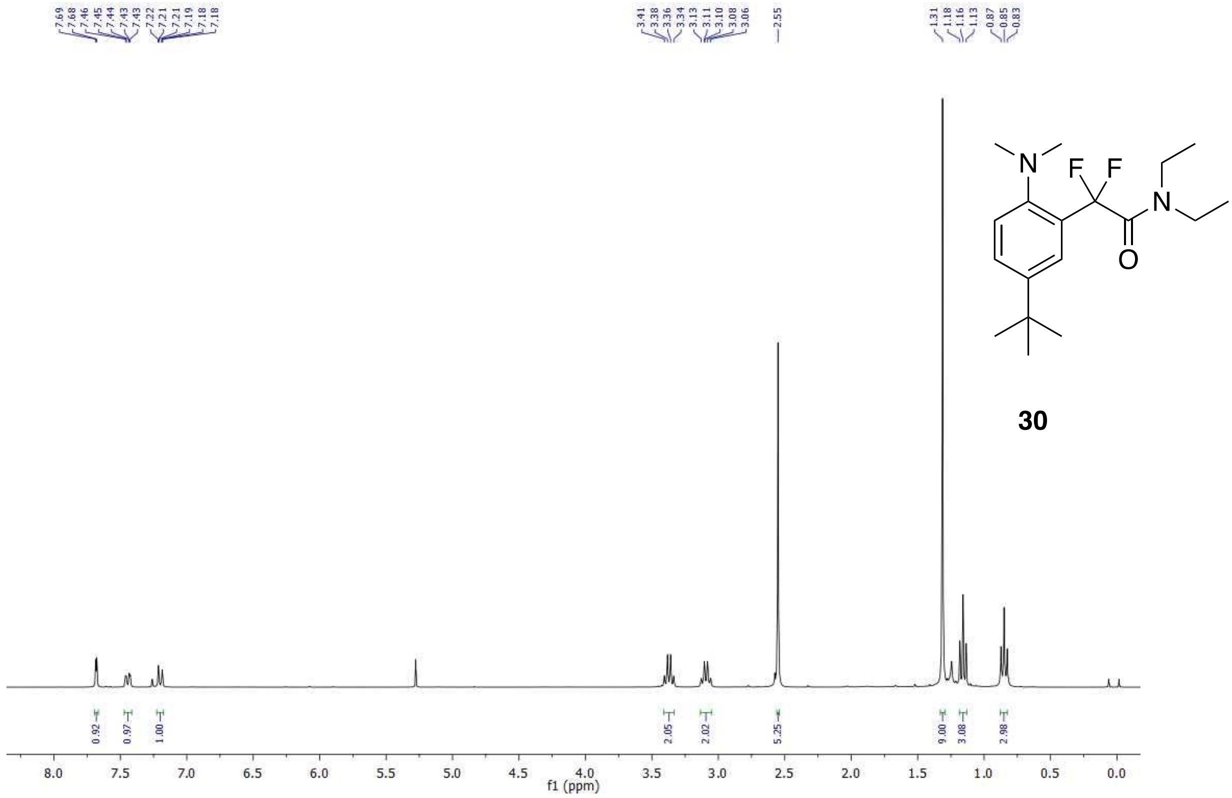

^{1}H NMR (400 MHz, $CDCl_3$) of compound **30**

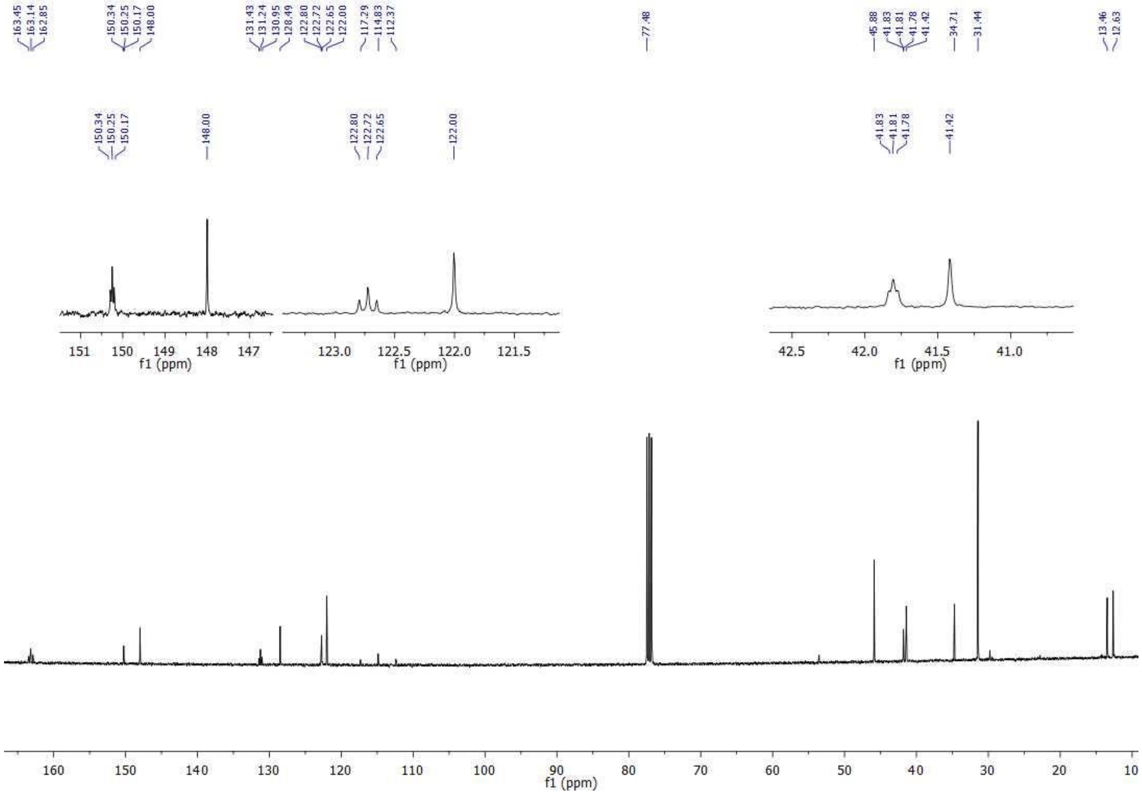

$^{13}C\{^{1}H\}$ NMR (100 MHz, $CDCl_3$) of compound **30**

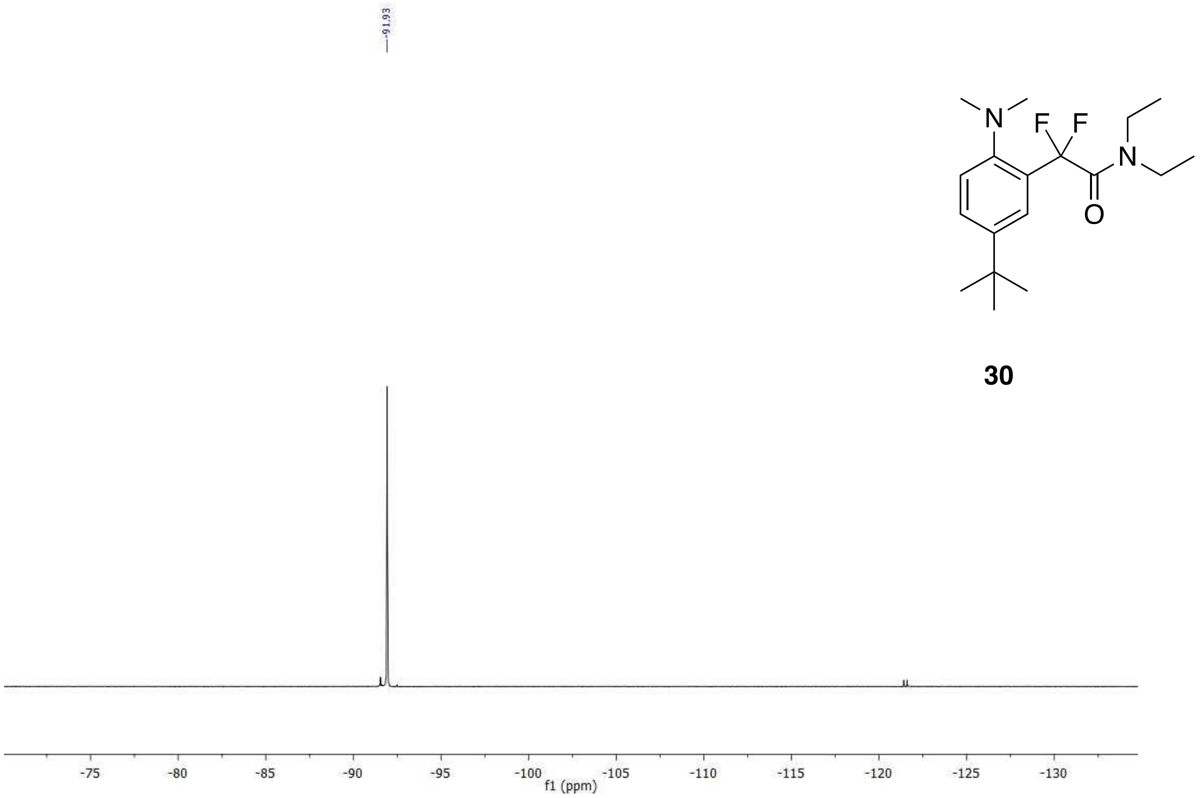

30

^{19}F NMR (376 MHz, $CDCl_3$) of compound **30**

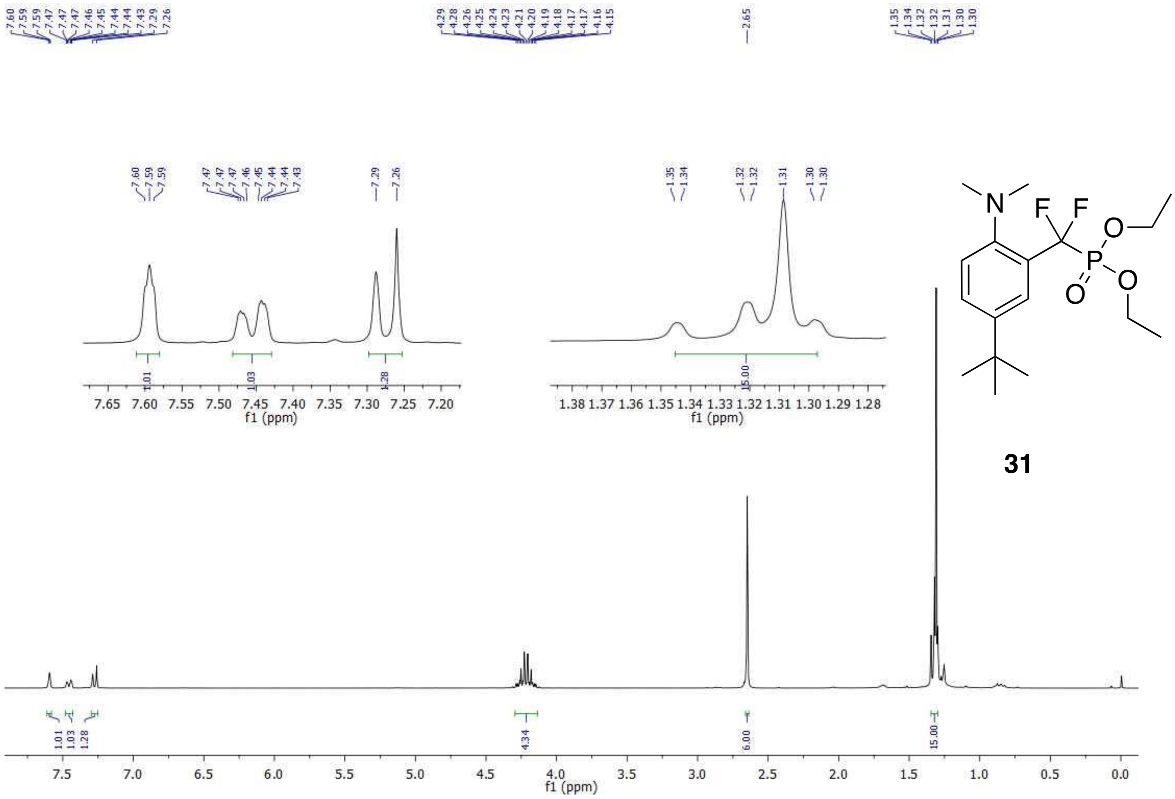

^{1}H NMR (400 MHz, $CDCl_3$) of compound **31**

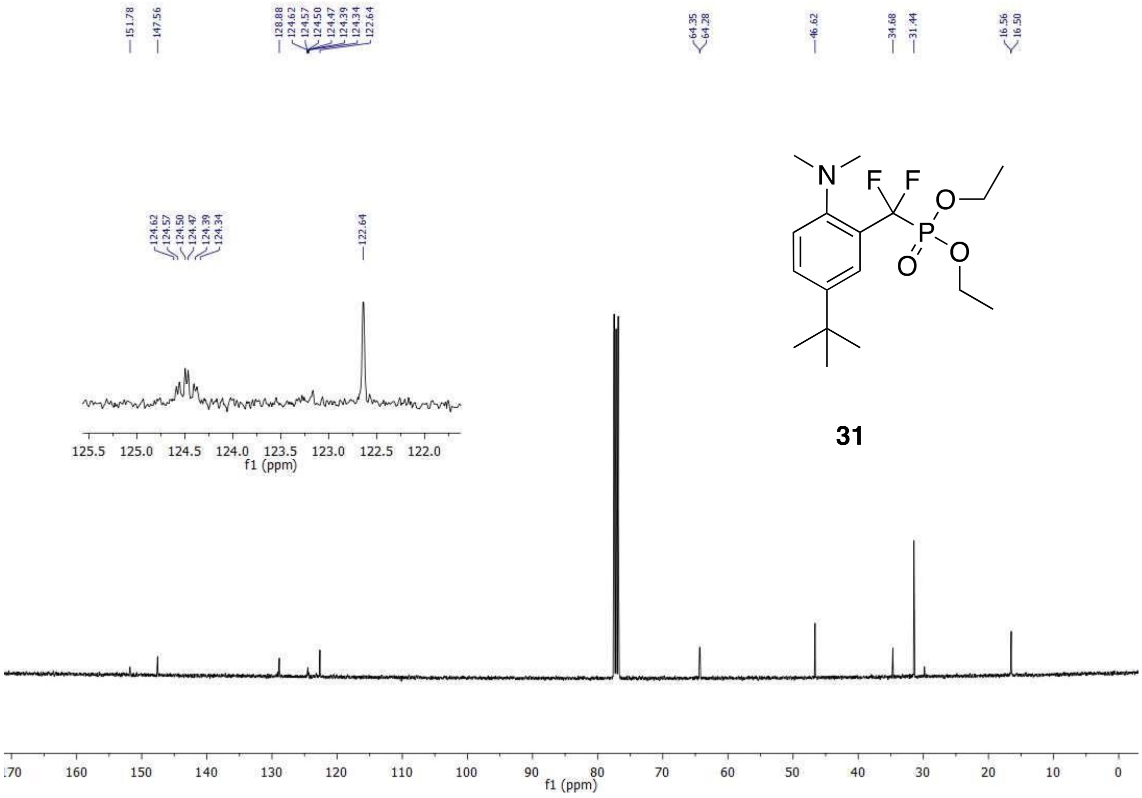

$^{13}C\{^{1}H\}$ NMR (100 MHz, $CDCl_3$) of compound **31**

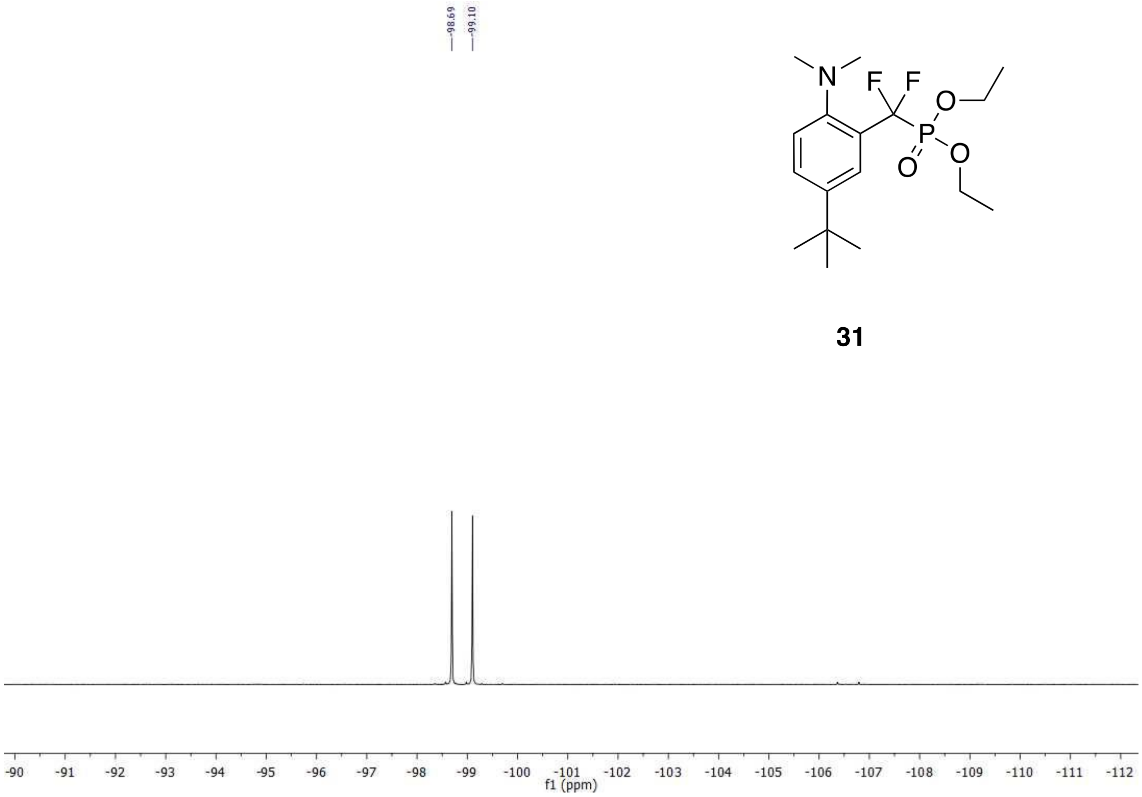

^{19}F NMR (376 MHz, $CDCl_3$) of compound **31**

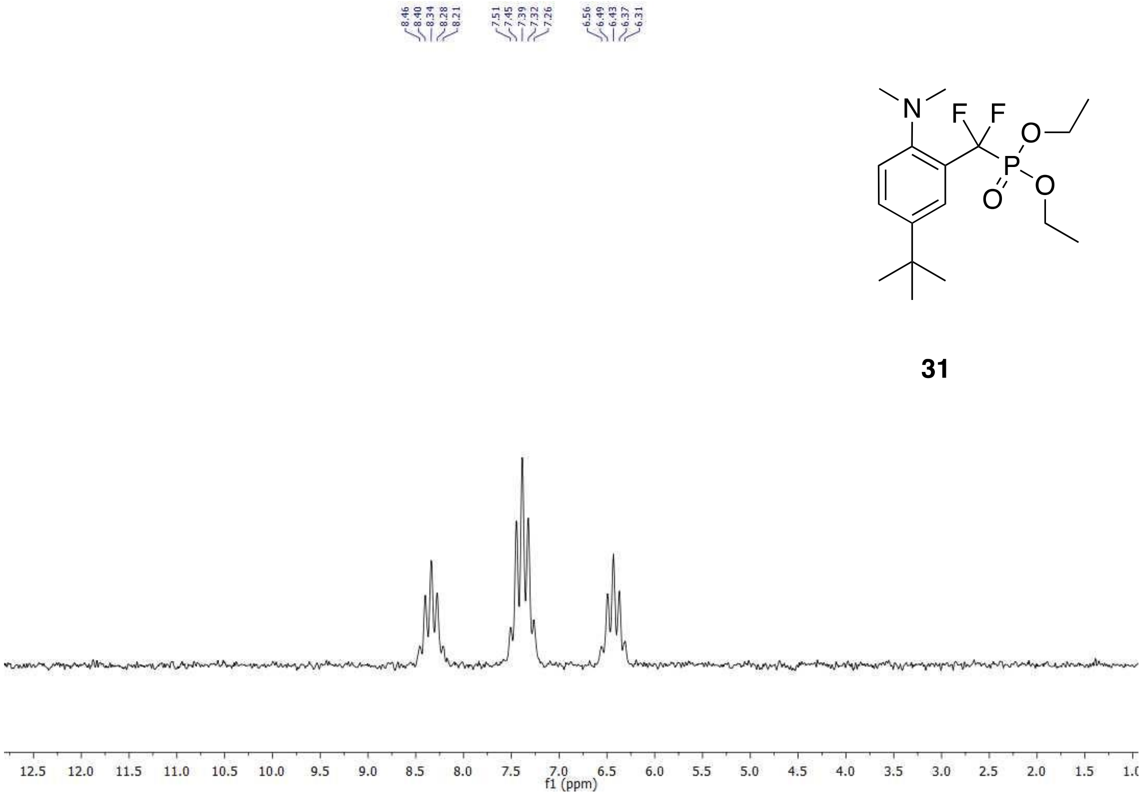

^{31}P NMR (162 MHz, $CDCl_3$) of compound **31**

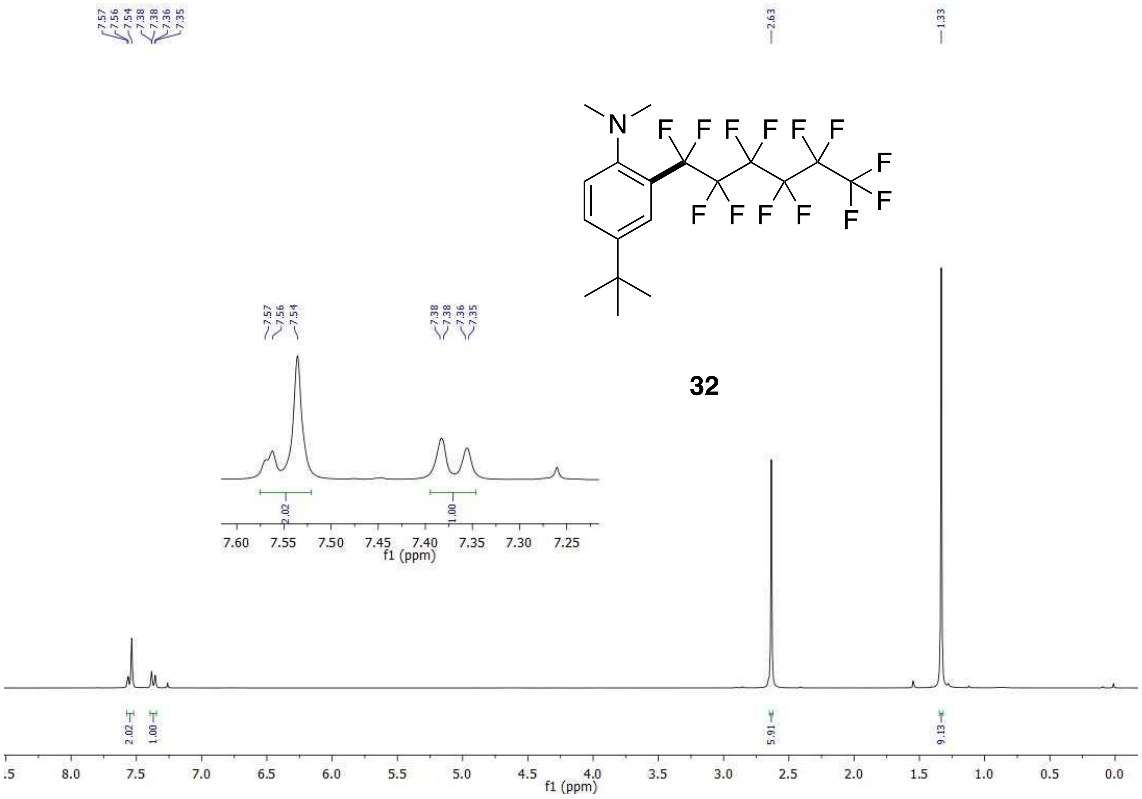

^{1}H NMR (300 MHz, $CDCl_3$) of compound **32**

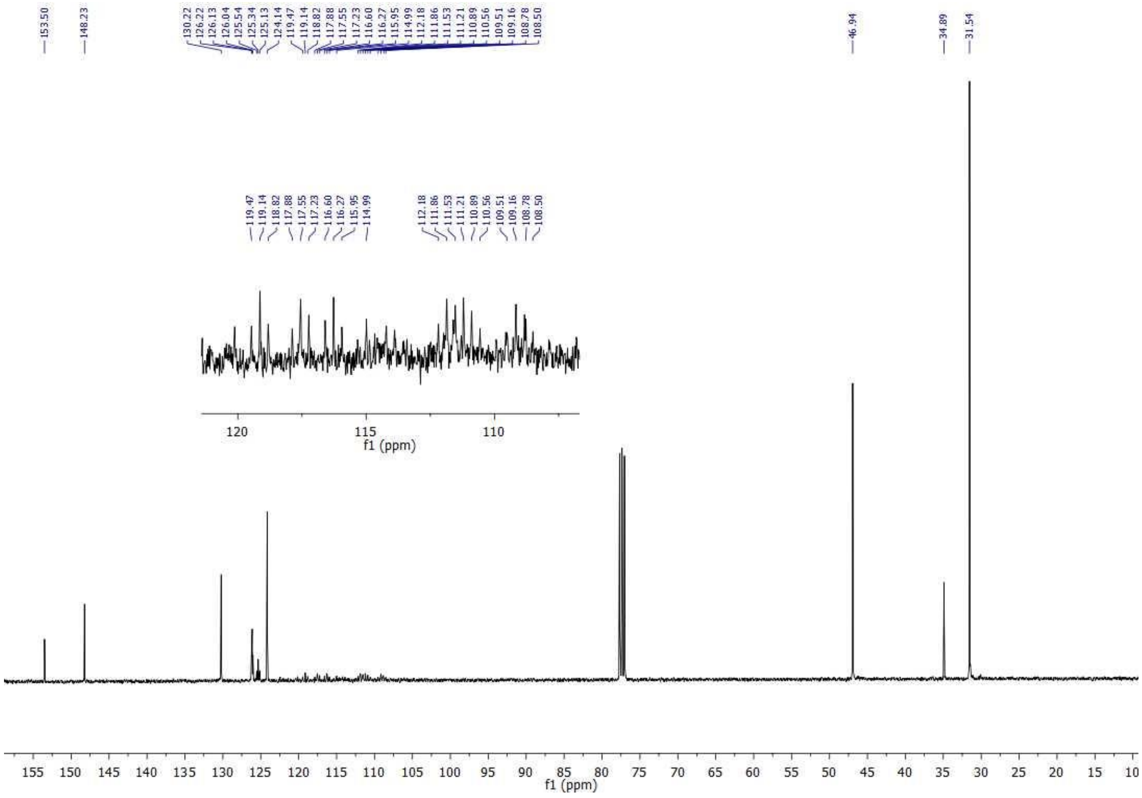

^{13}C{^{1}H} NMR (100 MHz, $CDCl_3$) of compound **32**

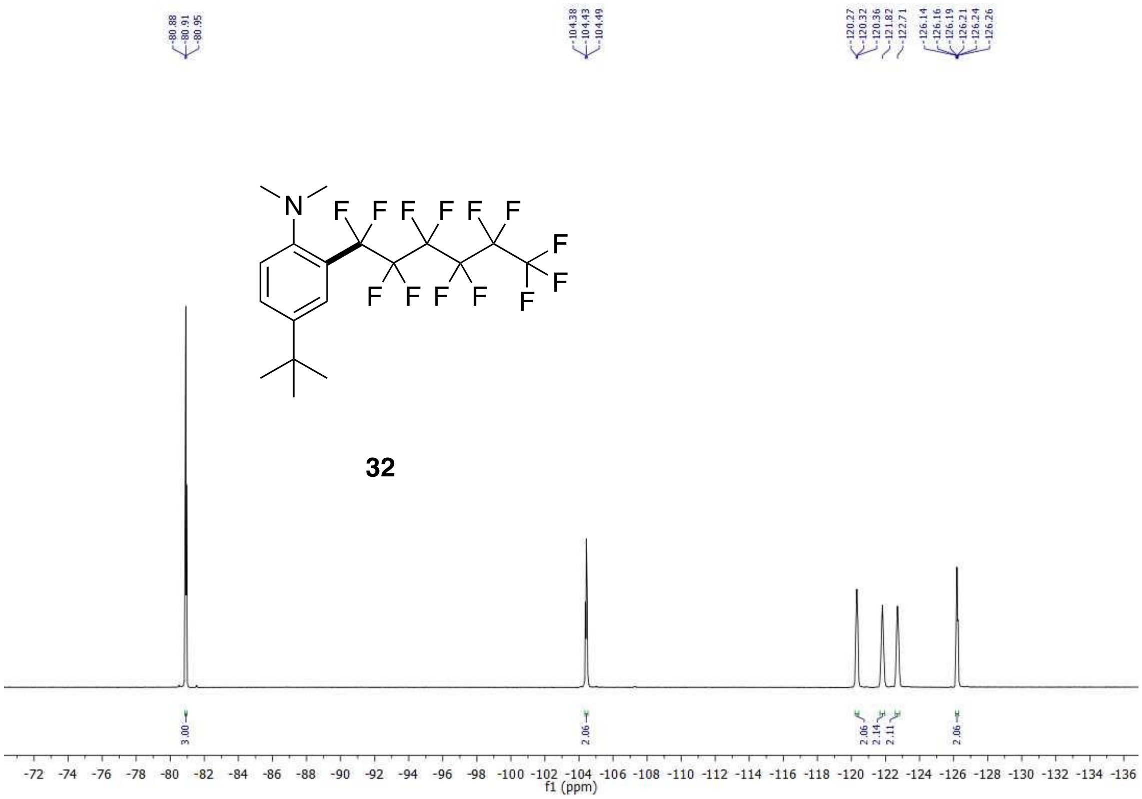

^{19}F NMR (376 MHz, $CDCl_3$) of compound **32**
